# Comparative Evaluation of Chemical and Photolytic Denitrosation Methods for Chemiluminescence Detection of Total *N*-Nitrosamines in Wastewater Samples

**DOI:** 10.1021/acs.est.2c09769

**Published:** 2023-05-04

**Authors:** Changcheng Pu, Teng Zeng

**Affiliations:** Department of Civil and Environmental Engineering, Syracuse University, 151 Link Hall, Syracuse, New York 13244, United States

**Keywords:** *N*-nitroso compounds, NDMA, acidic triiodide, UV photolysis, nitric oxide, denitrosation

## Abstract

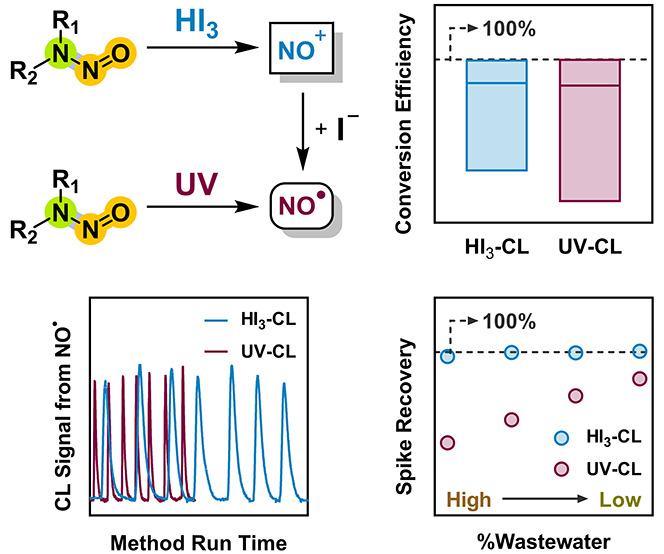

*N*-Nitrosamines form as byproducts during oxidative water treatment and occur as impurities in consumer and industrial products. To date, two methods based on chemiluminescence (CL) detection of nitric oxide liberated from *N*-nitrosamines via denitrosation with acidic triiodide (HI_3_) treatment or ultraviolet (UV) photolysis have been developed to enable the quantification of total *N*-nitrosamines (TONO) in environmental water samples. In this work, we configured an integrated experimental setup to compare the performance of HI_3_-CL and UV-CL methods with a focus on their applicability for TONO measurements in wastewater samples. With the use of a large-volume purge vessel for chemical denitrosation, the HI_3_-CL method achieved signal stability and detection limits comparable to those achieved by the UV-CL method which utilized a microphotochemical reactor for photolytic denitrosation. Sixty-six structurally diverse *N*-nitroso compounds (NOCs) yielded a range of conversion efficiencies relative to *N*-nitrosodimethylamine (NDMA) regardless of the conditions applied for denitrosation. On average, TONO measured in preconcentrated raw and chloraminated wastewater samples by the HI_3_-CL method were 2.1 ± 1.1 times those measured by the UV-CL method, pointing to potential matrix interferences as further confirmed by spike recovery tests. Overall, our comparative assessment of the HI_3_-CL and UV-CL methods serves as a basis for addressing methodological gaps in TONO analysis.

## Abstract 2

Two methods based on the acidic triiodide- and UV photolysis-chemiluminescence (CL) detection of nitric oxide are compared to assess their performance for total *N*-nitrosamine analysis in wastewater samples.

## Introduction

*N*-Nitrosamines are a subgroup of *N*-nitroso compounds (NOCs) characterized by the general structure of R_2_N–N=O and known to occur as mixtures in consumer and industrial products (e.g., cosmetics, tobacco, and rubber additives).^1^ NOCs are of significant public health concern because many of them exhibit mutagenic and carcinogenic activities.^2−4^ To date, fifteen NOCs have been classified by the U.S. Department of Health and Human Services National Toxicology Program as “reasonably anticipated to be human carcinogens” based on evidence from earlier experimental animal studies.^1^ Over the past few years, NOCs have attracted public attention due to the detection of *N*-nitrosamines as impurities in some marketed drugs,^5^ which has not only spurred renewed interest in the assessment of their carcinogenic potential^6−8^ but also led to increased regulatory scrutiny in the drug manufacturing process.^9,10^ Concerns over *N*-nitrosamine exposure, however, have also stemmed from their formation as byproducts during oxidative treatment processes (e.g., chlor(am)ination or ozonation) in drinking water production and wastewater recycling.^11−13^ Currently, *N*-nitrosodimethylamine (NDMA) and five other *N*-nitrosamines are included in the U.S. Environmental Protection Agency (EPA)’s Fifth Contaminant Candidate List.^14^

To complement mass spectrometry-based techniques routinely applied for the analysis of specific *N*-nitrosamines, Kulshrestha et al.^15^ adapted an assay utilizing the acidic triiodide reagent for chemical denitrosation (originally established by Feelisch and colleagues for analyzing *S*-nitrosothiols and nitrosylhemes in biological fluids^16^) to quantify the level of total *N*-nitrosamines (TONO) in recreational water samples by ozone-based chemiluminescence (CL) detection of nitric oxide (NO^•^). Upon treatment by acidic triiodide, *N*-nitrosamines are hypothesized to undergo heterolytic cleavage of the N–NO bond via the formation of a cage-like cyclic transition state with HI_3_ acid followed by elimination of the NO group^17^ or via protolytic denitrosation^18,19^ to release the nitrosonium ion (NO^+^), which can be reduced by iodide to produce NO^•^. Once purged into the reaction cell of a nitric oxide analyzer by an inert carrier gas (e.g., N_2_), NO^•^ further reacts with ozone generated directly from ambient air or pure oxygen to form the excited state nitrogen dioxide (NO_2_*), which emits light in the near-infrared region upon its relaxation to the ground state.^20^ Calibrating the emitted light intensity measured by the photomultiplier tube inside the nitric oxide analyzer with NDMA standard solutions provides a means to quantify TONO in aqueous samples as NDMA equivalents.^15^ Mitch and colleagues have since applied their acidic triiodide-based CL method (hereafter abbreviated as “HI_3_-CL”) to analyze TONO in various environmental water samples, ranging from drinking water and recycled wastewater to stormwater and raw wastewater, and concluded that specific *N*-nitrosamines of current interest only constitute a small fraction of TONO.^21−25^ Nevertheless, earlier work by Hausladen et al. questioned the applicability of the HI_3_-CL method for analyzing NO^•^-releasing compounds in biological matrices due to poor CL signal stability and potential side reactions involving nitrosyl iodide formed in their setup.^26^ To overcome the limitations noted with the HI_3_-CL method, Breider and von Gunten developed an alternative assay utilizing ultraviolet (UV) photolysis for photolytic denitrosation (originally established by Stamler et al. for analyzing *S*-nitrosothiols in mammalian plasma^27^) to measure TONO in aqueous samples.^28^ Upon UV irradiation at 254 nm, *N*-nitrosamines are hypothesized to undergo homolytic cleavage of the N–NO bond to produce NO^•^ and aminium radicals via the dissociation of *N*-nitrosoammonium ions rearranged from protonated the excited state of *N*-nitrosamines.^29,30^ Breider and von Gunten have applied their UV photolysis-based CL method (hereafter abbreviated as “UV-CL”) to analyze TONO in wastewater effluent and greywater samples as well as chloraminated and ozonated personal care product solutions, further highlighting the possible contribution of polymer-bound *N*-nitrosamines to TONO.^28^

Given the promising features of the UV-CL method and the need to reevaluate the HI_3_-CL method for TONO analysis, we assembled an integrated CL setup to enable a direct comparison of the two methods concerning their signal stability, inclusivity, and sensitivity, as well as applicability for wastewater analysis. Our specific objectives of this study were (i) to examine the CL signal repeatability and reproducibility of HI_3_-CL and UV-CL methods, (ii) to assess the inclusivity and sensitivity of HI_3_-CL and UV-CL methods by measuring the conversion efficiencies and detection limits of 66 structurally diverse *N*-nitrosamines, and (iii) to compare the performance of HI_3_-CL and UV-CL methods for TONO measurements in raw and chloraminated wastewater samples preconcentrated by dual-cartridge solid-phase extraction. Our work primarily focused on the methodological evaluation of TONO analysis rather than attempting to demonstrate the superiority of one technique over another.

## Materials and Methods

Chemical suppliers and purities are described in the Supporting Information (Text S1). *N*-Nitrosamine reference standards and isotope-labeled internal standards are listed in Table S1.

### CL System Setup and Operation

**Figure 1:**
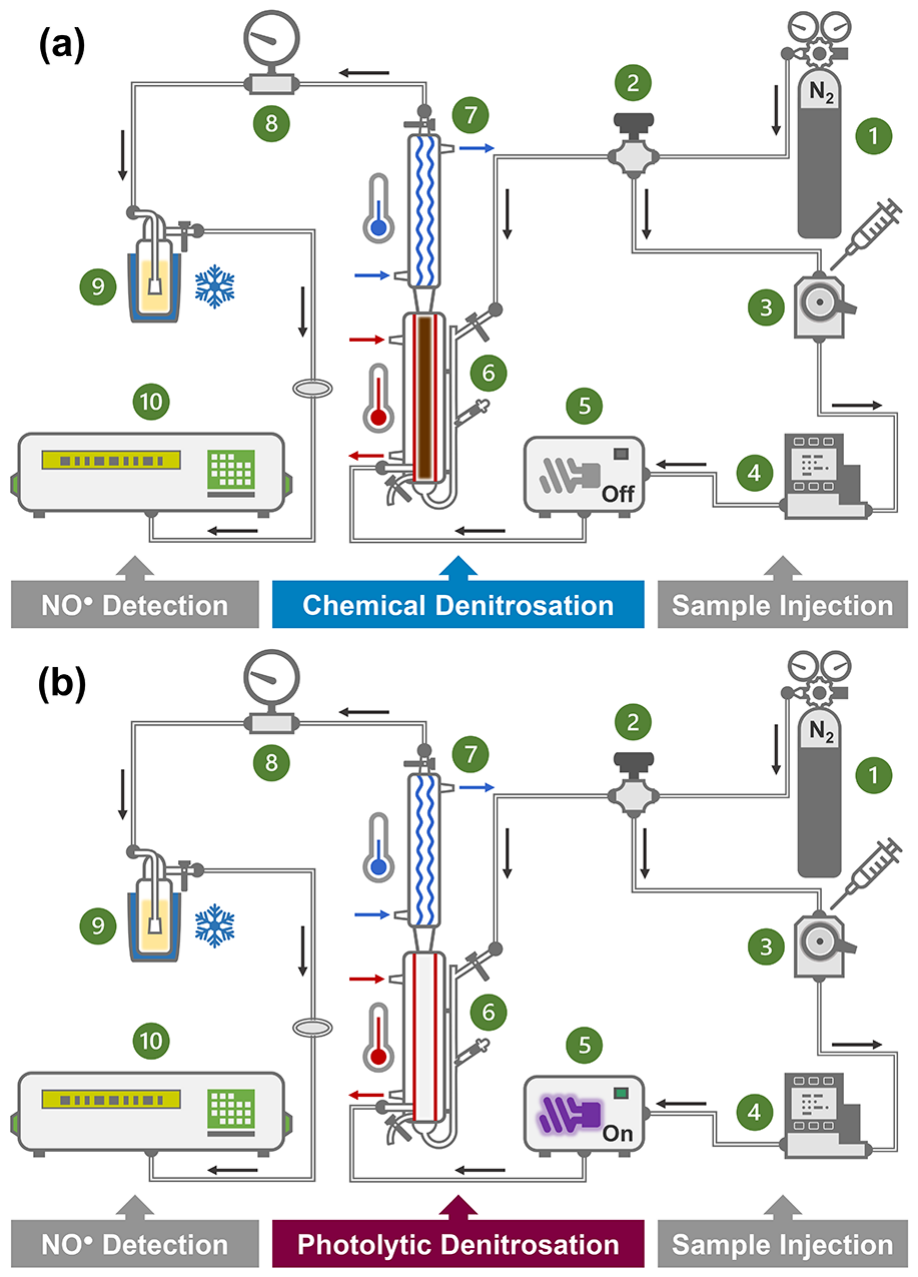
Schematic of the experimental setup assembled for total *N*-nitrosamine analysis using (a) the acidic triiodide-chemiluminescence (HI_3_-CL) method or (b) the UV photolysis-chemiluminescence (UV-CL) method: ① N_2_ cylinder; ② needle valve; ③ manual injector; ④ mass flow controller; ⑤ microphotochemical reactor; ⑥ large-volume purge vessel; ⑦ coil condenser; ⑧ low-pressure gauge; ⑨ base trap; and 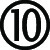 nitric oxide analyzer. Samples were injected via the manual injector (fitted with a 100-μL sample loop) and carried into the knitted reaction coil inside the microphotochemical reactor by ultrahigh-purity N_2_ (black arrows) controlled by a mass flow controller. The microphotochemical reactor was switched on for UV-CL mode and off for HI_3_-CL mode, respectively. Samples leaving the microphotochemical reactor were purged through a bottom injection port into the heated purge vessel (maintained at 80 °C; red arrows), which was constantly flushed with ultrahigh-purity N_2_ regulated by a needle valve. The purge vessel was filled with the acidic triiodide solution for HI_3_-CL mode and acetic acid only for UV-CL mode, respectively. Nitric oxide released from samples via chemical or photolytic denitrosation was then passed through a coil condenser (maintained at 5 °C; blue arrows) and a base trap (cooled with ice) before entering the nitric oxide analyzer for chemiluminescence detection.

Figure 1 presents a schematic of our experimental setup modified from prior work^15,28^ to facilitate a comparative assessment of the HI_3_-CL and UV-CL methods. Typically, 100 μL of *N*-nitrosamine calibration standard solutions or sample extracts were injected from a gas-tight microsyringe (Hamilton) into a Rheodyne Model 7725 injector (IDEX Health & Science) equipped with a 100-μL stainless steel sample loop. Samples were sent through a UVE microphotochemical reactor (Pickering Laboratories) housing a 254 nm low-pressure mercury lamp and a 1-mL knitted reaction coil by ultrahigh-purity N_2_ regulated at 17.5 ± 0.5 psia by an MC-Series 200 mass flow controller (Alicat Scientific) and further transferred into a water-jacketed purge vessel (maintained at 80 °C by a heated circulating bath) surmounted by a coil condenser (maintained at 5 °C by a refrigerated circulating bath operating with a 50/50 water/ethylene glycol mixture). Our purge vessel differed from the commercially available unit used in previous studies^26,28^ in that our vessel featured a significantly larger internal volume (∼55 mL) and a bottom sample injection port. To run the HI_3_-CL method (Figure 1a), the microphotochemical reactor was switched off and the purge vessel was filled with 40 mL of glacial acetic acid plus 4 mL of triiodide solution (freshly prepared by dissolving 2.16 g of potassium iodide and 0.456 g of iodine in 4 mL of deionized water). To run the UV-CL method (Figure 1b), the microphotochemical reactor was switched on and the purge vessel was filled with 40 mL of glacial acetic acid plus 4 mL of deionized water to match the liquid volume maintained in the HI_3_-CL method. For comparison, the commercially available purge vessel (Zysense) was also used for method evaluation. Throughout each analytical run, the solution inside the purge vessel was continuously deoxygenated with ultrahigh-purity N_2_ regulated at 0.2 L/min by a stainless steel needle valve (Swagelok). NO^•^ liberated from chemical or photolytic denitrosation was purged from the purge vessel sequentially through the coil condenser, a base trap (containing 1 N of NaOH solution) held within a Dewar flask of ice, a low-pressure gauge (to maintain an analyzer inlet pressure of −0.5 ± 0.5 psi), and 0.22-μm polyvinylidene difluoride syringe filters into the reaction cell of a CLD 88Yet NO/NO_*x*_ analyzer (EcoPhysics) equipped with a vacuum pump, a thermal ozone scrubber, a molybdenum converter, and an external stainless steel restrictor. Within the reaction cell, NO^•^ reacted with ozone to form NO_2_*, which, upon its relaxation to the ground state, emitted a photon detected by a photomultiplier tube. CL signals were monitored in real-time by a *Microsoft Excel* macro program and processed by *ACD/Labs Spectrus Processor 2021.2.2* for peak area integration. Quantification of TONO was performed by comparing the peak areas of samples to those of NDMA calibration standards measured within the same run. To compare the performance of the HI_3_-CL and UV-CL methods, the conversion efficiencies of *N*-nitrosamines and their equal-molar mixture to NO^•^ were determined by dividing the slopes of their 8-point calibration curves by that of the calibration curve of NDMA in the concentration range of 0.1–5.0 μM. The limits of detection (LODs) and limits of quantification (LOQs) were calculated as 3.3 and 10 times the ratios of the standard deviations of *y*-intercepts to the slopes of *N*-nitrosamine calibration curves, respectively (Section S3).

### Solid-Phase Extraction Protocol

Prior to TONO measurements, samples were preconcentrated by a dual-cartridge SPE protocol adapted from Krauss and Hollender.^31^ Surface-modified polymeric styrene-divinylbenzene copolymer cartridges (Strata-X 33 μm, 200 mg/6 mL; Phenomenex) were conditioned with pentane (2 mL), ethyl acetate (2 mL), methanol (5 mL × 2), and deionized water (5 mL × 2). Coconut-shell activated carbon cartridges (Enviro-Clean 521, 2000 mg/15 mL; United Chemical Technologies) were conditioned with pentane (5 mL), ethyl acetate (5 mL), methanol (5 mL × 2), and deionized water (10 mL × 2). SPE cartridges were connected (with Strata-X cartridges on the top) and mounted onto a glass block vacuum manifold system equipped with a vacuum gauge and bleed valve. Samples (250 mL in duplicate) were transferred from volumetric flasks through preconditioned cartridges at a flow rate of ∼5 mL/min under vacuum. SPE cartridges were then rinsed with 5 mL of deionized water, disconnected and dried separately under vacuum, and reconnected and eluted with 15 mL of dichloromethane into glass collection tubes (i.e., 5 mL for the reconnected dual cartridges followed by 10 mL for Enviro-Clean 521 cartridges after removing Strata-X cartridges). SPE extracts were dried over anhydrous sodium sulfate, transferred into pear-shaped flasks containing 0.8 mL of methanol:water mixture (95:5 v/v), and concentrated using an R-100 rotary evaporator (BUCHI) at 40 °C under a vacuum of 240 mbar until dichloromethane was completely removed to avoid interfering signals in CL detection.^32^ SPE extracts were further reconstituted with methanol:water to 1 mL and stored in amber autosampler vials at −20 °C until analysis. With this protocol, the recoveries of *N*-nitrosamines were determined in drinking water as well as wastewater samples to establish the performance baseline of SPE. To ensure analytical specificity, target quantification of specific *N*-nitrosamines in SPE recovery tests was performed by isotope dilution liquid chromatography-high-resolution mass spectrometry (LC-HRMS) using a Thermo Fisher Scientific TriPlus RSH autosampler and liquid handling system hyphenated with a Vanquish Horizon ultra-high-performance liquid chromatograph and an Orbitrap Exploris 240 quadrupole-Orbitrap mass spectrometer (Section S6).

### Wastewater Sample Collection and Analysis

Wastewater samples (i.e., 24-h flow-proportional composite samples) selected for method evaluation were collected from six full-scale wastewater treatment plants (WWTPs) employing suspended or attached growth systems for BOD_5_ removal and/or nitrification, chemical precipitation for phosphorus removal, and chlorination or UV for seasonal disinfection. Upon return to the laboratory, samples were vacuum filtered sequentially through 2.7-μm and 0.7-μm glass fiber filters. Selected samples were further treated with 2 mM of preformed monochloramine and stored in amber glass bottles in the dark for 7 days at 21 ± 1 °C to promote the conversion of chloramine-reactive precursors to TONO. Sample aliquots (250 mL in duplicate) were then treated with 1.1 mM of l-ascorbic acid to quench chloramine residuals followed by 20 mM of sulfamic acid to eliminate nitrite,^21^ preconcentrated following the SPE protocol described above, and analyzed by the HI_3_-CL and UV-CL methods. Quantification of TONO was performed by injections of 100-μL SPE extracts along with NDMA calibration standards and method blanks.

### Data Analysis

Graphical and basic statistical analyses (e.g., Spearman’s correlation analysis) were performed using *GraphPad Prism 8.4*. Elastic net regression, bidirectional stepwise regression, and best subsets regression analyses (Section S5) were performed sequentially using the *glmnet*(33) and *olsrr*(34) packages in *R 4.2.1*. Gaussian error propagation was applied to account for uncertainties associated with replicate analyses when applicable.

## Results and Discussion

### CL Signal Repeatability and Reproducibility

**Figure 2:**
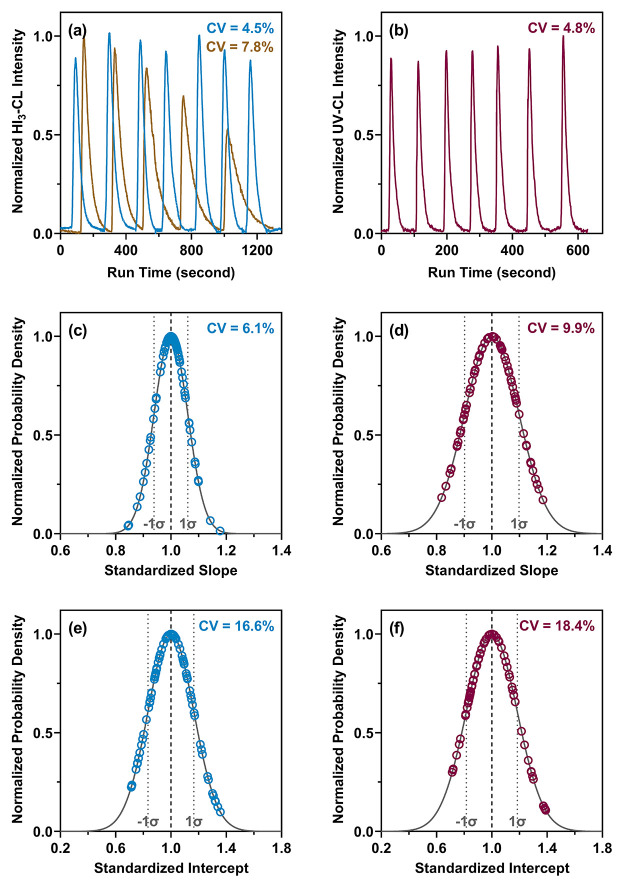
Comparison of the chemiluminescence signal stability for the acidic triiodide-chemiluminescence (HI_3_-CL) and UV photolysis-chemiluminescence (UV-CL) methods via intraday and interday injections of *N*-nitrosodimethylamine (NDMA) calibration standards: (a) Chemiluminescence peaks resulting from seven consecutive injections of a 1-μM NDMA standard solution with the HI_3_-CL method that utilized the large-volume purge vessel (blue line) and the small-volume purge vessel (brown line), respectively, for chemical denitrosation. CV represents the coefficient of variation of chemiluminescence peak areas. (b) Chemiluminescence peaks resulting from seven consecutive injections of a 1-μM NDMA standard solution with the UV-CL method that utilized the microphotochemical reactor for photolytic denitrosation. CV represents the coefficient of variation of chemiluminescence peak areas. (c) Probability distribution of the standardized slopes of NDMA standard curves measured by the HI_3_-CL method over the study period. The gray solid line represents the Gaussian distribution fit of standardized slopes derived from the injections of 80 sets of NDMA calibration standards (0.1, 0.2, 0.5, 1.0, 1.5, 2.0, 3.0, and 5.0 μM). The gray dotted lines mark one standard deviation (±1σ) from the mean. CV represents the coefficient of variation of standardized slopes. (d) Probability distribution of the standardized slopes of NDMA standard curves measured by the UV-CL method over the study period. The gray solid line represents the Gaussian distribution fit of standardized slopes derived from the injections of 73 sets of NDMA calibration standards (0.1, 0.2, 0.5, 1.0, 1.5, 2.0, 3.0, and 5.0 μM). The gray dotted lines mark one standard deviation (±1σ) from the mean. CV represents the coefficient of variation of standardized slopes. (e) Probability distribution of the standardized intercepts of NDMA standard curves measured by the HI_3_-CL method over the study period. The gray solid line represents the Gaussian distribution fit of standardized intercepts derived from the injections of 80 sets of NDMA calibration standards (0.1–5.0 μM). The gray dotted lines mark one standard deviation (±1σ) from the mean. CV represents the coefficient of variation of standardized intercepts. (f) Probability distribution of the standardized intercepts of NDMA standard curves measured by the UV-CL method over the study period. The gray solid line represents the Gaussian distribution fit of standardized intercepts derived from the injections of 73 sets of NDMA calibration standards (0.1–5.0 μM). The gray dotted lines mark one standard deviation (±1σ) from the mean. CV represents the coefficient of variation of standardized intercepts.

To examine the repeatability and reproducibility of CL signals produced by the HI_3_-CL and UV-CL methods, NDMA was selected as a reference compound to evaluate the intraday and interday signal stability in our setup. Consecutive injections of a 1-μM NDMA standard with our HI_3_-CL method showed relatively consistent signal intensities and satisfactory repeatability (i.e., the coefficient of variation (CV) of CL peak areas was 4.5% for seven injections; Figure 2a), which was in contrast to what Breider and von Gunten observed with injections of the same NDMA standard in their setup (i.e., CV = 19.7% for six injections due to signal attenuation).^28^ One key difference between our setup and that assembled by Breider and von Gunten, apart from the specifications of nitric oxide analyzers, was the purge vessel configuration. Our setup utilized a large-volume purge vessel with a bottom injection port, whereas Breider and von Gunten used a commercially available small-volume purge vessel with a top injection port. Indeed, replacing the large-volume vessel with the small-volume unit led to apparent peak broadening (i.e., CV = 7.8% for five injections; Figure 2a), which was likely caused by the dilution effect on the acidic triiodide solution resulting from continuous sample injections.^35^ For our setup, five 100-μL injections of the NDMA standard would dilute the acidic triiodide solution (i.e., ∼4 mL with constant N_2_ purging) inside the small-volume purge vessel by ∼11% and presumably lowered the conversion efficiency of NDMA to NO^•^. Contrary to prior speculation by Hausladen et al.,^26^ spiking sodium nitrite or NDMA into the freshly prepared acidic triiodide solution did not produce characteristic UV–visible absorption bands of nitrosyl iodide in the gas phase above the solution (Figure S1). Concurrent injections of a stoichiometric amount of dimethylamine did not alter signal intensities of NDMA either, suggesting that the suppression of NO^•^ production due to NDMA reformation via the nitrosation of dimethylamine by nitrosyl iodide was minimal in our setup. Given the extremely low pH (i.e., pH < 0.5) of the acidic triiodide solution with a large excess of iodine (i.e., ∼40 mM), dimethylamine should be extensively protonated and therefore unreactive with nitrosyl iodide,^36^ even if any formed. With the use of the large-volume purge vessel, consecutive injections of the 1-μM NDMA standard also showed good signal repeatability with our UV-CL method (i.e., CV = 4.8% for seven injections; Figure 2b), which corroborated the finding of Breider and von Gunten (i.e., CV = 9.1% for nine injections).^28^ Over the study period, repeated interday injections of eight NDMA standards in the range of 0.1–5.0 μM demonstrated comparable signal reproducibility with the HI_3_-CL (i.e., CV = 5.9–11.6% for 80 injections of each standard; Figure S2) and UV-CL methods (i.e., CV = 9.8–15.9% for 73 injections of each standard; Figure S3). Comparing the CV of the slopes and intercepts of NDMA calibration curves further confirmed that the HI_3_-CL method achieved similar signal reproducibility (i.e., CV = 6.1 and 16.6%, respectively, for 80 curves; Figure 2c,e) as the UV-CL method (i.e., CV = 9.9 and 18.4%, respectively, for 73 curves; Figure 2d,f). Taken together, the repeatability and reproducibility of HI_3_-CL and UV-CL measurements supported the suitability of our integrated setup for TONO analysis with both methods. Still, monitoring CL signals with interspersed NDMA standards within and across analytical runs is critical considering the sensitivity of signal responses to fluctuations in gas flow rates and pressure inside the analyzer.^37^

### Method Inclusivity and Sensitivity

**Figure 3:**
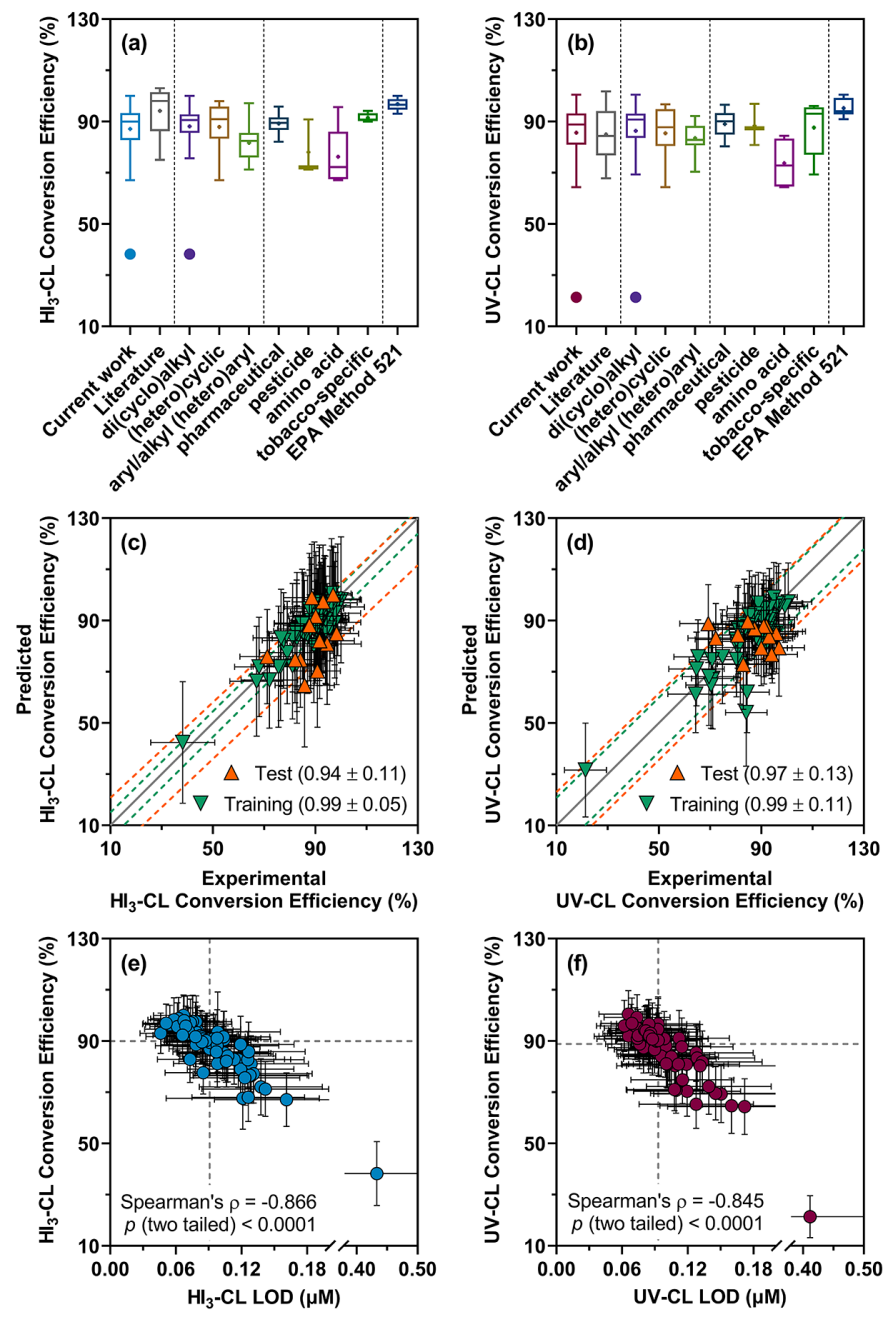
Comparison of the conversion efficiencies and detection limits of *N*-nitrosamines for the HI_3_-CL and UV-CL methods: (a) Box-and-whiskers plots of *N*-nitrosamine conversion efficiencies measured by the HI_3_-CL method (Table S3) along with the literature data (Table S5). Each box extends from the 25th to 75th percentiles. The whiskers extend down to the 25th percentile minus 1.5 times the interquartile range and up to the 75th percentile plus 1.5 times the interquartile range. The centerline and “+” sign mark the median and mean, respectively. The filled circle represents the outlier *N*-nitrosodiisobutylamine. “Current work” (*n* = 66) represents data from this work. “Literature” (*n* = 6) represents data reported by Kulshrestha et al.^15^ “di(cyclo)alkyl” (*n* = 36), “(hetero)cyclic” (*n* = 20), and “alkyl/aryl (hetero)aryl” (*n* = 10) represent data for *N*-nitrosamines featuring different subclasses of substituents on the amine nitrogen. “pharmaceutical” (*n* = 18), “pesticide” (*n* = 3), “amino acid” (*n* = 6), and “tobacco-specific” (*n* = 5) represent data for *N*-nitrosamines formed from different types of precursors. “EPA 521 Method” (*n* = 7) represents data for *N*-nitrosamines included in EPA Method 521. (b) Box-and-whiskers plots of *N*-nitrosamine conversion efficiencies measured by the UV-CL method (Table S4) along with the literature data (Table S5). Each box extends from the 25th to 75th percentiles. The whiskers extend down to the 25th percentile minus 1.5 times the interquartile range and up to the 75th percentile plus 1.5 times the interquartile range. The centerline and “+” sign mark the median and mean, respectively. The filled circle represents the outlier *N*-nitrosodiisobutylamine. “Current work” (*n* = 66) represents data from this work. “Literature” represents data (*n* = 10) reported by Breider and von Gunten.^28^ “di(cyclo)alkyl” (*n* = 36), “(hetero)cyclic” (*n* = 20), and “alkyl/aryl (hetero)aryl” (*n* = 10) represent data for *N*-nitrosamines featuring different subclasses of substituents on the amine nitrogen. “pharmaceutical” (*n* = 18), “pesticide” (*n* = 3), “amino acid” (*n* = 6), and “tobacco-specific” (*n* = 5) represent data for *N*-nitrosamines formed from different types of precursors. “EPA 521 Method” (*n* = 7) represents data for *N*-nitrosamines included in EPA Method 521. (c) Cross plot of the conversion efficiencies of *N*-nitrosamines with reference to NDMA measured by the HI_3_-CL method and those predicted by the regression model containing nine molecular descriptors (Table S8). Error bars represent the 95% confidence intervals of experimental *N*-nitrosamine conversion efficiencies or the standard errors of predicted conversion efficiencies; where absent, bars fall within symbols. The gray solid line represents the line of identity. The green dashed lines bracket one standard deviation from the mean ratio (i.e., 0.99 ± 0.05) of predicted to experimental conversion efficiencies for the training data set (*R*_adj_^2^ = 0.84). The orange dashed lines bracket one standard deviation from the mean ratio (i.e., 0.94 ± 0.11) of predicted to experimental conversion efficiencies for the test data set (*Q*^2^ = 0.85). (d) Cross plot of the conversion efficiencies of *N*-nitrosamines with reference to NDMA measured by the UV-CL method and those predicted by the regression model containing six molecular descriptors (Table S9). Error bars represent the 95% confidence intervals of experimental *N*-nitrosamine conversion efficiencies or the standard errors of predicted conversion efficiencies; where absent, bars fall within symbols. The gray solid line represents the line of identity. The green dashed lines bracket one standard deviation from the mean ratio (i.e., 0.99 ± 0.11) of predicted to experimental conversion efficiencies for the training data set (*R*_adj_^2^ = 0.81). The orange dashed lines bracket one standard deviation from the mean ratio (i.e., 0.97 ± 0.13) of predicted to experimental conversion efficiencies for the test data set (*Q*^2^ = 0.86). (e) Cross plot of the conversion efficiencies of *N*-nitrosamines with reference to NDMA and the LODs measured by the HI_3_-CL method (Table S3). Error bars represent the 95% confidence intervals of *N*-nitrosamine conversion efficiencies and LODs; where absent, bars fall within symbols. The gray dashed lines mark the median conversion efficiency and the median LOD of *N*-nitrosamines, respectively. (f) Cross plot of the conversion efficiencies of *N*-nitrosamines with reference to NDMA and the LODs measured by the UV-CL method (Table S4). Error bars represent the 95% confidence intervals of *N*-nitrosamine conversion efficiencies and LODs; where absent, bars fall within symbols. The gray dashed lines mark the median conversion efficiency and the median LOD of *N*-nitrosamines, respectively.

One key assumption underlying TONO quantification was that the conversion efficiencies of specific *N*-nitrosamines to NO^•^ were comparable to that of NDMA via either chemical or photolytic denitrosation,^15,28^ but it remains unclear to what extent such an assumption applies to *N*-nitrosamines less frequently targeted NOCs such as *N*-nitroso derivatives of pharmaceuticals and pesticides or their building blocks, *N*-nitrosoamino acids, and tobacco-specific *N*-nitrosamines. To assess the inclusivity of our HI_3_-CL and UV-CL methods, the conversion efficiencies of 66 *N*-nitrosamines featuring structurally diverse substituents on the amine nitrogen (Table S2) were measured with reference to NDMA. With the HI_3_-CL method, the conversion efficiencies of these *N*-nitrosamines spanned a range of 38 ± 12% to 99 ± 8% with a median value of 90% (Figure 3a). On average, the conversion efficiencies of *N*-nitrosamines featuring di(cyclo)alkyl (i.e., 88 ± 13%; *n* = 36), (hetero)cyclic (i.e., 88 ± 13%; *n* = 20), and alkyl/aryl (hetero)aryl (i.e., 82 ± 11%; *n* = 10) substituents on the amine nitrogen were not statistically different (Tukey’s multiple comparisons test *p* = 0.2671–0.9820) but significantly lower than that of NDMA (Mann–Whitney *U* test *p* = 0.0009). Furthermore, the conversion efficiencies of *N*-nitroso derivatives of pharmaceuticals (i.e., 89 ± 8%; *n* = 18) and pesticides (i.e., 78 ± 13%; *n* = 3) and *N*-nitrosoamino acids (i.e., 76 ± 13%; *n* = 6) were lower than those of *N*-nitrosamines (i.e., 97 ± 7%; *n* = 7) targeted by EPA Method 521^38^ (Tukey’s multiple comparisons test *p* < 0.0001–0.0214), but the conversion efficiencies of tobacco-specific *N*-nitrosamines (i.e., 92 ± 7%; *n* = 5) did not differ from those of EPA Method 521 *N*-nitrosamines (Tukey’s multiple comparisons test *p* = 0.3567). With the UV-CL method, the conversion efficiencies of *N*-nitrosamines ranged from 21 ± 8% to 99 ± 9% with a median value of 89% (Figure 3b) and exhibited a positive correlation with those determined by the HI_3_-CL method (Spearman’s ρ = 0.507; *p* < 0.0001; Figure S7). On average, the conversion efficiencies of *N*-nitrosamines featuring three different subclasses of amine nitrogen substituents (i.e., 86 ± 15% for di(cyclo)alkyl, 85 ± 13% for (hetero)cyclic, and 84 ± 10% for alkyl/aryl (hetero)aryl, respectively) were not statistically different from those derived from the HI_3_-CL method (Tukey’s multiple comparisons test *p* = 0.9168–0.9993). The conversion efficiencies of *N*-nitroso derivatives of pharmaceuticals (i.e., 89 ± 10%) and pesticides (i.e., 88 ± 10%) as well as tobacco-specific *N*-nitrosamines (i.e., 88 ± 13%) were higher than those of *N*-nitrosoamino acids (i.e., 74 ± 12%; Tukey’s multiple comparisons test *p* = 0.0002–0.0171) but did not differ from those of EPA Method 521 *N*-nitrosamines (i.e., 95 ± 7%; Tukey’s multiple comparisons test *p* = 0.1643–0.4320). In addition, the conversion efficiencies of the equal-molar mixture of *N*-nitrosamines measured by the HI_3_-CL and UV-CL methods (i.e., 90 ± 9 and 91 ± 11%, respectively) converged reasonably well with the values estimated by averaging the conversion efficiencies of 66 *N*-nitrosamines (i.e., 87 ± 12 and 86 ± 14%, respectively). Generally, the conversion efficiencies of commonly studied *N*-nitrosamines determined herein were similar to those reported by previous studies (Table S5). For example, the mean conversion efficiency of EPA Method 521 *N*-nitrosamines measured by our HI_3_-CL method (i.e., 97 ± 7%) agreed with that reported by Kulshrestha et al. (i.e., 103 ± 3% as a mixture).^15^ Moreover, the conversion efficiencies of EPA Method 521 *N*-nitrosamines, *N*-nitrosomorpholine, *N*-nitrosodiphenylamine, and *N*-nitrosodiethanolamine (i.e., 91 ± 11 to 99 ± 9%) measured by our UV-CL method fell within the range (i.e., 68 ± 5 to 102 ± 6%) reported by Breider and von Gunten.^28^ Taking the average of all conversion efficiencies measured in this work and these two prior studies yielded a global conversion efficiency of 86 ± 13% relative to NDMA; however, applying this value as a single correction factor for TONO may not capture the variability in NO^•^ production resulting from chemical or photolytic denitrosation of *N*-nitrosamines.

Given that chemical and photolytic denitrosation reactions proceed through the heterolytic and homolytic cleavage of the N–NO bond in *N*-nitrosamines, respectively (Figures S8 and S9), correlation analyses were performed to evaluate whether variations in the heterolytic and homolytic N–NO bond dissociation energies (i.e., BDE_N–NO_het_ and BDE_N–NO_homo_) serve to rationalize differences in the conversion efficiencies of *N*-nitrosamines. Two graph neural network models, Fast, Accurate Bond dissociation Enthalpy Tool (ALFABET)^39^ and Bond Dissociation Network (BonDNet),^40^ were applied to calculate the BDE_N–NO_homo_ of *N*-nitrosamines (Table S6). ALFABET was trained on 290,664 unique homolytic BDEs for 42,557 neutral molecules,^39^ whereas BonDNet was trained on 64,312 unique homolytic and heterolytic BDEs for neutral and charged molecules and their fragments.^40^ For the PubChem BDE data set,^41^ ALFABET and BonDNet achieved mean absolute errors of 0.58 and 0.47 kcal/mol, respectively, for predicted BDEs,^39,40^ which fell below the chemical accuracy threshold of 1 kcal/mol.^42^ Predicting BDE_N–NO_het_ of *N*-nitrosamines was not feasible with either model due to the lack of experimental heterolytic BDE data, but earlier investigations demonstrated that titration calorimetry-measured BDE_N–NO_het_ of *N*-nitrosodiphenylamine derivatives^43^ and *N*-nitrosoureas^44^ showed strong linear correlations with the p*K*_a_’s of their precursors. Nevertheless, the conversion efficiencies of *N*-nitrosamines measured by the HI_3_-CL method only showed a weak correlation (Figure S10) with the p*K*_a_’s of their corresponding secondary amine precursors (Table S7), whereas the conversion efficiencies of *N*-nitrosamines measured by the UV-CL method were not correlated with BDE_N–NO_homo_ (Figure S10). Given the lack of predictive power of N–NO bond energetic data, a regression analysis workflow utilizing elastic net regression, bidirectional stepwise regression, and best subsets regression was implemented to explore whether molecular descriptors of *N*-nitrosamines might serve as predictors for their conversion efficiencies (Figure 3c,d). Molecular descriptors prioritized as statistically significant predictor variables with this workflow highlighted that the chemical denitrosation of *N*-nitrosamines upon HI_3_ treatment was influenced by a combination of their electronic, topological, and steric properties, whereas the photolytic denitrosation of *N*-nitrosamines upon UV irradiation at 254 nm was largely governed by their electronic properties (Section S5). Collectively, these analyses pointed to the necessity of experimentally determining the compound-specific conversion efficiencies of *N*-nitrosamines to constrain the inclusivity of HI_3_-CL and UV-CL methods for TONO measurements.

To evaluate the sensitivity of our HI_3_-CL and UV-CL methods, the LODs and LOQs of *N*-nitrosamines were calculated based on an injection volume of 100 μL. For both methods, the LODs of *N*-nitrosamines showed strong negative correlations with their conversion efficiencies (Figure 3e,f), confirming the correspondence of method sensitivity to the susceptibility of *N*-nitrosamines to chemical or photolytic denitrosation. Except for *N*-nitrosodiisobutylamine (which featured the lowest conversion efficiency among all tested *N*-nitrosamines), the LODs of *N*-nitrosamines measured by the HI_3_-CL method ranged from 0.05 to 0.16 μM with a median value of 0.09 μM, while the LODs for the UV-CL method were not statistically different (paired *t*-test *p* = 0.1829), ranging from 0.06 to 0.17 μM with a median value of 0.09 μM. For comparison, the LODs of *N*-nitrosamines measured herein bracketed the values reported by Kulshrestha et al.^15^ (i.e., 0.11 μM with 100-μL injections of NDMA) for their HI_3_-CL method as well as those reported by Breider and von Gunten for their UV-CL method (i.e., 0.07–0.13 μM with 200-μL injections of 10 *N*-nitrosamines).^28^ On average, the LODs of the equal-molar mixture of *N*-nitrosamines measured by the HI_3_-CL and UV-CL methods were 0.09 ± 0.05 and 0.10 ± 0.06 μM, respectively. Considering a median SPE recovery of 66% for *N*-nitrosamines (see below), the median LODs in wastewater samples would be 41 and 42 ng/L as NDMA for the HI_3_-CL and UV-CL methods, respectively, assuming a 250-fold sample concentration factor. Overall, the inclusivity of HI_3_-CL and UV-CL methods was comparable despite the heterogeneity in the conversion efficiencies of *N*-nitrosamines; however, the sensitivity of either method for TONO measurements would depend not only on denitrosation conditions and analyzer specifications but also on operational parameters such as injection volumes, gas flow rates, and pressure.

### Application to Wastewater Analysis

To date, studies investigating the occurrence and formation of TONO in water treatment and reuse scenarios have primarily applied SPE protocols modified from EPA Method 521^38^ for sample preconcentration. Typically, samples (quenched by l-ascorbic acid and sulfamic acid) were passed through activated carbon-based SPE cartridges, which were eluted with either methanol alone^32^ or dichloromethane plus methanol^22,23^ to improve the recoveries of NDMA over continuous liquid–liquid extraction with ethyl acetate.^21^ Our dual-cartridge SPE protocol eliminated the use of methanol as the cartridge eluant to minimize the co-extraction of wastewater matrix components that might contribute to signal attenuation and baseline drift in CL detection (Figure S11). With dichloromethane as the only solvent used for cartridge elution,^31^ the SPE recoveries of volatile *N*-nitrosamines improved probably due to the reduction in time required for rotary evaporation and N_2_ blowdown. For example, the recoveries of NDMA (i.e., 87 ± 6%) and *N*-nitrosomorpholine (i.e., 94 ± 4%) measured in drinking water by our protocol exceeded those obtained using the modified EPA Method 521 (i.e., 45–56% for NDMA and 71% for *N*-nitrosomorpholine, respectively; Table S15).^22,23,32^ On the other hand, the recoveries of some nonvolatile *N*-nitrosamines declined after excluding methanol for cartridge elution from our protocol. For example, the SPE recovery of *N*-nitrosodiethanolamine (i.e., 48 ± 4%) in drinking water was lower than that measured with the combined use of dichloromethane and methanol as the cartridge eluants (i.e., 76%).^22^ On average, the SPE recoveries of *N*-nitrosamines in wastewater influent (i.e., 59 ± 23%), wastewater effluent (i.e., 62 ± 23%), and drinking water samples (i.e., 66 ± 25%) were not statistically different (Tukey’s multiple comparisons test *p* = 0.3621–0.7995). Our protocol, although did not yield high recoveries for all analytes, was able to extract structurally diverse *N*-nitrosamines covering a range of Log*D* values (i.e., from −1.42 for *N*-nitrosodiethanolamine to 4.32 for *N*-nitrosofluoxetine) from wastewater matrices with moderate recoveries. For comparison, the mean SPE recoveries of EPA Method 521 *N*-nitrosamines measured in wastewater by our protocol (i.e., 78 ± 10%) overlapped with those reported by Krauss and Hollender (i.e., 68 ± 39% with the joint application of Oasis HLB and activated carbon cartridges; Table S15)^31^ and Breider and von Gunten (i.e., 46–61% with the use of Oasis HLB cartridges alone).^28^ Taking into account the balance between matrix effects and recoveries of both volatile and nonvolatile *N*-nitrosamines, the dual-cartridge SPE protocol was applied without additional optimization.

**Figure 4:**
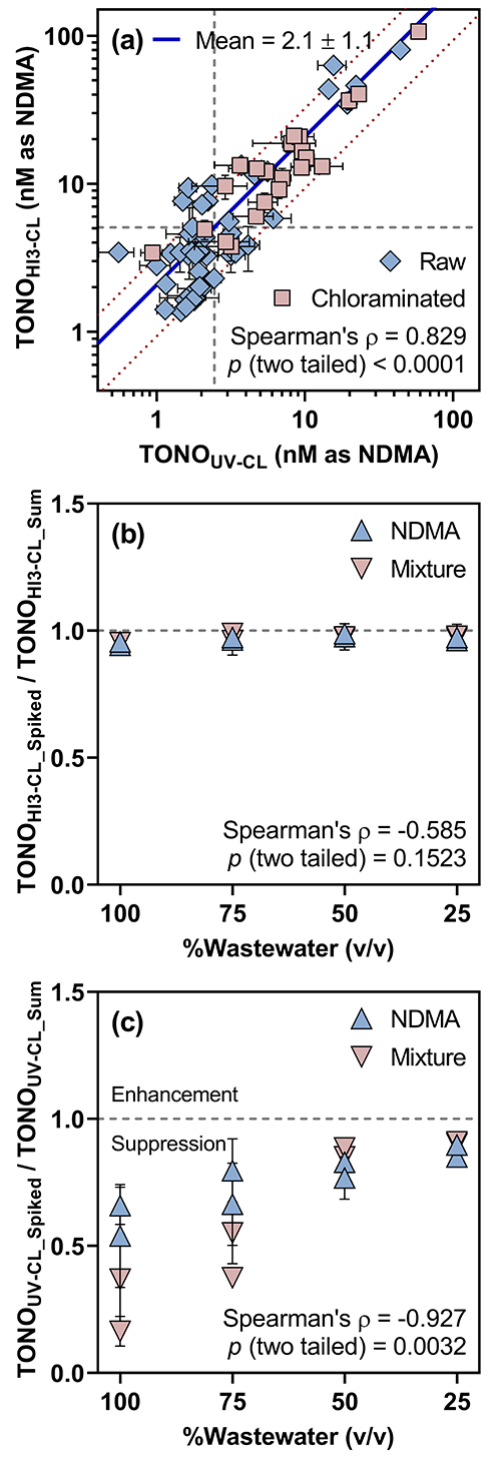
Comparison of total *N*-nitrosamine (TONO) analysis for wastewater samples by the HI_3_-CL and UV-CL methods: (a) Cross plot of TONO measured in raw and chloraminated wastewater samples by the HI_3_-CL method as NDMA equivalents (TONO_HI_3_ – CL_) and TONO measured by the UV-CL method (TONO_UV – CL_) as NDMA equivalents. Blue rhombuses represent TONO measured for raw wastewater samples (*n* = 48). Red squares represent TONO measured for chloraminated wastewater samples (*n* = 23). Error bars represent the standard deviations of TONO; where absent, bars fall within symbols. The gray dashed lines mark the median TONO_HI_3_ – CL_ and TONO_UV – CL_. The blue solid line represents the mean ratio of TONO_HI_3_ – CL_ to TONO_UV – CL_ (*n* = 71). The red dotted lines represent one standard deviation from the mean ratio of TONO_HI_3_ – CL_ to TONO_UV – CL_. (b) Scatter plot of the ratio of TONO_HI_3_ – CL_ for NDMA-spiked or mixture-spiked wastewater samples (TONO_HI_3_ – CL_Spiked_) to the sum of TONO_HI_3_ – CL_ for corresponding non-spiked samples and TONO_HI_3_ – CL_ for NDMA-spiked or mixture-spiked deionized water (TONO_HI_3_ – CL_Sum_) versus the volumetric percentage of wastewater (i.e., 100, 75, 50, and 25%) diluted into deionized water for analysis (Figure S12). Blue upward-pointing triangles represent the NDMA spike. Red downward-pointing triangles represent the spike of an equal-molar mixture of *N*-nitrosamines. Error bars represent the standard deviations of ratios calculated by analyzing three influent and effluent samples, respectively; where absent, bars fall within symbols. The gray dashed line marks the ratio of 1.0. Note that the *x*-axis is reversed. (c) Scatter plot of the ratio of TONO_UV – CL_ for NDMA-spiked or mixture-spiked wastewater samples (TONO_UV – CL_Spiked_) to the sum of TONO_UV – CL_ for corresponding non-spiked samples and TONO_UV – CL_ for NDMA-spiked or mixture-spiked deionized water (TONO_UV – CL_Sum_) versus the volumetric percentage of wastewater (i.e., 100, 75, 50, and 25%) diluted into deionized water for analysis (Figure S13). Blue upward-pointing triangles represent the NDMA spike. Red downward-pointing triangles represent the spike of an equal-molar mixture of *N*-nitrosamines. Error bars represent the standard deviations of ratios calculated by analyzing three influent and effluent samples, respectively; where absent, bars fall within symbols. The gray dashed line marks the ratio of 1.0. Note that the *x*-axis is reversed.

To characterize the performance of HI_3_-CL and UV-CL methods for TONO quantification in wastewater, a panel of 71 raw (i.e., non-chloraminated) and chloraminated wastewater samples were preconcentrated by the SPE protocol described above. For these samples, TONO measured by the HI_3_-CL method (i.e., TONO_HI_3_ – CL_) ranged from 1.2 to 107 nM as NDMA equivalents, whereas TONO measured by the UV-CL method (i.e., TONO_UV – CL_) fell within a narrower range of 0.65 to 62 nM as NDMA equivalents. On average, the ratio of TONO_HI_3_ – CL_ to TONO_UV – CL_ varied from 0.91 ± 0.14 to 6.2 ± 1.7 with a mean value of 2.1 ± 1.1 for raw and chloraminated wastewater samples. Qualitatively, TONO_HI_3_ – CL_ exhibited a strong positive correlation with TONO_UV – CL_ (Spearman’s ρ = 0.829; *p* < 0.0001; Figure 4a), but TONO measurements by the HI_3_-CL and UV-CL methods were not quantitatively equivalent, possibly due to CL signal suppression or enhancement associated with matrix co-extractives.

To test this hypothesis, six WWTP influent and effluent samples (i.e., either undiluted or diluted serially with deionized water) were spiked with either 5 nM of NDMA or the equal-molar mixture of *N*-nitrosamines and analyzed for TONO (Figures S12 and S13). Five nM was chosen as the spike level to represent the median TONO_HI_3_ – CL_ (i.e., 5.1 nM) measured for the 71 wastewater samples. On average, the ratio of TONO_HI_3_ – CL_ for NDMA-spiked or mixture-spiked wastewater samples to the sum of TONO_HI_3_ – CL_ for corresponding non-spiked samples and spiked deionized water (Figure 4b) approached 1.0 (i.e., 0.97 ± 0.03) irrespective of the extent of dilution. In contrast, the ratio of TONO_UV – CL_ for the identical NDMA-spiked or mixture-spiked samples to the sum of TONO_UV – CL_ for non-spiked samples and spiked deionized water was lowest (i.e., 0.43 ± 0.17) for undiluted samples (i.e., 100% wastewater) and progressively increased (i.e., from 0.57 ± 0.13 to 0.89 ± 0.04) with higher degrees of dilution (Figure 4c; the volumetric percentage of wastewater was reduced to 75, 50, and 25% in the serial dilutions). Given that the spike recoveries of NDMA and the *N*-nitrosamine mixture exhibited a strong negative correlation with TONO_UV – CL_ (Spearman’s ρ = −0.927; *p* = 0.0032) but no statistically significant correlation with TONO_HI_3_ – CL_across samples, it was plausible that CL signals attributable to TONO_UV – CL_ were disproportionately suppressed by UV-absorbing and/or NO^•^-scavenging matrix components of wastewater origin. Such signal reduction in TONO_UV – CL_ was not observed by Breider and von Gunten who tested the spike recovery of a 15-nM nine-component *N*-nitrosamine mixture in a wastewater effluent sample,^28^ presumably because of compositional differences in TONO and matrix co-extractives among samples analyzed in this and their work. For undiluted wastewater, TONO_HI_3_ – CL_ for NDMA-spiked samples matched those for mixture-spiked samples with a mean ratio of 1.03 ± 0.05 as would have been expected from the conversion efficiency of the *N*-nitrosamine mixture (i.e., 90 ± 9%). Conversely, TONO_UV – CL_ for NDMA-spiked samples far exceeded those of mixture-spiked samples with a mean ratio of 3.09 ± 1.35 despite the comparable conversion efficiency measured for the *N*-nitrosamine mixture (i.e., 91 ± 11%), suggesting the preferential suppression of TONO_UV – CL_ by matrix co-extractives for certain *N*-nitrosamines over NDMA. Combined with the concentration patterns observed for raw and chloraminated samples, our spike recovery tests supported that TONO measurements by the HI_3_-CL method were less susceptible to uncertainties associated with wastewater-derived matrix interferences although such effects were likely of less concern when applying either the HI_3_-CL or UV-CL method to analyze TONO in less complex aqueous matrices.

### Environmental Implications

Overall, this study addressed several practical considerations when implementing the HI_3_-CL and UV-CL methods for TONO analysis in wastewater, which typically serve as a predominant source of TONO and their precursors. Starting with the baseline characterization of CL signal stability via intraday and interday injections of NDMA standards, we showed that both methods were able to achieve repeatable and reproducible signals in an integrated experimental setup configured with a large-volume purge vessel for chemical denitrosation and a UV microphotochemical reactor for photolytic denitrosation, respectively. Regardless of the denitrosation conditions applied, *N*-nitrosamines featuring structurally diverse substituents on the amine nitrogen yielded a range of conversion efficiencies to NO^•^ relative to NDMA; however, the variability in the conversion efficiencies of *N*-nitrosamines likely originated from differences in their electronic, topological, and/or steric molecular properties and could not be readily rationalized by variations in their heterolytic and homolytic N–NO bond energetic data. Correcting TONO by applying a generic conversion efficiency (e.g., 86 ± 13% based on data from this and prior work) should therefore incorporate an uncertainty analysis to account for the heterogeneity in NO^•^ production via denitrosation reactions. Through the comparative analysis of TONO in an array of raw and chloraminated wastewater samples and spike recovery tests with NDMA and an equal-molar mixture of *N*-nitrosamines, we demonstrated that TONO measurements by the HI_3_-CL method were less sensitive to interferences from wastewater matrix co-extractives when coupled with a dual-cartridge SPE protocol for sample preconcentration. Going forward, developing a protocol that maximizes the recoveries of *N*-nitrosamine mixtures from aqueous samples would be essential because previous and current TONO measurements at best captured varying fractions of *N*-nitrosamines that were amenable to preconcentration by specific SPE methods and should technically be defined as “extractable” TONO. Complicating matters further, the SPE recoveries and conversion efficiencies of *N*-nitrosamines vary with experimental conditions and instrumental configurations, so some level of data standardization would be required to compare TONO reported by different research groups. Nonetheless, at the very least, corrections for discrepancies in SPE recoveries and conversion efficiencies among *N*-nitrosamines should be made before specifying the fractional contributions of known or newly identified *N*-nitrosamines to TONO in any given sample. Our findings in this work invite further studies to establish robust class-based methods for characterizing *N*-nitrosamines of environmental and health significance.

## Supporting Information Available

CL signal stability of NDMA standards; HI_3_-CL and UV-CL method performance metrics; chemical and photolytic denitrosation mechanisms of *N*-nitrosamines; molecular descriptors selected as predictors for the conversion efficiencies of *N*-nitrosamines; SPE-LC-HRMS method performance metrics and validation results; effects of dilution on the spike recoveries of NDMA and the *N*-nitrosamine mixture in wastewater; and example LC-HRMS chromatograms of *N*-nitrosamines (PDF)
