# Supplementary material for: Comparative Evaluation of Chemical and Photolytic Denitrosation Methods for Chemiluminescence Detection of Total N-Nitrosamines in Wastewater Samples
Source: Environ Sci Technol. 2023 May 4;57(19):7526–36. doi: 10.1021/acs.est.2c09769 (PMC10193580; doi:10.1021/acs.est.2c09769)
Supplement: Supplementary file 1 — es2c09769_si_001.pdf [file es2c09769_si_001.pdf]

Supporting Information for

Comparative Evaluation of Chemical and Photolytic Denitrosation Methods for Chemiluminescence Detection of Total *N*-Nitrosamines in Wastewater Samples

Changcheng Pu[1], Teng Zeng[,1]*

[1]Department of Civil and Environmental Engineering, Syracuse University, 151 Link Hall, Syracuse, New York 13244, United States

*Corresponding Author: Teng Zeng: Email: tezeng@syr.edu; Phone: +1-315-443-1099

(Total 101 pages, 4 texts, 15 tables, 14 figures)

Table of Contents

1. Chemicals, reagents, and supplies S3

2. Chemiluminescence signal stability of *N*-nitrosodimethylamine (NDMA) standards S14

3. HI_3-CL and UV-CL method performance S18

4. Chemical and photolytic denitrosation mechanisms of *N*-nitrosamines S41

5. Molecular descriptors as predictors for the conversion efficiencies of *N*-nitrosamines S47

6. SPE-LC-HRMS method performance and validation S51

7. Effects of wastewater dilution on the spike recoveries of NDMA and the *N*-nitrosamine mixture S66

8. Example LC-HRMS chromatograms of *N*-nitrosamines S68

References S100

1. Chemicals, reagents, and supplies

Chemicals and reagents were used as received without further purification. Methanol (HPLC and LC-MS grade), water (HPLC and LC-MS grade), methylene chloride (Optima grade), pentane (HPLC grade), ethyl acetate (HPLC grade), acetic acid (glacial HPLC grade), formic acid solution (FA; ≥99.0%; LC-MS grade), poly(ethylene glycol) (average M.W. 6000), sodium hypochlorite solution (NaOCl; 5.65-6% laboratory grade), sodium sulfate (anhydrous, ≥99.0%), and sodium hydroxide (>99%) were purchased from Fisher Scientific. Ammonium chloride (NH_4Cl; 99.5%), L-ascorbic acid (99%), dimethylamine (DMA; 40 wt.% solution in water), morpholine (99+%), potassium iodide (KI; 99+%), iodine (I_2; 99.5%), sodium nitrite ($NaNO_2$; ≥99.0%), sodium dihydrogen phosphate monohydrate (99+%), and sodium phosphate dibasic heptahydrate (99+%) were purchased from ACROS Organics. Sulfamic acid (99.3%) and *N,N*-diethyl-*p*-phenylenediamine sulfate salt (≥98.0%) were purchased from Sigma-Aldrich.

N-Nitrosamine reference standards and isotope-labeled internal standards (ILIS) were purchased from or custom synthesized by Enamine, Sigma-Aldrich, Toronto Research Chemicals, and C/D/N Isotopes as high-purity substances (**Table S1**). Stock solutions of *N*-nitrosamines were prepared by dissolving or diluting a gravimetrically weighted amount of solid or liquid reference standards into LC-MS grade water. Calibration standards were prepared by diluting predetermined volumes of stock solutions into deionized water.

Preformed monochloramine (NH_2Cl) working solutions were prepared by dissolving NH_4Cl solids in deionized water (resistivity 18.2 MΩ•cm; produced by a Thermo Scientific Barnstead MicroPure UV/UF water purification system) adjusted to pH 9, followed by dropwise addition of standardized NaOCl solution to obtain a Cl:N molar ratio of 1:1.2. The concentration of NH_2Cl was determined using a Thermo Scientific Evolution 201 UV-visible spectrophotometer by deconvoluting solution absorbance at 245 nm ($\varepsilon_{245nm}(NH_2Cl) = 445\ M^{-1}\ cm^{-1}$) and 295 nm ($\varepsilon_{295nm}(NHCl_2) = 14\ M^{-1}\ cm^{-1}$).[1]

Two SPE cartridges, Strata-X (33 µm polymeric reversed phase, 200 mg/6 mL) and Enviro-Clean 521 (coconut shell activated carbon, 2000 mg/15 mL), were purchased from Phenomenex and United Chemical Technologies,

respectively. Quartz cuvettes (1-I-10 and 9-Q-10-GL14-S) and septum caps (GL14-S) for spectrophotometric measurements were purchased from Starna Cells.

High-density polyethylene (HDPE) sampling bottles, large-volume sample transfer tubes, and microsyringes were rinsed 5 times with HPLC grade methanol, followed by 5 times with deionized water, and dried overnight. Volumetric glassware and purge vessels were rinsed with HPLC grade methanol and deionized water and dried at 70 °C in a Fisherbrand Isotemp general purpose heating and drying oven. Non-volumetric glassware was rinsed and combusted at 450 °C in a Thermo Scientific Lindberg/Blue M Moldatherm box furnace for 5 h.

**Table S1.** List of *N*-nitrosamine reference standards and isotope-labeled internal standards							
Compound	Acronym	Supplier	Purity	CAS	Molecular Formula	Molecular Weight	Category
N-Nitrosodimethylamine	NDMA	Sigma-Aldrich	99.9%	62-75-9	$C_2H_6N_2O$	74.08	dialkyl
N-Nitrosomethylethylamine	NMEA	TRC	97.0%	10595-95-6	$C_3H_8N_2O$	88.11	dialkyl
N-Nitrosodiethylamine	NDEA	TRC	98.0%	55-18-5	$C_4H_{10}N_2O$	102.14	dialkyl
N-Nitrosodipropylamine	NDPA	TRC	98.0%	621-64-7	$C_6H_{14}N_2O$	130.19	dialkyl
N-Nitrosodibutylamine	NDBA	TRC	98.0%	924-16-3	$C_8H_{18}N_2O$	158.25	dialkyl
N-Nitrosopyrrolidine	NPYR	TRC	98.0%	930-55-2	$C_4H_8N_2O$	100.12	cyclic
N-Nitrosopiperidine	NPIP	TRC	98.0%	100-75-4	$C_5H_{10}N_2O$	114.15	cyclic
N-Nitrosomorpholine	NMOR	TRC	98.0%	59-89-2	$C_4H_8N_2O_2$	116.12	heterocyclic
N-Nitrosodiphenylamine	NDPhA	TRC	98.0%	86-30-6	$C_{12}H_{10}N_2O$	198.23	diaryl
N-Nitrosodiethanolamine	NDELA	TRC	98.0%	1116-54-7	$C_4H_{10}N_2O_3$	134.14	dialkyl
N-Nitrososarcosine	NSAR	TRC	98.0%	13256-22-9	$C_3H_6N_2O_3$	118.09	dialkyl
N-Nitrosoproline	NPRO	TRC	98.0%	7519-36-0	$C_5H_8N_2O_3$	144.13	cyclic
N-Nitrosopipecolic Acid	NPIC	Enamine	95.0%	4515-18-8	$C_6H_{10}N_2O_3$	158.16	cyclic
N-Nitrosohydroxyproline	NHPRO	TRC	95.0%	30310-80-6	$C_5H_8N_2O_4$	160.13	cyclic
N-Nitrosothiazolidine-4-Carboxylic Acid	NTCA	TRC	98.0%	88381-44-6	$C_4H_6N_2O_3S$	162.16	heterocyclic
N-Nitroso-2-Methylthiazolidine 4-Carboxylic Acid	NMTCA	TRC	95.0%	103659-08-1	$C_5H_8N_2O_3S$	176.19	heterocyclic
N-Nitrosonornicotine	NNN	TRC	100.0%	80508-23-2	$C_9H_{11}N_3O$	177.21	cyclic
N-Nitrosoanatabine	NAT	TRC	99.8%	887407-16-1	$C_{10}H_{11}N_3O$	189.22	cyclic
N-Nitrosoanabasine	NAB	TRC	99.8%	37620-20-5	$C_{10}H_{13}N_3O$	191.23	cyclic
4-(Methylnitrosamino)-1-(3-Pyridyl)-1-Butanone	NNK	TRC	100.0%	64091-91-4	$C_{10}H_{13}N_3O_2$	207.23	dialkyl
4-(Methylnitrosamino)-1-(3-Pyridyl)-1-Butanol	NNAL	TRC	95.0%	76014-81-8	$C_{10}H_{15}N_3O_2$	209.25	dialkyl
N-Nitrosomethylisopropylamine	NMIPA	Enamine	95.0%	30533-08-5	$C_4H_{10}N_2O$	102.14	dialkyl
N-Nitrosoethylpropylamine	NEPA	Enamine	95.0%	25413-61-0	$C_5H_{12}N_2O$	116.16	dialkyl
N-Nitrosomethylbutylamine	NMBA	Enamine	95.0%	7068-83-9	$C_5H_{12}N_2O$	116.16	dialkyl
N-Nitrosomethylisobutylamine	NMIBA	Enamine	95.0%	34419-76-6	$C_5H_{12}N_2O$	116.16	dialkyl
N-Nitroso-*tert*-Butylmethylamine	NTBMA	Enamine	95.0%	2504-18-9	$C_5H_{12}N_2O$	116.16	dialkyl
N-Nitrosomethylamylamine	NMAA	TRC	97.0%	13256-07-0	$C_6H_{14}N_2O$	130.19	dialkyl
N-Nitrosodiisopropylamine	NDIPA	TRC	97.0%	601-77-4	$C_6H_{14}N_2O$	130.19	dialkyl
N-Nitroso-*tert*-Butylethylamine	NTBEA	Enamine	95.0%	3398-69-4	$C_6H_{14}N_2O$	130.19	dialkyl
N-Nitrosomethyl-*N,N*-Dimethylethylamine	NMDEA	Enamine	95.0%	23834-30-2	$C_5H_{13}N_3O$	131.18	dialkyl
N-Nitrosodiisobutylamine	NDIBA	TRC	97.0%	997-95-5	$C_8H_{18}N_2O$	158.25	dialkyl
N-Nitrosodiamylamine	NDAA	TRC	97.0%	13256-06-9	$C_{10}H_{22}N_2O$	186.30	dialkyl
N-Nitrosoethylbenzylamine	NEBzA	Enamine	95.0%	20689-96-7	$C_9H_{12}N_2O$	164.21	dialkyl
N-Nitrosodibenzylamine	NDBzA	TRC	98.0%	5336-53-8	$C_{14}H_{14}N_2O$	226.28	dialkyl
1-Nitroso-4-Methylpiperidine	NMPIP	Enamine	95.0%	15104-03-7	$C_6H_{12}N_2O$	128.18	cyclic
N-Nitroso-2-Pyrrolidinmethanol	NPYRM	Enamine	95.0%	68292-94-4	$C_5H_{10}N_2O_2$	130.15	cyclic
N-Nitrosopiperazine	NPPZ	TRC	98.0%	5632-47-3	$C_4H_9N_3O$	115.14	heterocyclic
1-Nitroso-4-Phenylpiperazine	NPhPPZ	TRC	98.0%	14340-33-1	$C_{10}H_{13}N_3O$	191.23	heterocyclic
N-Nitrosoindoline	NIND	Enamine	95.0%	7633-57-0	$C_8H_8N_2O$	148.17	cyclic alkylaryl

Table S1. List of *N*-nitrosamine reference standards and isotope-labeled internal standards (continued)							
Compound	Acronym	Supplier	Purity	CAS	Molecular Formula	Molecular Weight	Category
1-Nitroso-1,2,3,4-Tetrahydroquinoline	NTHQ	Enamine	95.0%	5825-44-5	$C_9H_{10}N_2O$	162.19	cyclic alkylaryl
N-Nitrosomethylcyclohexylamine	NMChA	Enamine	95.0%	5432-28-0	$C_7H_{14}N_2O$	142.20	alkylcycloalkyl
N-Nitrosodicyclohexylamine	NDChA	TRC	98.0%	947-92-2	$C_{12}H_{22}N_2O$	210.32	dicycloalkyl
N-Nitrosomethylphenylamine	NMPhA	TRC	95.0%	614-00-6	$C_7H_8N_2O$	136.15	alkylaryl
N-Nitrosoethylphenylamine	NEPhA	Enamine	95.0%	612-64-6	$C_8H_{10}N_2O$	150.18	alkylaryl
N-Nitroso-*tert*-Butylphenylamine	NTBPhA	Sigma-Aldrich	97.0%	24642-84-0	$C_{10}H_{14}N_2O$	178.24	alkylaryl
N-Nitroso-4-Methylaminopyridine	NMAPY	Enamine	95.0%	16219-99-1	$C_6H_7N_3O$	137.14	alkylheteroaryl
N-Nitrososimazine	NSIM	TRC	99.5%	6494-81-1	$C_7H_{11}ClN_6O$	230.66	alkylheteroaryl
N-Nitrosoatrazine	NATR	TRC	98.0%	56525-09-8	$C_8H_{13}ClN_6O$	244.68	alkylheteroaryl
N-Nitrosoglyphosate	NGLP	TRC	98.0%	56516-72-4	$C_3H_7N_2O_6P$	198.07	dialkyl
N-Nitrosobetahistine	NBTH	TRC	97.0%	32635-81-7	$C_8H_{11}N_3O$	165.20	dialkyl
N-Nitrosoephedrine	NEPD	TRC	98.0%	17608-59-2	$C_{10}H_{14}N_2O_2$	194.23	dialkyl
N-Nitrosofenfluramine	NFFA	TRC	97.0%	19023-40-6	$C_{12}H_{15}F_3N_2O$	260.26	dialkyl
N-Nitrosonortriptyline	NNTP	TRC	97.0%	55855-42-0	$C_{19}H_{20}N_2O$	292.38	dialkyl
N-Nitrosodesipramine	NDSP	TRC	97.0%	57164-17-7	$C_{18}H_{21}N_3O$	295.39	dialkyl
N-Nitrosofluoxetine	NFXT	TRC	98.0%	150494-06-7	$C_{17}H_{17}F_3N_2O_2$	338.33	dialkyl
N-Nitrosopropranolol	NPPN	TRC	97.0%	84418-35-9	$C_{16}H_{20}N_2O_3$	288.35	dialkyl
N-Nitrosoatenolol	NATN	TRC	96.0%	134720-04-0	$C_{14}H_{21}N_3O_4$	295.34	dialkyl
N-Nitrosometoprolol	NMTP	TRC	95.0%	134720-05-1	$C_{15}H_{24}N_2O_4$	296.37	dialkyl
N-Nitrososotalol	NSOT	TRC	98.0%	134720-07-3	$C_{12}H_{19}N_3O_4S$	301.36	dialkyl
N-Nitrosonadolol	NNAD	TRC	97.0%	134720-06-2	$C_{17}H_{26}N_2O_5$	338.40	dialkyl
N-Nitrosonebivolol	NNBV	TRC	98.0%	1391051-68-5	$C_{22}H_{24}F_2N_2O_5$	434.44	dialkyl
N-Nitrosodesloratadine	NDLT	TRC	96.0%	1246819-22-6	$C_{19}H_{18}ClN_3O$	339.82	cyclic
N-Nitrosonorfloxacin	NNFX	TRC	98.0%	74011-53-3	$C_{16}H_{17}FN_4O_4$	348.33	heterocyclic
N-Nitrosociprofloxacin	NCFX	TRC	97.0%	864443-44-7	$C_{17}H_{17}FN_4O_4$	360.35	heterocyclic
N-Nitrosodiclofenac	NDCF	TRC	98.0%	66505-80-4	$C_{14}H_{10}Cl_2N_2O_3$	325.15	diaryl
N-Nitrosoiminostilbene	NISB	TRC	97.0%	38652-29-8	$C_{14}H_{10}N_2O$	222.25	cyclic diaryl
N-Nitrosoiminodibenzyl	NIDB	TRC	97.0%	7458-08-4	$C_{14}H_{12}N_2O$	224.26	cyclic diaryl
N-Nitrosodimethyl-d_6-Amine	NDMA-d_6	C/D/N	98% D	17829-05-9	$C_2D_6N_2O$	80.12	ILIS
N-Nitrosodiethyl-d_{10}-Amine	NDEA-d_{10}	C/D/N	99% D	1219794-54-3	$C_4D_{10}N_2O$	112.20	ILIS
N-Nitrosodi-*n*-Propyl-d_{14}-Amine	NDPA-d_{14}	C/D/N	98% D	93951-96-3	$C_6D_{14}N_2O$	144.28	ILIS
N-Nitrosopyrrolidine-d_8	NPYR-d_8	C/D/N	98% D	1219802-09-1	$C_4D_8N_2O$	108.17	ILIS
N-Nitrosomorpholine-d_8	NMOR-d_8	C/D/N	98% D	1219805-76-1	$C_4D_8N_2O_2$	124.17	ILIS
N-Nitrosodiphenyl-2,2',4,4',6,6'-d_6-Amine	NDPhA-d_6	C/D/N	98% D	93951-95-2	$C_{12}H_4D_6N_2O$	204.26	ILIS
N-Nitrosobis(2-Hydroxyethyl)-d_8-Amine	NDELA-d_8	C/D/N	98% D	1173019-53-8	$C_4H_2D_8N_2O$	142.18	ILIS
N-Methyl-d_3-*N*-Nitrosoglycine	NSAR-d_3	C/D/N	99% D	1189871-94-0	$C_3H_3D_3N_2O_3$	121.11	ILIS

TRC = Toronto Research Chemicals. C/D/N = C/D/N Isotopes. ILIS = Isotope-labeled internal standard.

**Table S2.** Molecular structures of *N*-nitrosamines			
Compound	SMILES	Molecular Structure	Category
NDMA (EPA 521/8270)	CN(C)N=O	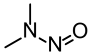	dialkyl
NMEA (EPA 521/8270)	CCN(C)N=O	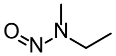	dialkyl
NDEA (EPA 521/8270)	CCN(CC)N=O	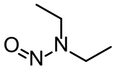	dialkyl
NDPA (EPA 521/8270)	CCCN(CCC)N=O	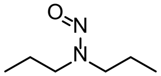	dialkyl
NDBA (EPA 521/8270)	CCCCN(CCCC)N=O	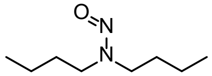	dialkyl
NPYR (EPA 521/8270)	C1CCN(C1)N=O	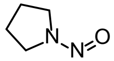	cyclic (pyrrolidine)
NPIP (EPA 521/8270)	C1CCN(CC1)N=O	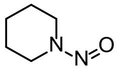	cyclic (piperidine)
NMOR (EPA 8270)	C1COCCN1N=O	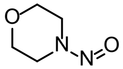	heterocyclic (morpholine)
NDPhA (EPA 8270)	C1=CC=C(C=C1)N(C2=CC=CC=C2)N=O	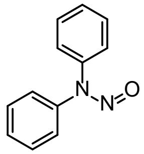	diaryl (aniline)
NDELA	C(CO)N(CCO)N=O	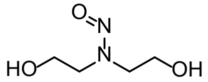	dialkyl
NSAR	CN(CC(=O)O)N=O	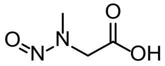	dialkyl *N*-nitrosoamino acid
NPRO	C1C[C@H](N(C1)N=O)C(=O)O	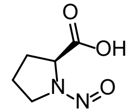	cyclic (pyrrolidine) *N*-nitrosoamino acid
NPIC	C1CCN(C(C1)C(=O)O)N=O	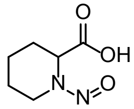	cyclic (piperidine) *N*-nitrosoamino acid
NHPRO	C1[C@H](CN([C@@H]1C(=O)O)N=O)O	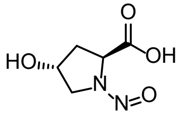	cyclic (pyrrolidine) *N*-nitrosoamino acid

**Table S2.** Molecular structures of *N*-nitrosamines (continued)			
Compound	SMILES	Molecular Structure	Category
NTCA	C1C(N(CS1)N=O)C(=O)O	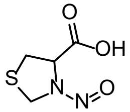	heterocyclic (thiazolidine) *N*-nitrosoamino acid
NMTCA	CC1N(C(CS1)C(=O)O)N=O	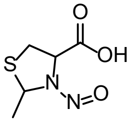	heterocyclic (thiazolidine) *N*-nitrosoamino acid
NNN	C1CC(N(C1)N=O)C2=CN=CC=C2	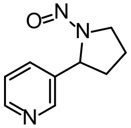	cyclic (pyrrolidine) tobacco-specific *N*-nitrosamine
NAT	C1C=CCN(C1C2=CN=CC=C2)N=O	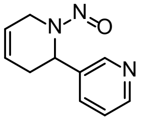	cyclic (tetrahydropyridine) tobacco-specific *N*-nitrosamine
NAB	C1CCN(C(C1)C2=CN=CC=C2)N=O	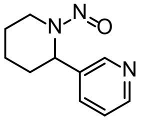	cyclic tobacco-specific *N*-nitrosamine
NNK	CN(CCCC(=O)C1=CN=CC=C1)N=O	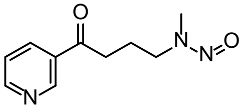	dialkyl tobacco-specific *N*-nitrosamine
NNAL	CN(CCCC(C1=CN=CC=C1)O)N=O	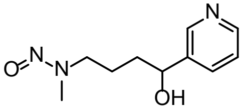	dialkyl tobacco-specific *N*-nitrosamine
NMIPA	CC(C)N(C)N=O	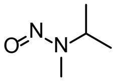	dialkyl
NEPA	CCCN(CC)N=O	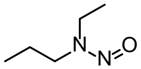	dialkyl
NMBA	CCCCN(C)N=O	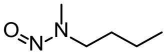	dialkyl
NMIBA	CC(C)CN(C)N=O	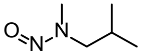	dialkyl
NTBMA	CC(C)(C)N(C)N=O	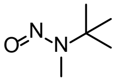	dialkyl

Table S2. Molecular structures of *N*-nitrosamines (continued)

Compound	SMILES	Molecular Structure	Category
NMAA	CCCCCN(C)N=O	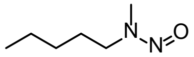	dialkyl
NDIPA	CC(C)N(C(C)C)N=O	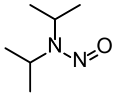	dialkyl
NTBEA	CCN(C(C)(C)C)N=O	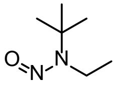	dialkyl
NMDEA	CN(C)CCN(C)N=O	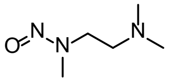	dialkyl
NDIBA	CC(C)CN(CC(C)C)N=O	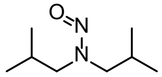	dialkyl
NDAA	CCCCCN(CCCCC)N=O	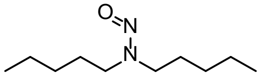	dialkyl
NEBzA	CCN(CC1=CC=CC=C1)N=O	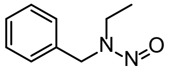	dialkyl
NDBzA	C1=CC=C(C=C1)CN(CC2=CC=CC=C2)N=O	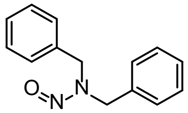	dialkyl
NMPIP	CC1CCN(CC1)N=O	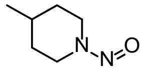	cyclic (piperidine)
NPYRM	C1CC(N(C1)N=O)CO	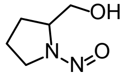	cyclic (pyrrolidine)
NPPZ	C1CN(CCN1)N=O	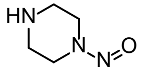	heterocyclic (piperazine)
NPhPPZ	C1CN(CCN1C2=CC=CC=C2)N=O	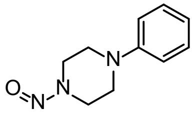	heterocyclic (piperazine)
NIND	C1CN(C2=CC=CC=C21)N=O	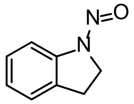	cyclic alkylaryl (indoline)

Compound	SMILES	Molecular Structure	Category
NTHQ	C1CC2=CC=CC=C2N(C1)N=O	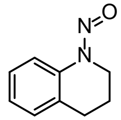	cyclic alkylaryl (tetrahydroquinoline)
NMChA	CN(C1CCCCC1)N=O	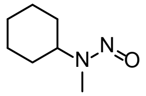	alkylcycloalkyl
NDChA	C1CCC(CC1)N(C2CCCCC2)N=O	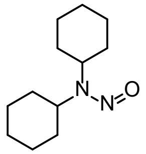	dicycloalkyl
NMPhA	CN(C1=CC=CC=C1)N=O	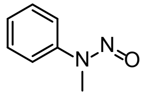	alkylaryl (aniline)
NEPhA	CCN(C1=CC=CC=C1)N=O	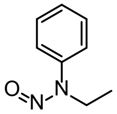	alkylaryl (aniline)
NTBPhA	CC(C)(C)N(C1=CC=CC=C1)N=O	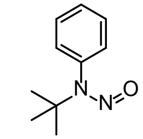	alkylaryl (aniline)
NMAPY	CN(C1=CC=NC=C1)N=O	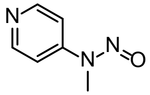	alkylheteroaryl (aminopyridine)
NSIM	CCNC1=NC(=NC(=N1)Cl)N(CC)N=O	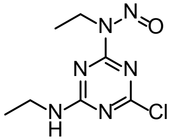	alkylheteroaryl (triazine) *N*-nitroso pesticide
NATR	CCN(C1=NC(=NC(=N1)NC(C)C)Cl)N=O	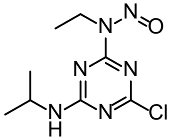	alkylheteroaryl (triazine) *N*-nitroso pesticide
NGLP	C(C(=O)O)N(CP(=O)(O)O)N=O	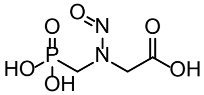	dalkyl *N*-nitroso pesticide

Table: **Table S2.** Molecular structures of *N*-nitrosamines (continued)

Table S2. Molecular structures of *N*-nitrosamines (continued)			
Compound	SMILES	Molecular Structure	Category
NBTH	CN(CCC1=CC=CC=N1)N=O	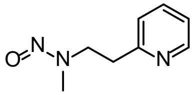	dialkyl *N*-nitroso pharmaceutical
NEPD	CC(C(C1=CC=CC=C1)O)N(C)N=O	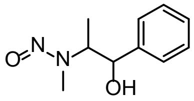	dialkyl *N*-nitroso pharmaceutical
NFFA	CCN(C(C)CC1=CC(=CC=C1)C(F)(F)F)N=O	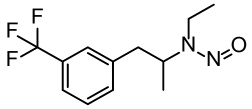	dialkyl *N*-nitroso pharmaceutical
NNTP	CN(CCC=C1C2=CC=CC=C2CCC3=CC=CC=C31)N=O	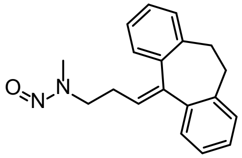	dialkyl *N*-nitroso pharmaceutical
NDSP	CN(CCCN1C2=CC=CC=C2CCC3=CC=CC=C31)N=O	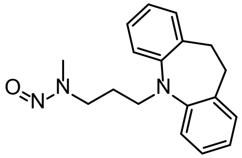	dialkyl *N*-nitroso pharmaceutical
NFXT	CN(CCC(C1=CC=CC=C1)OC2=CC=C(C=C2)C(F)(F)F)N=O	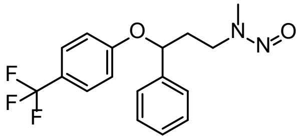	dialkyl *N*-nitroso pharmaceutical
NPPN	CC(C)N(CC(COC1=CC=CC2=CC=CC=C21)O)N=O	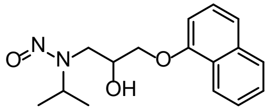	dialkyl *N*-nitroso pharmaceutical
NATN	CC(C)N(CC(COC1=CC=C(C=C1)CC(=O)N)O)N=O	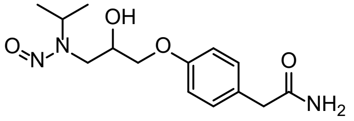	dialkyl *N*-nitroso pharmaceutical
NMTP	CC(C)N(CC(COC1=CC=C(C=C1)CCOC)O)N=O	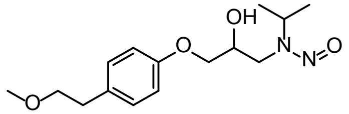	dialkyl *N*-nitroso pharmaceutical

**Table S2.** Molecular structures of *N*-nitrosamines (continued)			
Compound	SMILES	Molecular Structure	Category
NSOT	CC(C)N(CC(C1=CC=C(C=C1)NS(=O)(=O)C)O)N=O	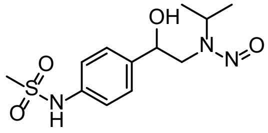	dialkyl<br>*N*-nitroso pharmaceutical
NNAD	CC(C)(C)N(CC(COC1=CC=CC2=C1CC(C(C2)O)O)O)N=O	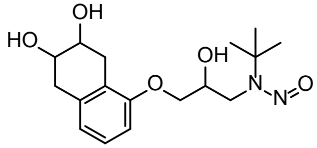	dialkyl<br>*N*-nitroso pharmaceutical
NNBV	C1CC2=C(C=CC(=C2)F)OC1C(CN(CC(C3CCC4=C(O3)C=CC(=C4)F)O)N=O)O	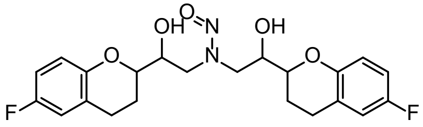	dialkyl<br>*N*-nitroso pharmaceutical
NDLT	C1CC2=C(C=CC(=C2)Cl)C(=C3CCN(CC3)N=O)C4=C1C=CC=N4	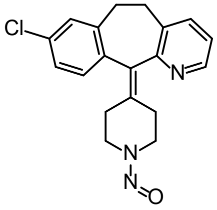	cyclic<br>(piperidine)<br>*N*-nitroso pharmaceutical
NNFX	CCN1C=C(C(=O)C2=CC(=C(C=C21)N3CCN(CC3)N=O)F)C(=O)O	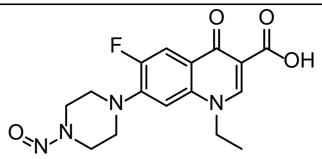	heterocyclic<br>(piperazine)<br>*N*-nitroso pharmaceutical
NCFX	C1CC1N2C=C(C(=O)C3=CC(=C(C=C32)N4CCN(CC4)N=O)F)C(=O)O	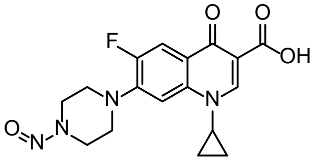	heterocyclic<br>(piperazine)<br>*N*-nitroso pharmaceutical
NDCF	C1=CC=C(C(=C1)CC(=O)O)N(C2=C(C=CC=C2Cl)Cl)N=O	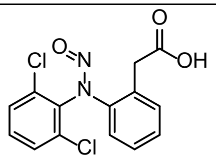	diaryl<br>*N*-nitroso pharmaceutical
NISB	C1=CC=C2C(=C1)C=CC3=CC=CC=C3N2N=O	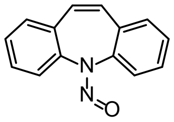	cyclic diaryl<br>*N*-nitroso pharmaceutical<br>building block

**Table S2.** Molecular structures of *N*-nitrosamines (continued)			
Compound	SMILES	Molecular Structure	Category
NIDB	C1CC2=CC=CC=C2N(C3=CC=CC=C31)N=O	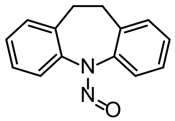	cyclic diaryl<br>*N*-nitroso pharmaceutical building block
EPA 521 = *N*-Nitrosamines targeted by EPA Method 521. EPA 8270 = *N*-Nitrosamines targeted by EPA Method 8270.			

2. Chemiluminescence signal stability of *N*-nitrosodimethylamine (NDMA) standards

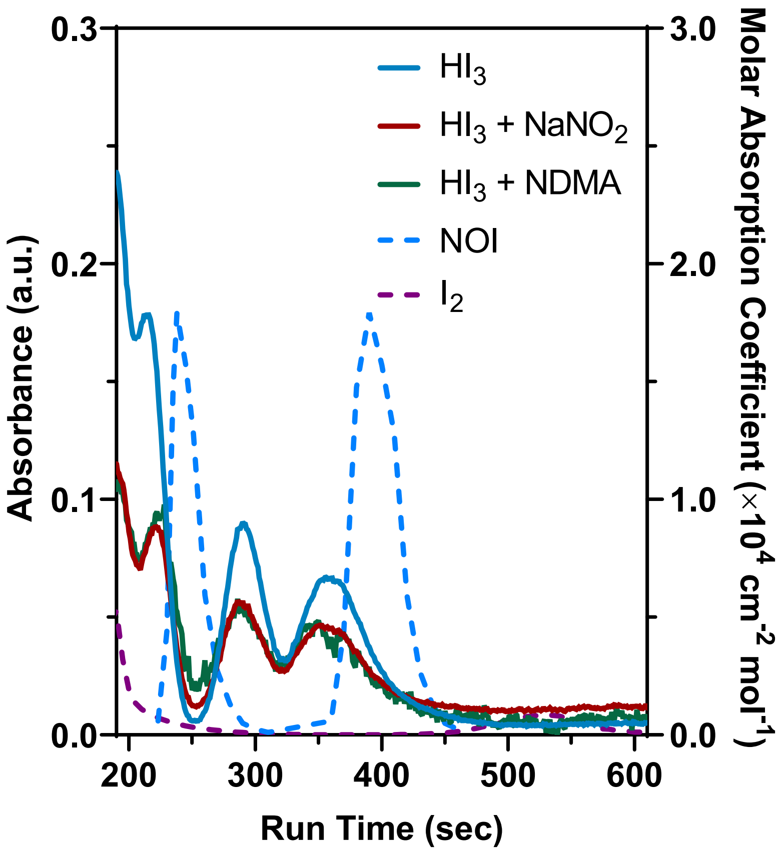

Figure S1. Gas-phase UV-visible absorbance spectra measured above a 100-µL aliquot of the freshly prepared acidic triiodide solution (HI_3) and the same aliquot of the acidic triiodide solution spiked with 50 µL of 2.5 mM sodium nitrite solution[2] (HI_3 + $NaNO_2$) or 100 µL of 10 µM NDMA solution (HI_3 + NDMA) overlaid with the molar absorption coefficients (plotted on the right *y*-axis) of nitrosyl iodide (NOI) and iodine (I_2) documented in Jet Propulsion Laboratory Publication 10-6.[3] Gas-phase spectrophotometric measurements were performed in a Starna Cells 9-Q-10-GL14-S quartz cuvette (1-cm pathlength; flushed with N_2 through a GL14-S septum cap) using a Thermo Scientific Evolution 201 UV-visible spectrophotometer. UV-visible absorbance spectra were recorded from 190 to 800 nm in 1-nm increments and corrected for blank and long-wavelength baseline.

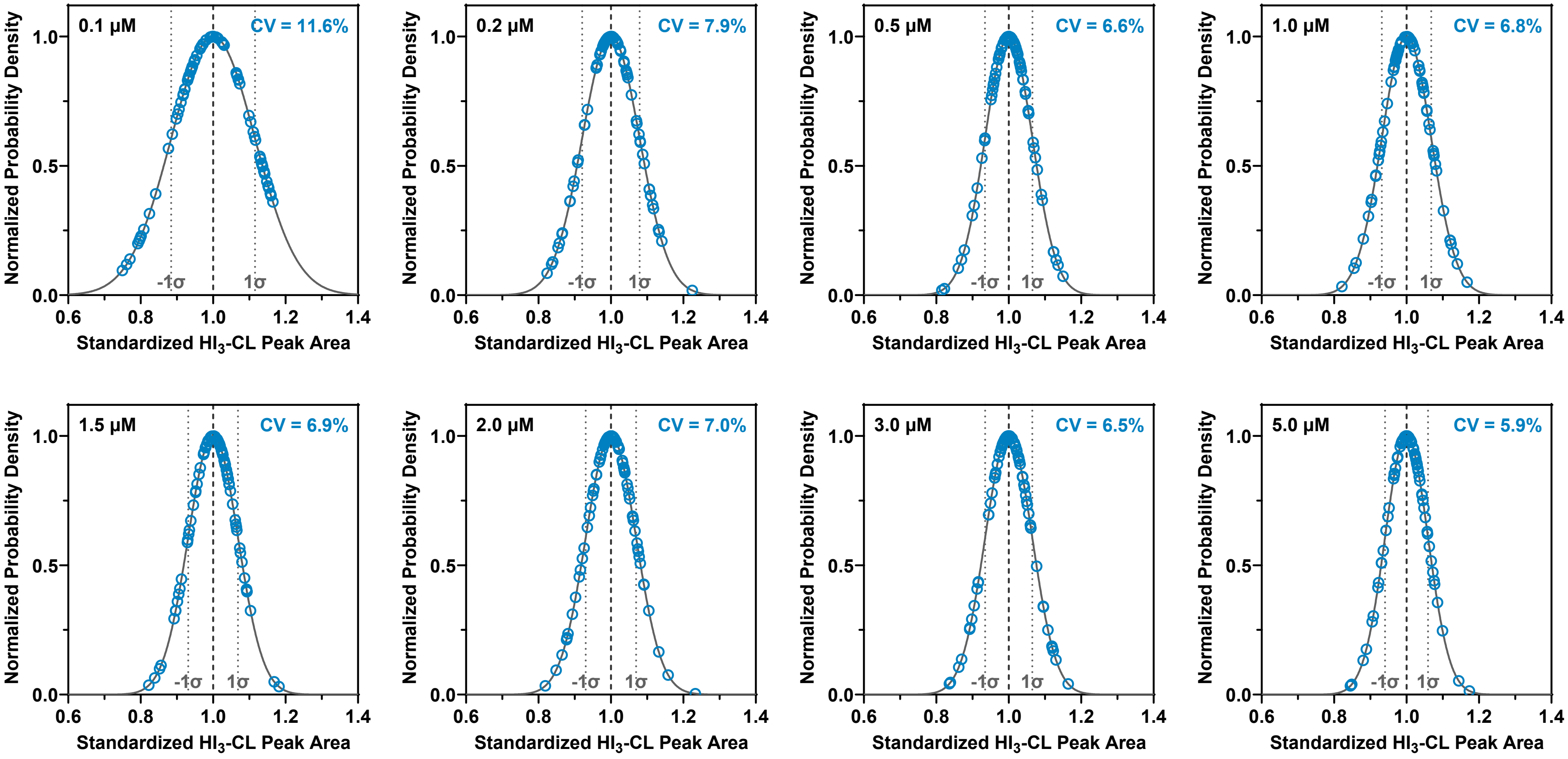

Figure S2. Probability distribution of the standardized chemiluminescence peak areas of *N*-nitrosodimethylamine (NDMA) calibration standards (0.1, 0.2, 0.5, 1.0, 1.5, 2.0, 3.0, and 5.0 μM) measured by the acidic triiodide-chemiluminescence (HI_3-CL) method. On each plot, the grey solid line represents the Gaussian distribution fit of standardized chemiluminescence peak areas resulting from replicate injections (n = 80) of NDMA standards. The grey dotted lines mark one standard deviation (±1σ) from the mean. CV represents the coefficient of variation of standardized chemiluminescence peak areas.

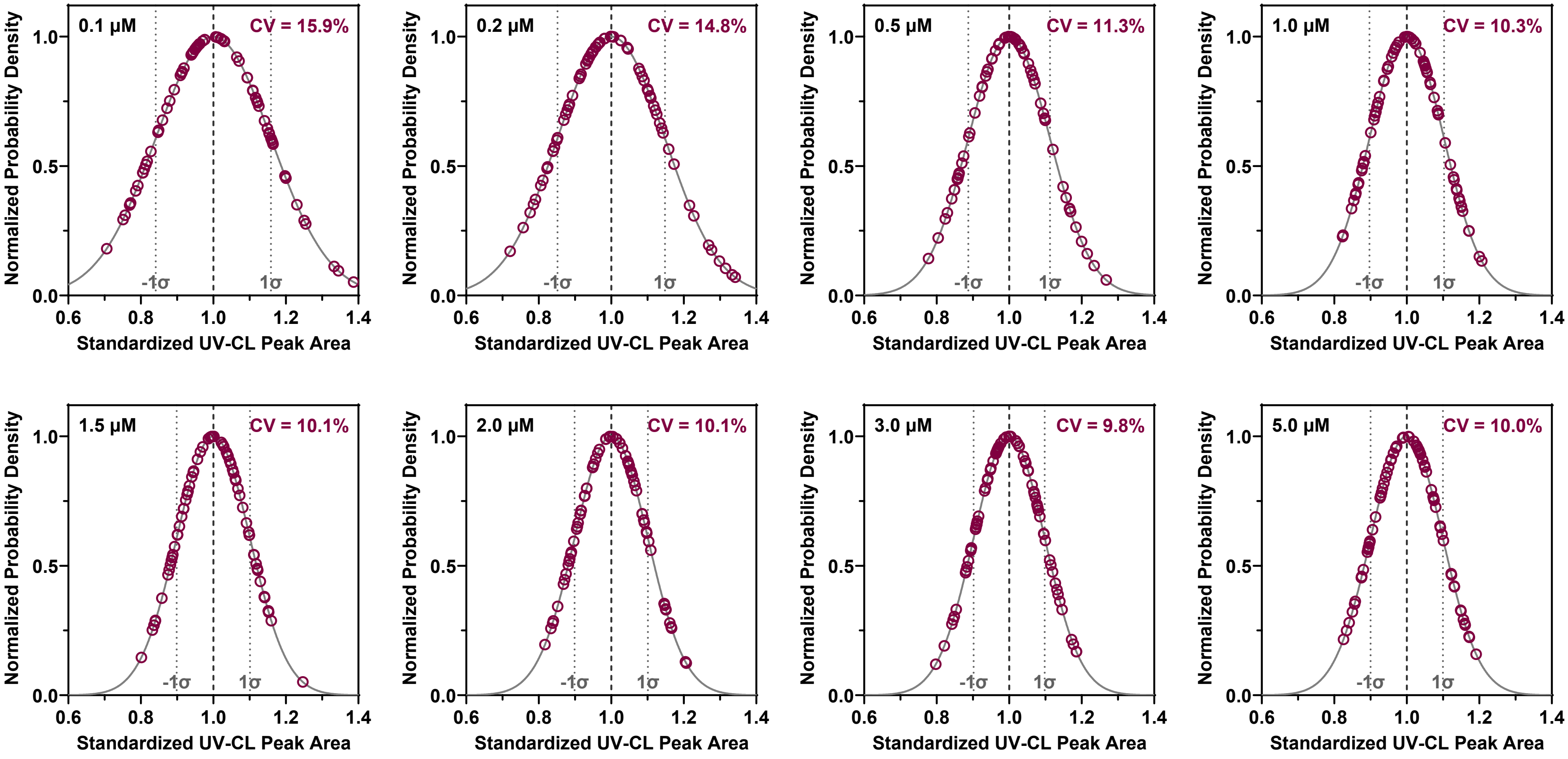

Figure S3. Probability distribution of the standardized chemiluminescence peak areas of *N*-nitrosodimethylamine (NDMA) calibration standards (0.1, 0.2, 0.5, 1.0, 1.5, 2.0, 3.0, and 5.0 μM) measured by the UV photolysis-chemiluminescence (UV-CL) method. On each plot, the grey solid line represents the Gaussian distribution fit of standardized chemiluminescence peak areas resulting from replicate injections (n = 73) of NDMA standards. The grey dotted lines mark one standard deviation (±1σ) from the mean. CV represents the coefficient of variation of standardized chemiluminescence peak areas.

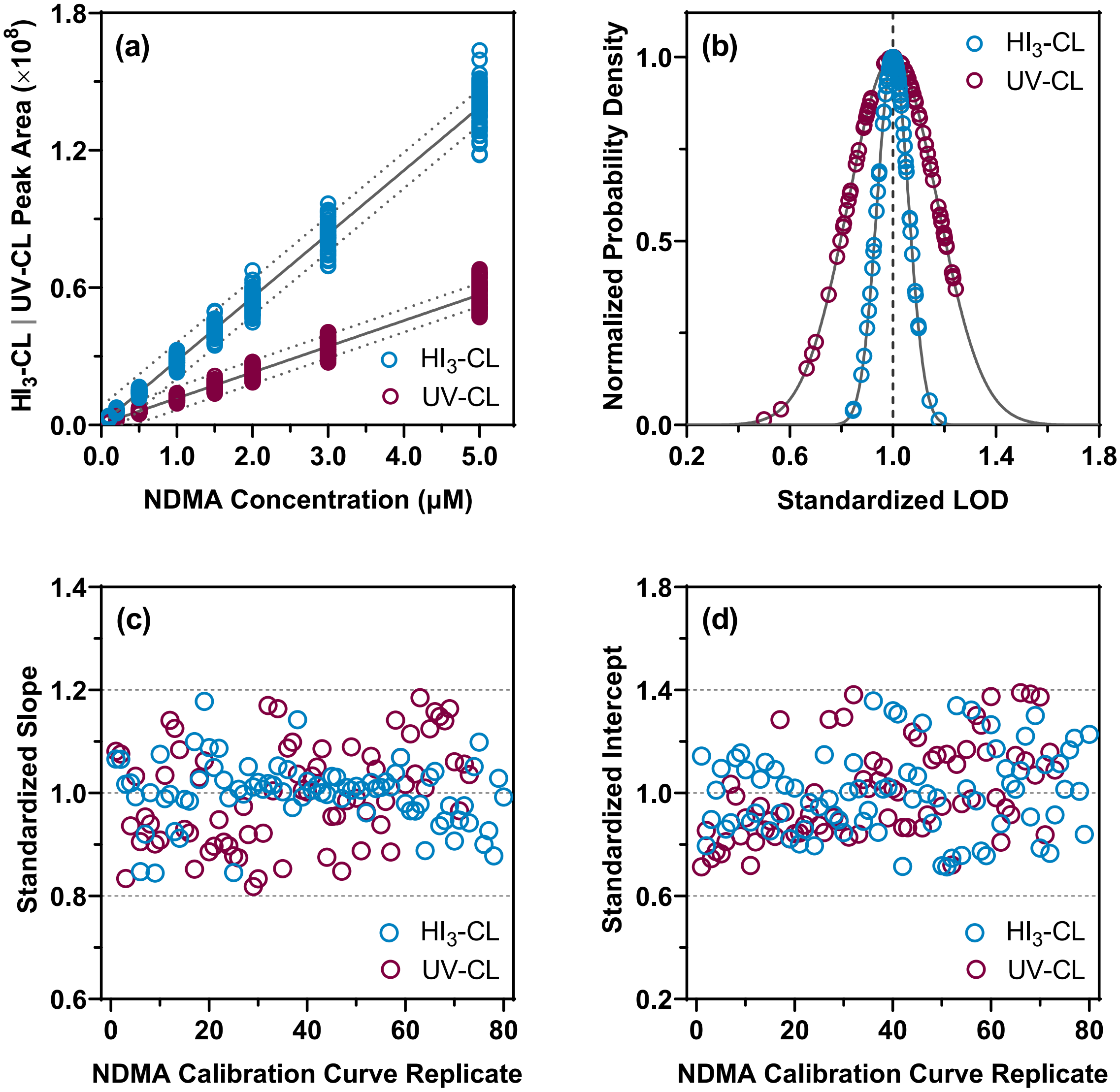

Figure S4. Long-term signal reproducibility of the acidic triiodide-chemiluminescence (HI_3-CL) and UV photolysis-chemiluminescence (UV-CL) methods: **(a)** Calibration curves of *N*-nitrosodimethylamine (NDMA) standards measured by the HI_3-CL ($n = 80$) and UV-CL ($n = 73$) methods over the study period (~1 year). The grey solid lines represent the mean slopes derived from the linear least squares regression analysis of chemiluminescence peak areas (averaged from replicate injections) versus NDMA concentrations (0.1, 0.2, 0.5, 1.0, 1.5, 2.0, 3.0, and 5.0 μM). The grey dotted lines bracket the 95% confidence interval for the slopes. **(b)** Probability distribution of the standardized limits of detection (LODs) of NDMA calibration curves measured by the HI_3-CL ($n = 80$) and UV-CL ($n = 73$) methods. The grey solid lines represent the Gaussian distribution fit of standardized LODs. **(c)** Standardized slopes of NDMA calibration curves measured by the HI_3-CL ($n = 80$) and UV-CL ($n = 73$) methods. **(d)** Standardized intercepts of NDMA calibration curves measured by the HI_3-CL ($n = 80$) and UV-CL ($n = 73$) methods.

3. HI_3-CL and UV-CL method performance

For each *N*-nitrosamine or their equal-molar mixture, the conversion efficiencies,[4] mass-based limits of detection ($LODs_{Mass}$),[5] limits of detection (LODs),[6] and limits of quantification (LOQs)[6] were calculated for the HI_3-CL and UV-CL methods by the following equations and summarized in **Tables S3** and **S4**:

$$\text{Conversion Efficiency} = \frac{\text{Slope}_{N\text{-Nitrosamine Calibration Cruve}}}{\text{Slope}_{\text{NDMA Calibration Cruve}}} \times 100\% \tag{S1}$$

$$\text{LOD}_{\text{Mass}}\ (\text{pmol}) = \text{LOD}\ (\mu\text{M}) \times \frac{10^6\ \text{pmol}}{1\ \mu\text{mol}} \times 100\ \mu\text{L} \times \frac{1\ \text{mL}}{1000\ \mu\text{L}} \times \frac{1\ \text{L}}{1000\ \text{mL}} \tag{S2}$$

$$\text{LOD}\ (\mu\text{M}) = 3.3 \times \frac{\text{Standard Deviation of } y\text{-Intercept}_{N\text{-Nitrosamine Calibration Cruve}}}{\text{Slope}_{N\text{-Nitrosamine Calibration Cruve}}} \tag{S3}$$

$$\text{LOQ}\ (\mu\text{M}) = 10 \times \frac{\text{Standard Deviation of } y\text{-Intercept}_{N\text{-Nitrosamine Calibration Cruve}}}{\text{Slope}_{N\text{-Nitrosamine Calibration Cruve}}} \tag{S4}$$

Table S3. Conversion efficiencies and detection limits of *N*-nitrosamines measured by the HI_3-CL method						
Compound	Conversion Efficiency	R^2	LOD_{Mass} (pmol)	LOD (μM)	LOQ (μM)	Category
NDMA	100(±8)%	0.9996	6.7(±2.7)	0.07(±0.03)	0.20(±0.08)	dialkyl
NMEA	99(±8)%	0.9996	6.2(±2.6)	0.06(±0.03)	0.19(±0.08)	dialkyl
NDEA	96(±8)%	0.9997	5.4(±2.2)	0.05(±0.02)	0.17(±0.07)	dialkyl
NDPA	95(±13)%	0.9994	7.5(±4.4)	0.08(±0.04)	0.23(±0.13)	dialkyl
NDBA	98(±9)%	0.9996	5.8(±2.4)	0.06(±0.02)	0.18(±0.07)	dialkyl
NPYR	97(±11)%	0.9994	7.0(±4.1)	0.07(±0.04)	0.21(±0.12)	cyclic
NPIP	93(±8)%	0.9998	4.6(±1.9)	0.05(±0.02)	0.14(±0.06)	cyclic
NMOR	94(±16)%	0.9987	9.8(±5.7)	0.10(±0.06)	0.30(±0.17)	heterocyclic
NDPhA	83(±11)%	0.9985	11.7(±4.8)	0.12(±0.05)	0.35(±0.14)	diaryl
NDELA	78(±8)%	0.9992	8.5(±3.5)	0.08(±0.03)	0.26(±0.11)	dialkyl
NSAR	76(±11)%	0.9982	12.9(±5.3)	0.13(±0.05)	0.39(±0.16)	dialkyl
NPRO	83(±9)%	0.9994	7.3(±3.0)	0.07(±0.03)	0.22(±0.09)	cyclic
NPIC	96(±9)%	0.9996	6.1(±2.5)	0.06(±0.03)	0.19(±0.08)	cyclic
NHPRO	68(±12)%	0.9986	12.1(±7.0)	0.12(±0.07)	0.37(±0.21)	cyclic
NTCA	68(±9)%	0.9983	12.6(±5.2)	0.13(±0.05)	0.38(±0.16)	heterocyclic
NMTCA	67(±10)%	0.9973	16.1(±6.6)	0.16(±0.07)	0.49(±0.20)	heterocyclic
NNN	91(±10)%	0.9992	8.8(±3.6)	0.09(±0.04)	0.27(±0.11)	cyclic
NAT	90(±10)%	0.9992	8.9(±3.6)	0.09(±0.04)	0.27(±0.11)	cyclic
NAB	90(±10)%	0.9990	9.6(±3.9)	0.10(±0.04)	0.29(±0.12)	cyclic
NNK	92(±9)%	0.9993	7.9(±3.2)	0.08(±0.03)	0.24(±0.10)	dialkyl
NNAL	94(±9)%	0.9993	8.0(±3.3)	0.08(±0.03)	0.24(±0.10)	dialkyl
NMIPA	90(±10)%	0.9992	8.6(±3.5)	0.09(±0.04)	0.26(±0.11)	dialkyl
NEPA	92(±10)%	0.9993	8.4(±3.4)	0.08(±0.03)	0.25(±0.10)	dialkyl
NMBA	92(±10)%	0.9993	8.2(±3.4)	0.08(±0.03)	0.25(±0.10)	dialkyl
NMIBA	86(±12)%	0.9983	12.6(±5.2)	0.13(±0.05)	0.38(±0.16)	dialkyl
NTBMA	83(±11)%	0.9983	12.6(±5.1)	0.13(±0.05)	0.38(±0.16)	dialkyl
NMAA	93(±10)%	0.9993	7.9(±3.2)	0.08(±0.03)	0.24(±0.10)	dialkyl
NDIPA	76(±11)%	0.9984	12.3(±5.0)	0.12(±0.05)	0.37(±0.15)	dialkyl
NTBEA	81(±9)%	0.9990	9.8(±4.0)	0.10(±0.04)	0.30(±0.12)	dialkyl
NMDEA	91(±11)%	0.9989	10.4(±4.3)	0.10(±0.04)	0.32(±0.13)	dialkyl
NDIBA	38(±12)%	0.9887	43.3(±17.7)	0.43(±0.18)	1.31(±0.54)	dialkyl
NDAA	98(±10)%	0.9994	7.5(±3.1)	0.08(±0.03)	0.23(±0.09)	dialkyl
NEBzA	83(±10)%	0.9986	11.3(±4.6)	0.11(±0.05)	0.34(±0.14)	dialkyl
NDBzA	81(±11)%	0.9982	12.9(±5.3)	0.13(±0.05)	0.39(±0.16)	dialkyl
NMPIP	97(±7)%	0.9997	5.1(±2.1)	0.05(±0.02)	0.15(±0.06)	cyclic
NPYRM	93(±10)%	0.9995	7.2(±2.9)	0.07(±0.03)	0.22(±0.09)	cyclic
NPPZ	98(±10)%	0.9993	7.9(±3.2)	0.08(±0.03)	0.24(±0.10)	heterocyclic
NPhPPZ	77(±11)%	0.9983	12.8(±5.2)	0.13(±0.05)	0.39(±0.16)	heterocyclic
NIND	98(±10)%	0.9995	6.7(±2.8)	0.07(±0.03)	0.20(±0.08)	cyclic alkylaryl
NTHQ	85(±11)%	0.9988	10.6(±4.3)	0.11(±0.04)	0.32(±0.13)	cyclic alkylaryl
NMChA	88(±10)%	0.9990	9.7(±4.0)	0.10(±0.04)	0.29(±0.12)	alkylcycloalkyl
NDChA	91(±10)%	0.9994	7.8(±3.2)	0.08(±0.03)	0.24(±0.10)	dicycloalkyl
NMPhA	77(±11)%	0.9982	13.1(±5.4)	0.13(±0.05)	0.40(±0.16)	alkylaryl
NEPhA	86(±10)%	0.9989	10.3(±4.2)	0.10(±0.04)	0.31(±0.13)	alkylaryl
NTBPhA	79(±11)%	0.9985	11.9(±4.9)	0.12(±0.05)	0.36(±0.15)	alkylaryl
NMAPY	97(±9)%	0.9994	7.3(±3.0)	0.07(±0.03)	0.22(±0.09)	alkylheteroaryl
NSIM	71(±11)%	0.9979	14.2(±5.8)	0.14(±0.06)	0.43(±0.18)	alkylheteroaryl
NATR	72(±11)%	0.9980	13.8(±5.6)	0.14(±0.06)	0.42(±0.17)	alkylheteroaryl
NGLP	91(±10)%	0.9991	9.4(±3.9)	0.09(±0.04)	0.29(±0.12)	dialkyl
NBTH	92(±9)%	0.9995	6.6(±2.7)	0.07(±0.03)	0.20(±0.08)	dialkyl
NEPD	91(±9)%	0.9992	8.4(±3.5)	0.08(±0.03)	0.26(±0.10)	dialkyl
NFFA	89(±9)%	0.9993	7.8(±3.2)	0.08(±0.03)	0.24(±0.10)	dialkyl
NNTP	90(±9)%	0.9993	8.4(±3.4)	0.08(±0.03)	0.25(±0.10)	dialkyl

**Table S3.** Conversion efficiencies and detection limits of *N*-nitrosamines measured by the HI_3-CL method (continued)						
Compound	Conversion Efficiency	R^2	LOD_{Mass} (pmol)	LOD (μM)	LOQ (μM)	Category
NDSP	90(±11)%	0.9992	8.7(±3.6)	0.09(±0.04)	0.26(±0.11)	dialkyl
NFXT	94(±9)%	0.9995	7.1(±2.9)	0.07(±0.03)	0.22(±0.09)	dialkyl
NPPN	88(±14)%	0.9990	9.8(±5.6)	0.10(±0.06)	0.30(±0.17)	dialkyl
NATN	92(±13)%	0.9993	7.8(±4.5)	0.08(±0.05)	0.24(±0.14)	dialkyl
NMTP	89(±9)%	0.9991	9.1(±3.7)	0.09(±0.04)	0.28(±0.11)	dialkyl
NSOT	92(±10)%	0.9989	10.3(±4.2)	0.10(±0.04)	0.31(±0.13)	dialkyl
NNAD	85(±11)%	0.9988	10.4(±4.3)	0.10(±0.04)	0.32(±0.13)	dialkyl
NNBV	87(±10)%	0.9991	9.1(±3.7)	0.09(±0.04)	0.28(±0.11)	dialkyl
NDLT	96(±8)%	0.9995	6.9(±2.8)	0.07(±0.03)	0.21(±0.09)	cyclic
NNFX	91(±11)%	0.9990	9.8(±4.0)	0.10(±0.04)	0.30(±0.12)	heterocyclic
NCFX	89(±11)%	0.9985	11.9(±4.9)	0.12(±0.05)	0.36(±0.15)	heterocyclic
NDCF	82(±10)%	0.9988	10.6(±4.3)	0.11(±0.04)	0.32(±0.13)	diaryl
NISB	84(±10)%	0.9988	10.7(±4.4)	0.11(±0.04)	0.33(±0.13)	cyclic diaryl
NIDB	86(±9)%	0.9990	9.7(±4.0)	0.10(±0.04)	0.29(±0.12)	cyclic diaryl
Mixture	90(±9)%	0.9991	9.3(±5.4)	0.09(±0.05)	0.28(±0.16)	equal-molar

LOD_{Mass} = Limit of detection (mass-based) at 100 μL injection volume. LOD = Limit of detection at 100 μL injection volume. LOQ = Limit of quantification at 100 μL injection volume. Values are given as mean ± (95% confidence interval).

Table S4. Conversion efficiencies and detection limits of *N*-nitrosamines measured by the UV-CL method						
Compound	Conversion Efficiency	R^2	LOD_{Mass} (pmol)	LOD (μM)	LOQ (μM)	Category
NDMA	100(±9)%	0.9995	6.6(±2.7)	0.07(±0.03)	0.20(±0.08)	dialkyl
NMEA	99(±9)%	0.9994	7.4(±3.0)	0.07(±0.03)	0.22(±0.09)	dialkyl
NDEA	93(±10)%	0.9995	7.2(±2.9)	0.07(±0.03)	0.22(±0.09)	dialkyl
NDPA	91(±5)%	0.9992	8.6(±3.5)	0.09(±0.03)	0.26(±0.11)	dialkyl
NDBA	93(±8)%	0.9993	7.9(±3.2)	0.08(±0.03)	0.24(±0.10)	dialkyl
NPYR	96(±8)%	0.9995	6.8(±2.8)	0.07(±0.03)	0.21(±0.08)	cyclic
NPIP	94(±10)%	0.9993	8.3(±3.4)	0.08(±0.03)	0.25(±0.10)	cyclic
NMOR	95(±9)%	0.9992	8.4(±3.4)	0.08(±0.03)	0.26(±0.10)	heterocyclic
NDPhA	91(±11)%	0.9987	11.2(±4.6)	0.11(±0.05)	0.34(±0.14)	diaryl
NDELA	93(±11)%	0.9993	8.2(±3.4)	0.08(±0.03)	0.25(±0.10)	dialkyl
NSAR	83(±10)%	0.9989	10.4(±4.2)	0.10(±0.04)	0.31(±0.13)	dialkyl
NPRO	80(±10)%	0.9988	10.8(±4.4)	0.11(±0.04)	0.33(±0.13)	cyclic
NPIC	65(±9)%	0.9983	12.8(±5.2)	0.13(±0.05)	0.39(±0.16)	cyclic
NHPRO	84(±9)%	0.9990	9.9(±4.1)	0.10(±0.04)	0.30(±0.12)	cyclic
NTCA	65(±11)%	0.9973	16.0(±6.5)	0.16(±0.07)	0.48(±0.20)	heterocyclic
NMTCA	64(±11)%	0.9969	17.2(±7.0)	0.17(±0.07)	0.52(±0.21)	heterocyclic
NNN	93(±9)%	0.9993	8.2(±3.4)	0.08(±0.03)	0.25(±0.10)	cyclic
NAT	95(±7)%	0.9994	7.4(±3.0)	0.07(±0.03)	0.22(±0.09)	cyclic
NAB	96(±11)%	0.9991	9.3(±3.8)	0.09(±0.04)	0.28(±0.12)	cyclic
NNK	85(±10)%	0.9989	10.3(±4.2)	0.10(±0.04)	0.31(±0.13)	dialkyl
NNAL	69(±11)%	0.9976	15.0(±6.1)	0.15(±0.06)	0.46(±0.19)	dialkyl
NMIPA	81(±9)%	0.9989	10.2(±4.1)	0.10(±0.04)	0.31(±0.13)	dialkyl
NEPA	91(±8)%	0.9994	7.4(±3.0)	0.07(±0.03)	0.22(±0.09)	dialkyl
NMBA	92(±9)%	0.9995	6.6(±2.7)	0.07(±0.03)	0.20(±0.08)	dialkyl
NMIBA	84(±8)%	0.9990	9.6(±3.9)	0.10(±0.04)	0.29(±0.12)	dialkyl
NTBMA	75(±9)%	0.9986	11.5(±4.7)	0.12(±0.05)	0.35(±0.14)	dialkyl
NMAA	93(±9)%	0.9993	8.4(±3.4)	0.08(±0.03)	0.25(±0.10)	dialkyl
NDIPA	70(±10)%	0.9978	14.5(±5.9)	0.15(±0.06)	0.44(±0.18)	dialkyl
NTBEA	71(±8)%	0.9988	10.8(±4.4)	0.11(±0.04)	0.33(±0.13)	dialkyl
NMDEA	81(±9)%	0.9989	10.0(±4.1)	0.10(±0.04)	0.30(±0.12)	dialkyl
NDIBA	21(±8)%	0.9824	41.2(±16.8)	0.41(±0.17)	1.25(±0.51)	dialkyl
NDAA	91(±10)%	0.9991	9.4(±3.8)	0.09(±0.04)	0.28(±0.12)	dialkyl
NEBzA	72(±11)%	0.9980	13.9(±5.7)	0.14(±0.06)	0.42(±0.17)	dialkyl
NDBzA	85(±9)%	0.9991	9.4(±3.8)	0.09(±0.04)	0.28(±0.12)	dialkyl
NMPIP	95(±9)%	0.9991	9.2(±3.8)	0.09(±0.04)	0.28(±0.11)	cyclic
NPYRM	71(±9)%	0.9987	10.9(±4.5)	0.11(±0.04)	0.33(±0.14)	cyclic
NPPZ	81(±11)%	0.9985	11.9(±4.9)	0.12(±0.05)	0.36(±0.15)	heterocyclic
NPhPPZ	89(±10)%	0.9994	7.6(±3.1)	0.08(±0.03)	0.23(±0.09)	heterocyclic
NIND	94(±9)%	0.9991	9.2(±3.7)	0.09(±0.04)	0.28(±0.11)	cyclic alkylaryl
NTHQ	97(±10)%	0.9991	9.3(±3.8)	0.09(±0.04)	0.28(±0.12)	cyclic alkylaryl
NMChA	91(±11)%	0.9990	9.8(±4.0)	0.10(±0.04)	0.30(±0.12)	alkylcycloalkyl
NDChA	89(±10)%	0.9992	8.6(±3.5)	0.09(±0.04)	0.26(±0.11)	dicycloalkyl
NMPhA	86(±10)%	0.9991	9.3(±3.8)	0.09(±0.04)	0.28(±0.12)	alkylaryl
NEPhA	84(±11)%	0.9988	10.5(±4.3)	0.10(±0.04)	0.32(±0.13)	alkylaryl
NTBPhA	70(±10)%	0.9985	11.9(±4.9)	0.12(±0.05)	0.36(±0.15)	alkylaryl
NMAPY	92(±10)%	0.9994	7.5(±3.1)	0.07(±0.03)	0.23(±0.09)	alkylheteroaryl
NSIM	87(±10)%	0.9993	8.0(±3.3)	0.08(±0.03)	0.24(±0.10)	alkylheteroaryl
NATR	81(±9)%	0.9989	10.1(±4.1)	0.10(±0.04)	0.31(±0.13)	alkylheteroaryl
NGLP	97(±10)%	0.9995	6.9(±2.8)	0.07(±0.03)	0.21(±0.09)	dialkyl
NBTH	97(±10)%	0.9992	8.4(±3.5)	0.08(±0.03)	0.26(±0.10)	dialkyl
NEPD	90(±10)%	0.9991	9.1(±3.7)	0.09(±0.04)	0.28(±0.11)	dialkyl
NFFA	88(±10)%	0.9989	10.0(±4.1)	0.10(±0.04)	0.30(±0.12)	dialkyl
NNTP	90(±11)%	0.9991	9.3(±3.8)	0.09(±0.04)	0.28(±0.12)	dialkyl

**Table S4.** Conversion efficiencies and detection limits of *N*-nitrosamines measured by the UV-CL method (continued)						
Compound	Conversion Efficiency	R^2	LOD_{Mass} (pmol)	LOD (μM)	LOQ (μM)	Category
NDSP	92(±11)%	0.9992	8.5(±3.5)	0.09(±0.03)	0.26(±0.11)	dialkyl
NFXT	96(±10)%	0.9996	6.2(±2.5)	0.06(±0.03)	0.19(±0.08)	dialkyl
NPPN	94(±10)%	0.9989	8.2(±3.3)	0.08(±0.03)	0.25(±0.10)	dialkyl
NATN	91(±14)%	0.9992	8.8(±5.1)	0.09(±0.05)	0.27(±0.15)	dialkyl
NMTP	88(±11)%	0.9986	11.4(±4.7)	0.11(±0.05)	0.35(±0.14)	dialkyl
NSOT	93(±10)%	0.9992	8.6(±3.5)	0.09(±0.04)	0.26(±0.11)	dialkyl
NNAD	92(±14)%	0.9992	8.4(±5.0)	0.08(±0.05)	0.25(±0.15)	dialkyl
NNBV	94(±9)%	0.9993	8.1(±3.3)	0.08(±0.03)	0.24(±0.10)	dialkyl
NDLT	87(±9)%	0.9992	9.0(±3.7)	0.09(±0.04)	0.27(±0.11)	cyclic
NNFX	83(±12)%	0.9982	12.9(±5.3)	0.13(±0.05)	0.39(±0.16)	heterocyclic
NCFX	85(±11)%	0.9983	12.8(±5.2)	0.13(±0.05)	0.39(±0.16)	heterocyclic
NDCF	82(±12)%	0.9981	13.3(±5.4)	0.13(±0.05)	0.40(±0.17)	diaryl
NISB	81(±10)%	0.9987	11.1(±4.6)	0.11(±0.05)	0.34(±0.14)	cyclic diaryl
NIDB	80(±16)%	0.9982	13.1(±7.6)	0.13(±0.08)	0.40(±0.23)	cyclic diaryl
Mixture	91(±11)%	0.9990	9.6(±5.5)	0.10(±0.06)	0.29(±0.17)	equal-molar

LOD_{Mass} = Limit of detection (mass-based) at 100 μL injection volume. LOD = Limit of detection at 100 μL injection volume. LOQ = Limit of quantification at 100 μL injection volume. Values are given as mean ± (95% confidence interval).

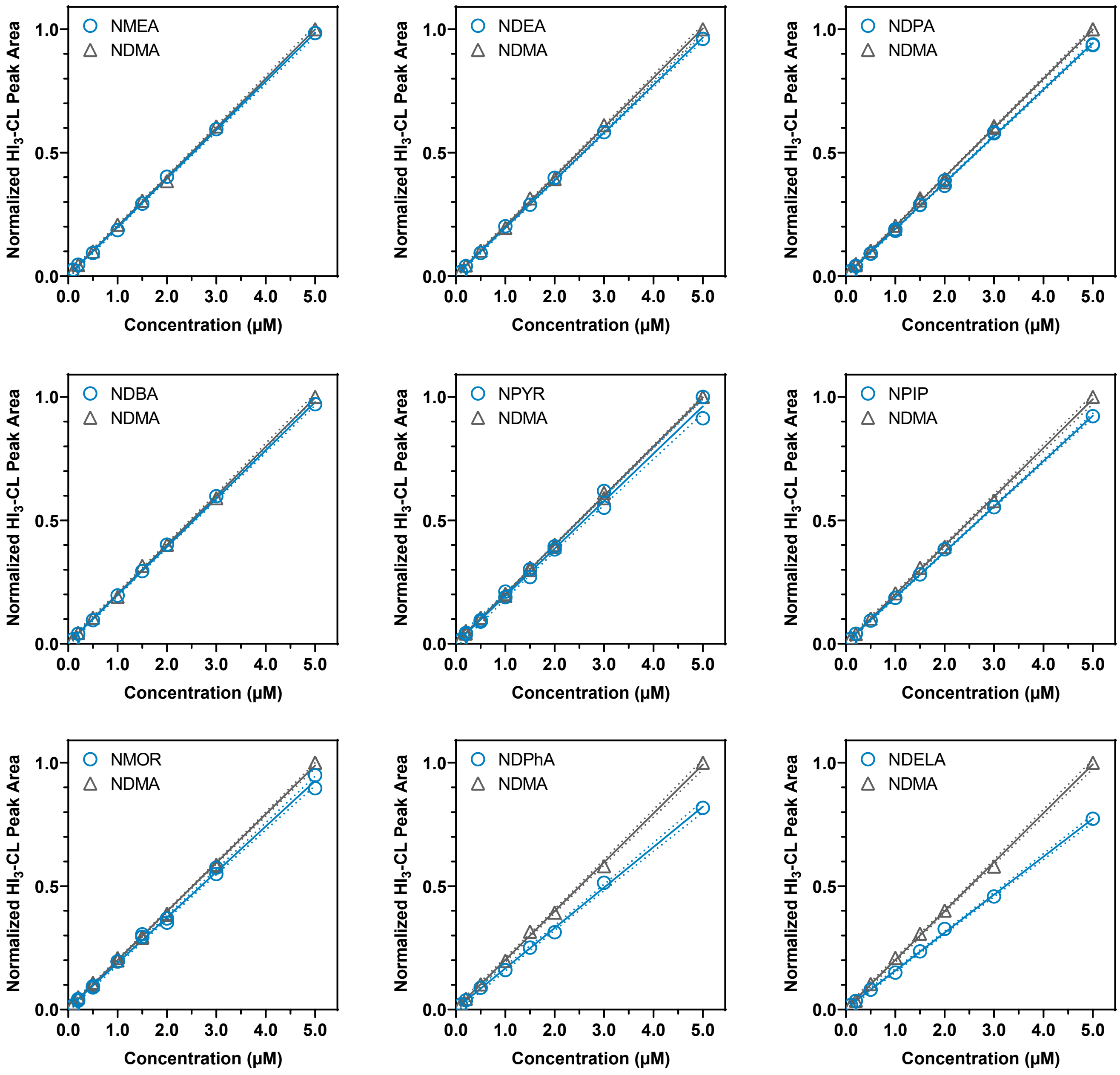

Figure S5a. Calibration curves of *N*-nitrosomethylethylamine (NMEA), *N*-nitrosodiethylamine (NDEA), *N*-nitrosodipropylamine (NDPA), *N*-nitrosodibutylamine (NDBA), *N*-nitrosopyrrolidine (NPYR), *N*-nitrosopiperidine (NPIP), *N*-nitrosomorpholine (NMOR), *N*-nitrosodiphenylamine (NDPhA), or *N*-nitrosodiethanolamine (NDELA) versus *N*-nitrosodimethylamine (NDMA) measured by the acidic triiodide-chemiluminescence (HI_3-CL) method. On each plot, the grey solid line represents the slope derived from the linear least squares regression analysis of normalized chemiluminescence peak areas versus NDMA concentrations (0.1, 0.2, 0.5, 1.0, 1.5, 2.0, 3.0, and 5.0 μM). The grey dotted lines bracket the 95% confidence interval for the slope. The blue solid line represents the slope derived from the linear least squares regression analysis of normalized chemiluminescence peak areas versus *N*-nitroso compound concentrations (0.1, 0.2, 0.5, 1.0, 1.5, 2.0, 3.0, and 5.0 μM). The blue dotted lines bracket the 95% confidence interval for the slope.

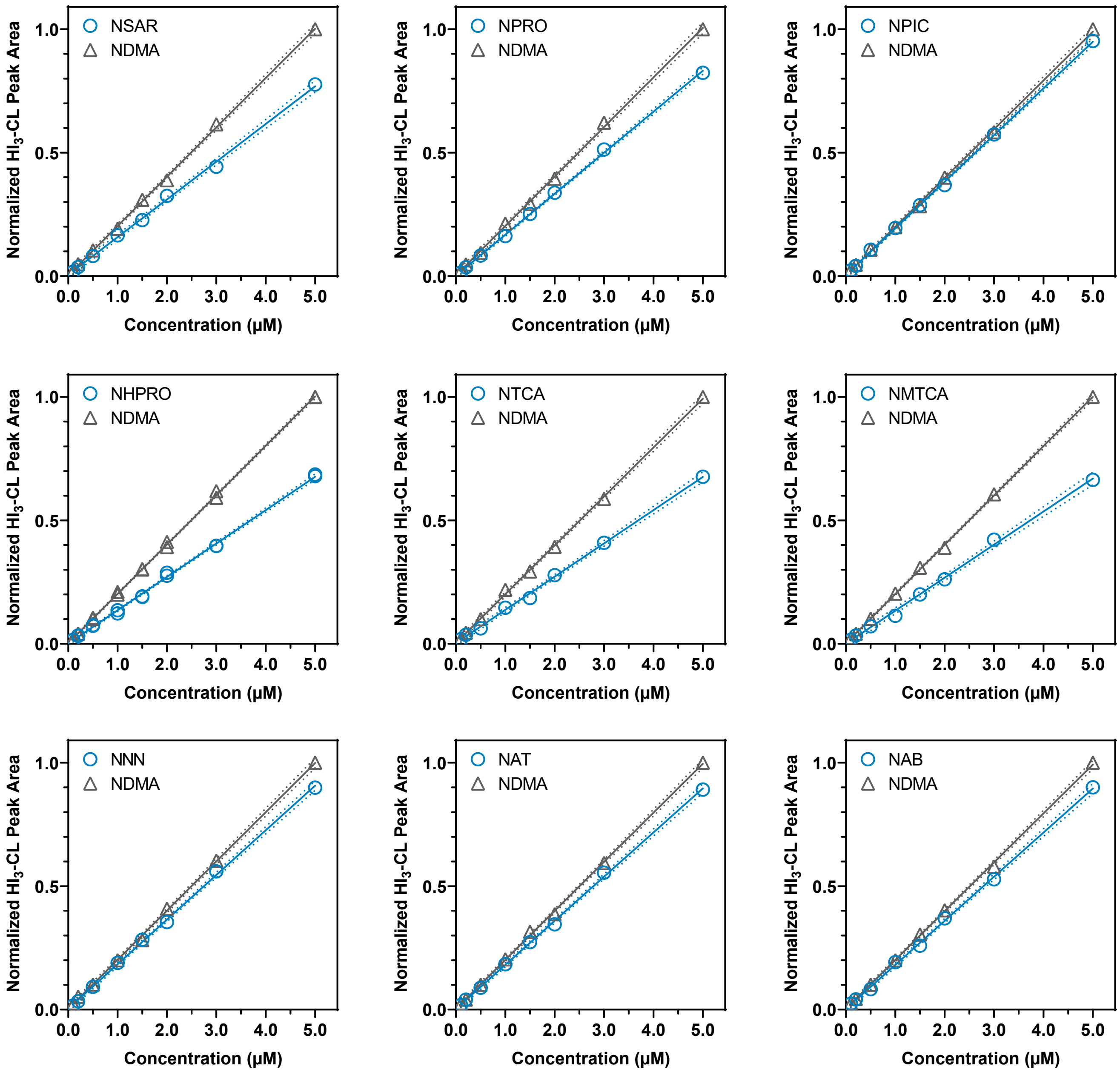

Figure S5b. Calibration curves of *N*-nitrososarcosine (NSAR), *N*-nitrosoproline (NPRO), *N*-nitrosopipecolic acid (NPIC), *N*-nitrosohydroxyproline (NHPRO), *N*-nitrosothiazolidine-4-carboxylic acid (NTCA), *N*-nitroso-2-methylthiazolidine 4-carboxylic acid (NMTCA), *N*-nitrosonornicotine (NNN), *N*-nitrosoanatabine (NAT), or *N*-nitrosoanabasine (NAB) versus *N*-nitrosodimethylamine (NDMA) measured by the acidic triiodide-chemiluminescence (HI_3-CL) method. On each plot, the grey solid line represents the slope derived from the linear least squares regression analysis of normalized chemiluminescence peak areas versus NDMA concentrations (0.1, 0.2, 0.5, 1.0, 1.5, 2.0, 3.0, and 5.0 μM). The grey dotted lines bracket the 95% confidence interval for the slope. The blue solid line represents the slope derived from the linear least squares regression analysis of normalized chemiluminescence peak areas versus *N*-nitroso compound concentrations (0.1, 0.2, 0.5, 1.0, 1.5, 2.0, 3.0, and 5.0 μM). The blue dotted lines bracket the 95% confidence interval for the slope.

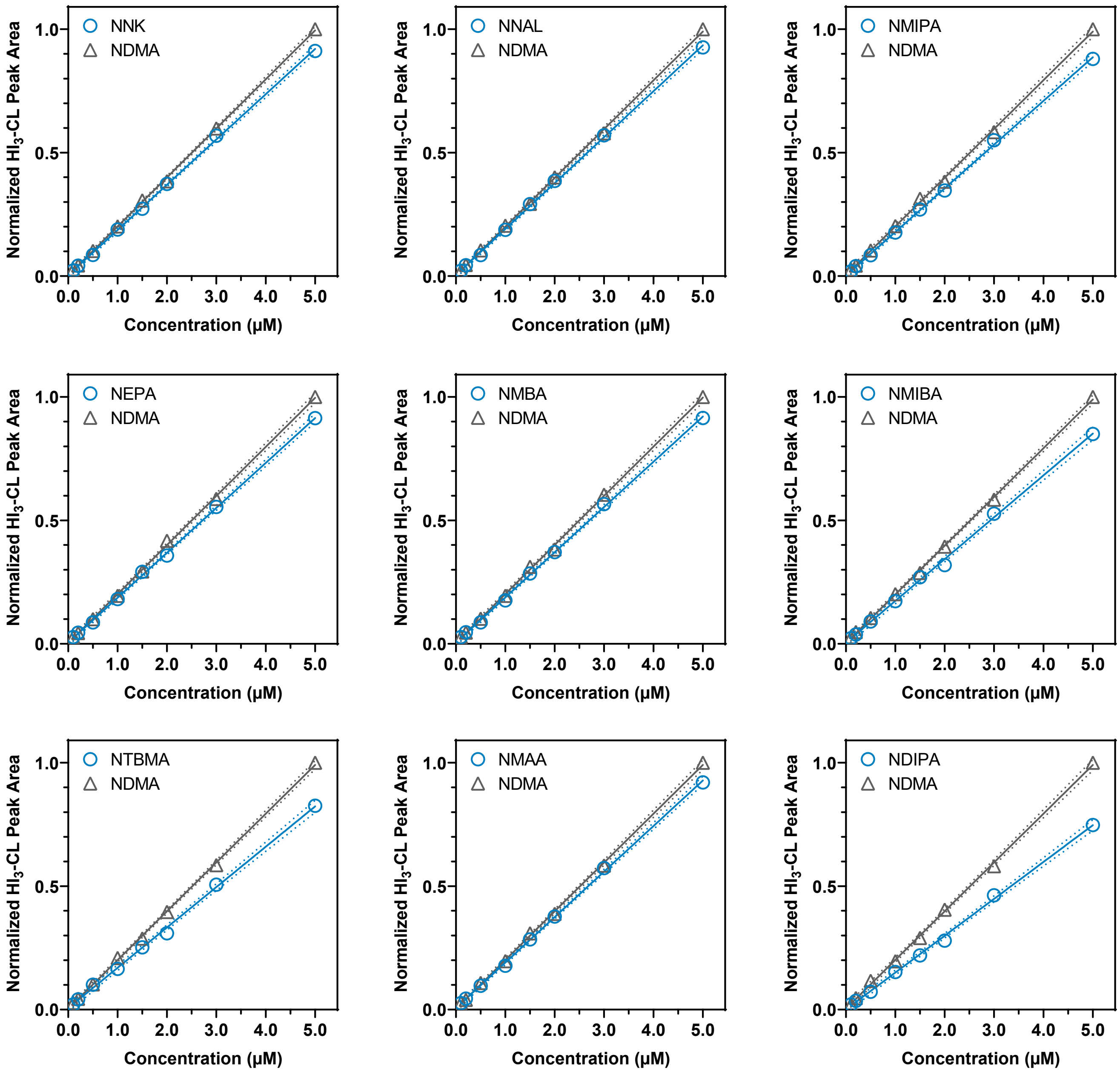

Figure S5c. Calibration curves of 4-(methylnitrosamino)-1-(3-pyridyl)-1-butanone (NNK), 4-(methylnitrosamino)-1-(3-pyridyl)-1-butanol (NNAL), *N*-nitrosomethylisopropylamine (NMIPA), *N*-nitrosoethylpropylamine (NEPA), *N*-nitrosomethylbutylamine (NMBA), *N*-nitrosomethylisobutylamine (NMIBA), *N*-nitroso-*tert*-butylmethylamine (NTBMA), *N*-nitrosomethylamylamine (NMAA), or *N*-nitrosodiisopropylamine (NDIPA) versus *N*-nitrosodimethylamine (NDMA) measured by the acidic triiodide-chemiluminescence (HI_3-CL) method. On each plot, the grey solid line represents the slope derived from the linear least squares regression analysis of normalized chemiluminescence peak areas versus NDMA concentrations (0.1, 0.2, 0.5, 1.0, 1.5, 2.0, 3.0, and 5.0 μM). The grey dotted lines bracket the 95% confidence interval for the slope. The blue solid line represents the slope derived from the linear least squares regression analysis of normalized chemiluminescence peak areas versus *N*-nitroso compound concentrations (0.1, 0.2, 0.5, 1.0, 1.5, 2.0, 3.0, and 5.0 μM). The blue dotted lines bracket the 95% confidence interval for the slope.

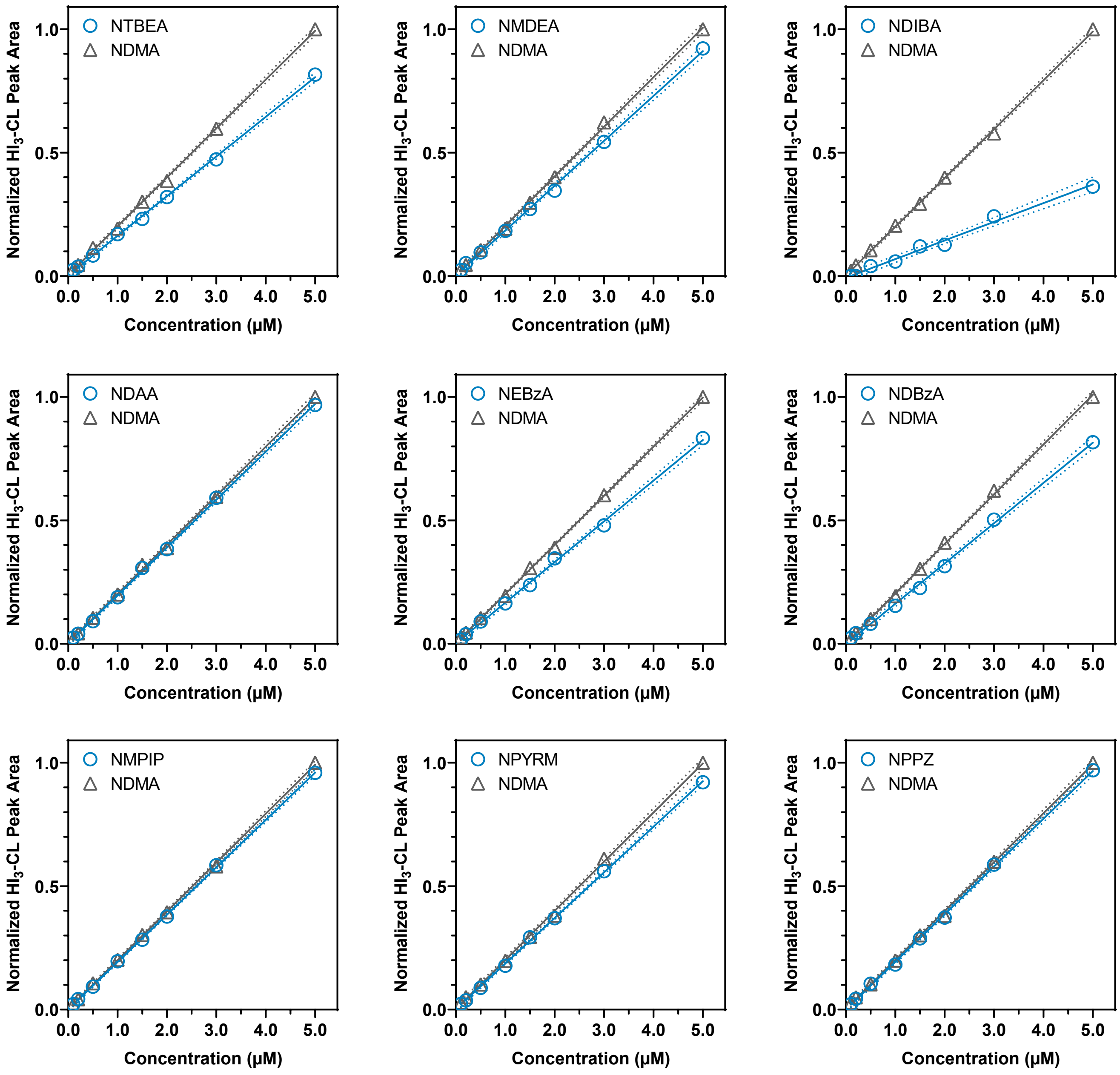

Figure S5d. Calibration curves of *N*-nitroso-*tert*-butylethylamine (NTBEA), *N*-nitrosomethyl-*N,N*-dimethylethylamine (NMDEA), *N*-nitrosodiisobutylamine (NDIBA), *N*-nitrosodiamylamine (NDAA), *N*-nitrosoethylbenzylamine (NEBzA), *N*-nitrosodibenzylamine (NDBzA), 1-nitroso-4-methylpiperidine (NMPIP), *N*-nitroso-2-pyrrolidinmethanol (NPYRM), or *N*-nitrosopiperazine (NPPZ) versus *N*-nitrosodimethylamine (NDMA) measured by the acidic triiodide-chemiluminescence (HI_3-CL) method. On each plot, the grey solid line represents the slope derived from the linear least squares regression analysis of normalized chemiluminescence peak areas versus NDMA concentrations (0.1, 0.2, 0.5, 1.0, 1.5, 2.0, 3.0, and 5.0 μM). The grey dotted lines bracket the 95% confidence interval for the slope. The blue solid line represents the slope derived from the linear least squares regression analysis of normalized chemiluminescence peak areas versus *N*-nitroso compound concentrations (0.1, 0.2, 0.5, 1.0, 1.5, 2.0, 3.0, and 5.0 μM). The blue dotted lines bracket the 95% confidence interval for the slope.

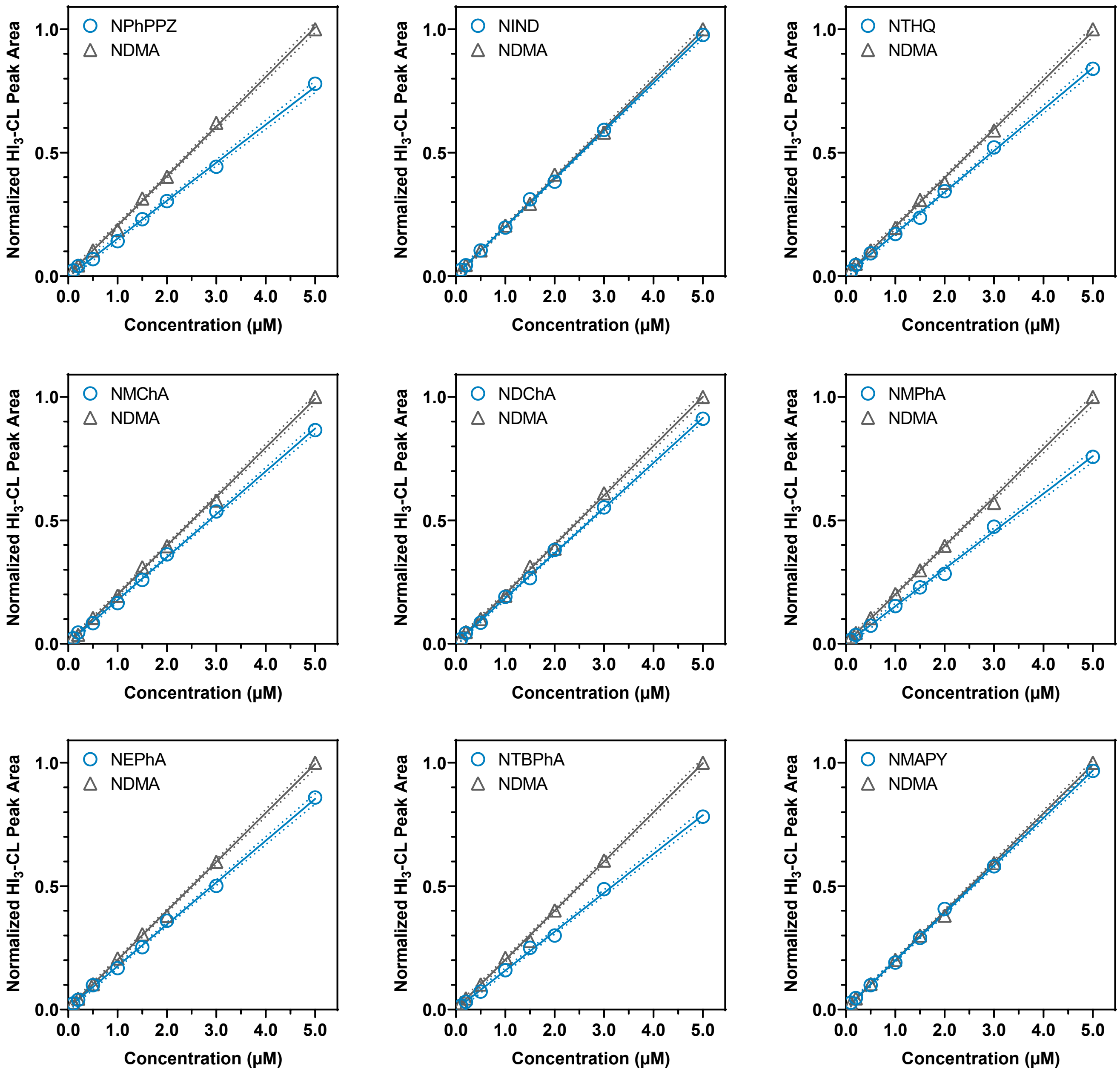

Figure S5e. Calibration curves of 1-nitroso-4-phenylpiperazine (NPhPPZ), *N*-nitrosoindoline (NIND), 1-nitroso-1,2,3,4-tetrahydroquinoline (NTHQ), *N*-nitrosomethylcyclohexylamine (NMChA), *N*-nitrosodicyclohexylamine (NDChA), *N*-nitrosomethylphenylamine (NMPhA), *N*-nitrosoethylphenylamine (NEPhA), *N*-nitroso-*tert*-butylphenylamine (NTBPhA), or *N*-nitroso-4-methylaminopyridine (NMAPY) versus *N*-nitrosodimethylamine (NDMA) measured by the acidic triiodide-chemiluminescence (HI_3-CL) method. On each plot, the grey solid line represents the slope derived from the linear least squares regression analysis of normalized chemiluminescence peak areas versus NDMA concentrations (0.1, 0.2, 0.5, 1.0, 1.5, 2.0, 3.0, and 5.0 μM). The grey dotted lines bracket the 95% confidence interval for the slope. The blue solid line represents the slope derived from the linear least squares regression analysis of normalized chemiluminescence peak areas versus *N*-nitroso compound concentrations (0.1, 0.2, 0.5, 1.0, 1.5, 2.0, 3.0, and 5.0 μM). The blue dotted lines bracket the 95% confidence interval for the slope.

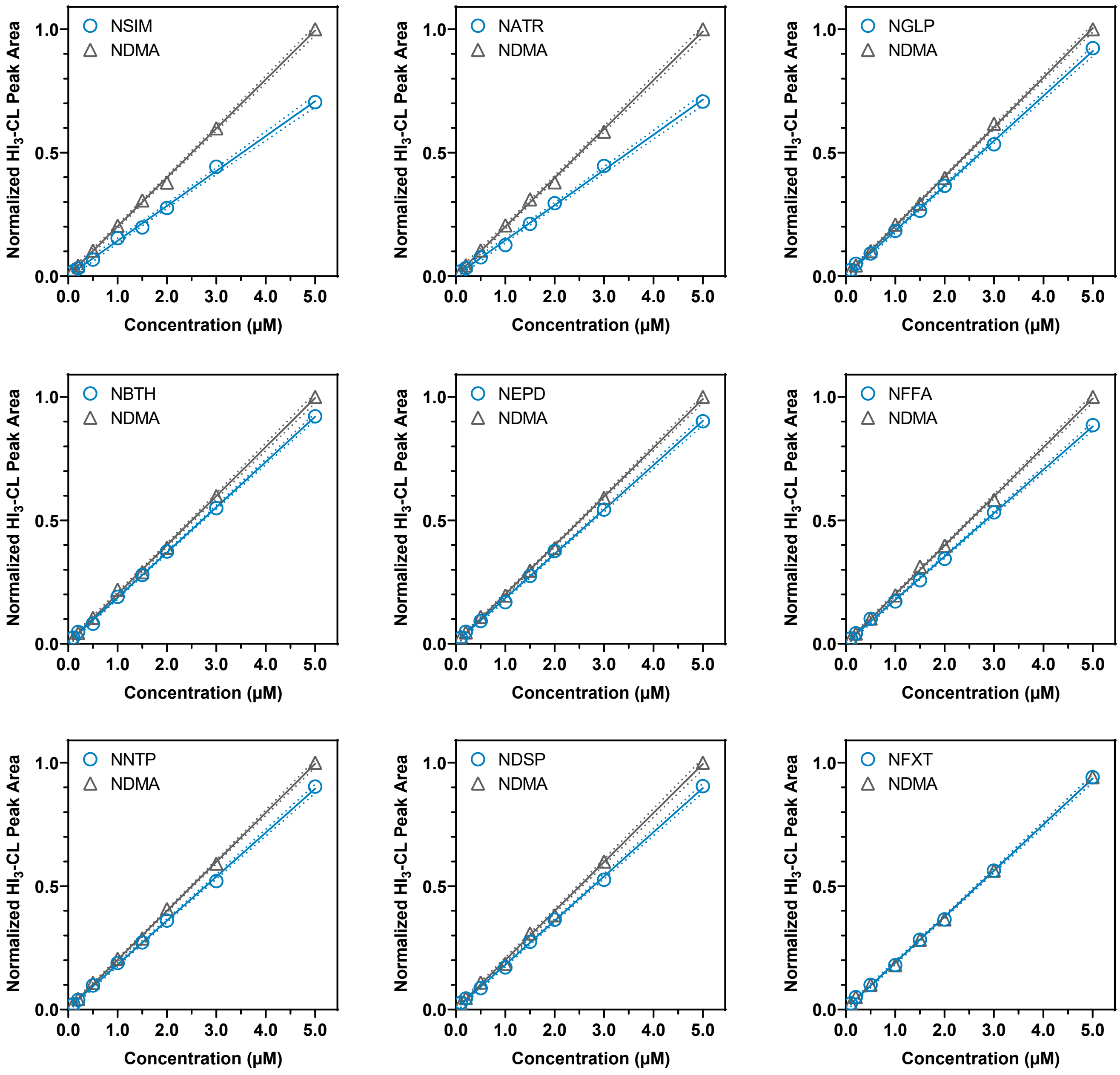

Figure S5f. Calibration curves of *N*-nitrososimazine (NSIM), *N*-nitrosoatrazine (NATR), *N*-nitrosoglyphosate (NGLP), *N*-nitrosobetahistine (NBTH), *N*-nitrosoephedrine (NEPD), *N*-nitrosofenfluramine (NFFA), *N*-nitrosonortriptyline (NNTP), *N*-nitrosodesipramine (NDSP), or *N*-nitrosofluoxetine (NFXT) versus *N*-nitrosodimethylamine (NDMA) measured by the acidic triiodide-chemiluminescence (HI_3-CL) method. On each plot, the grey solid line represents the slope derived from the linear least squares regression analysis of normalized chemiluminescence peak areas versus NDMA concentrations (0.1, 0.2, 0.5, 1.0, 1.5, 2.0, 3.0, and 5.0 μM). The grey dotted lines bracket the 95% confidence interval for the slope. The blue solid line represents the slope derived from the linear least squares regression analysis of normalized chemiluminescence peak areas versus *N*-nitrosamine concentrations (0.1, 0.2, 0.5, 1.0, 1.5, 2.0, 3.0, and 5.0 μM). The blue dotted lines bracket the 95% confidence interval for the slope.

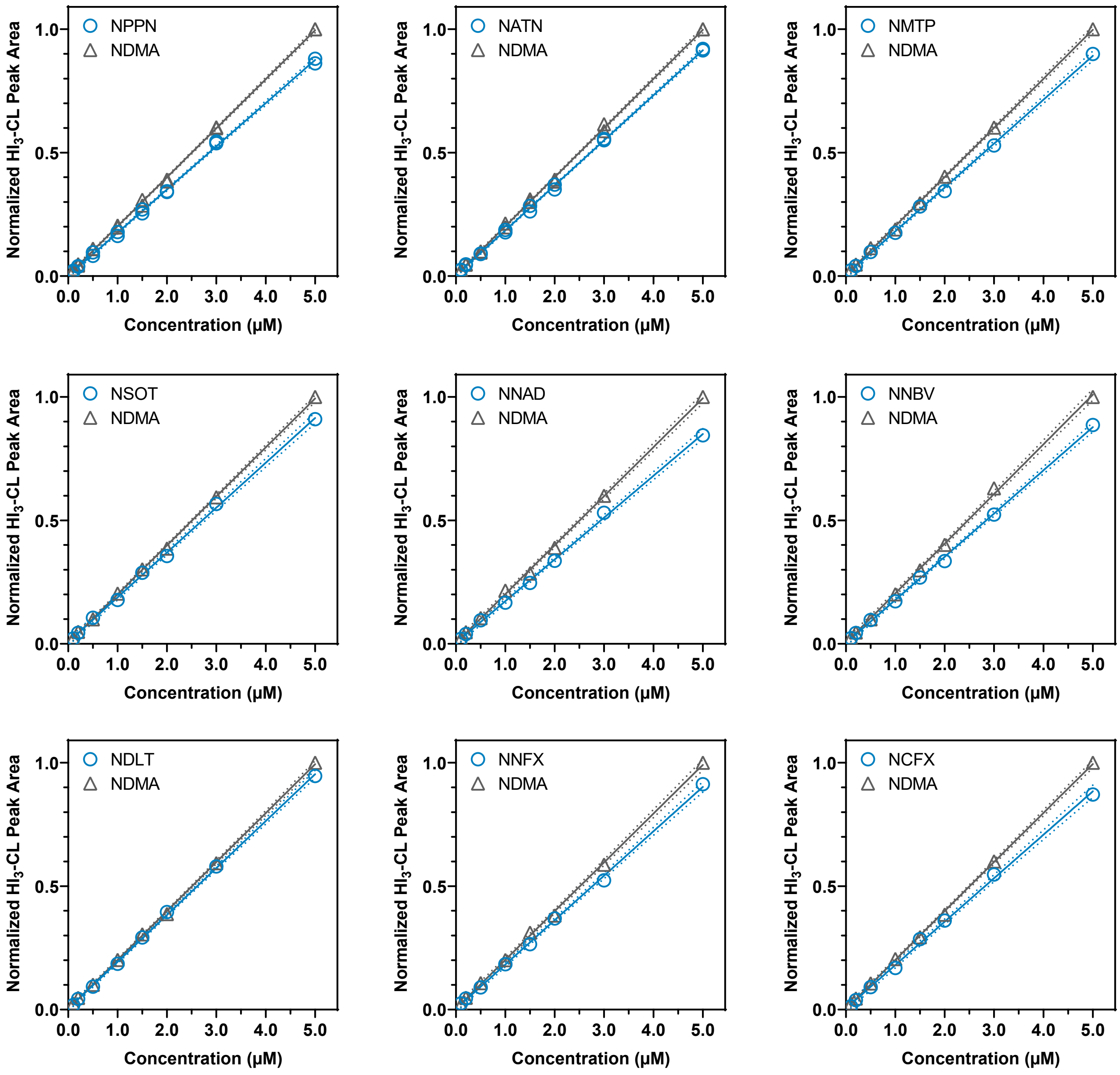

Figure S5g. Calibration curves of *N*-nitrosopropranolol (NPPN), *N*-nitrosoatenolol (NATN), *N*-nitrosometoprolol (NMTP), *N*-nitrososotalol (NSOT), *N*-nitrosonadolol (NNAD), *N*-nitrosonebivolol (NNBV), *N*-nitrosodesloratadine (NDLT), *N*-nitrosonorfloxacin (NNFX), or *N*-nitrosociprofloxacin (NCFX) versus *N*-nitrosodimethylamine (NDMA) measured by the acidic triiodide-chemiluminescence (HI_3-CL) method. On each plot, the grey solid line represents the slope derived from the linear least squares regression analysis of normalized chemiluminescence peak areas versus NDMA concentrations (0.1, 0.2, 0.5, 1.0, 1.5, 2.0, 3.0, and 5.0 μM). The grey dotted lines bracket the 95% confidence interval for the slope. The blue solid line represents the slope derived from the linear least squares regression analysis of normalized chemiluminescence peak areas versus *N*-nitrosamine concentrations (0.1, 0.2, 0.5, 1.0, 1.5, 2.0, 3.0, and 5.0 μM). The blue dotted lines bracket the 95% confidence interval for the slope.

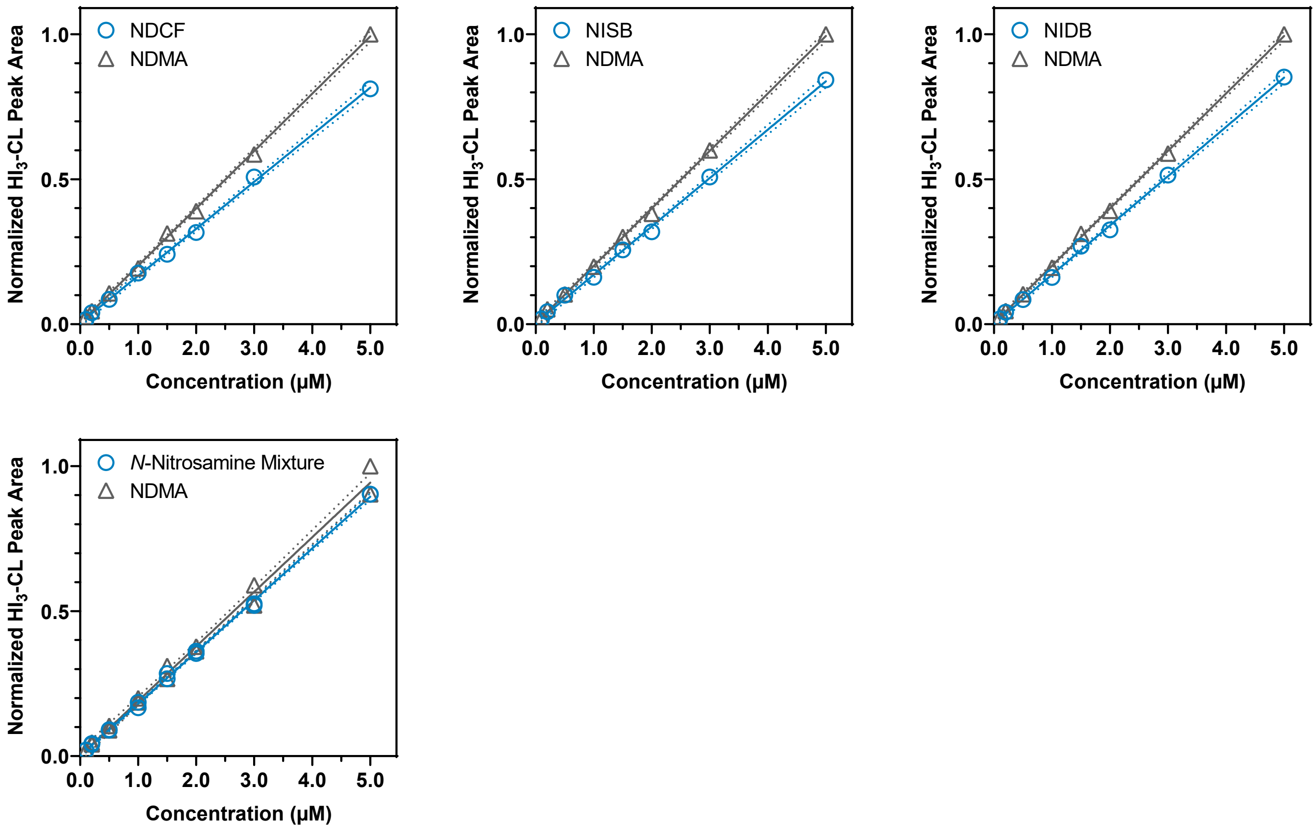

Figure S5h. Calibration curves of *N*-nitrosodiclofenac (NDCF), *N*-nitrosoiminostilbene (NISB), *N*-nitrosoiminodibenzyl (NIDB), or the equal-molar mixture of *N*-nitrosamines versus *N*-nitrosodimethylamine (NDMA) measured by the acidic triiodide-chemiluminescence (HI_3-CL) method. On each plot, the grey solid line represents the slope derived from the linear least squares regression analysis of normalized chemiluminescence peak areas versus NDMA concentrations (0.1, 0.2, 0.5, 1.0, 1.5, 2.0, 3.0, and 5.0 μM). The grey dotted lines bracket the 95% confidence interval for the slope. The blue solid line represents the slope derived from the linear least squares regression analysis of normalized chemiluminescence peak areas versus *N*-nitrosamine concentrations (0.1, 0.2, 0.5, 1.0, 1.5, 2.0, 3.0, and 5.0 μM). The blue dotted lines bracket the 95% confidence interval for the slope.

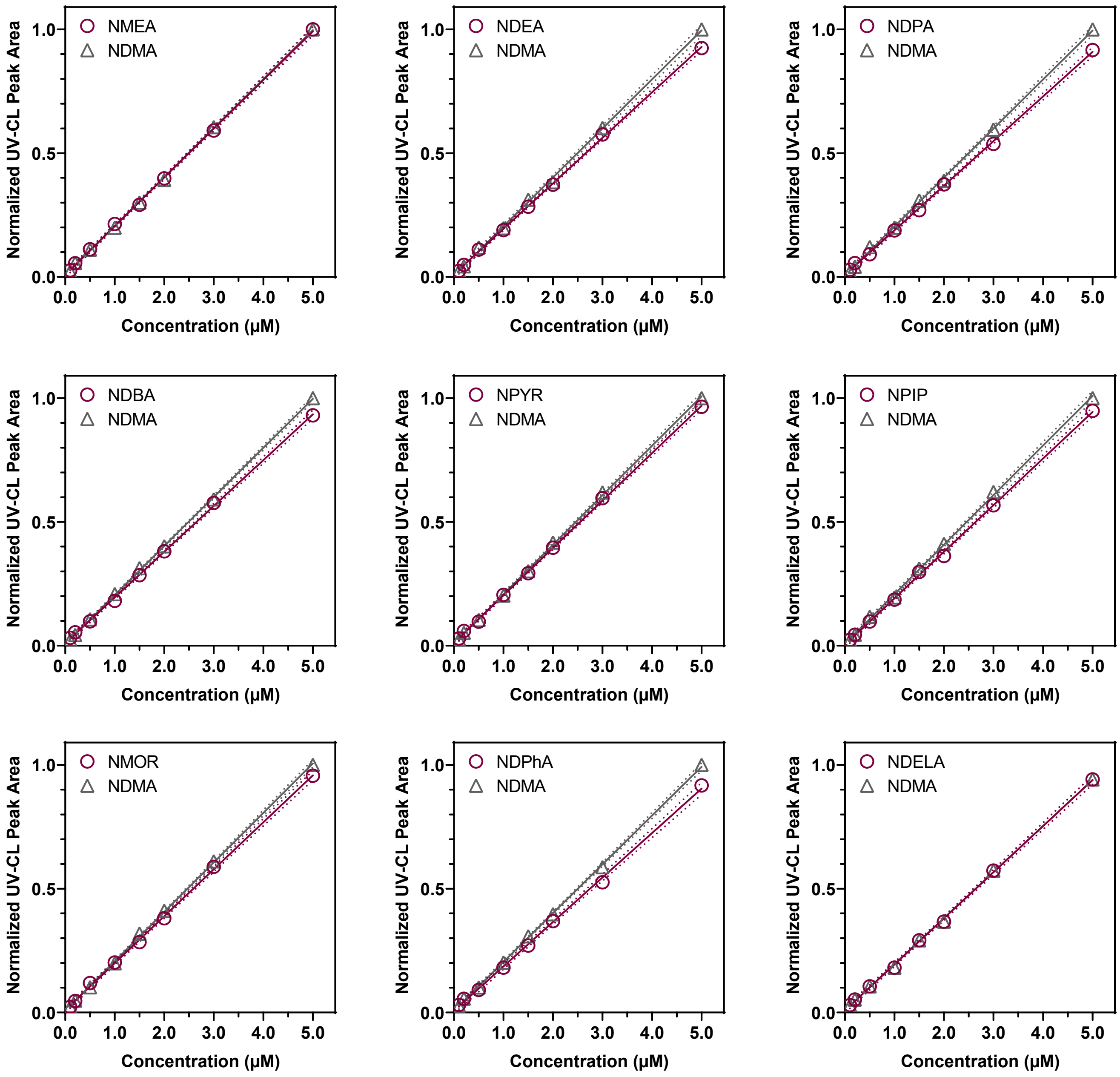

Figure S6a. Calibration curves of *N*-nitrosomethylethylamine (NMEA), *N*-nitrosodiethylamine (NDEA), *N*-nitrosodipropylamine (NDPA), *N*-nitrosodibutylamine (NDBA), *N*-nitrosopyrrolidine (NPYR), *N*-nitrosopiperidine (NPIP), *N*-nitrosomorpholine (NMOR), *N*-nitrosodiphenylamine (NDPhA), or *N*-nitrosodiethanolamine (NDELA) versus *N*-nitrosodimethylamine (NDMA) measured by the UV photolysis-chemiluminescence (UV-CL) method. On each plot, the grey solid line represents the slope derived from the linear least squares regression analysis of normalized chemiluminescence peak areas versus NDMA concentrations (0.1, 0.2, 0.5, 1.0, 1.5, 2.0, 3.0, and 5.0 μM). The grey dotted lines bracket the 95% confidence interval for the slope. The red solid line represents the slope derived from the linear least squares regression analysis of normalized chemiluminescence peak areas versus *N*-nitroso compound concentrations (0.1, 0.2, 0.5, 1.0, 1.5, 2.0, 3.0, and 5.0 μM). The red dotted lines bracket the 95% confidence interval for the slope.

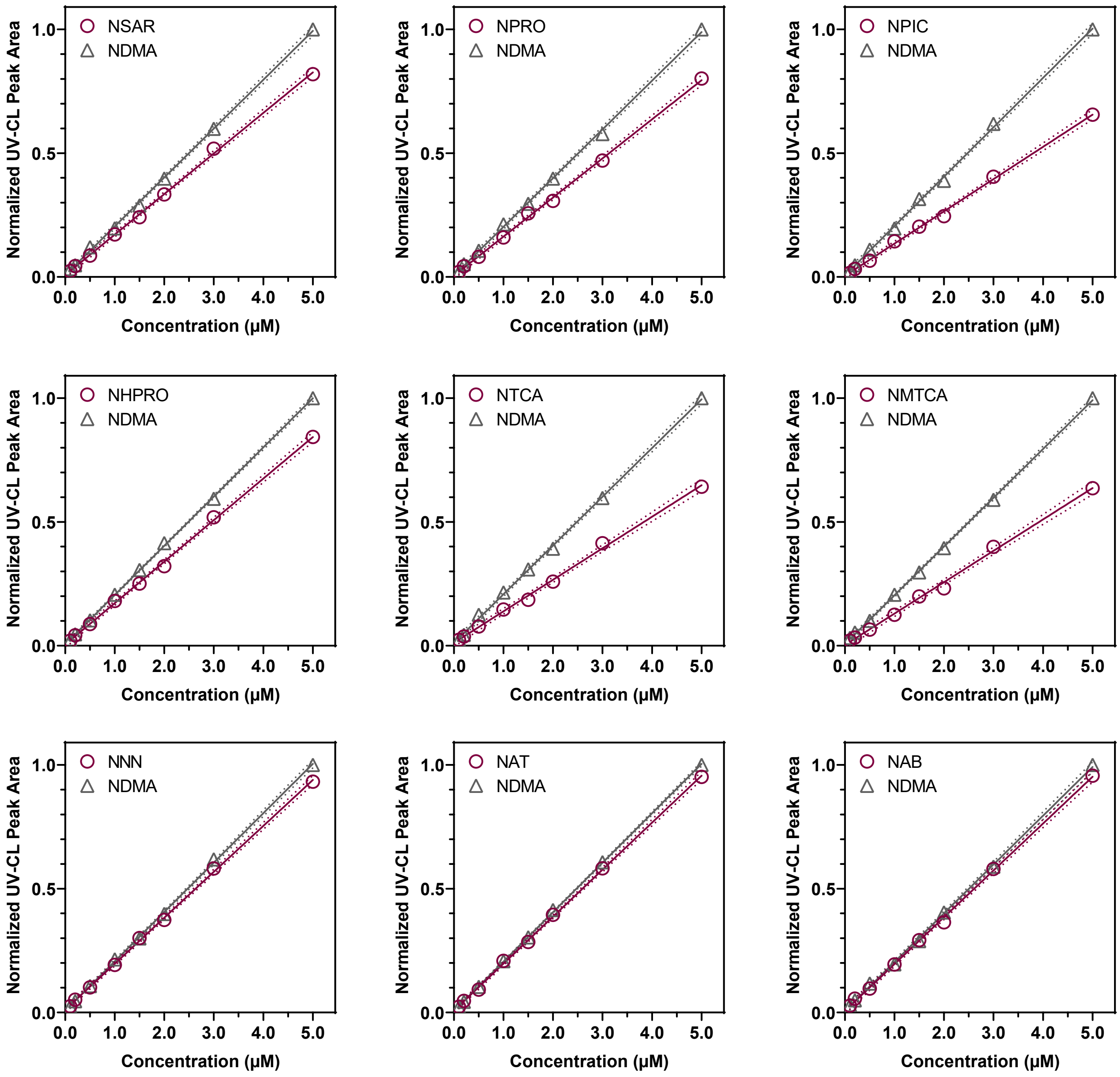

Figure S6b. Calibration curves of *N*-nitrososarcosine (NSAR), *N*-nitrosoproline (NPRO), *N*-nitrosopipecolic acid (NPIC), *N*-nitrosohydroxyproline (NHPRO), *N*-nitrosothiazolidine-4-carboxylic acid (NTCA), *N*-nitroso-2-methylthiazolidine 4-carboxylic acid (NMTCA), *N*-nitrosonornicotine (NNN), *N*-nitrosoanatabine (NAT), or *N*-nitrosoanabasine (NAB) versus *N*-nitrosodimethylamine (NDMA) measured by the UV photolysis-chemiluminescence (UV-CL) method. On each plot, the grey solid line represents the slope derived from the linear least squares regression analysis of normalized chemiluminescence peak areas versus NDMA concentrations (0.1, 0.2, 0.5, 1.0, 1.5, 2.0, 3.0, and 5.0 μM). The grey dotted lines bracket the 95% confidence interval for the slope. The red solid line represents the slope derived from the linear least squares regression analysis of normalized chemiluminescence peak areas versus *N*-nitroso compound concentrations (0.1, 0.2, 0.5, 1.0, 1.5, 2.0, 3.0, and 5.0 μM). The red dotted lines bracket the 95% confidence interval for the slope.

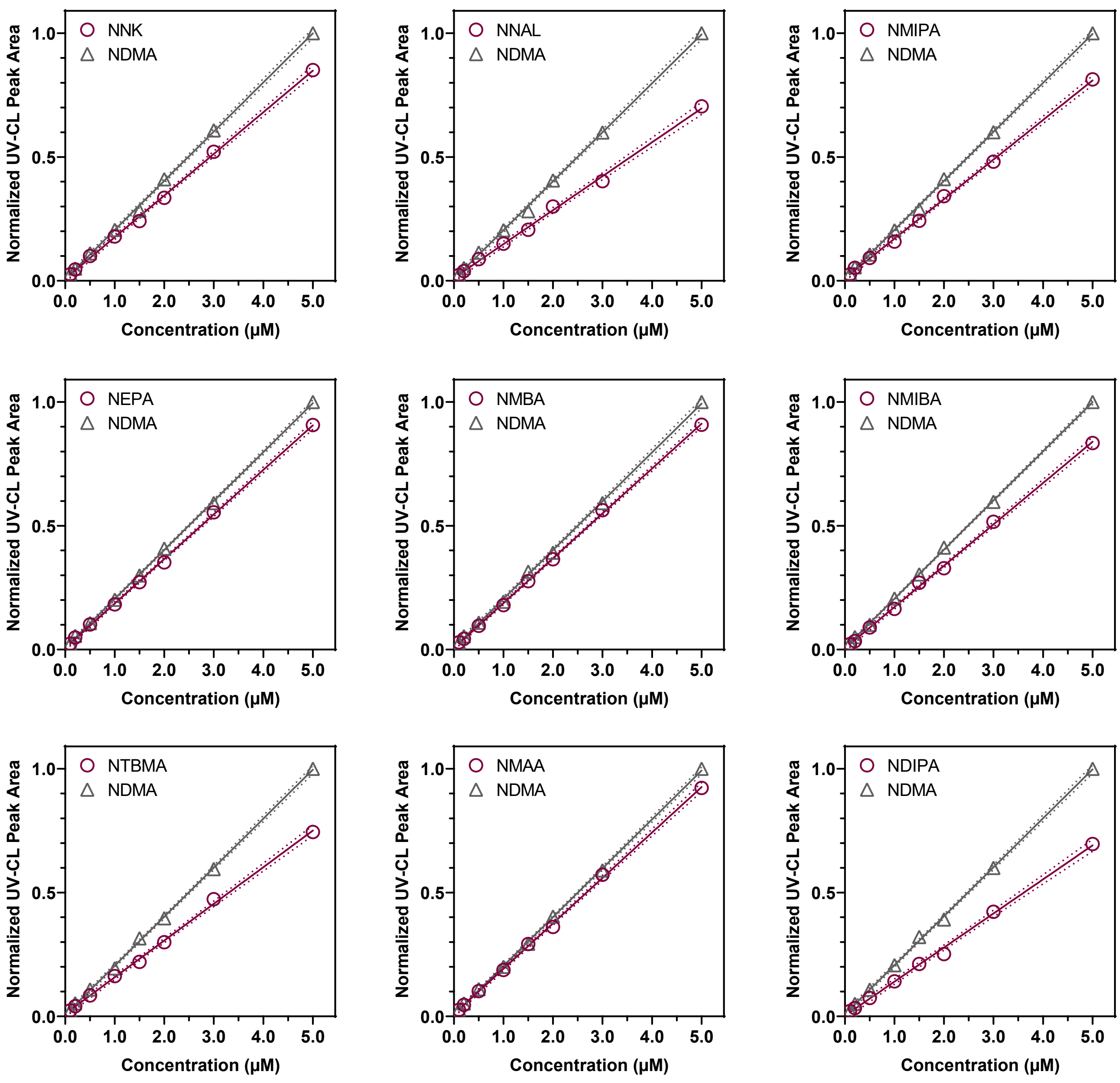

Figure S6c. Calibration curves of 4-(methylnitrosamino)-1-(3-pyridyl)-1-butanone (NNK), 4-(methylnitrosamino)-1-(3-pyridyl)-1-butanol (NNAL), *N*-nitrosomethylisopropylamine (NMIPA), *N*-nitrosoethylpropylamine (NEPA), *N*-nitrosomethylbutylamine (NMBA), *N*-nitrosomethylisobutylamine (NMIBA), *N*-nitroso-*tert*-butylmethylamine (NTBMA), *N*-nitrosomethylamylamine (NMAA), or *N*-nitrosodiisopropylamine (NDIPA) versus *N*-nitrosodimethylamine (NDMA) measured by the UV photolysis-chemiluminescence (UV-CL) method. On each plot, the grey solid line represents the slope derived from the linear least squares regression analysis of normalized chemiluminescence peak areas versus NDMA concentrations (0.1, 0.2, 0.5, 1.0, 1.5, 2.0, 3.0, and 5.0 μM). The grey dotted lines bracket the 95% confidence interval for the slope. The red solid line represents the slope derived from the linear least squares regression analysis of normalized chemiluminescence peak areas versus *N*-nitroso compound concentrations (0.1, 0.2, 0.5, 1.0, 1.5, 2.0, 3.0, and 5.0 μM). The red dotted lines bracket the 95% confidence interval for the slope.

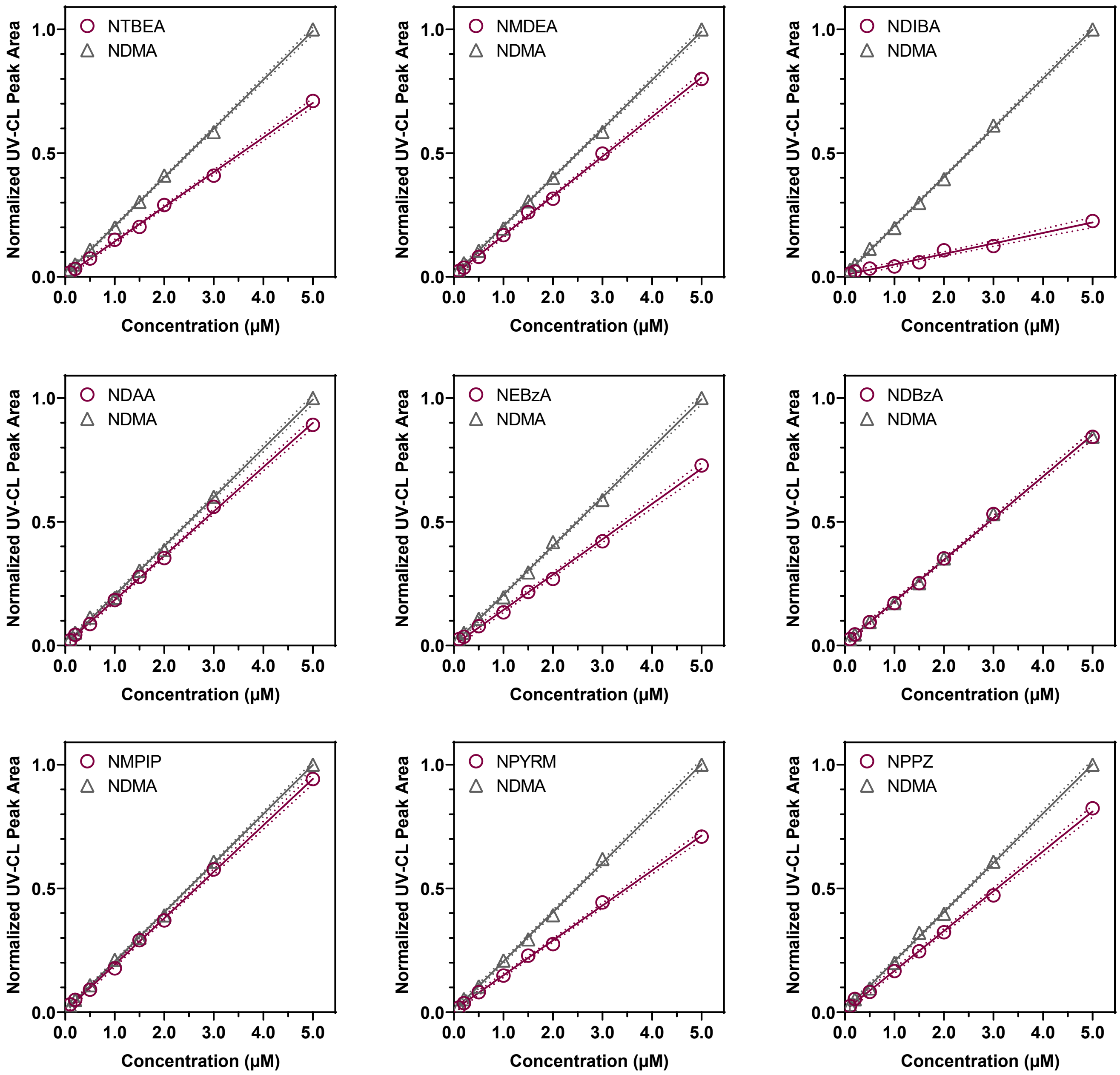

Figure S6d. Calibration curves of *N*-nitroso-*tert*-butylethylamine (NTBEA), *N*-nitrosomethyl-*N,N*-dimethylethylamine (NMDEA), *N*-nitrosodiisobutylamine (NDIBA), *N*-nitrosodiamylamine (NDAA), *N*-nitrosoethylbenzylamine (NEBzA), *N*-nitrosodibenzylamine (NDBzA), 1-nitroso-4-methylpiperidine (NMPIP), *N*-nitroso-2-pyrrolidinmethanol (NPYRM), or *N*-nitrosopiperazine (NPPZ) versus *N*-nitrosodimethylamine (NDMA) measured by the UV photolysis-chemiluminescence (UV-CL) method. On each plot, the grey solid line represents the slope derived from the linear least squares regression analysis of normalized chemiluminescence peak areas versus NDMA concentrations (0.1, 0.2, 0.5, 1.0, 1.5, 2.0, 3.0, and 5.0 μM). The grey dotted lines bracket the 95% confidence interval for the slope. The red solid line represents the slope derived from the linear least squares regression analysis of normalized chemiluminescence peak areas versus *N*-nitroso compound concentrations (0.1, 0.2, 0.5, 1.0, 1.5, 2.0, 3.0, and 5.0 μM). The red dotted lines bracket the 95% confidence interval for the slope.

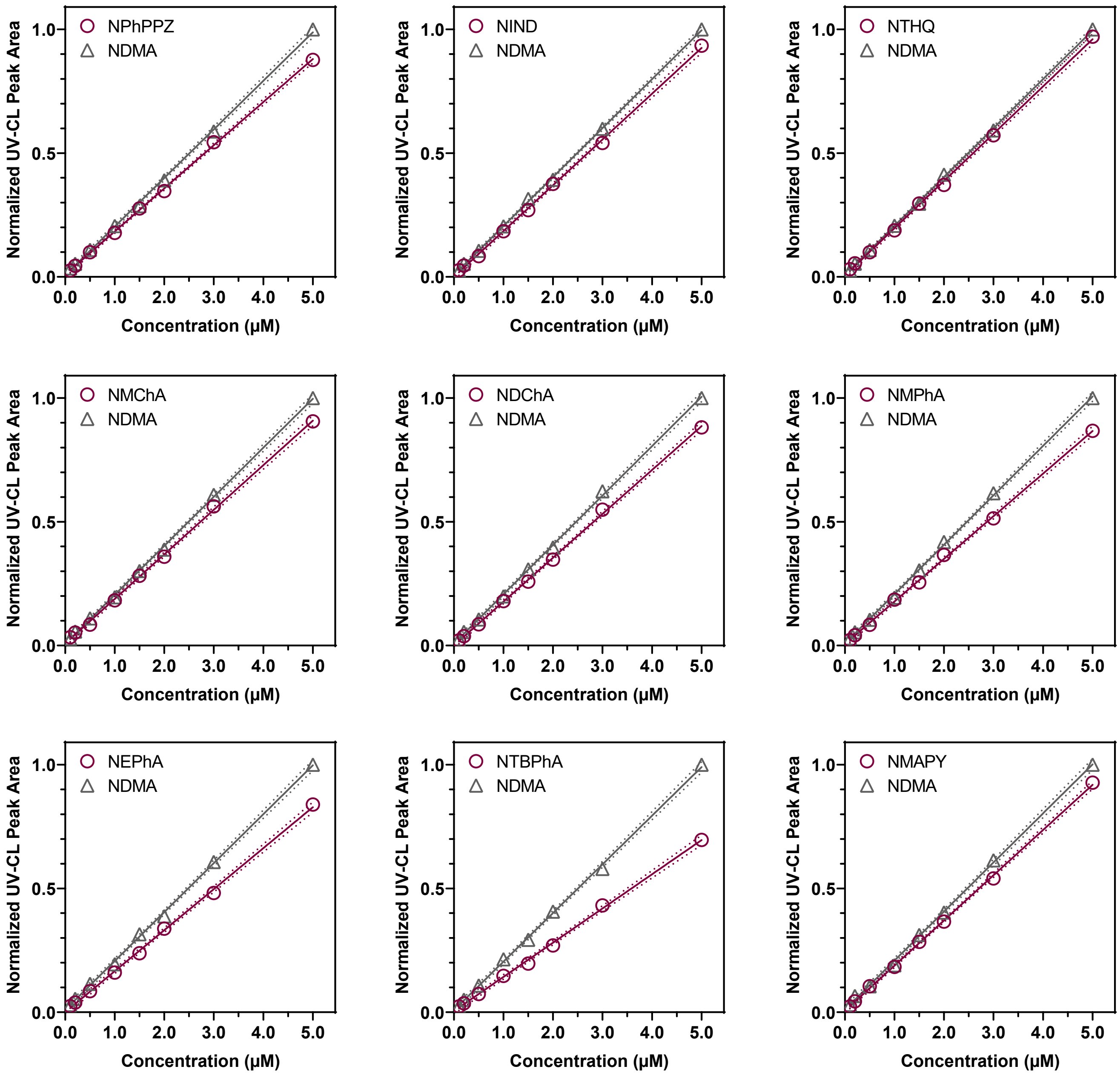

Figure S6e. Calibration curves of 1-nitroso-4-phenylpiperazine (NPhPPZ), *N*-nitrosoindoline (NIND), 1-nitroso-1,2,3,4-tetrahydroquinoline (NTHQ), *N*-nitrosomethylcyclohexylamine (NMChA), *N*-nitrosodicyclohexylamine (NDChA), *N*-nitrosomethylphenylamine (NMPhA), *N*-nitrosoethylphenylamine (NEPhA), *N*-nitroso-*tert*-butylphenylamine (NTBPhA), or *N*-nitroso-4-methylaminopyridine (NMAPY) versus *N*-nitrosodimethylamine (NDMA) measured by the UV photolysis-chemiluminescence (UV-CL) method. On each plot, the grey solid line represents the slope derived from the linear least squares regression analysis of normalized chemiluminescence peak areas versus NDMA concentrations (0.1, 0.2, 0.5, 1.0, 1.5, 2.0, 3.0, and 5.0 μM). The grey dotted lines bracket the 95% confidence interval for the slope. The red solid line represents the slope derived from the linear least squares regression analysis of normalized chemiluminescence peak areas versus *N*-nitroso compound concentrations (0.1, 0.2, 0.5, 1.0, 1.5, 2.0, 3.0, and 5.0 μM). The red dotted lines bracket the 95% confidence interval for the slope.

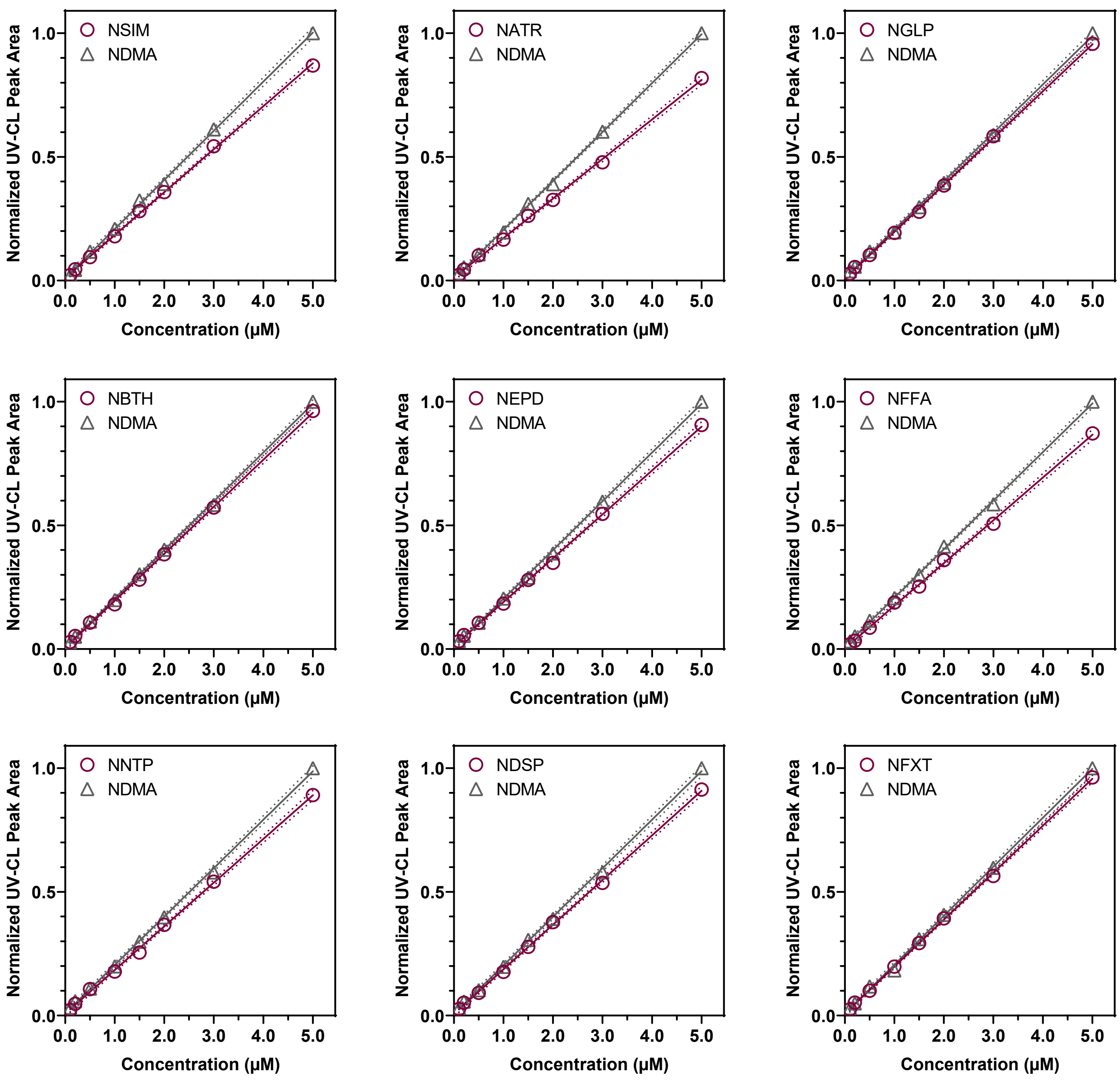

Figure S6f. Calibration curves of *N*-nitrososimazine (NSIM), *N*-nitrosoatrazine (NATR), *N*-nitrosoglyphosate (NGLP), *N*-nitrosobetahistine (NBTH), *N*-nitrosoephedrine (NEPD), *N*-nitrosofenfluramine (NFFA), *N*-nitrosonortriptyline (NNTP), *N*-nitrosodesipramine (NDSP), or *N*-nitrosofluoxetine (NFXT) versus *N*-nitrosodimethylamine (NDMA) measured by the UV photolysis-chemiluminescence (UV-CL) method. On each plot, the grey solid line represents the slope derived from the linear least squares regression analysis of normalized chemiluminescence peak areas versus NDMA concentrations (0.1, 0.2, 0.5, 1.0, 1.5, 2.0, 3.0, and 5.0 μM). The grey dotted lines bracket the 95% confidence interval for the slope. The red solid line represents the slope derived from the linear least squares regression analysis of normalized chemiluminescence peak areas versus *N*-nitrosamine concentrations (0.1, 0.2, 0.5, 1.0, 1.5, 2.0, 3.0, and 5.0 μM). The red dotted lines bracket the 95% confidence interval for the slope.

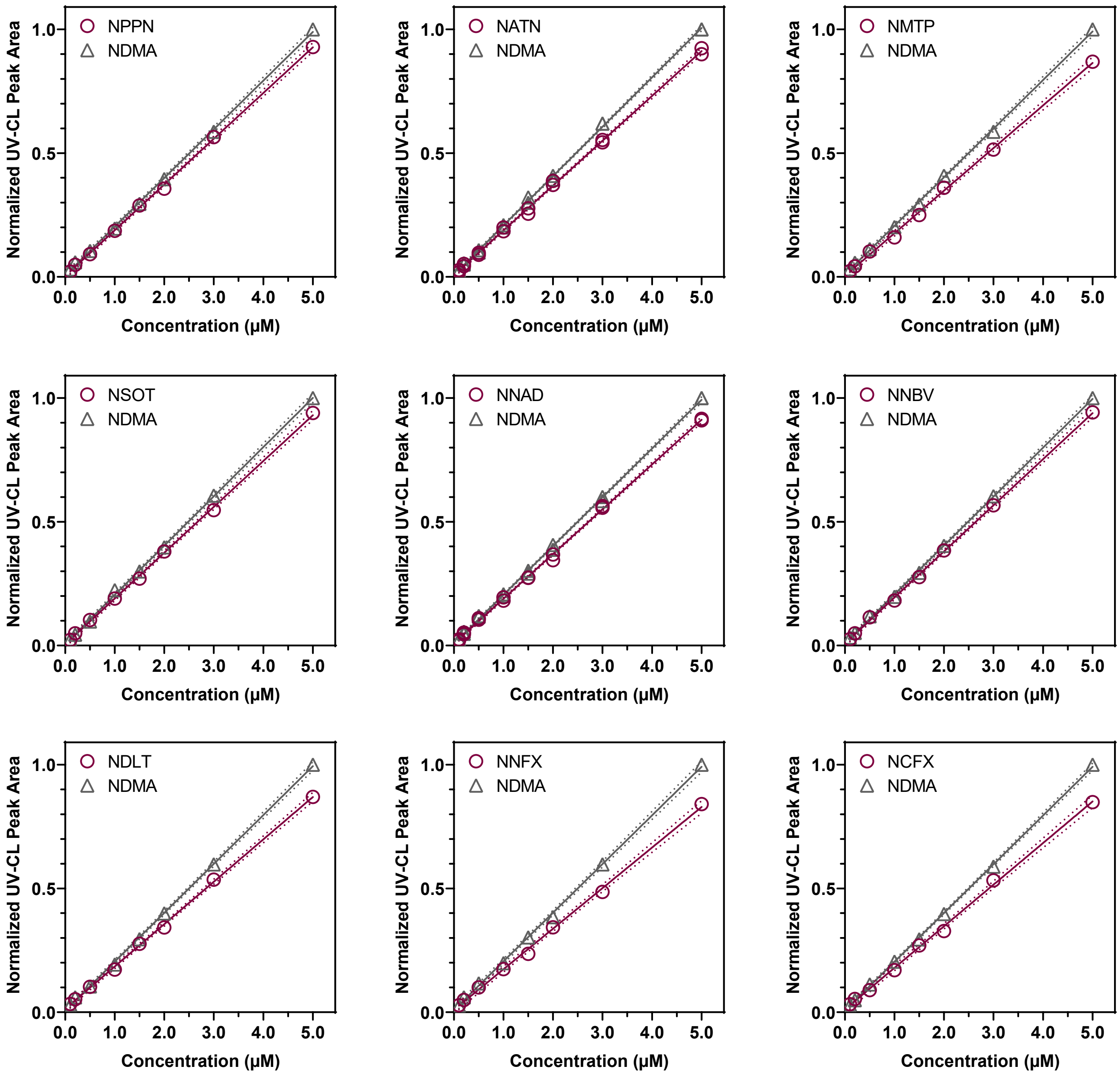

Figure S6g. Calibration curves of *N*-nitrosopropranolol (NPPN), *N*-nitrosoatenolol (NATN), *N*-nitrosometoprolol (NMTP), *N*-nitrososotalol (NSOT), *N*-nitrosonadolol (NNAD), *N*-nitrosonebivolol (NNBV), *N*-nitrosodesloratadine (NDLT), *N*-nitrosonorfloxacin (NNFX), or *N*-nitrosociprofloxacin (NCFX) versus *N*-nitrosodimethylamine (NDMA) measured by the UV photolysis-chemiluminescence (UV-CL) method. On each plot, the grey solid line represents the slope derived from the linear least squares regression analysis of normalized chemiluminescence peak areas versus NDMA concentrations (0.1, 0.2, 0.5, 1.0, 1.5, 2.0, 3.0, and 5.0 μM). The grey dotted lines bracket the 95% confidence interval for the slope. The red solid line represents the slope derived from the linear least squares regression analysis of normalized chemiluminescence peak areas versus *N*-nitrosamine concentrations (0.1, 0.2, 0.5, 1.0, 1.5, 2.0, 3.0, and 5.0 μM). The red dotted lines bracket the 95% confidence interval for the slope.

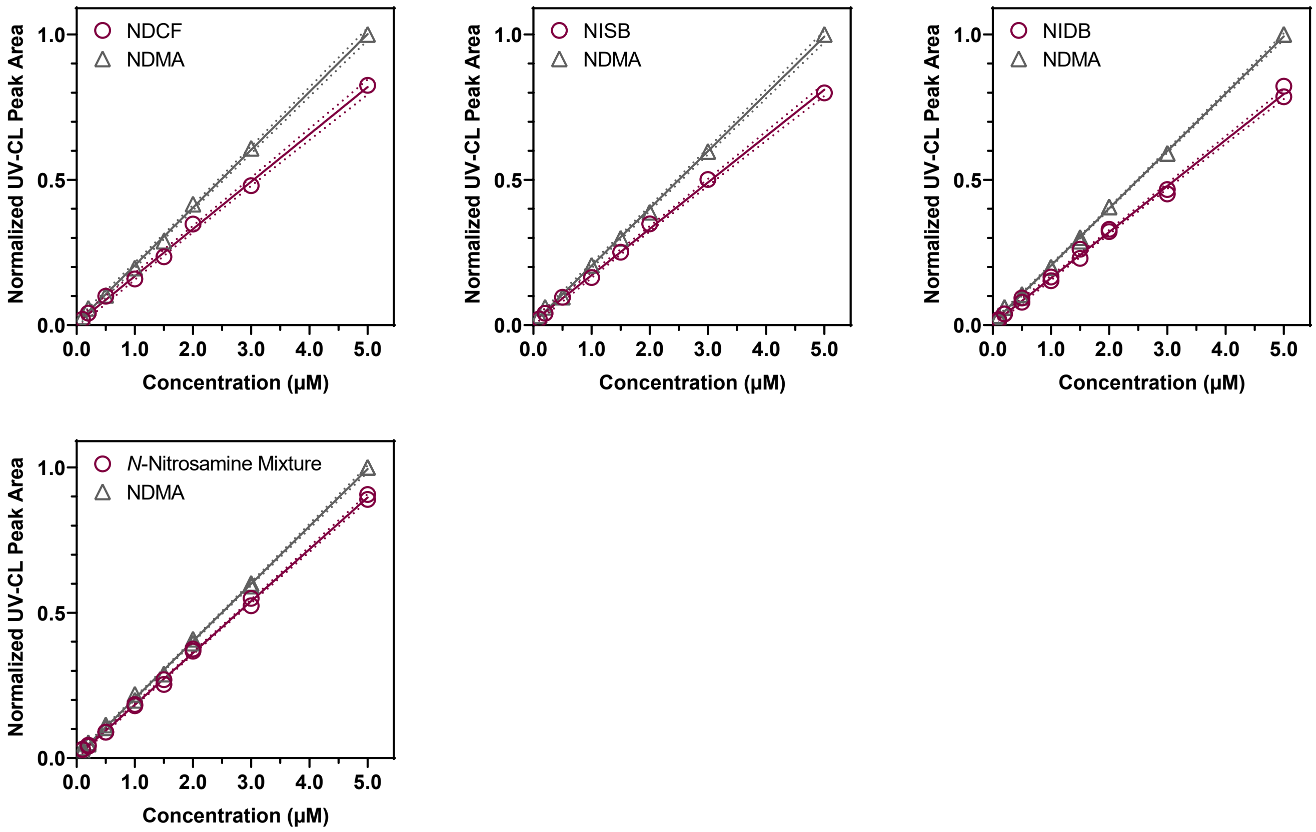

Figure S6h. Calibration curves of *N*-nitrosodiclofenac (NDCF), *N*-nitrosoiminostilbene (NISB), *N*-nitrosoiminodibenzyl (NIDB), or the equal-molar mixture of *N*-nitrosamines versus *N*-nitrosodimethylamine (NDMA) measured by the UV photolysis-chemiluminescence (UV-CL) method. On each plot, the grey solid line represents the slope derived from the linear least squares regression analysis of normalized chemiluminescence peak areas versus NDMA concentrations (0.1, 0.2, 0.5, 1.0, 1.5, 2.0, 3.0, and 5.0 μM). The grey dotted lines bracket the 95% confidence interval for the slope. The red solid line represents the slope derived from the linear least squares regression analysis of normalized chemiluminescence peak areas versus *N*-nitrosamine concentrations (0.1, 0.2, 0.5, 1.0, 1.5, 2.0, 3.0, and 5.0 μM). The red dotted lines bracket the 95% confidence interval for the slope.

**Table S5.** Conversion efficiencies of *N*-nitrosamines reported in the literature							
Compound	Conversion Efficiency	R^2	Injection Volume (µL)	LOD_{Mass} (pmol)	LOD (µM)	LOQ (µM)	Reference
NDMA	100(±0)%	-	100	11	0.11	-	Kulshrestha and Mitch[4] Method: HI_3-CL
NMOR	96(±2)%	-	100	-	-	-	
NDBA	101(±2)%	-	100	-	-	-	
NDPhA	90(±2)%	-	100	-	-	-	
NDELA	75(±1)%	-	100	-	-	-	
EPA 521 Mix	103(±3)%	-	100	-	-	-	
NDMA	100(±4)%	0.99	200	14	0.07	0.21	Breider and von Gunten[5] Method: UV-CL
NMEA	102(±6)%	0.97	200	14	0.07	0.22	
NDEA	83(±4)%	0.99	200	16	0.08	0.24	
NDPA	79(±5)%	0.99	200	14	0.07	0.21	
NDBA	83(±6)%	0.99	200	14	0.07	0.20	
NDELA	70(±5)%	0.99	200	26	0.13	0.40	
NDPhA	92(±5)%	0.98	200	20	0.10	0.31	
NPIP	88(±6)%	0.99	200	18	0.09	0.27	
NPYR	86(±6)%	0.98	200	20	0.10	0.30	
NMOR	68(±5)%	0.98	200	24	0.12	0.36	
EPA 8270 Mix	87(±5)%	0.99	200	20	0.10	0.30	

LOD_{Mass} = Limit of detection (mass-based). LOD = Limit of detection. LOQ = Limit of quantification. EPA 521 Mix = Equal-molar mixture of *N*-nitrosodimethylamine, *N*-nitrosomethylethylamine, *N*-nitrosodiethylamine, *N*-nitrosodipropylamine, *N*-nitrosodibutylamine, *N*-nitrosopyrrolidine, and *N*-nitrosopiperidine. EPA 8270 Mix = Equal-molar mixture of *N*-nitrosodimethylamine, *N*-nitrosomethylethylamine, *N*-nitrosodiethylamine, *N*-nitrosodipropylamine, *N*-nitrosodibutylamine, *N*-nitrosopyrrolidine, *N*-nitrosopiperidine, *N*-nitrosomorpholine, and *N*-nitrosodiphenylamine. Note that the conversion efficiencies originally reported in Breider and von Gunten were recalculated with reference to NDMA for comparison with those measured herein.

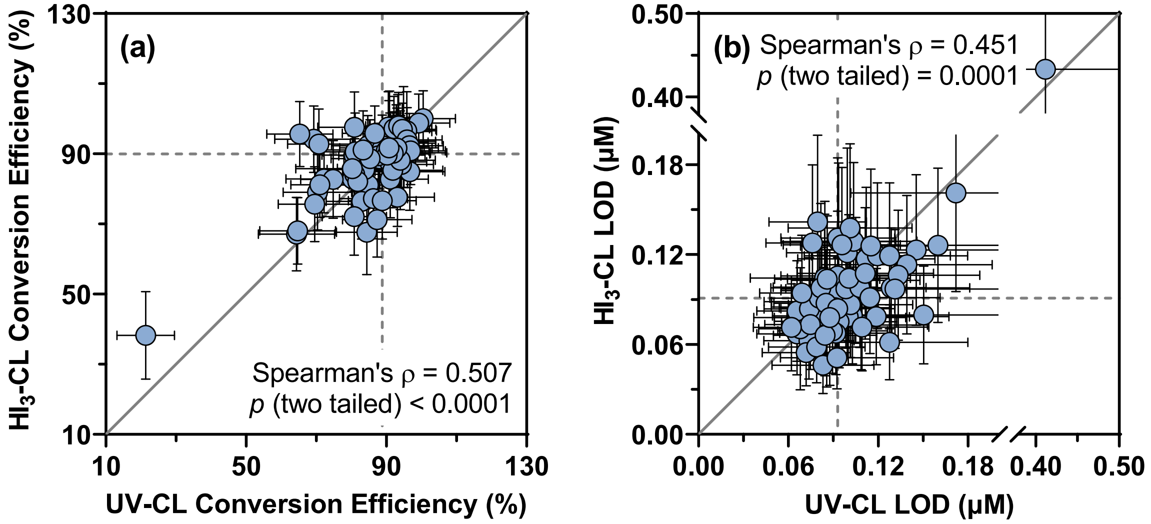

Figure S7. Correlations between the conversion efficiencies and limits of detection (LODs) of *N*-nitrosamines measured by the acidic triiodide-chemiluminescence (HI_3-CL) and UV photolysis-chemiluminescence (UV-CL) methods: **(a)** Cross plot of the conversion efficiencies of *N*-nitrosamines measured by the HI_3-CL method in Table S3 and those measured by the UV-CL method in Table S4. Error bars represent the standard deviations of *N*-nitrosamine conversion efficiencies; where absent, bars fall within symbols. The grey dashed lines mark the median conversion efficiencies of *N*-nitrosamines measured by the HI_3-CL and UV-CL methods, respectively. **(b)** Cross plot of the LODs of *N*-nitrosamines measured by the HI_3-CL method in Table S3 and those measured by the UV-CL method in Table S4 based on an injection volume of 100 μL. Error bars represent the standard deviations of *N*-nitrosamine LODs; where absent, bars fall within symbols. The grey dashed lines mark the median LODs of *N*-nitrosamines measured by the HI_3-CL and UV-CL methods, respectively.

4. Chemical and photolytic denitrosation mechanisms of *N*-nitrosamines

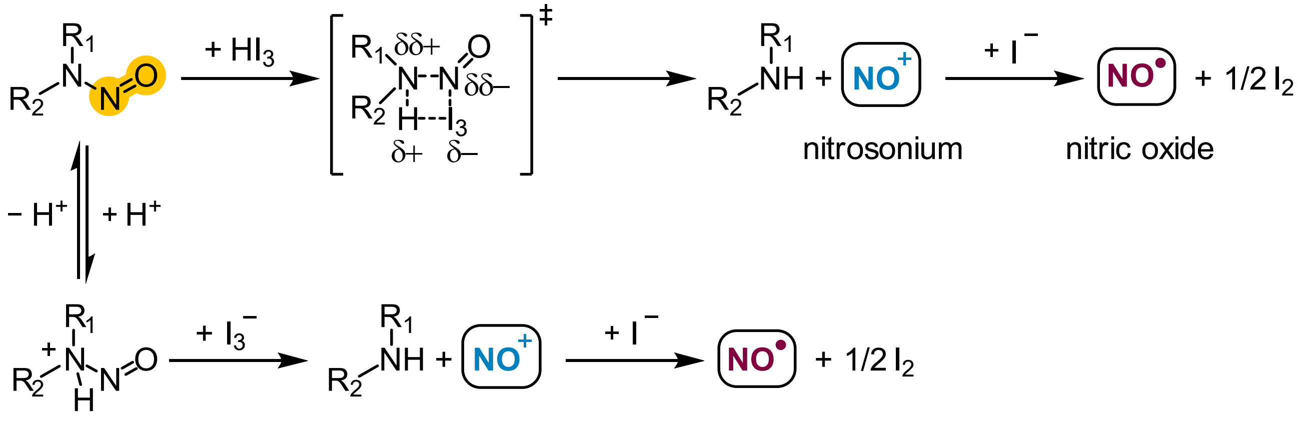

Figure S8. Chemical denitrosation mechanisms proposed for *N*-nitrosamines with acidic triiodide treatment adapted from Baliga *et al.* (1970),[7] Keefer *et al.* (1988),[8] Feelisch *et al.* (2002),[9] and Beard and Swager (2021).[10]

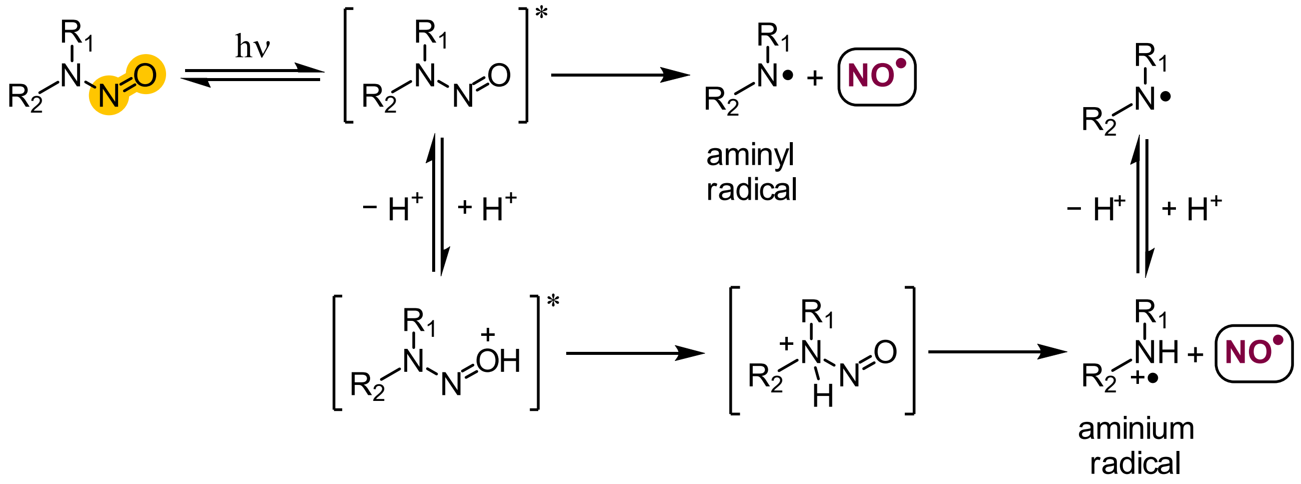

Figure S9. Photolytic denitrosation mechanisms proposed for *N*-nitrosamines with UV photolysis adapted from Polo and Chow (1976),[11] Stefan and Bolton (2002),[12] and Beard and Swager (2021).[10]

**Table S6.** Homolytic bond dissociation energies of the N-NO bond in *N*-nitrosamines				
Compound	Acronym	BDE (ALFABET) (kcal/mol)	BDE (BonDNet) (kcal/mol)	Category
N-Nitrosodimethylamine	NDMA	46.5	46.6	dialkyl
N-Nitrosomethylethylamine	NMEA	45.5	47.5	dialkyl
N-Nitrosodiethylamine	NDEA	44.8	48.0	dialkyl
N-Nitrosodipropylamine	NDPA	44.6	48.0	dialkyl
N-Nitrosodibutylamine	NDBA	44.9	48.0	dialkyl
N-Nitrosopyrrolidine	NPYR	45.1	43.1	cyclic
N-Nitrosopiperidine	NPIP	48.1	48.2	cyclic
N-Nitrosomorpholine	NMOR	46.3	48.0	heterocyclic
N-Nitrosodiphenylamine	NDPhA	30.2	39.9	diaryl
N-Nitrosodiethanolamine	NDELA	41.8	47.5	dialkyl
N-Nitrososarcosine	NSAR	45.5	46.6	dialkyl
N-Nitrosoproline	NPRO	42.9	46.6	cyclic
N-Nitrosopipecolic Acid	NPIC	46.0	47.3	cyclic
N-Nitrosohydroxyproline	NHPRO	42.6	46.6	cyclic
N-Nitrosothiazolidine-4-Carboxylic Acid	NTCA	41.9	45.9	heterocyclic
N-Nitroso-2-Methylthiazolidine 4-Carboxylic Acid	NMTCA	42.7	46.4	heterocyclic
N-Nitrosonornicotine	NNN	45.2	47.5	cyclic
N-Nitrosoanatabine	NAT	45.4	48.7	cyclic
N-Nitrosoanabasine	NAB	46.4	48.7	cyclic
4-(Methylnitrosamino)-1-(3-Pyridyl)-1-Butanone	NNK	45.3	46.8	dialkyl
4-(Methylnitrosamino)-1-(3-Pyridyl)-1-Butanol	NNAL	45.3	47.0	dialkyl
N-Nitrosomethylisopropylamine	NMIPA	46.2	48.2	dialkyl
N-Nitrosoethylpropylamine	NEPA	44.9	48.0	dialkyl
N-Nitrosomethylbutylamine	NMBA	45.3	48.0	dialkyl
N-Nitrosomethylisobutylamine	NMIBA	45.2	47.7	dialkyl
N-Nitroso-*tert*-Butylmethylamine	NTBMA	46.6	48.4	dialkyl
N-Nitrosomethylamylamine	NMAA	45.3	47.7	dialkyl
N-Nitrosodiisopropylamine	NDIPA	44.0	49.1	dialkyl
N-Nitroso-*tert*-Butylethylamine	NTBEA	45.7	48.7	dialkyl
N-Nitrosomethyl-*N,N*-Dimethylethylamine	NMDEA	45.7	47.5	dialkyl
N-Nitrosodiisobutylamine	NDIBA	44.4	48.0	dialkyl
N-Nitrosodiamylamine	NDAA	44.9	48.2	dialkyl
N-Nitrosoethylbenzylamine	NEBzA	45.4	48.0	dialkyl
N-Nitrosodibenzylamine	NDBzA	45.8	48.4	dialkyl
1-Nitroso-4-Methylpiperidine	NMPIP	47.3	48.2	cyclic
N-Nitroso-2-Pyrrolidinmethanol	NPYRM	44.5	46.8	cyclic
N-Nitrosopiperazine	NPPZ	46.8	48.4	heterocyclic
1-Nitroso-4-Phenylpiperazine	NPhPPZ	47.7	48.7	heterocyclic
N-Nitrosoindoline	NIND	39.4	39.7	cyclic alkylaryl

**Table S6.** Homolytic bond dissociation energies of the N-NO bond in *N*-nitrosamines (continued)				
Compound	Acronym	BDE (ALFABET) (kcal/mol)	BDE (BonDNet) (kcal/mol)	Category
1-Nitroso-1,2,3,4-Tetrahydroquinoline	NTHQ	39.0	41.3	cyclic alkylaryl
N-Nitrosomethylcyclohexylamine	NMChA	46.6	48.7	alkylcycloalkyl
N-Nitrosodicyclohexylamine	NDChA	45.6	48.4	dicycloalkyl
N-Nitrosomethylphenylamine	NMPhA	39.3	42.7	alkylaryl
N-Nitrosoethylphenylamine	NEPhA	39.5	43.4	alkylaryl
N-Nitroso-*tert*-Butylphenylamine	NTBPhA	38.6	49.1	alkylaryl
N-Nitroso-4-Methylaminopyridine	NMAPY	40.3	42.4	alkylheteroaryl
N-Nitrososimazine	NSIM	45.5	44.7	alkylheteroaryl
N-Nitrosoatrazine	NATR	45.5	43.6	alkylheteroaryl
N-Nitrosoglyphosate	NGLP	44.0	46.1	dialkyl
N-Nitrosobetahistine	NBTH	45.5	47.5	dialkyl
N-Nitrosoephedrine	NEPD	46.0	48.0	dialkyl
N-Nitrosofenfluramine	NFFA	44.8	48.4	dialkyl
N-Nitrosonortriptyline	NNTP	45.6	49.8	dialkyl
N-Nitrosodesipramine	NDSP	45.8	50.7	dialkyl
N-Nitrosofluoxetine	NFXT	45.3	48.7	dialkyl
N-Nitrosopropranolol	NPPN	45.4	48.7	dialkyl
N-Nitrosoatenolol	NATN	45.4	48.7	dialkyl
N-Nitrosometoprolol	NMTP	45.4	48.9	dialkyl
N-Nitrososotalol	NSOT	45.3	48.7	dialkyl
N-Nitrosonadolol	NNAD	44.6	48.9	dialkyl
N-Nitrosonebivolol	NNBV	42.5	48.9	dialkyl
N-Nitrosodesloratadine	NDLT	46.5	50.5	cyclic
N-Nitrosonorfloxacin	NNFX	47.7	48.9	heterocyclic
N-Nitrosociprofloxacin	NCFX	47.7	49.6	heterocyclic
N-Nitrosodiclofenac	NDCF	26.5	41.0	diaryl
N-Nitrosoiminostilbene	NISB	23.6	40.1	cyclic diaryl
N-Nitrosoiminodibenzyl	NIDB	25.1	40.8	cyclic diaryl

BDE = Bond dissociation energy. ALFABET = Fast, Accurate Bond dissociation Enthalpy Tool.[13] BonDNet = Bond Dissociation Network.[14]

**Table S7.** pK_a's of *N*-nitrosamine precursors				
Compound	*N*-Nitrosamine Precursor	CAS	SMILES	pK_a
NDMA	Dimethylamine	124-40-3	CNC	9.53
NMEA	Methylethylamine	624-78-2	CCNC	9.54
NDEA	Diethylamine	109-89-7	CCNCC	9.58
NDPA	Dipropylamine	142-84-7	CCCNCCC	9.73
NDBA	Dibutylamine	111-92-2	CCCCNCCCC	9.72
NPYR	Pyrrolidine	123-75-1	C1CCNC1	9.42
NPIP	Piperidine	110-89-4	C1CCNCC1	9.43
NMOR	Morpholine	110-91-8	C1COCCN1	7.32
NDPhA	Diphenylamine	122-39-4	C1=CC=C(C=C1)NC2=CC=CC=C2	0.80
NDELA	Diethanolamine	111-42-2	C(CO)NCCO	7.62
NSAR	Sarcosine	107-97-1	CNCC(=O)O	8.87
NPRO	L-Proline	147-85-3	C1C[C@H](NC1)C(=O)O	8.86
NPIC	Piperidine-2-Carboxylic Acid	535-75-1	C1CCNC(C1)C(=O)O	8.91
NHPRO	L-Hydroxyproline	51-35-4	C1[C@H](CN[C@@H]1C(=O)O)O	8.26
NTCA	Thiazolidine-4-Carboxylic Acid	444-27-9	C1C(NCS1)C(=O)O	4.42
NMTCA	2-Methylthiazolidine-4-Carboxylic Acid	4165-32-6	CC1NC(CS1)C(=O)O	2.71
NNN	Nornicotine	5746-86-1	C1CC(NC1)C2=CN=CC=C2	7.67
NAT	Anatabine	2743-90-0	C1C=CCNC1C2=CN=CC=C2	6.91
NAB	Anabasine	13078-04-1	C1CCNC(C1)C2=CN=CC=C2	7.73
NNK	4-(Methylamino)-1-(3-Pyridyl)-1-Butanone	2055-23-4	CNCCCC(=O)C1=CN=CC=C1	8.81
NNAL	4-(Methylamino)-1-(3-Pyridyl)-1-Butanol	76030-54-1	CNCCCC(C1=CN=CC=C1)O	9.29
NMIPA	Methylisopropylamine	4747-21-1	CC(C)NC	9.58
NEPA	Ethylpropylamine	20193-20-8	CCCNCC	9.64
NMBA	Methylbutylamine	110-68-9	CCCCNC	9.55
NMIBA	Methylisobutylamine	625-43-4	CC(C)CNC	9.67
NTBMA	*tert*-Butylmethylamine	14610-37-8	CC(C)(C)NC	9.64
NMAA	Methylamylamine	25419-06-1	CCCCCNC	9.54
NDIPA	Diisopropylamine	108-18-9	CC(C)NC(C)C	9.69
NTBEA	*tert*-Butylethylamine	4432-77-3	CCNC(C)(C)C	9.70
NMDEA	*N,N,N'*-Trimethylethylenediamine	142-25-6	CNCCN(C)C	8.54
NDIBA	Diisobutylamine	110-96-3	CC(C)CNCC(C)C	9.95
NDAA	Diamylamine	2050-92-2	CCCCCNCCCCC	9.72
NEBzA	Ethylbenzylamine	14321-27-8	CCNCC1=CC=CC=C1	8.15
NDBzA	Dibenzylamine	103-49-1	C1=CC=C(C=C1)CNCC2=CC=CC=C2	7.35
NMPIP	4-Methylpiperidine	626-58-4	CC1CCNCC1	9.39
NPYRM	2-Pyrrolidinemethanol	498-63-5	C1CC(NC1)CO	8.40
NPPZ	Piperazine	110-85-0	C1CNCCN1	4.46
NPhPPZ	1-Phenylpiperazine	92-54-6	C1CN(CCN1)C2=CC=CC=C2	7.66
NIND	Indoline	496-15-1	C1CNC2=CC=CC=C21	4.04

**Table S7.** pK_a's of *N*-nitrosamine precursors (continued)				
Compound	*N*-Nitrosamine Precursor	CAS	SMILES	pK_a
NTHQ	1,2,3,4-Tetrahydroquinoline	635-46-1	C1CC2=CC=CC=C2NC1	4.26
NMChA	Methylcyclohexylamine	100-60-7	CNC1CCCCC1	9.68
NDChA	Dicyclohexylamine	101-83-7	C1CCC(CC1)NC2CCCCC2	10.03
NMPhA	Methylphenylamine	100-61-8	CNC1=CC=CC=C1	4.09
NEPhA	Ethylphenylamine	103-69-5	CCNC1=CC=CC=C1	4.29
NTBPhA	*tert*-Butylphenylamine	937-33-7	CC(C)(C)NC1=CC=CC=C1	5.38
NMAPY	4-Methylaminopyridine	1121-58-0	CNC1=CC=NC=C1	-3.37
NSIM	Simazine	122-34-9	CCNC1=NC(=NC(=N1)Cl)NCC	-25.11
NATR	Atrazine	1912-24-9	CCNC1=NC(=NC(=N1)Cl)NC(C)C	-25.12
NGLP	Glyphosate	1071-83-6	C(C(=O)O)NCP(=O)(O)O	5.92
NBTH	Betahistine	5638-76-6	CNCCC1=CC=CC=N1	8.38
NEPD	Ephedrine	299-42-3	C[C@@H]([C@@H](C1=CC=CC=C1)O)NC	8.18
NFFA	Fenfluramine	458-24-2	CCNC(C)CC1=CC(=CC=C1)C(F)(F)F	8.76
NNTP	Nortriptyline	72-69-5	CNCCC=C1C2=CC=CC=C2CCC3=CC=CC=C31	8.98
NDSP	Desipramine	50-47-5	CNCCCN1C2=CC=CC=C2CCC3=CC=CC=C31	8.59
NFXT	Fluoxetine	54910-89-3	CNCCC(C1=CC=CC=C1)OC2=CC=C(C=C2)C(F)(F)F	8.08
NPPN	Propranolol	525-66-6	CC(C)NCC(COC1=CC=CC2=CC=CC=C21)O	7.96
NATN	Atenolol	29122-68-7	CC(C)NCC(COC1=CC=C(C=C1)CC(=O)N)O	7.96
NMTP	Metoprolol	51384-51-1	CC(C)NCC(COC1=CC=C(C=C1)CCOC)O	7.96
NSOT	Sotalol	3930-20-9	CC(C)NCC(C1=CC=C(C=C1)NS(=O)(=O)C)O	8.46
NNAD	Nadolol	42200-33-9	CC(C)(C)NCC(COC1=CC=CC2=C1C[C@@H]([C@@H](C2)O)O)O	8.04
NNBV	Nebivolol	99200-09-6	C1CC2=C(C=CC(=C2)F)OC1C(CNCC(C3CCC4=C(O3)C=CC(=C4)F)O)O	6.64
NDLT	Desloratadine	100643-71-8	C1CC2=C(C=CC(=C2)Cl)C(=C3CCNCC3)C4=C1C=CC=N4	8.69
NNFX	Norfloxacin	70458-96-7	CCN1C=C(C(=O)C2=CC(=C(C=C21)N3CCNCC3)F)C(=O)O	7.47
NCFX	Ciprofloxacin	85721-33-1	C1CC1N2C=C(C(=O)C3=CC(=C(C=C32)N4CCNCC4)F)C(=O)O	7.47
NDCF	Diclofenac	15307-86-5	C1=CC=C(C(=C1)CC(=O)O)NC2=C(C=CC=C2Cl)Cl	-0.27
NISB	Iminostilbene	256-96-2	C1=CC=C2C(=C1)C=CC3=CC=CC=C3N2	0.43
NIDB	Iminodibenzyl	494-19-9	C1CC2=CC=CC=C2NC3=CC=CC=C31	0.52

pK_a predicted at 353.15 K (i.e., the purge vessel was heated at 80 °C) by *MarvinSketch (Version 22.19.0)*.[15]

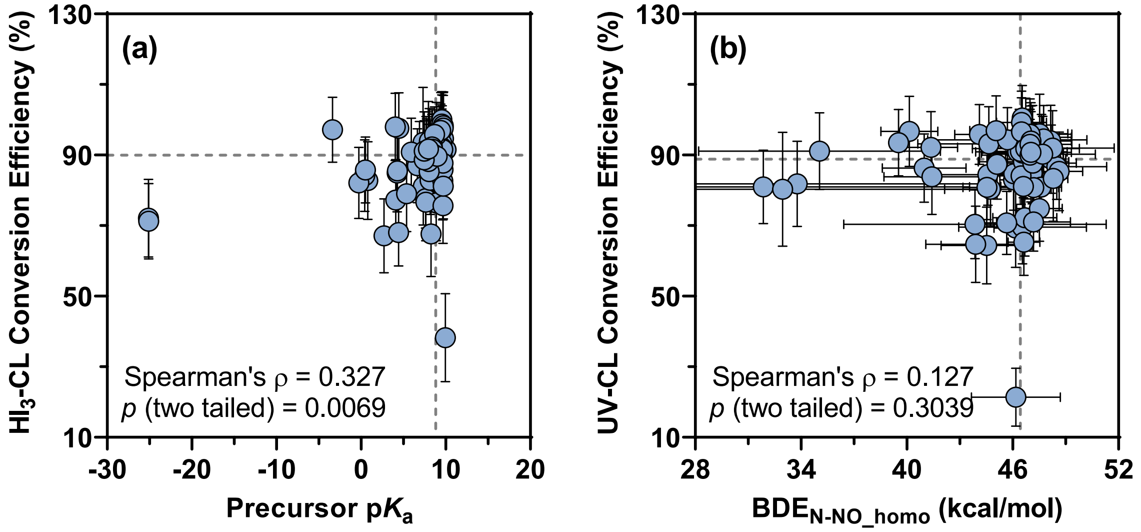

Figure S10. Correlations between the conversion efficiencies of *N*-nitrosamines measured by the acidic triiodide-chemiluminescence (HI_3-CL) and UV photolysis-chemiluminescence (UV-CL) methods and the N-NO bond energetic data: **(a)** Cross plot of the conversion efficiencies of *N*-nitrosamines with reference to NDMA measured by the HI_3-CL method and the pK_a's of corresponding *N*-nitrosamine precursors predicted for 80 °C (i.e., the purge vessel temperature) by *MarvinSketch 22.19.0* in Table S7. Error bars represent the 95% confidence intervals of *N*-nitrosamine conversion efficiencies; where absent, bars fall within symbols. The grey dashed lines mark the median conversion efficiency of *N*-nitrosamines and the median pK_a of their corresponding precursors, respectively. **(b)** Cross plot of the conversion efficiencies of *N*-nitrosamines with reference to NDMA measured by the UV-CL method and the average homolytic N-NO bond dissociation energies ($BDE_{N\text{-}NO\_homo}$) predicted by Fast, Accurate Bond dissociation Enthalpy Tool (ALFABET) and Bond Dissociation Network (BonDNet) in Table S6. Error bars represent the 95% confidence intervals of *N*-nitrosamine conversion efficiencies or the standard deviations of $BDE_{N\text{-}NO\_homo}$ predicted by ALFABET (Fast, Accurate Bond dissociation Enthalpy Tool[13]) and BonDNet (Bond Dissociation Network[14]); where absent, bars fall within symbols. The grey dashed lines mark the median conversion efficiency and the median $BDE_{N\text{-}NO\_homo}$ of *N*-nitrosamines, respectively.

5. Molecular descriptors as predictors for the conversion efficiencies of *N*-nitrosamines

To explore whether molecular descriptors of *N*-nitrosamines might serve as predictors for their conversion efficiencies measured by the HI_3-CL and UV-CL methods, additional regression analyses were performed using the conversion efficiencies as the response variables and 3,979 unique molecular descriptors calculated by *alvaDesc*[16] via the Online Chemical Modeling Environment (OCHEM),[17] *Mordred*,[18] and *PaDEL*[19] as predictor variables. Elastic net regression was first implemented for variable selection using the *glmnet* package[20] in *R 4.2.1* considering the high number of predictor variables (n = 3,979) relative to response variables (n = 67). The regularization parameter λ and the mixing parameter α (i.e., from 0 for Ridge regression to 1 for LASSO regression with a step size of 0.01) were tuned using 10-fold cross-validation based on the training dataset (i.e., 80% of the full dataset). With the optimal combination of λ (i.e., 2.03 for HI_3-CL and 2.43 for UV-CL datasets, respectively) and α (i.e., 0.7 for HI_3-CL and 0.9 for UV-CL datasets, respectively), 24 and 16 molecular descriptors were selected as the most relevant variables for predicting the conversion efficiencies measured by the HI_3-CL and UV-CL methods, respectively. Given the multicollinearity among some of these variables, bidirectional stepwise regression (i.e., a combination of forward selection and backward elimination) was applied to extract a parsimonious collection of statistically significant descriptors (i.e., those with the entry and removal criterion set at a p value of 0.05 and a variance inflation factor of less than 5) using the *olsrr* package[21] in *R*. Cross validation was performed by evaluating the coefficients of determination (Q^2_{CV}) for the validation dataset (i.e., 20% of the training dataset) against the adjusted R-squared values (R^2_{adj}) for stepwise regression of the training dataset over 5,000 iterations. To develop the final quantitative structure-activity relationship (QSAR) models, best subsets regression was implemented to identify the combinations of stepwise-prioritized descriptors that produced the highest coefficients of determination (Q^2) for the test dataset (i.e., 20% of the full dataset). Molecular descriptors selected into the final QSAR models are summarized in **Tables S8** and **S9**.

Overall, the chemical denitrosation of *N*-nitrosamines via HI_3 treatment was influenced by a combination of their electronic, topological, and steric properties, whereas the photolytic denitrosation of *N*-nitrosamines via UV photolysis was largely governed by their electronic properties. *N*-Nitrosamines with a less uniform distribution

of electronegativities distributions (atoms separated by seven bonds; AATSC7e),[22] more stable and symmetric structures (SpMin8_Bhs),[22] a more uneven mass distribution (VE1sign_B(m)),[16] greater atomic displacements and increased flexibility to accommodate conformational changes (DISPv),[16] CH2R2 substructures (C-002),[16] shorter geometric means of topological distances between primary and tertiary carbons (MDEC-13),[22] a more even distribution of atomic numbers across the molecule (atoms that are one bond apart; MATS1Z),[22] more localized charges (atoms pairs separated by four bonds; GATS4c),[22] and a more homogeneous distribution of atomic numbers across the molecule (atoms pairs separated by four bonds; GATS4Z)[22] were more prone to undergo the heterolytic cleavage of the N-NO bond upon HI_3 treatment. *N*-Nitrosamines with a more uniform distribution of ionization potentials at longer distances (atoms pairs separated by seven bonds; MATS7i),[22] a more consistent distribution of intrinsic states at longer distances (atoms pairs separated by six bonds; MATS6s),[22] a less uniform distribution of ionization potentials at shorter distances (atoms pairs separated by three bonds; GATS3i),[22] a more uneven charge distribution (JGI3),[22] more complex topological and electronic structures (SM04_AEA(ri)),[16] and a smaller hydrophobic surface area associated with Log*P* values between 0.00 and 0.10 (SlogP_VSA4)[18] were more prone to undergo the homolytic cleavage of the N-NO bond upon UV irradiation at 254 nm.

**Table S8.** Molecular descriptors selected into the QSAR model for predicting the conversion efficiencies of *N*-nitrosamines measured by the HI_3-CL method								
Descriptor	Description	Estimate	Standard Error	*t* Value	*p* Value	Variance Inflation Factor	R^2_{adj}	Q^2
(Intercept)	-	60.30	8.11	7.43	<0.00001	-	0.84	0.85
AATSC7e	Average centered Broto-Moreau autocorrelation - lag 7 / weighted by Sanderson electronegativities	-48.12	21.97	-2.19	0.03564	1.14		
SpMin8_Bhs	Smallest absolute eigenvalue of Burden modified matrix - n 8 / weighted by relative I-state	10.84	3.22	3.36	0.00196	1.16		
VE1sign_B(m)	Coefficient sum of the last eigenvector from Burden matrix weighted by mass	-18.78	8.56	-2.19	0.03547	1.94		
DISPv	Displacement value / weighted by van der Waals volume	1.97	0.45	4.38	0.00011	1.37		
C-002	CH2R2	2.28	0.39	5.80	<0.00001	1.40		
MDEC-13	Molecular distance edge between all primary and tertiary carbons	-2.72	0.71	-3.83	0.00055	1.37		
MATS1Z	Moran coefficient of lag 1 weighted by atomic number	56.22	17.89	3.14	0.00353	1.32		
GATS4c	Geary coefficient of lag 4 weighted by Gasteiger charge	-12.94	2.34	-5.53	<0.00001	1.37		
GATS4Z	Geary coefficient of lag 4 weighted by atomic number	21.28	5.12	4.15	0.00022	1.41		

Table S9. Molecular descriptors selected into the QSAR model for predicting the conversion efficiencies of *N*-nitrosamines measured by the UV-CL method

Descriptor	Description	Estimate	Standard Error	*t* Value	*p* Value	Variance Inflation Factor	R^2_{adj}	Q^2
(Intercept)	-	133.38	5.88	22.69	<0.00001	-	0.81	0.86
MATS7i	Moran autocorrelation of lag 7 weighted by ionization potential	22.59	6.62	3.41	0.00160	1.18		
MATS6s	Moran autocorrelation of lag 6 weighted by I-state	10.81	2.83	3.83	0.00050	1.09		
GATS3i	Geary autocorrelation of lag 3 weighted by ionization potential	-21.62	5.29	-4.09	0.00023	1.19		
JGI3	Mean topological charge index of order 3	-391.93	49.80	-7.87	<0.00001	1.15		
SM04_AEA(ri)	Spectral moment of order 4 from augmented edge adjacency mat. weighted by resonance integral	0.84	0.40	2.07	0.04588	1.28		
SlogP_VSA4	MOE (Molecular Operating Environment) Log*P* VSA descriptor 4 (0.00 <= x < 0.10)	-0.89	0.33	-2.69	0.01073	1.12		

6. SPE-LC-HRMS method performance and validation

Specific *N*-nitrosamines were quantified by liquid chromatography-high-resolution mass spectrometry (LC-HRMS). LC-HRMS instrument settings and method parameters are summarized in **Tables S10** and **S12**, respectively. SPE extracts were analyzed by a Thermo Fisher Scientific TriPlus RSH autosampler and liquid handling system hyphenated with a Vanquish Horizon ultra-high-performance liquid chromatograph and an Orbitrap Exploris 240 quadrupole-Orbitrap mass spectrometer equipped with an OptaMax NG heated electrospray ionization probe. Typically, 20 µL of SPE extracts were injected onto an Agilent Poroshell 120 phenyl-hexyl column (150 × 2.1 mm, 3 µm; preceded with a 10 × 2.1 mm guard cartridge) running water and methanol (both acidified with 0.1% v/v formic acid) as the mobile phases at a flow rate of 200 µL/min under a column temperature of 35 °C. High-resolution accurate mass screening was performed in positive and negative electrospray ionization modes using the following source parameters: a spray voltage of 3,400 V(+) or 2,000 V(-), a sheath gas of 30 arbitrary units, an auxiliary gas of 5 arbitrary units, a sweep gas of 0 arbitrary units, an ion transfer tube temperature of 320 °C, and a vaporizer temperature of 275 °C. External mass calibration was performed before each analytical run using the Pierce FlexMix calibration solution (Thermo Scientific). Internal mass calibration was enabled through EASY-IC (fluoranthene, *m/z* 202.0782) lock mass during data acquisition. Full scan mass spectra were acquired from 50 to 500 Da with a mass resolution of 60,000 at *m/z* 200 with a radio frequency lens of 70%, a standard automatic gain control target, an automatic maximum injection time mode, an intensity threshold of 10^5, and a mass tolerance of 5 ppm. Full scan triggered data-dependent tandem mass (dd-MS2) spectra were acquired with a mass resolution of 15,000 at *m/z* 200 with an *m/z* isolation window of 2 using higher energy collisional dissociation across four normalized collision energies (i.e., 15%, 30%, 45%, and 60%). *N*-Nitrosamines were confirmed by verifying their chromatographic retention times and dd-MS2 spectra against their respective reference standards. Calibration standards (deionized water spiked with *N*-nitrosamines (1-500 ng/L; 7 calibration levels) plus 100 ng/L of each ILIS and preconcentrated by SPE) and methods blanks (deionized water spiked with ILIS and preconcentrated by SPE) were run with each sample sequence. Quality control samples (deionized water spiked with 100 ng/mL of *N*-nitrosamines and ILIS) were analyzed in triplicate to

evaluate the intraday and interday precisions and accuracies of the LC-HRMS method. For each *N*-nitrosamine, the instrumental limit of detection (LOD),[6] instrumental limit of quantification (LOQ),[6] intraday precision and accuracy, and interday precision and accuracy were calculated by the following equations:

$$\text{LOD (ng/mL)} = 3.3 \times \frac{\text{Standard Deviation of } y\text{-Intercept}_{N\text{-Nitrosamine Calibration Cruve}}}{\text{Slope}_{N\text{-Nitrosamine Calibration Cruve}}} \quad \text{(S5)}$$

$$\text{LOQ (ng/mL)} = 10 \times \frac{\text{Standard Deviation of } y\text{-Intercept}_{N\text{-Nitrosamine Calibration Cruve}}}{\text{Slope}_{N\text{-Nitrosamine Calibration Cruve}}} \quad \text{(S6)}$$

$$\text{Intraday Precision} = \frac{\text{Standard Deviation of Concentration}_{\text{Three Replicate Analyses of QC Sample within 1 Day}}}{\text{Mean Concentration}_{\text{Three Replicate Analyses of QC Sample within 1 Day}}} \times 100\% \quad \text{(S7)}$$

$$\text{Intraday Accuracy} = \frac{\text{Measured Concentration}_{\text{Three Replicate Analyses of QC Sample within 1 Day}} - \text{Target Concentration}}{\text{Target Concentration}} \times 100\% \quad \text{(S8)}$$

$$\text{Interday Precision} = \frac{\text{Standard Deviation of Concentration}_{\text{Three Replicate Analyses of QC Sample over 3 Days}}}{\text{Mean Concentration}_{\text{Three Replicate Analyses of QC Sample over 3 Days}}} \times 100\% \quad \text{(S9)}$$

$$\text{Interday Accuracy} = \frac{\text{Measured Concentration}_{\text{Three Replicate Analyses of QC Sample over 3 Days}} - \text{Target Concentration}}{\text{Target Concentration}} \times 100\% \quad \text{(S10)}$$

Target quantification of *N*-nitrosamines was performed in *TraceFinder 5.1* (Thermo Fisher Scientific) by comparing the ratios of their peak areas to those of assigned ILIS to the corresponding ratios in *N*-nitrosamine calibration standards. Calibration curves for *N*-nitrosamines were constructed in *TraceFinder 5.1* (**Table S11**) by the non-weighted linear least squares regression (mean $R^2 = 0.9938 \pm 0.0087$). The median instrumental LODs and LOQs were 1.03 and 3.10 ng/mL, respectively.

Matrix samples used for method validation included drinking water samples (n = 3) as well as wastewater samples created by mixing equal volumes of influent or effluent samples (n = 3) collected from six full-scale wastewater treatment plants (WWTPs) in Upstate New York. Samples (in triplicate) were either (1) spiked with 100 ng/L of *N*-nitrosamines before SPE (i.e., 100 ng/L into 250 mL of samples subjected to SPE), (2) spiked with

25 ng/mL of *N*-nitrosamines after SPE (i.e., 25 ng into 1 mL of SPE extracts), or (3) not spiked with any *N*-nitrosamine before or after SPE. SPE extracts of spiked and non-spiked samples were further amended with 50 ng/mL of eight ILIS and analyzed by the LC-HRMS method described above. For each *N*-nitrosamine, the method detection limits (MDLs),[23] method quantification limits (MQLs),[24] absolute SPE recoveries,[25] ion suppression/enhancement,[25] and matrix factors[25] were calculated by the following equations:

$$\text{MDL (ng/L)} = 3.143 \times \text{Standard Deviation of Concentration}_{\text{Seven Replicate Analyses of Low-Concentration } N\text{-Nitrosamine Spiked Matrix Sample}} \quad \text{(S11)}$$

$$\text{MQL (ng/L)} = 3 \times \text{MDL} \quad \text{(S12)}$$

$$\text{Ion Suppression/Enhancement} = \frac{\dfrac{\text{Peak Area}_{N\text{-Nitrosamine Spiked in Matrix Sample after SPE}}}{\text{Peak Area}_{\text{ILIS}}} - \dfrac{\text{Peak Area}_{\text{Non-Spiked Matrix Sample after SPE}}}{\text{Peak Area}_{\text{ILIS}}}}{\dfrac{\text{Peak Area}_{N\text{-Nitrosamine Spiked in Deionized Water after SPE}}}{\text{Peak Area}_{\text{ILIS}}}} \times 100\% \quad \text{(S13)}$$

$$\text{Matrix Factor} = \frac{\dfrac{\text{Peak Area}_{N\text{-Nitrosamine Spiked in Matrix Sample before SPE}}}{\text{Peak Area}_{\text{ILIS}}} - \dfrac{\text{Peak Area}_{\text{Non-Spiked Matrix Sample before SPE}}}{\text{Peak Area}_{\text{ILIS}}}}{\dfrac{\text{Peak Area}_{N\text{-Nitrosamine Spiked in Deionized Water before SPE}}}{\text{Peak Area}_{\text{ILIS}}}} \times 100\% \quad \text{(S14)}$$

$$\text{SPE Recovery} = \frac{\dfrac{\text{Peak Area}_{N\text{-Nitrosamine Spiked in Matrix Sample before SPE}}}{\text{Peak Area}_{\text{ILIS}}} - \dfrac{\text{Peak Area}_{\text{Matrix Sample before SPE}}}{\text{Peak Area}_{\text{ILIS}}}}{\dfrac{\text{Peak Area}_{N\text{-Nitrosamine Spiked in Matrix Sample after SPE}}}{\text{Peak Area}_{\text{ILIS}}} - \dfrac{\text{Peak Area}_{\text{Matrix Sample after SPE}}}{\text{Peak Area}_{\text{ILIS}}}} \times 100\% \quad \text{(S15)}$$

SPE-LC-HRMS method validation results are summarized in **Tables S13** and **S14**. On average, the intraday and interday precisions were 2.7±1.8% and 5.5±2.9%, respectively, and the intraday and interday accuracies were 104±8% and 106±15%, respectively. The median MDLs in drinking water, WWTP effluent, and WWTP influent samples were 2.5, 2.6, and 3.1 ng/L, respectively. The median MQLs in drinking water, WWTP effluent, and WWTP influent samples were 7.5, 7.9, and 9.3 ng/L, respectively. On average, the ion suppression/enhancement in WWTP effluent and influent samples were 100±15% and 100±19%, respectively, and the matrix factors in WWTP effluent and influent samples were 94±15% and 93±19%, respectively.

**Table S10.** LC-HRMS instrument settings		
LC TriPlus RSH Autosampler and Liquid Handling System		
Injection Volume (µL)	20	
Bottom Sense Sample Vial	False	
Pre-Clean with Solvent 1	3	
Pre-Clean with Solvent 2	3	
Pre-Clean with Sample	0	
Sample Aspirate Flow Rate (µL/s)	10	
Filling Strokes	2	
Pullup Delay (ms)	500	
Inject Sample Flow Rate (µL/s)	5	
Air Volume (µL)	0	
Post-Clean with Solvent 1	3	
Post-Clean with Solvent 2	3	
Clean Valve with Solvent 1 (µL)	100	
Clean Valve with Solvent 2 (µL)	100	
Sample Vial Depth (mm)	30.0	
Height from Bottom of Sample Vial (mm)	1	
Clean Valve Flow Rate (µL/s)	100	
Wash Volume (%)	70	
Look Ahead	False	
Get Ready Delay (s)	5.0	
Injection Tool	LS1	
Wait For Sample Temperature	False	
sampleTemperature (°C)	4.0	
standbyTemperature (°C)	4.0	
Look Ahead Delay (min)	0.0	
Vanquish Horizon UHPLC System		
Flow Rate: 0.2 mL/min	Mobile Phase A (H_2O + 0.1% FA)	Mobile Phase B (MeOH + 0.1% FA)
Time (min)		
0.0	95%	95%
4.0	95%	95%
7.5	90%	10%
9.5	80%	20%
14.0	20%	80%
15.0	10%	9%
20.0	10%	90%
20.5	95%	5%
26.5	95%	5%
Orbitrap Exploris 240 MS System		
Application Mode: Small Molecule<br>Method Duration (min): 26.5	Positive H-ESI	Negative H-ESI
	Ion Source	
Spray Voltage (V) | Static	3,400	2,000
Sheath Gas (Arb) | Static	30	30
Auxiliary Gas (Arb) | Static	5	5
Sweep Gas (Arb) | Static	0	0
Ion Transfer Tube Temperature (°C)	320	320
Vaporizer Temperature (°C)	275	275
	MS Global Settings	
Infusion Mode	Liquid Chromatography	Liquid Chromatography
Expected LC Peak Width (s)	3	3
Mild Trapping	False	False
Default Charge State	1	1
Enable Xcalibur AcquireX Method Modifications	False	False
Internal Mass Calibration	EASY-IC™	EASY-IC™
Mode	Run Start	Run Start

**Table S10.** LC-HRMS instrument settings (continued)		
LC TriPlus RSH Autosampler and Liquid Handling System		
	Divert Valve A	
Time (min)	Position	Position
0	1 – 6	1 – 6
0.5	1 – 2	1 – 2
30	1 – 6	1 – 6
	Full Scan	
Start Time (min)	0.5	0.5
End Time (min)	26.5	26.5
Orbitrap Resolution FWHM at m/z 200	60,000	60,000
Scan Range (m/z)	50 – 500	50 – 500
RF Lens (%)	70	70
AGC Target	Standard	Standard
Maximum Injection Time Mode	Auto	Auto
Microscans	1	1
Data Type	Profile	Profile
Polarity	Positive	Negative
Source Fragmentation	Disabled	Disabled
	Targeted Mass	
Mass List Type	m/z	m/z
Time Mode	Retention Time Window	Retention Time Window
Include Intensity Threshold	False	False
Mass Tolerance (ppm)	5	5
Set Collision Energy per Compound	False	False
Perform Dependent Scan on Most Intense Ion if No Targets are Found	False	False
	Data Dependent ddMS^2 Scan	
Data Dependent Mode	Number of Scans	Number of Scans
Number of Dependent Scans	10	10
Multiplex Ions	False	False
Isolation Window (m/z)	2	2
Isolation Offset	Off	Off
Collision Energy Type	Normalized	Normalized
HCD Collision Energies (%)	15, 30, 45, 60	15, 30, 45, 60
Orbitrap Resolution FWHM at m/z 200	15,000	15,000
Scan Range Mode	Auto	Auto
AGC Target	Standard	Standard
Maximum Injection Time Mode	Auto	Auto
Microscans	1	1
Data Type	Profile	Profile

**Table S11.** *TraceFinder 5.1* settings	
Peak Detection	
Threshold Override	1 E5
S/N Ratio Threshold	3
Mass Tolerance (ppm)	5
Detection Algorithm	Genesis
Detection Method	Highest peak
Smoothing	7
Threshold	1
Min Peak Height (S/N)	3
Peak S/N cutoff	200
Valley Rise (%)	2
Valley S/N	1.1
# Background Scans	5
Isotopic Pattern	
Fit Threshold (%)	50
Allowed Mass Deviation (ppm)	10
Allowed Intensity Deviation (%)	10

**Table S12.** LC-HRMS method parameters for *N*-nitrosamines and isotope-labeled internal standards							
Compound	Acronym	Adduct	Scan Mode	Quantifier *m/z*	Qualifier *m/z*	Retention Time (min)	Category
N-Nitrosodimethylamine	NDMA	[M+H]+	Full	75.0555	58.0525	3.4	dialkyl
N-Nitrosomethylethylamine	NMEA	[M+H]+	Full	89.0711	61.0397	6.2	dialkyl
N-Nitrosodiethylamine	NDEA	[M+H]+	Full	103.0867	75.0554	12.3	dialkyl
N-Nitrosodipropylamine	NDPA	[M+H]+	Full	131.1179	89.0710	16.0	dialkyl
N-Nitrosodibutylamine	NDBA	[M+H]+	Full	159.1492	103.0867	17.2	dialkyl
N-Nitrosopyrrolidine	NPYR	[M+H]+	Full	101.0710	55.0543	7.5	cyclic
N-Nitrosopiperidine	NPIP	[M+H]+	Full	115.0869	69.0699	13.9	cyclic
N-Nitrosomorpholine	NMOR	[M+H]+	Full	117.0659	87.0679	5.8	heterocyclic
N-Nitrosodiphenylamine	NDPhA	[M+H]+	Full	199.0866	169.0885	17.7	diaryl
N-Nitrosodiethanolamine	NDELA	[M+H]+	Full	135.0764	84.9597	2.6	dialkyl
N-Nitrososarcosine	NSAR	[M+H]+	Full	119.0451	44.0495	3.0	dialkyl
N-Nitrosoproline	NPRO	[M+H]+	Full	145.0608	70.0652	5.4	cyclic
N-Nitrosopipecolic Acid	NPIC	[M+H]+	Full	159.0764	51.9400	13.7	cyclic
N-Nitrosohydroxyproline	NHPRO	[M+H]+	Full	161.0557	68.0496	2.4	cyclic
N-Nitrosothiazolidine-4-Carboxylic Acid	NTCA	[M+H]+	Full	163.0173	133.9660	6.5	heterocyclic
N-Nitroso-2-Methylthiazolidine 4-Carboxylic Acid	NMTCA	[M+H]+	Full	177.0328	147.9656	13.3	heterocyclic
N-Nitrosonornicotine	NNN	[M+H]+	Full	178.0975	148.0996	5.7	cyclic
N-Nitrosoanatabine	NAT	[M+H]+	Full	190.0975	160.0995	11.5	cyclic
N-Nitrosoanabasine	NAB	[M+H]+	Full	192.1131	162.1153	11.9	cyclic
4-(Methylnitrosamino)-1-(3-Pyridyl)-1-Butanone	NNK	[M+H]+	Full	208.1081	122.0601	14.3	dialkyl
4-(Methylnitrosamino)-1-(3-Pyridyl)-1-Butanol	NNAL	[M+H]+	Full	210.1237	180.1259	4.9	dialkyl
N-Nitrosomethylisopropylamine	NMIPA	[M+H]+	Full	103.0867	61.0397	11.6	dialkyl
N-Nitrosoethylpropylamine	NEPA	[M+H]+	Full	117.1023	116.0706	17.9	dialkyl
N-Nitrosomethylbutylamine	NMBA	[M+H]+	Full	117.1023	61.0397	15.7	dialkyl
N-Nitrosomethylisobutylamine	NMIBA	[M+H]+	Full	117.1023	75.0555	15.0	dialkyl
N-Nitroso-*tert*-Butylmethylamine	NTBMA	[M+H]+	Full	117.1023	57.0698	14.4	dialkyl
N-Nitrosomethylamylamine	NMAA	[M+H]+	Full	131.1184	71.0855	16.3	dialkyl
N-Nitrosodiisopropylamine	NDIPA	[M+H]+	Full	131.1184	89.0709	15.6	dialkyl
N-Nitroso-*tert*-Butylethylamine	NTBEA	[M+H]+	Full	131.1179	75.0555	16.0	dialkyl
N-Nitrosomethyl-*N,N*-Dimethylethylamine	NMDEA	[M+H]+	Full	132.1131	58.0653	2.1	dialkyl
N-Nitrosodiisobutylamine	NDIBA	[M+H]+	Full	159.1497	57.0700	17.1	dialkyl
N-Nitrosodiamylamine	NDAA	[M+H]+	Full	187.1810	71.0857	17.9	dialkyl
N-Nitrosoethylbenzylamine	NEBzA	[M+H]+	Full	165.1023	91.0544	16.6	dialkyl
N-Nitrosodibenzylamine	NDBzA	[M+H]+	Full	227.1179	91.0544	17.9	dialkyl
1-Nitroso-4-Methylpiperidine	NMPIP	[M+H]+	Full	129.1023	69.0700	15.6	cyclic
N-Nitroso-2-Pyrrolidinmethanol	NPYRM	[M+H]+	Full	138.0662	108.0685	4.8	cyclic
N-Nitrosopiperazine	NPPZ	[M+H]+	Full	116.0819	86.0840	2.0	heterocyclic
1-Nitroso-4-Phenylpiperazine	NPhPPZ	[M+H]+	Full	192.1131	162.1155	15.6	heterocyclic
N-Nitrosoindoline	NIND	[M+H]+	Full	149.0708	91.0545	16.5	cyclic alkylaryl

Table S12. LC-HRMS method parameters for *N*-nitrosamines and isotope-labeled internal standards (continued)

Compound	Acronym	Adduct	Scan Mode	Quantifier *m/z*	Qualifier *m/z*	Retention Time (min)	Category
1-Nitroso-1,2,3,4-Tetrahydroquinoline	NTHQ	[M+H]+	Full	163.0866	105.0575	17.2	cyclic alkylaryl
N-Nitrosomethylcyclohexylamine	NMChA	[M+H]+	Full	143.1179	61.0398	16.3	alkylcycloalkyl
N-Nitrosodicyclohexylamine	NDChA	[M+H]+	Full	211.1805	83.0857	18.2	dicycloalkyl
N-Nitrosomethylphenylamine	NMPhA	[M+H]+	Full	137.0709	107.0732	16.2	alkylaryl
N-Nitrosoethylphenylamine	NEPhA	[M+H]+	Full	151.0866	105.0450	16.7	alkylaryl
N-Nitroso-*tert*-Butylphenylamine	NTBPhA	[M+H]+	Full	179.1184	93.0575	16.7	alkylaryl
N-Nitroso-4-Methylaminopyridine	NMAPY	[M+H]+	Full	138.0662	108.0685	4.8	alkylheteroaryl
N-Nitrososimazine	NSIM	[M+H]+	Full	231.0756	186.0545	17.2	alkylheteroaryl
N-Nitrosoatrazine	NATR	[M+H]+	Full	245.0912	215.0939	17.6	alkylheteroaryl
N-Nitrosoglyphosate	NGLP	[M+H]+	Full	199.0115	169.0890	1.3	dialkyl
N-Nitrosobetahistine	NBTH	[M+H]+	Full	166.0975	93.0575	3.9	dialkyl
N-Nitrosoephedrine	NEPD	[M+H]+	Full	195.1128	61.0398	15.7	dialkyl
N-Nitrosofenfluramine	NFFA	[M+H]+	Full	261.1214	159.0420	17.4	dialkyl
N-Nitrosonortriptyline	NNTP	[M+H]+	Full	293.1648	233.1333	18.8	dialkyl
N-Nitrosodesipramine	NDSP	[M+H]+	Full	296.1757	266.1784	18.9	dialkyl
N-Nitrosofluoxetine	NFXT	[M+H]+	Full	339.1315	177.1027	18.3	dialkyl
N-Nitrosopropranolol	NPPN	[M+H]+	Full	289.1547	259.1571	18.1	dialkyl
N-Nitrosoatenolol	NATN	[M+H]+	Full	296.1605	266.1793	15.3	dialkyl
N-Nitrosometoprolol	NMTP	[M+H]+	Full	297.1809	223.1571	16.9	dialkyl
N-Nitrososotalol	NSOT	[M+H]+	Full	302.1169	197.0510	15.4	dialkyl
N-Nitrosonadolol	NNAD	[M+H]+	Full	339.1915	309.1946	16.3	dialkyl
N-Nitrosonebivolol	NNBV	[M+H]+	Full	435.1726	405.1761	18.5	dialkyl
N-Nitrosodesloratadine	NDLT	[M+H]+	Full	340.1211	310.1241	17.9	cyclic
N-Nitrosonorfloxacin	NNFX	[M+H]+	Full	349.1307	319.1340	18.6	heterocyclic
N-Nitrosociprofloxacin	NCFX	[M+H]+	Full	361.1307	245.0783	19.2	heterocyclic
N-Nitrosodiclofenac	NDCF	[M-H]-	Full	322.9998	282.9832	17.3	diaryl
N-Nitrosoiminostilbene	NISB	[M+H]+	Full	223.0866	193.0892	17.7	cyclic diaryl
N-Nitrosoiminodibenzyl	NIDB	[M+H]+	Full	225.1022	195.1046	18.0	cyclic diaryl
N-Nitrosodimethyl-d_6-Amine	NDMA-d_6	[M+H]+	Full	81.0932	64.0904	3.2	ILIS
N-Nitrosodiethyl-d_{10}-Amine	NDEA-d_{10}	[M+H]+	Full	113.1494	81.0932	12.0	ILIS
N-Nitrosodi-*n*-Propyl-d_{14}-Amine	NDPA-d_{14}	[M+H]+	Full	145.2058	97.1213	15.8	ILIS
N-Nitrosopyrrolidine-d_8	NPYR-d_8	[M+H]+	Full	109.1212	62.0830	6.8	ILIS
N-Nitrosomorpholine-d_8	NMOR-d_8	[M+H]+	Full	125.1161	95.1184	5.5	ILIS
N-Nitrosodiphenyl-2,2',4,4',6,6'-d_6-Amine	NDPhA-d_6	[M+H]+	Full	205.1239	175.1267	17.7	ILIS
N-Nitrosobis(2-Hydroxyethyl)-d_8-Amine	NDELA-d_8	[M+H]+	Full	143.1266	113.1285	2.4	ILIS
N-Methyl-d_3-*N*-Nitrosoglycine	NSAR-d_3	[M+H]+	Full	122.0640	104.9636	2.9	ILIS

ILIS = Isotope-labeled internal standard.

Table S13. SPE-LC-HRMS method validation for *N*-nitrosamines											
Compound	LOD (ng/mL)	LOQ (ng/mL)	R^2	Intraday Precision	Intraday Accuracy	Interday Precision	Interday Accuracy	MDL (ng/L)	MQL (ng/L)	Ion Suppression / Enhancement	Matrix Factor
NDMA	0.96	2.88	0.9986	1.6%	2.8%	115%	118%	2.2 – 2.4	6.6 – 7.3	99(±14)%	94(±12)%
NMEA	0.75	2.25	0.9964	1.5%	3.3%	117%	120%	1.9 – 2.2	5.6 – 6.6	96(±15)%	92(±10)%
NDEA	0.82	2.45	0.9953	2.0%	7.0%	114%	118%	1.9 – 2.3	5.6 – 6.9	96(±14)%	94(±10)%
NDPA	0.63	1.88	0.9964	2.1%	2.1%	110%	120%	1.5 – 1.7	4.4 – 5.2	101(±14)%	96(±13)%
NDBA	0.68	2.04	0.9981	2.0%	4.4%	104%	112%	1.9 – 2.3	5.6 – 6.9	102(±14)%	90(±15)%
NPYR	1.16	3.48	0.9985	1.0%	1.0%	103%	114%	2.5 – 2.7	7.4 – 8.2	99(±14)%	95(±11)%
NPIP	0.74	2.22	0.9968	3.2%	6.0%	112%	122%	1.6 – 1.8	4.8 – 5.4	98(±16)%	93(±10)%
NMOR	0.83	2.50	0.9985	1.0%	1.6%	105%	114%	1.8 – 2.2	5.3 – 6.6	98(±14)%	94(±11)%
NDPhA	1.34	4.02	0.9982	1.6%	3.9%	92%	86%	9.2 – 11	28 – 34	101(±13)%	88(±17)%
NDELA	4.85	14.56	0.9670	8.9%	13.8%	113%	126%	20 – 24	61 – 73	114(±24)%	110(±19)%
NSAR	3.32	9.97	0.9923	1.0%	3.8%	106%	119%	-	-	97(±18)%	-
NPRO	1.47	4.41	0.9985	7.6%	10.7%	104%	114%	-	-	116(±15)%	-
NPIC	0.83	2.48	0.9920	2.1%	3.9%	116%	123%	-	-	84(±16)%	-
NHPRO	1.80	5.39	0.9928	3.3%	4.2%	111%	128%	5.9 – 6.5	18 – 20	120(±15)%	113(±19)%
NTCA	5.39	16.17	0.9460	2.5%	4.5%	111%	116%	-	-	100(±26)%	-
NMTCA	8.10	24.30	0.9604	2.2%	5.2%	110%	120%	-	-	78(±9)%	-
NNN	1.13	3.39	0.9981	1.5%	4.0%	101%	112%	8.2 – 9.4	25 – 28	102(±13)%	-
NAT	0.86	2.58	0.9885	1.9%	5.2%	110%	124%	6.4 – 8.2	19 – 25	91(±15)%	-
NAB	1.20	3.59	0.9812	4.7%	8.5%	101%	107%	7.1 – 10	21 – 31	107(±18)%	-
NNK	0.62	1.85	0.9927	1.8%	4.0%	96%	87%	4.4 – 5.3	13 – 16	95(±13)%	-
NNAL	2.59	7.77	0.9979	1.5%	4.6%	104%	109%	-	-	97(±16)%	-
NMIPA	2.51	7.52	0.9982	1.0%	2.5%	106%	116%	6.1 – 8.0	18 – 24	82(±16)%	84(±14)%
NEPA	0.75	2.26	0.9934	1.4%	3.5%	106%	107%	1.6 – 1.7	4.8 – 5.0	106(±14)%	101(±14)%
NMBA	1.69	5.08	0.9920	1.9%	2.9%	105%	107%	3.3 – 3.8	10 – 12	107(±15)%	101(±13)%
NMIBA	0.78	2.35	0.9933	1.6%	2.9%	106%	104%	1.7 – 1.9	5.2 – 5.8	108(±14)%	101(±14)%
NTBMA	0.73	2.19	0.9936	1.7%	3.4%	107%	104%	1.6 – 1.9	4.8 – 5.8	93(±18)%	92(±15)%
NMAA	0.63	1.88	0.9964	2.1%	2.6%	112%	120%	1.4 – 1.8	4.3 – 5.4	97(±14)%	96(±11)%
NDIPA	0.63	1.88	0.9964	1.9%	2.1%	108%	120%	1.5 – 1.8	4.5 – 5.3	102(±14)%	97(±13)%
NTBEA	0.73	2.19	0.9960	3.9%	9.8%	108%	115%	1.7 – 1.9	5.2 – 5.6	101(±14)%	99(±14)%
NMDEA	3.09	9.26	0.9963	1.9%	5.2%	106%	105%	-	-	82(±18)%	-
NDIBA	0.68	2.04	0.9981	2.0%	7.9%	104%	110%	2.0 – 2.1	6.0 – 6.3	103(±13)%	95(±13)%
NDAA	0.80	2.41	0.9949	1.9%	2.8%	103%	112%	3.3 – 3.7	10 – 11	107(±14)%	85(±15)%
NEBzA	1.03	3.08	0.9950	4.4%	11.5%	102%	117%	2.2 – 3.3	6.5 – 9.8	87(±12)%	87(±15)%
NDBzA	1.10	3.29	0.9981	4.5%	9.4%	103%	93%	6.4 – 7.3	19 – 22	111(±16)%	99(±22)%
NMPIP	1.69	5.06	0.9948	8.9%	12.7%	108%	90%	3.3 – 3.5	10 – 11	109(±15)%	57(±16)%
NPYRM	1.42	4.25	0.9970	3.1%	6.5%	111%	127%	-	-	98(±7)%	-
NPPZ	1.14	3.43	0.9958	1.9%	7.0%	97%	87%	-	-	106(±20)%	-
NPhPPZ	1.71	5.14	0.9985	2.3%	3.1%	98%	89%	5.5 – 6.1	16 – 18	107(±13)%	92(±14)%
NIND	0.74	2.23	0.9922	1.3%	2.5%	98%	88%	-	-	79(±21)%	-

Table S13. SPE-LC-HRMS method validation for *N*-nitrosamines (continued)

Compound	LOD (ng/mL)	LOQ (ng/mL)	R^2	Intraday Precision	Interday Precision	Intraday Accuracy	Interday Accuracy	MDL (ng/L)	MQL (ng/L)	Ion Suppression / Enhancement	Matrix Factor
NTHQ	1.15	3.44	0.9973	5.3%	12.1%	95%	88%	-	-	79(±23)%	-
NMChA	0.88	2.65	0.9954	1.2%	2.5%	102%	110%	1.8 – 2.2	5.5 – 6.6	94(±14)%	91(±17)%
NDChA	0.77	2.30	0.9982	1.7%	5.1%	95%	98%	3.1 – 3.2	9.4 – 9.6	112(±17)%	93(±19)%
NMPhA	0.77	2.30	0.9954	1.9%	6.1%	114%	119%	-	-	102(±12)%	-
NEPhA	0.91	2.73	0.9987	2.3%	7.7%	106%	114%	5.2 – 5.6	16 – 17	93(±15)%	100(±22)%
NTBPhA	0.93	2.79	0.9966	2.3%	4.5%	113%	128%	2.2 – 2.5	6.7 – 7.5	98(±16)%	94(±17)%
NMAPY	1.42	4.25	0.9970	3.1%	6.5%	111%	127%	-	-	98(±7)%	-
NSIM	0.99	2.97	0.9939	3.1%	8.1%	91%	82%	-	-	83(±22)%	-
NATR	0.83	2.50	0.9979	2.4%	8.1%	87%	84%	5.2 – 6.4	16 – 19	98(±20)%	99(±17)%
NGLP	-	-	-	-	-	-	-	-	-	-	-
NBTH	2.20	6.60	0.9966	1.1%	2.8%	114%	123%	-	-	106(±15)%	-
NEPD	0.92	2.76	0.9975	2.8%	6.0%	90%	87%	2.8 – 3.4	8.5 – 10	101(±13)%	101(±24)%
NFFA	1.04	3.12	0.9981	1.3%	5.0%	92%	88%	2.5 – 3.0	7.4 – 9.0	99(±15)%	91(±19)%
NNTP	1.30	3.91	0.9971	2.8%	4.6%	117%	118%	-	-	103(±14)%	-
NDSP	0.31	0.92	0.9970	4.6%	8.3%	108%	98%	1.2 – 1.3	3.6 – 4.0	111(±14)%	92(±16)%
NFXT	0.40	1.19	0.9945	5.6%	9.5%	93%	85%	1.6 – 2.1	4.8 – 6.2	107(±16)%	61(±12)%
NPPN	1.42	4.27	0.9976	1.0%	4.2%	102%	91%	-	-	106(±16)%	-
NATN	1.79	5.37	0.9976	2.3%	3.1%	89%	88%	-	-	112(±11)%	-
NMTP	1.06	3.19	0.9949	1.9%	4.3%	104%	105%	3.8 – 4.5	11 – 13	102(±12)%	108(±19)%
NSOT	1.63	4.88	0.9959	4.2%	5.0%	84%	81%	-	-	103(±13)%	-
NNAD	0.26	0.79	0.9979	1.5%	6.1%	98%	92%	-	-	119(±15)%	-
NNBV	0.42	1.25	0.9905	1.6%	3.2%	93%	83%	-	-	119(±14)%	-
NDLT	0.65	1.95	0.9943	2.1%	7.3%	106%	92%	2.1 – 2.9	6.4 – 8.7	105(±15)%	95(±18)%
NNFX	3.87	11.62	0.9981	4.5%	7.8%	113%	123%	-	-	97(±15)%	-
NCFX	3.89	11.68	0.9930	5.9%	8.5%	117%	127%	-	-	98(±22)%	-
NDCF	2.66	7.98	0.9945	2.1%	7.3%	96%	83%	-	-	91(±10)%	-
NISB	3.18	9.53	0.9918	5.6%	7.2%	94%	91%	13 – 16	40 – 47	110(±25)%	98(±23)%
NIDB	1.13	3.40	0.9909	3.0%	3.6%	103%	115%	-	-	96(±17)%	-

LOD = Instrumental limit of detection at 20 µL injection volume. LOQ = Instrumental limit of quantification at 20 µL injection volume. MDL = Method detection limit (given as a range measured in deionized water, WWTP effluent, and WWTP influent samples). MQL = Method quantification limit (given as a range measured in deionized water, WWTP effluent, and WWTP influent samples). Ion suppression / enhancement calculated by Equation S13 (given as mean ± (standard deviation) measured in WWTP effluent and influent samples). Matrix factors calculated by Equation S14 (given as mean ± (standard deviation) measured in WWTP effluent and influent samples).

Table S14. SPE recoveries of *N*-nitrosamines

Compound	K_H (atm-m^3/mol)	VP (mmHg)	Log*P*	Log$D_{pH=2}$	Molecular Weight	Drinking Water (*n* = 3)	WWTP Effluent (*n* = 3)	WWTP Influent (*n* = 3)
NDMA	1.74×10^{-6}	2.69	0.04	-0.04	74.08	87(±6)%	84(±8)%	80(±6)%
NMEA	9.12×10^{-7}	2.63	0.40	0.31	88.11	80(±6)%	77(±8)%	72(±8)%
NDEA	3.63×10^{-6}	8.51×10^{-1}	0.75	0.67	102.14	87(±5)%	77(±6)%	75(±4)%
NDPA	3.72×10^{-6}	1.91×10^{-1}	1.80	1.72	130.19	85(±4)%	79(±3)%	74(±6)%
NDBA	1.35×10^{-5}	4.68×10^{-2}	2.69	2.61	158.25	73(±8)%	65(±5)%	60(±6)%
NPYR	6.03×10^{-8}	6.17×10^{-2}	0.44	0.36	100.12	94(±6)%	92(±7)%	87(±8)%
NPIP	8.91×10^{-7}	9.12×10^{-2}	0.89	0.81	114.15	92(±5)%	87(±10)%	84(±12)%
NMOR	2.57×10^{-8}	1.20	-0.18	-0.26	116.12	94(±4)%	84(±8)%	77(±13)%
NDPhA	1.12×10^{-3}	7.76×10^{-4}	3.35	3.35	198.23	29(±3)%	26(±2)%	25(±1)%
NDELA	8.13×10^{-11}	2.63×10^{-7}	-1.34	-1.42	134.14	48(±4)%	39(±1)%	34(±6)%
NSAR	1.62×10^{-7}	1.10×10^{-3}	-0.62	-0.57	118.09	-	-	-
NPRO	3.24×10^{-8}	7.94×10^{-6}	0.01	0.05	144.13	-	-	-
NPIC	3.09×10^{-8}	7.41×10^{-6}	0.48	0.50	158.16	-	-	-
NHPRO	1.12×10^{-9}	3.16×10^{-7}	-1.17	-1.10	160.13	55(±5)%	48(±6)%	49(±5)%
NTCA	5.62×10^{-9}	1.82×10^{-5}	-0.16	-0.08	162.16	-	-	-
NMTCA	5.13×10^{-9}	1.26×10^{-5}	0.19	0.23	176.19	-	-	-
NNN	3.63×10^{-5}	1.70×10^{-4}	1.01	-0.04	177.21	24(±2)%	27(±2)%	27(±3)%
NAT	2.29×10^{-5}	5.37×10^{-4}	1.25	0.20	189.22	27(±3)%	27(±3)%	24(±1)%
NAB	1.86×10^{-5}	4.90×10^{-4}	1.46	0.40	191.23	24(±8)%	27(±3)%	30(±6)%
NNK	2.14×10^{-8}	2.69×10^{-7}	0.58	-0.49	207.23	23(±2)%	28(±4)%	29(±1)%
NNAL	7.41×10^{-9}	3.47×10^{-7}	0.49	-0.57	209.25	-	-	-
NMIPA	3.98×10^{-6}	1.20	0.81	0.73	102.14	82(±4)%	76(±7)%	77(±3)%
NEPA	5.62×10^{-6}	4.37×10^{-1}	1.27	1.19	116.16	94(±2)%	90(±4)%	81(±10)%
NMBA	5.75×10^{-6}	5.89×10^{-1}	1.36	1.28	116.16	94(±3)%	87(±3)%	90(±9)%
NMIBA	5.62×10^{-6}	9.33×10^{-1}	1.28	1.20	116.16	89(±13)%	86(±2)%	73(±3)%
NTBMA	5.75×10^{-6}	5.13×10^{-1}	1.09	1.01	116.16	92(±5)%	87(±3)%	92(±6)%
NMAA	5.01×10^{-6}	1.41×10^{-1}	1.81	1.73	130.19	88(±13)%	82(±8)%	80(±4)%
NDIPA	8.13×10^{-6}	3.98×10^{-1}	1.59	1.51	130.19	83(±11)%	78(±1)%	67(±2)%
NTBEA	6.76×10^{-6}	3.89×10^{-1}	1.45	1.37	130.19	85(±5)%	81(±4)%	75(±5)%
NMDEA	2.45×10^{-7}	3.80×10^{-1}	0.06	-3.52	131.18	-	-	-
NDIBA	7.76×10^{-6}	1.66	2.53	2.45	158.25	65(±5)%	67(±8)%	64(±2)%
NDAA	2.00×10^{-5}	1.48×10^{-3}	3.58	3.49	186.30	48(±1)%	43(±14)%	43(±4)%
NEBzA	2.40×10^{-6}	1.48×10^{-1}	2.12	2.04	164.21	95(±4)%	83(±4)%	75(±4)%
NDBzA	3.55×10^{-6}	5.13×10^{-7}	3.49	3.41	226.28	31(±6)%	29(±9)%	29(±5)%
NMPIP	1.41×10^{-6}	2.04×10^{-2}	1.18	1.10	128.18	96(±4)%	93(±1)%	92(±5)%
NPYRM	3.09×10^{-7}	2.14×10^{-5}	-0.19	-0.26	130.15	48(±12)%	44(±8)%	45(±5)%
NPPZ	1.20×10^{-5}	1.07×10^{-1}	-0.50	-3.79	115.14	-	-	-
NPhPPZ	1.00×10^{-5}	1.10×10^{-3}	1.78	0.94	191.23	63(±2)%	55(±5)%	52(±5)%
NIND	2.19×10^{-6}	3.80×10^{-2}	1.74	1.74	148.17	-	-	-

Table S14. SPE recoveries of *N*-nitrosamines (continued)								
Compound	K_H (atm-m^3/mol)	VP (mmHg)	Log$P$	Log$D_{pH=2}$	Molecular Weight	Drinking Water (n = 3)	WWTP Effluent (n = 3)	WWTP Influent (n = 3)
NTHQ	1.02×10^{-5}	9.33×10^{-3}	2.18	2.18	162.19	-	-	-
NMChA	1.82×10^{-6}	1.62×10^{-2}	1.84	1.76	142.20	96(±6)%	91(±6)%	89(±10)%
NDChA	1.86×10^{-6}	8.71×10^{-3}	3.64	3.56	210.32	48(±9)%	44(±10)%	42(±7)%
NMPhA	3.63×10^{-6}	8.71×10^{-1}	1.70	1.70	136.15	-	-	-
NEPhA	4.07×10^{-6}	7.41×10^{-1}	2.05	2.05	150.18	32(±3)%	35(±3)%	39(±6)%
NTBPhA	9.77×10^{-7}	2.63×10^{-1}	2.75	2.75	178.24	84(±9)%	80(±3)%	78(±2)%
NMAPY	5.89×10^{-6}	5.37×10^{-2}	0.48	-0.45	137.14	-	-	-
NSIM	1.82×10^{-8}	1.26×10^{-6}	2.03	2.03	230.66	-	-	-
NATR	1.74×10^{-8}	7.59×10^{-7}	2.45	2.45	244.68	32(±4)%	30(±5)%	29(±3)%
NGLP	7.24×10^{-11}	1.07×10^{-5}	-2.42	-2.42	198.07	-	-	-
NBTH	2.29×10^{-6}	4.17×10^{-2}	0.86	-0.66	165.20	-	-	-
NEPD	7.94×10^{-8}	1.48×10^{-5}	1.55	1.47	194.23	61(±1)%	67(±4)%	51(±8)%
NFFA	9.12×10^{-5}	2.29×10^{-2}	3.70	3.62	260.26	83(±10)%	78(±8)%	77(±8)%
NNTP	3.55×10^{-8}	4.17×10^{-7}	4.66	4.58	292.38	-	-	-
NDSP	3.72×10^{-7}	1.86×10^{-8}	4.13	3.93	295.39	47(±1)%	45(±8)%	41(±3)%
NFXT	4.68×10^{-6}	7.59×10^{-8}	4.40	4.32	338.33	50(±2)%	39(±8)%	34(±2)%
NPPN	4.79×10^{-11}	7.59×10^{-8}	2.82	2.74	288.35	-	-	-
NATN	9.55×10^{-10}	5.75×10^{-8}	0.66	0.58	295.34	-	-	-
NMTP	4.37×10^{-11}	1.26×10^{-5}	1.99	1.91	296.37	57(±4)%	53(±3)%	45(±8)%
NSOT	3.80×10^{-10}	1.86×10^{-9}	0.28	0.20	301.36	-	-	-
NNAD	8.71×10^{-11}	1.86×10^{-7}	1.10	1.02	338.40	-	-	-
NNBV	6.17×10^{-11}	6.76×10^{-7}	3.44	3.36	434.44	-	-	-
NDLT	1.48×10^{-10}	1.48×10^{-9}	4.20	2.72	339.82	61(±6)%	54(±14)%	42(±9)%
NNFX	1.55×10^{-9}	3.31×10^{-9}	1.68	1.59	348.33	-	-	-
NCFX	3.31×10^{-10}	1.45×10^{-6}	1.78	1.70	360.35	-	-	-
NDCF	1.66×10^{-9}	2.19×10^{-7}	4.20	4.19	325.15	-	-	-
NISB	1.02×10^{-5}	1.07×10^{-6}	3.72	3.72	222.25	41(±5)%	43(±8)%	38(±1)%
NIDB	1.10×10^{-5}	4.07×10^{-7}	3.92	3.92	224.26	-	-	-

K_H = Henry's law constant (at 25 °C) and VP = vapor pressure calculated by *OPERA V2.9.1.*;[26] Log$P$ and Log$D$ (at pH 2; the average pH of samples treated by 20 mM of sulfamic acid) calculated by *JChem for Excel (Version 20.2.0.589)*.[27] SPE recoveries calculated by Equation S15 (given as mean ± (standard deviation) measured in drinking water, WWTP effluent, and WWTP influent samples, respectively.)

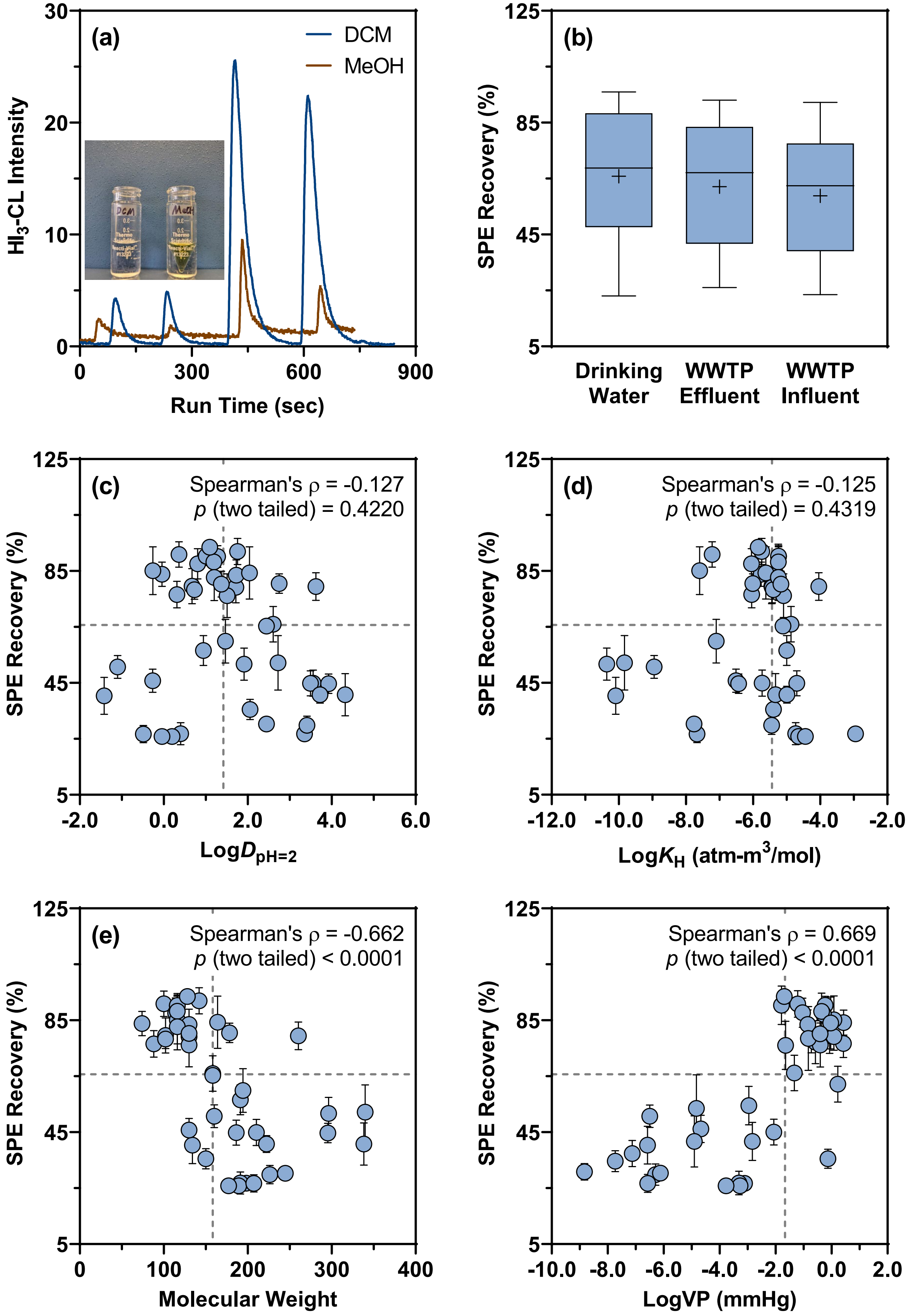
(a)
DCM
MeOH
HI3-CL Intensity
Run Time (sec)
(b)
SPE Recovery (%)
Drinking Water
WWTP Effluent
WWTP Influent
(c)
Spearman's ρ = -0.127
p (two tailed) = 0.4220
LogD pH=2
(d)
Spearman's ρ = -0.125
p (two tailed) = 0.4319
LogK H (atm-m3/mol)
(e)
Spearman's ρ = -0.662
p (two tailed) < 0.0001
Molecular Weight
Spearman's ρ = 0.669
p (two tailed) < 0.0001
LogVP (mmHg)

Figure S11. Solid-phase extraction (SPE) recoveries of *N*-nitrosamines in different matrix samples in relation to selected molecular properties: **(a)** Example chemiluminescence peaks resulting from injections of wastewater samples extracted by dichloromethane (DCM) or methanol (MeOH) with the acidic triiodide-chemiluminescence (HI_3-CL) method. The inset photo compares the color change of SPE extracts by switching from MeOH (the vial on the right) to DCM (the vial on the left) as the SPE cartridge eluant. Meints[28] suggested that DCM introduced interferences in chemiluminescence detection, which was not observed with our SPE protocol as DCM was completely removed during rotary evaporation. **(b)** Box-and-whiskers plots of SPE recoveries of *N*-nitrosamines in drinking water, WWTP effluent, and WWTP influent samples. Each box extends from the 25th to 75th percentiles. The whiskers extend down to the 25th percentile minus 1.5 times the interquartile range and up to the 75th percentile plus 1.5 times the interquartile range. The centerline and "+" sign in each box mark the median and mean, respectively. **(c)** Cross plot of the average SPE recoveries of *N*-nitrosamines in different matrices and their Log*D* values at pH 2 (i.e., the average pH of samples treated by 20 mM of sulfamic acid). Error bars represent the standard deviations of SPE recoveries in different matrices; where absent, bars fall within symbols. The grey dashed lines mark the median SPE recovery and the median $\text{Log}D_{\text{pH=2}}$ of *N*-nitrosamines, respectively. **(d)** Cross plot of the average SPE recoveries of *N*-nitrosamines in different matrices and their Henry's law constant (K_H). Error bars represent the standard deviations of SPE recoveries in different matrices; where absent, bars fall within symbols. The grey dashed lines mark the median SPE recovery and the median $\text{Log}K_H$ of *N*-nitrosamines, respectively. **(e)** Cross plot of the average SPE recoveries of *N*-nitrosamines in different matrices and their molecular weights. Error bars represent the standard deviations of SPE recoveries in different matrices; where absent, bars fall within symbols. The grey dashed lines mark the median SPE recovery and the median molecular weight of *N*-nitrosamines, respectively. **(f)** Cross plot of the average SPE recoveries of *N*-nitrosamines in different matrices and their vapor pressures (VP). Error bars represent the standard deviations of SPE recoveries in different matrices; where absent, bars fall within symbols. The grey dashed lines mark the median SPE recovery and the median LogVP of *N*-nitrosamines, respectively.

Table S15. SPE recoveries of *N*-nitrosamines reported in the literature

Compound	Drinking Water	WWTP Effluent	WWTP Influent	Reference
NDMA	108(±15)%	115(±5)%	116(±44)%	Krauss and Hollender[29] SPE Sorbents: Waters Oasis HLB + Mallinckrodt-Baker Bakerbond Carbon
NMOR	118(±4)%	119(±4)%	133(±24)%	
NMEA	58(±10)%	77(±3)%	0%	
NPYR	110(±4)%	117(±6)%	0%	
NDEA	78(±2)%	82(±7)%	57(±8)%	
NPIP	108(±1)%	102(±5)%	78(±9)%	
NDPA	70(±3)%	64(±2)%	32(±1)%	
NDBA	48(±11)%	54(±5)%	52(±8)%	
NDPhA	38(±8)%	20(±8)%	0%	
NDMA	45%	-	-	Dai *et al.*[30] SPE Sorbent: United Chemical Technologies Enviro-Clean 521 Activated Carbon
NDELA	76%	-	-	
NSAR	87%	-	-	
NDMA	48(±5)%	-	-	Zeng and Mitch[31] SPE Sorbent: Enviro-Clean 521 Activated Carbon
NMOR	71(±8)%	-	-	
NDMA	-	46-61%	-	Breider and von Gunten[5] SPE Sorbent: Waters Oasis HLB
NMEA	-	46-61%	-	
NDEA	-	46-61%	-	
NDPA	-	46-61%	-	
NDBA	-	46-61%	-	
NDELA	-	46-61%	-	
NDPhA	-	46-61%	-	
NPIP	-	46-61%	-	
NPYR	-	46-61%	-	
NDMA	56(±3)%	-	-	Meints[28] SPE Sorbent: Restek EPA Method 521 Activated Charcoal

7. Effects of wastewater dilution on the spike recoveries of NDMA and the *N*-nitrosamine mixture

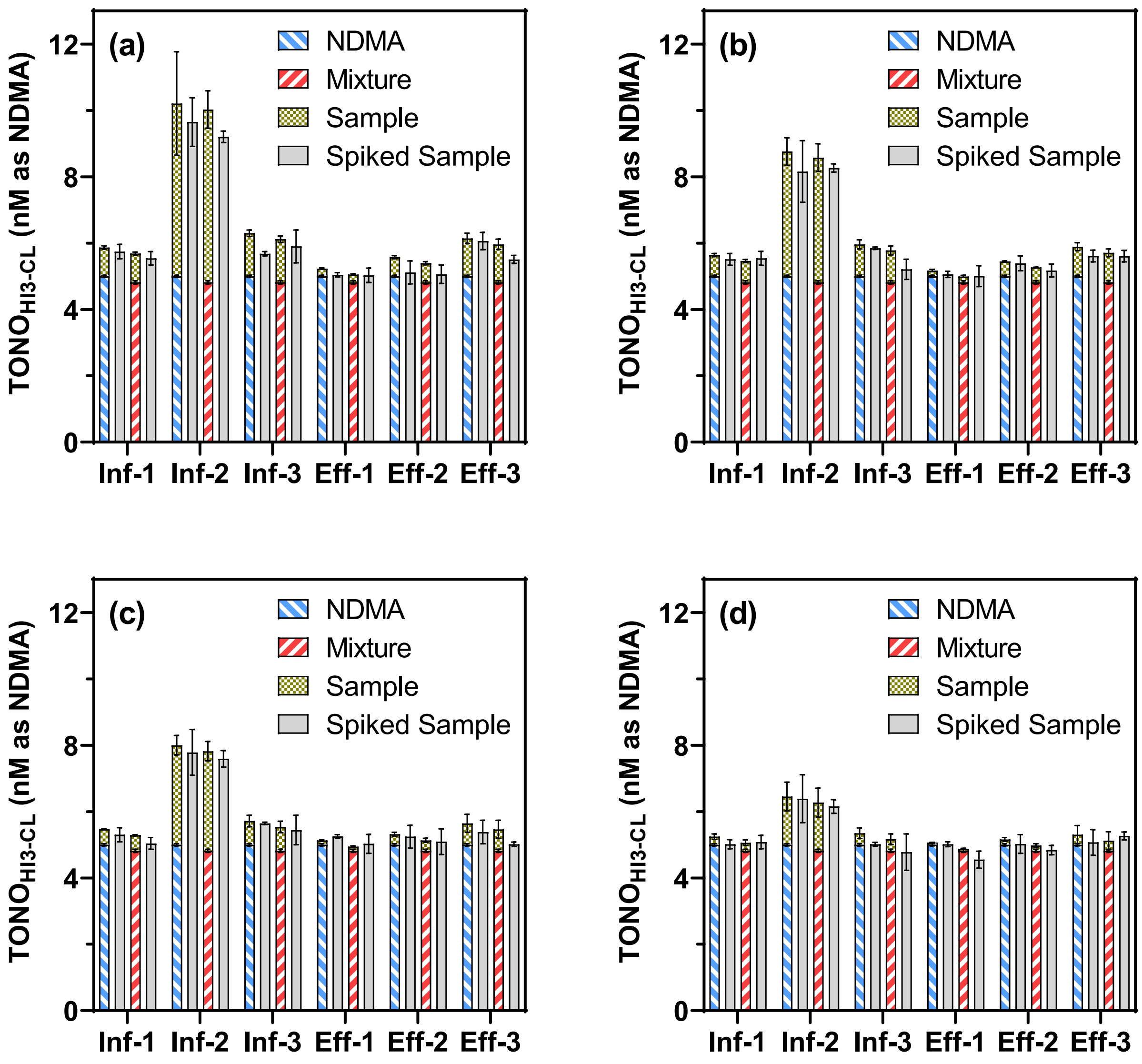

Figure S12. Effects of serial dilution on the spike recoveries of NDMA and the *N*-nitrosamine mixture (~5 nM) in wastewater: **(a)** Spike recoveries of NDMA and the equal-molar mixture of *N*-nitrosamines (Mixture) in six undiluted (i.e., 100%) WWTP influent (Inf) and effluent (Eff) samples when measured as $TONO_{HI_3\text{-}CL}$. Error bars represent the standard deviations of $TONO_{HI_3\text{-}CL}$. **(b)** Spike recoveries of NDMA and the equal-molar mixture of *N*-nitrosamines (Mixture) in six partially diluted (i.e., 75%) WWTP influent (Inf) and effluent (Eff) samples when measured as $TONO_{HI_3\text{-}CL}$. Error bars represent the standard deviations of $TONO_{HI_3\text{-}CL}$. **(c)** Spike recoveries of NDMA and the equal-molar mixture of *N*-nitrosamines (Mixture) in six partially diluted (i.e., 50%) WWTP influent (Inf) and effluent (Eff) samples when measured as $TONO_{HI_3\text{-}CL}$. Error bars represent the standard deviations of $TONO_{HI_3\text{-}CL}$. **(d)** Spike recoveries of NDMA and the equal-molar mixture of *N*-nitrosamines (Mixture) in six partially diluted (i.e., 25%) WWTP influent (Inf) and effluent (Eff) samples when measured as $TONO_{HI_3\text{-}CL}$. Error bars represent the standard deviations of $TONO_{HI_3\text{-}CL}$.

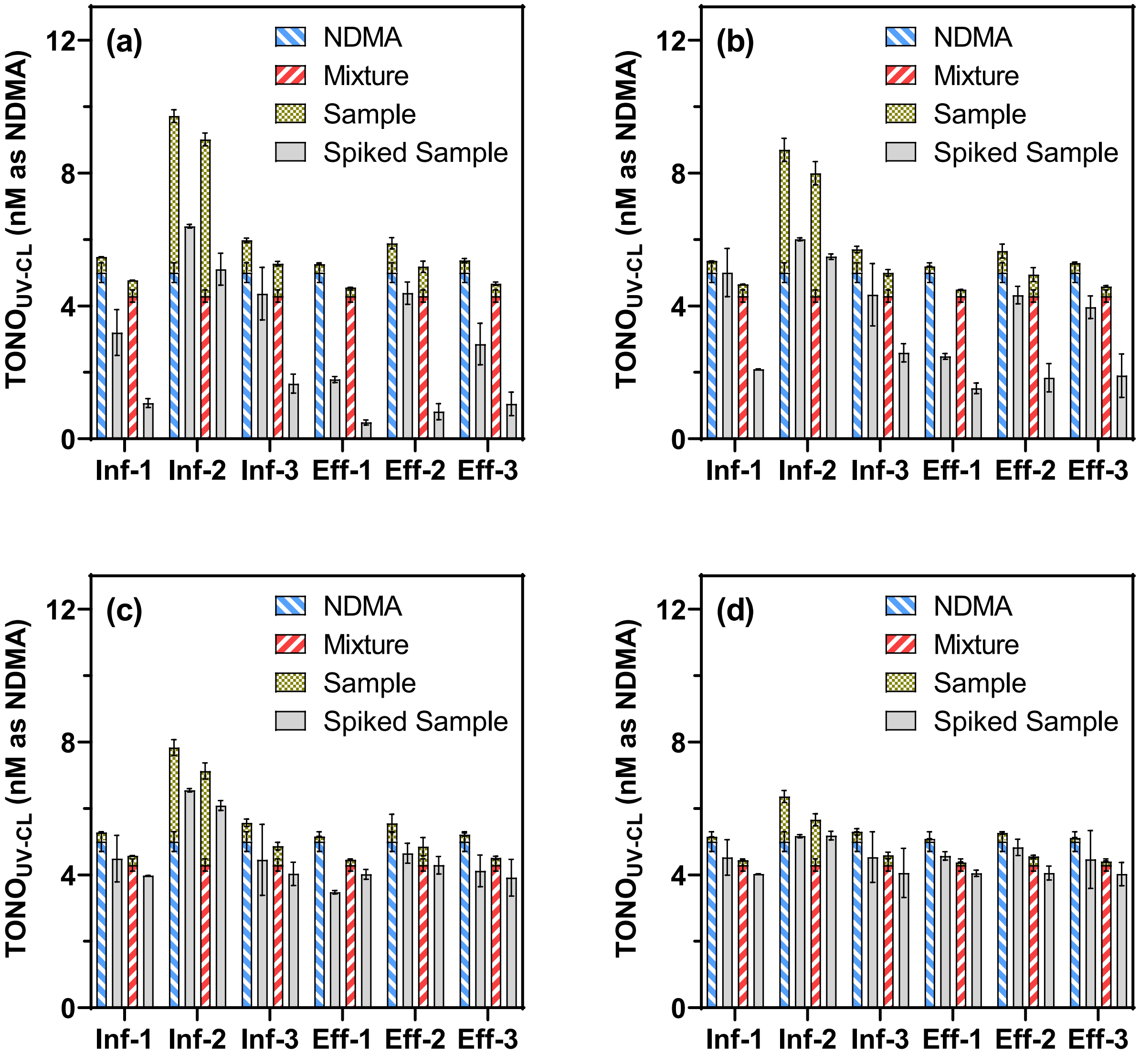

Figure S13. Effects of serial dilution on the spike recoveries of NDMA and the *N*-nitrosamine mixture (~5 nM) in wastewater: **(a)** Spike recoveries of NDMA and the equal-molar mixture of *N*-nitrosamines (Mixture) in six undiluted (i.e., 100%) WWTP influent (Inf) and effluent (Eff) samples when measured as TONO$_{UV-CL}$. Error bars represent the standard deviations of TONO$_{UV-CL}$. **(b)** Spike recoveries of NDMA and the equal-molar mixture of *N*-nitrosamines (Mixture) in six partially diluted (i.e., 75%) WWTP influent (Inf) and effluent (Eff) samples when measured as TONO$_{UV-CL}$. Error bars represent the standard deviations of TONO$_{UV-CL}$. **(c)** Spike recoveries of NDMA and the equal-molar mixture of *N*-nitrosamines (Mixture) in six partially diluted (i.e., 50%) WWTP influent (Inf) and effluent (Eff) samples when measured as TONO$_{UV-CL}$. Error bars represent the standard deviations of TONO$_{UV-CL}$. **(d)** Spike recoveries of NDMA and the equal-molar mixture of *N*-nitrosamines (Mixture) in six partially diluted (i.e., 25%) WWTP influent (Inf) and effluent (Eff) samples when measured as TONO$_{UV-CL}$. Error bars represent the standard deviations of TONO$_{UV-CL}$.

8. Example LC-HRMS chromatograms of *N*-nitrosamines

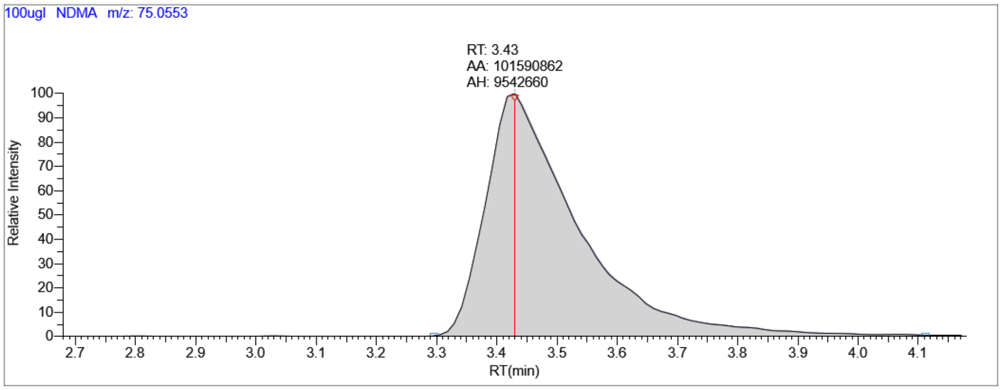

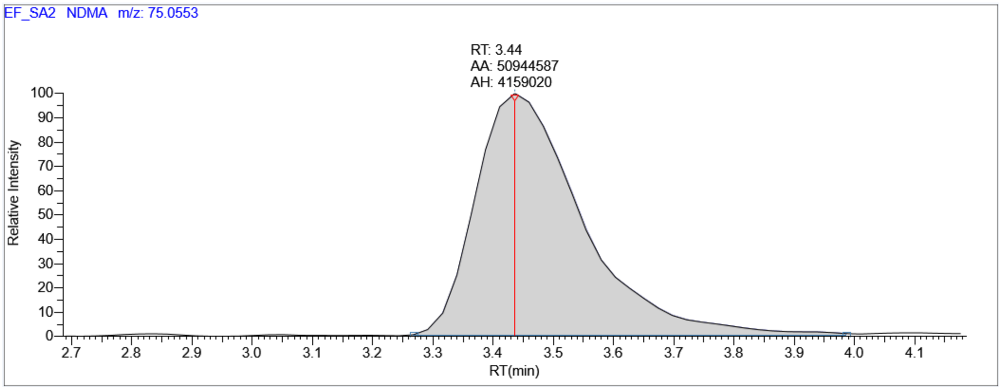

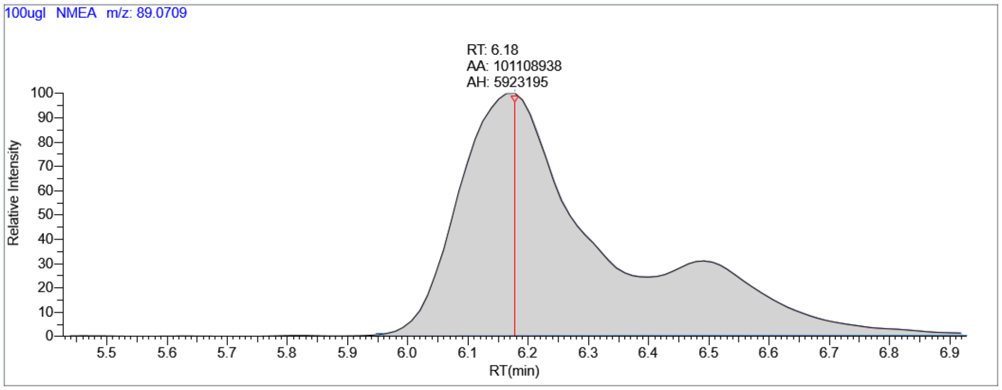

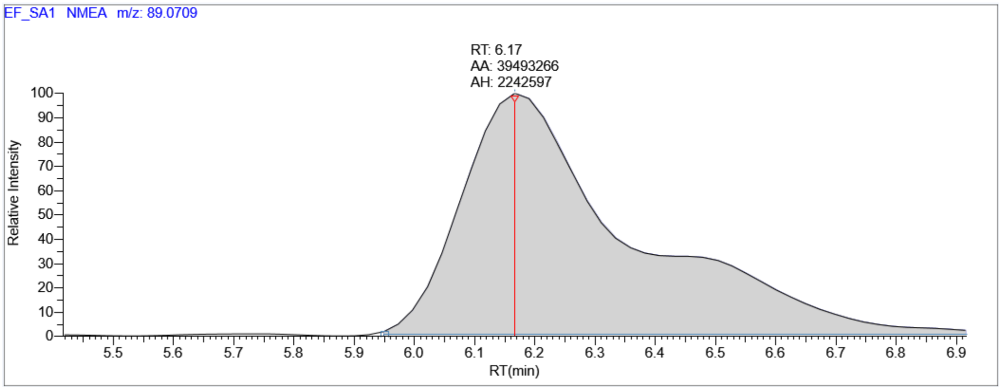

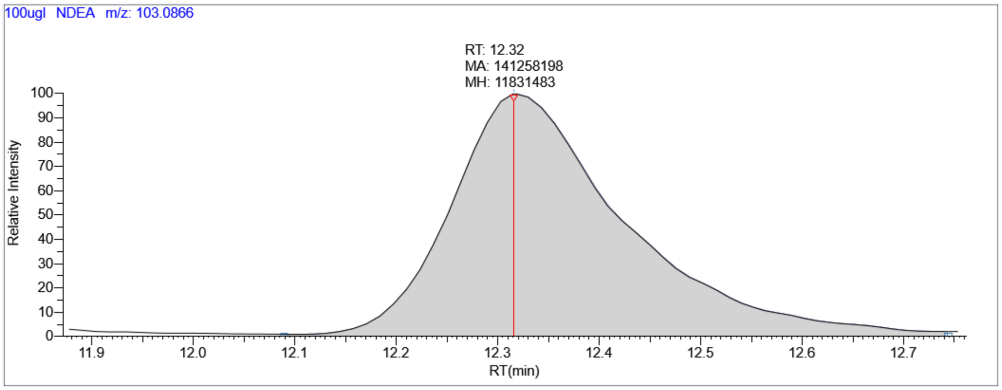
100ugl NDEA m/z: 103.0866
RT: 12.32
MA: 141258198
MH: 11831483
Relative Intensity
RT(min)

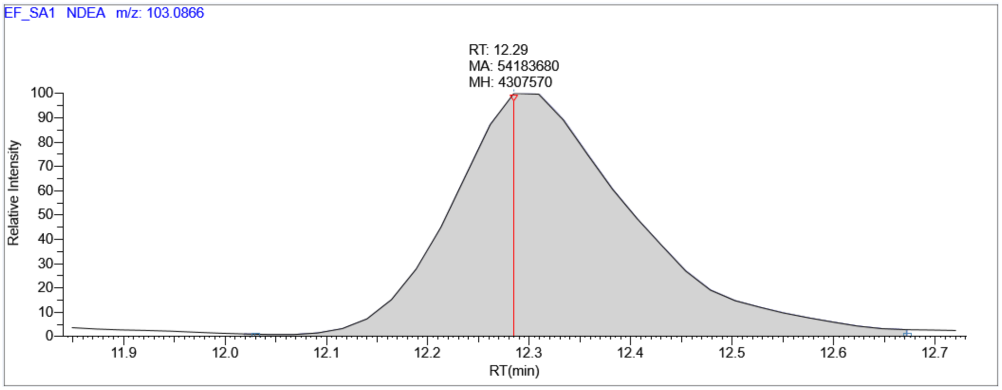
EF_SA1 NDEA m/z: 103.0866
RT: 12.29
MA: 54183680
MH: 4307570
Relative Intensity
RT(min)

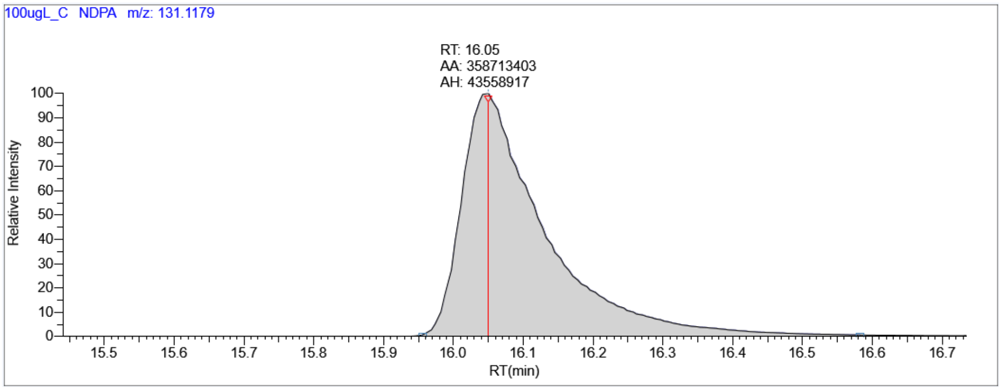
100ugL_C NDPA m/z: 131.1179
RT: 16.05
AA: 358713403
AH: 43558917
Relative Intensity
RT(min)

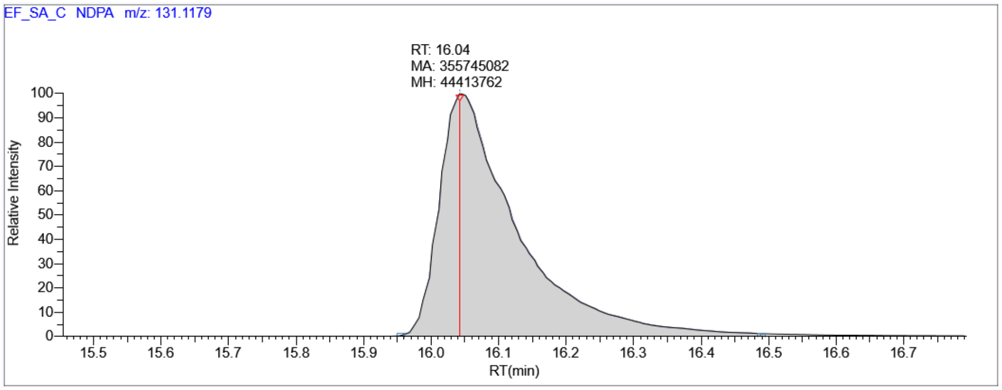
EF_SA_C NDPA m/z: 131.1179
RT: 16.04
MA: 355745082
MH: 44413762
Relative Intensity
RT(min)

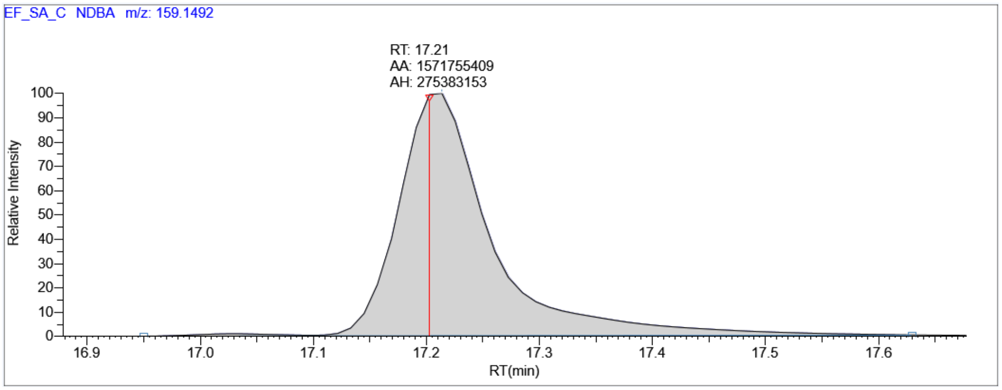
EF_SA_C NDBA m/z: 159.1492
RT: 17.21
AA: 1571755409
AH: 275383153
Relative Intensity
RT(min)

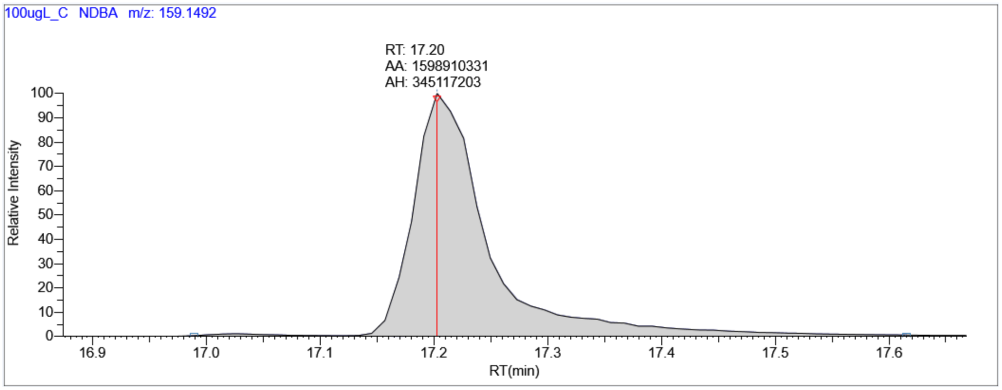
100ugL_C NDBA m/z: 159.1492
RT: 17.20
AA: 1598910331
AH: 345117203
Relative Intensity
RT(min)

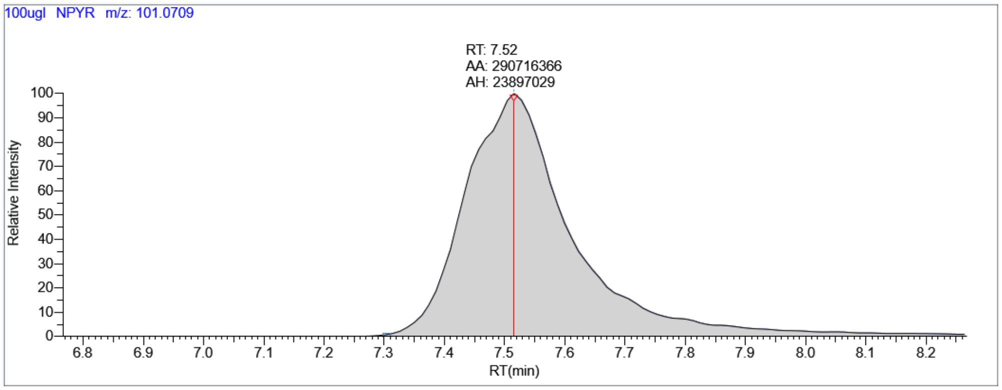
100ugl NPYR m/z: 101.0709
RT: 7.52
AA: 290716366
AH: 23897029
Relative Intensity
RT(min)

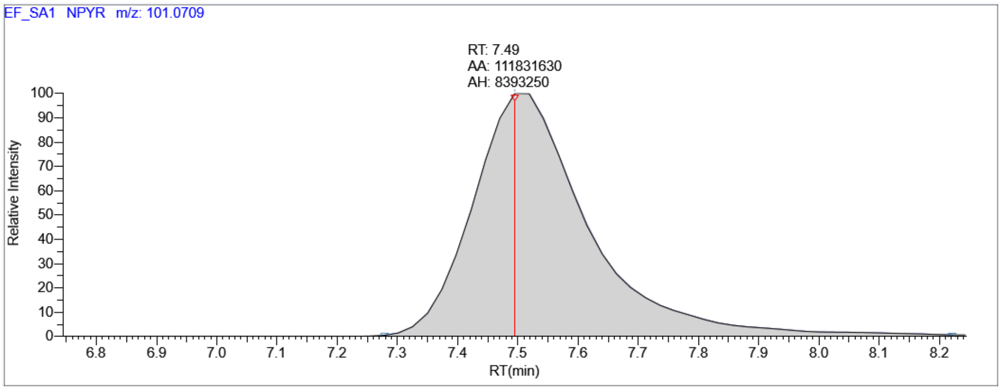
EF_SA1 NPYR m/z: 101.0709
RT: 7.49
AA: 111831630
AH: 8393250
Relative Intensity
RT(min)

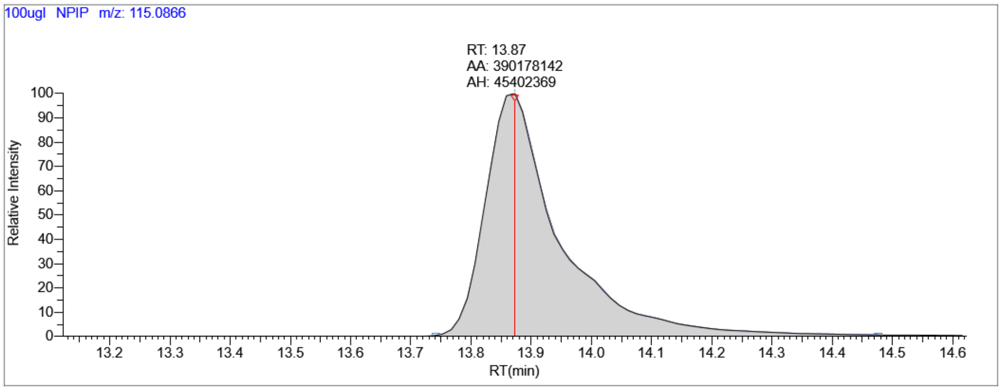
100ugl NPIP m/z: 115.0866
RT: 13.87
AA: 390178142
AH: 45402369
Relative Intensity
RT(min)

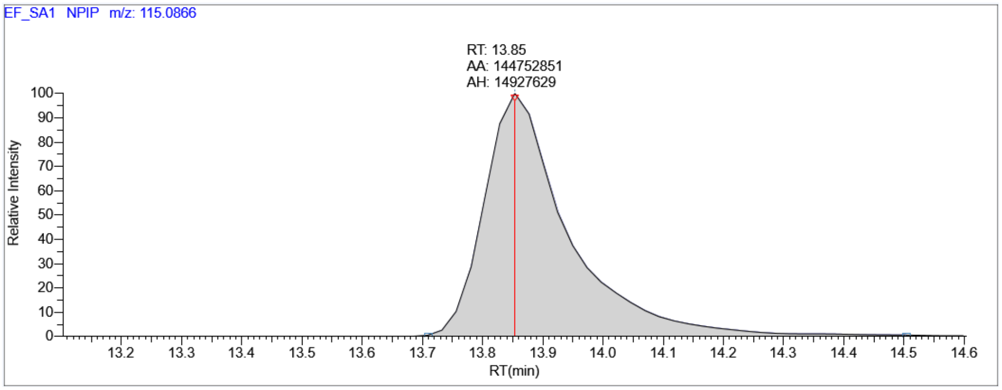
EF_SA1 NPIP m/z: 115.0866
RT: 13.85
AA: 144752851
AH: 14927629
Relative Intensity
RT(min)

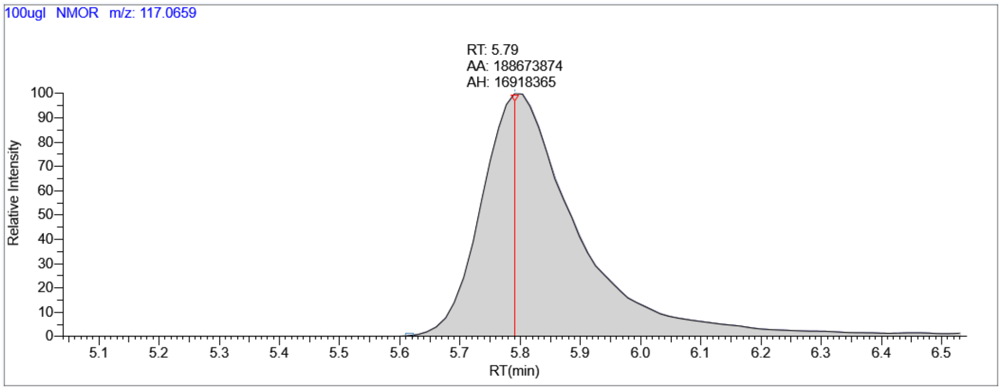
100ugl NMOR m/z: 117.0659
RT: 5.79
AA: 188673874
AH: 16918365
Relative Intensity
RT(min)

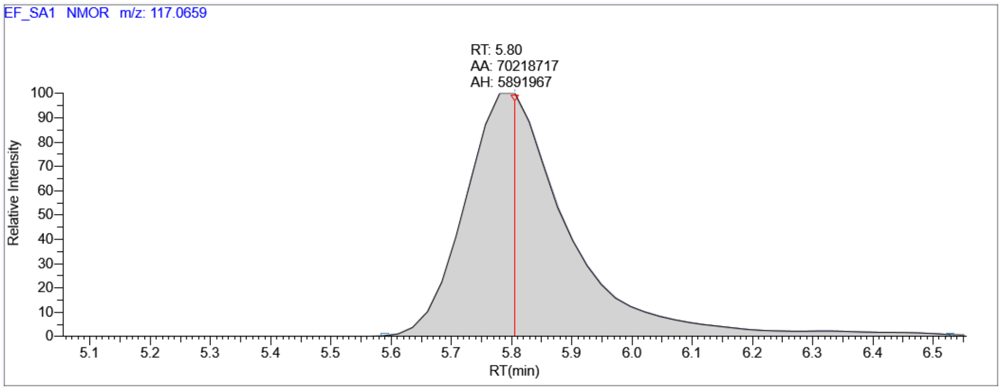
EF_SA1 NMOR m/z: 117.0659
RT: 5.80
AA: 70218717
AH: 5891967
Relative Intensity
RT(min)

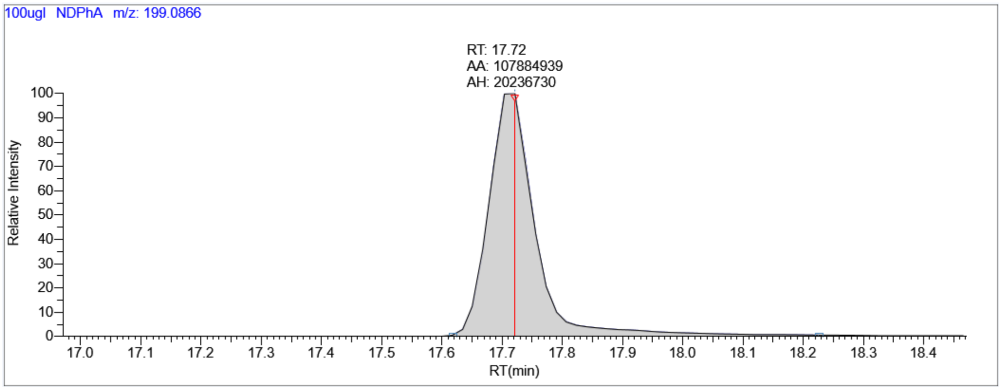
100ugl NDPhA m/z: 199.0866
RT: 17.72
AA: 107884939
AH: 20236730
Relative Intensity
RT(min)

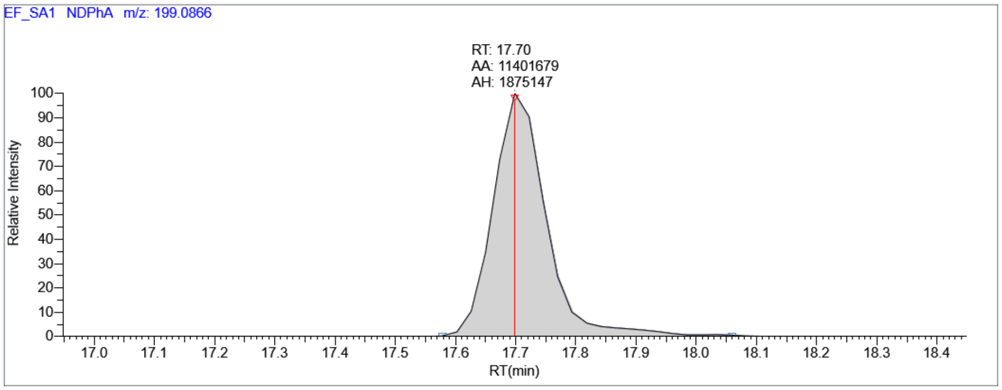
EF_SA1 NDPhA m/z: 199.0866
RT: 17.70
AA: 11401679
AH: 1875147
Relative Intensity
RT(min)

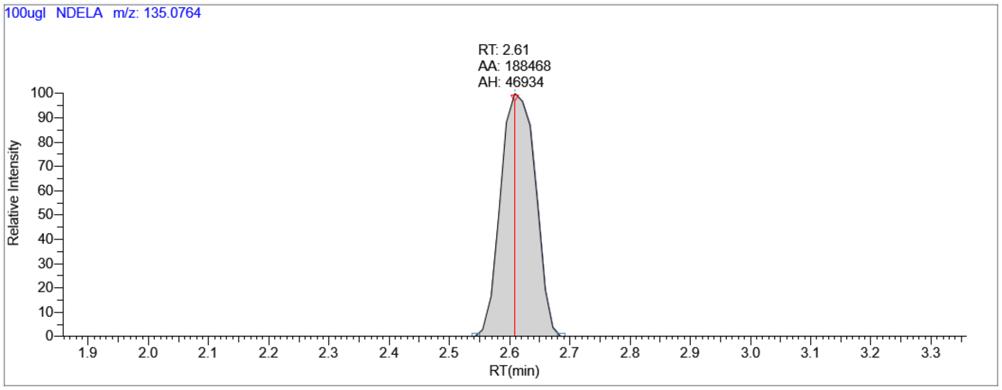
100ugl NDELA m/z: 135.0764
RT: 2.61
AA: 188468
AH: 46934
Relative Intensity
RT(min)

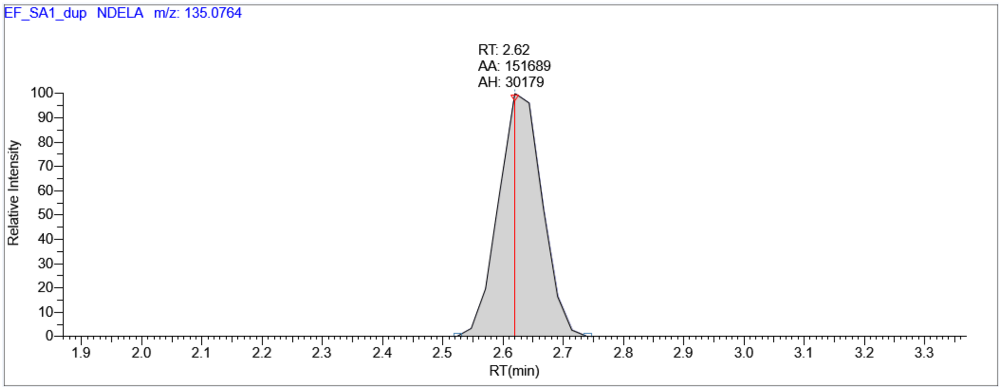
EF_SA1_dup NDELA m/z: 135.0764
RT: 2.62
AA: 151689
AH: 30179
Relative Intensity
RT(min)

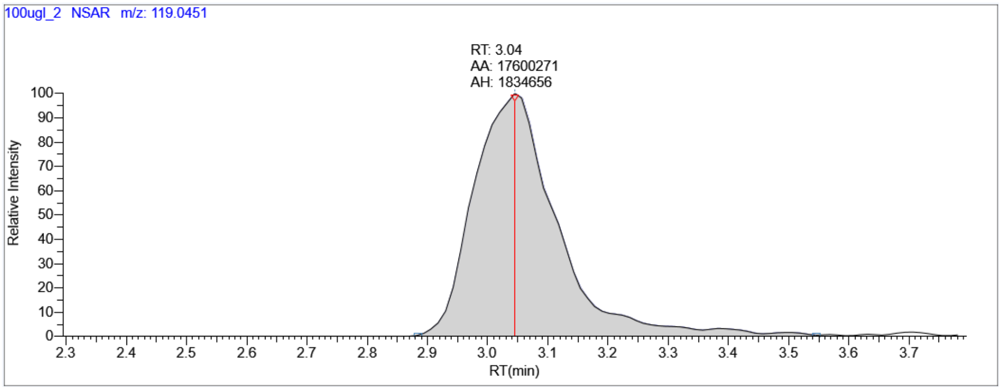
100ugl_2 NSAR m/z: 119.0451
RT: 3.04
AA: 17600271
AH: 1834656
Relative Intensity
RT(min)

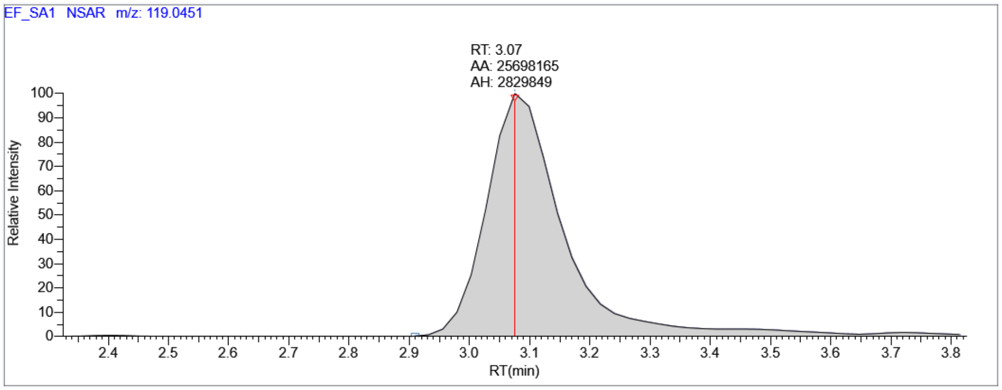
EF_SA1 NSAR m/z: 119.0451
RT: 3.07
AA: 25698165
AH: 2829849
Relative Intensity
RT(min)

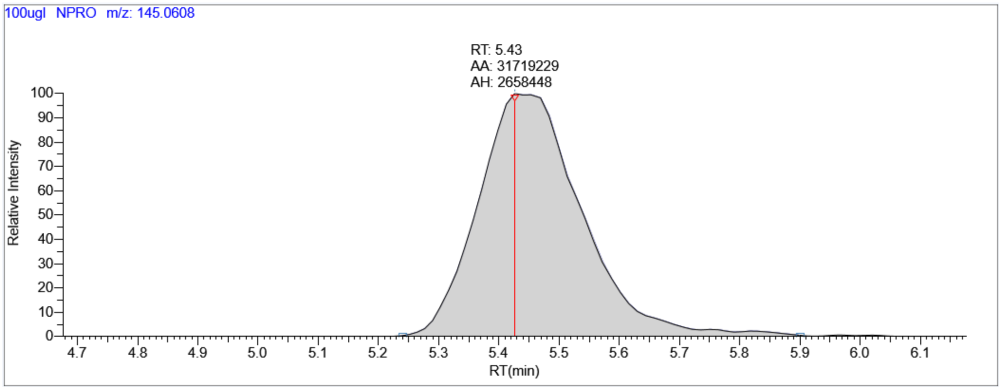
100ugl NPRO m/z: 145.0608
RT: 5.43
AA: 31719229
AH: 2658448
Relative Intensity
RT(min)

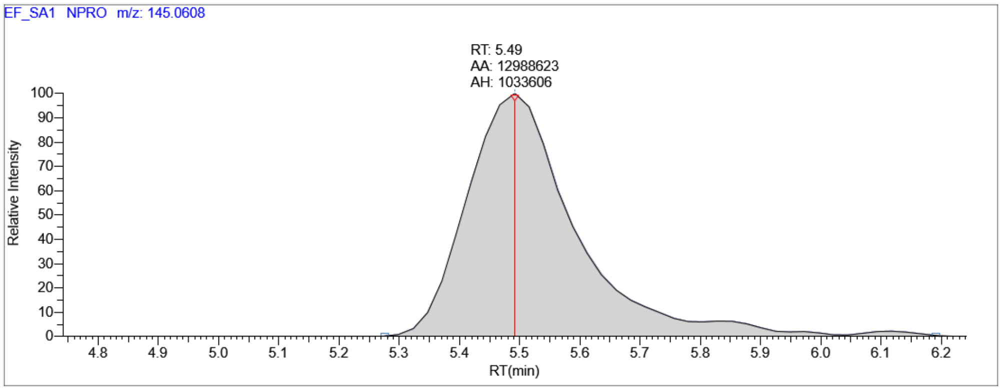
EF_SA1 NPRO m/z: 145.0608
RT: 5.49
AA: 12988623
AH: 1033606
Relative Intensity
RT(min)

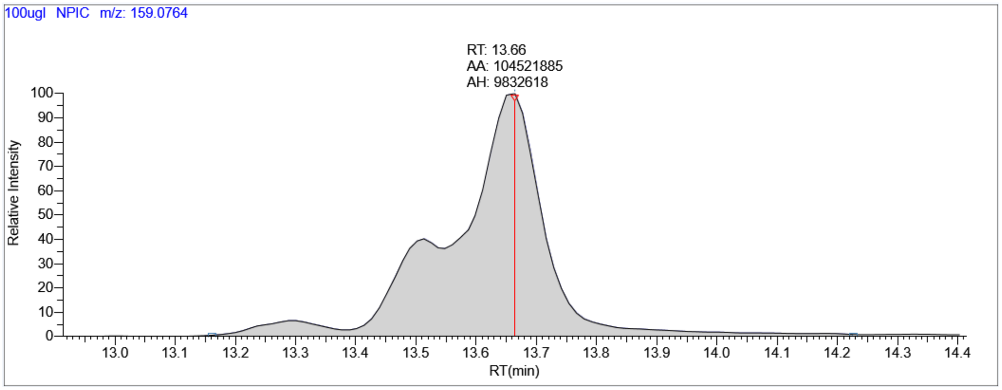
100ugl NPIC m/z: 159.0764
RT: 13.66
AA: 104521885
AH: 9832618
Relative Intensity
RT(min)

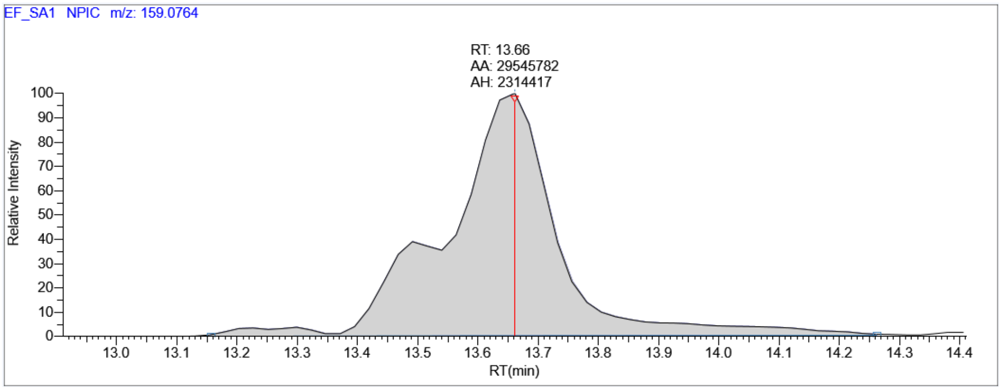
EF_SA1 NPIC m/z: 159.0764
RT: 13.66
AA: 29545782
AH: 2314417
Relative Intensity
RT(min)

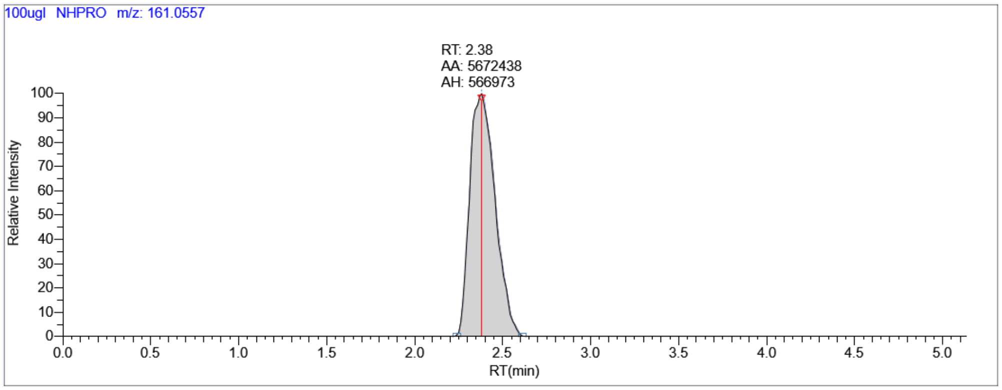
100ugl NHPRO m/z: 161.0557
RT: 2.38
AA: 5672438
AH: 566973
Relative Intensity
RT(min)

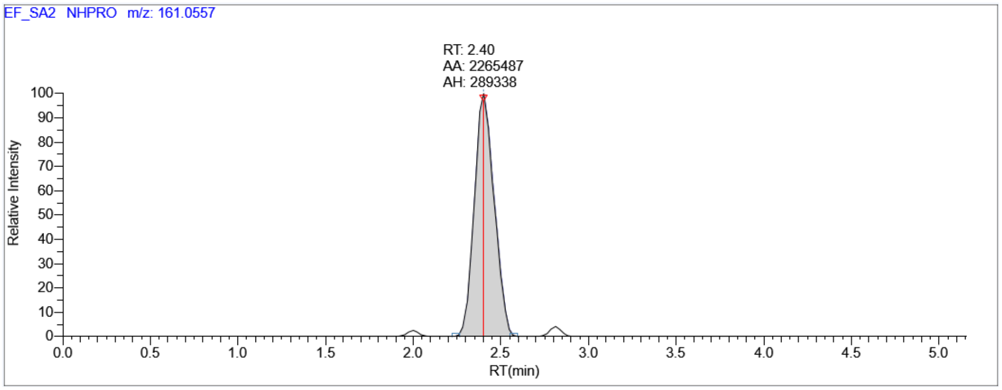
EF_SA2 NHPRO m/z: 161.0557
RT: 2.40
AA: 2265487
AH: 289338
Relative Intensity
RT(min)

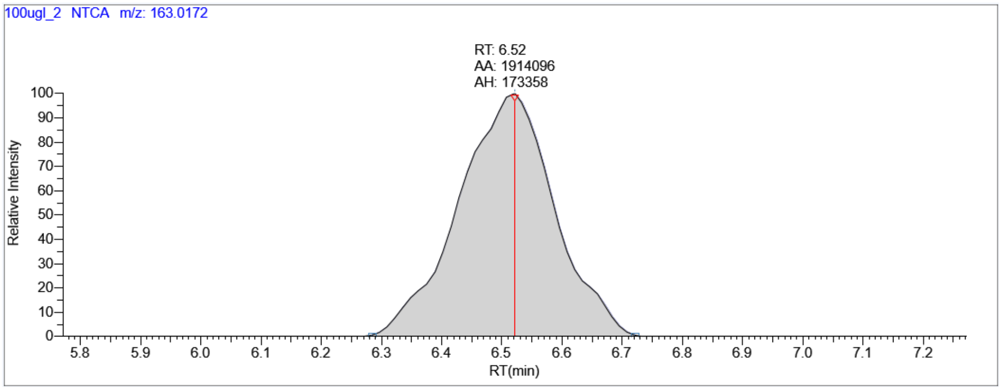

100ugl_2 NTCA m/z: 163.0172
RT: 6.52
AA: 1914096
AH: 173358
Relative Intensity
RT(min)

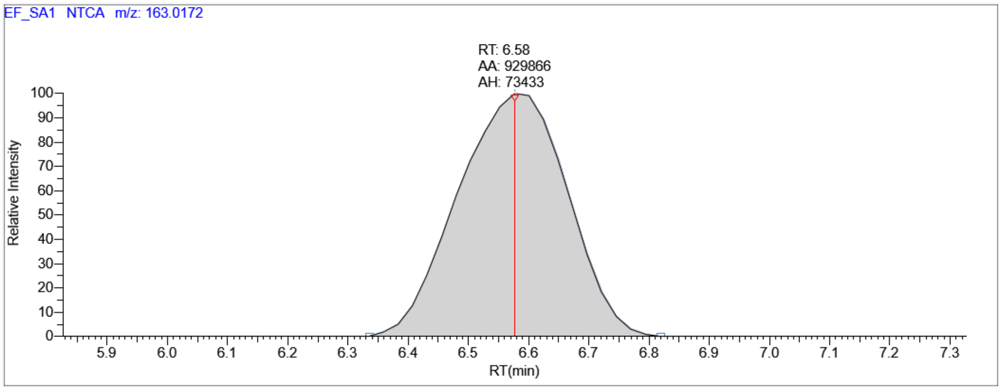

EF_SA1 NTCA m/z: 163.0172
RT: 6.58
AA: 929866
AH: 73433
Relative Intensity
RT(min)

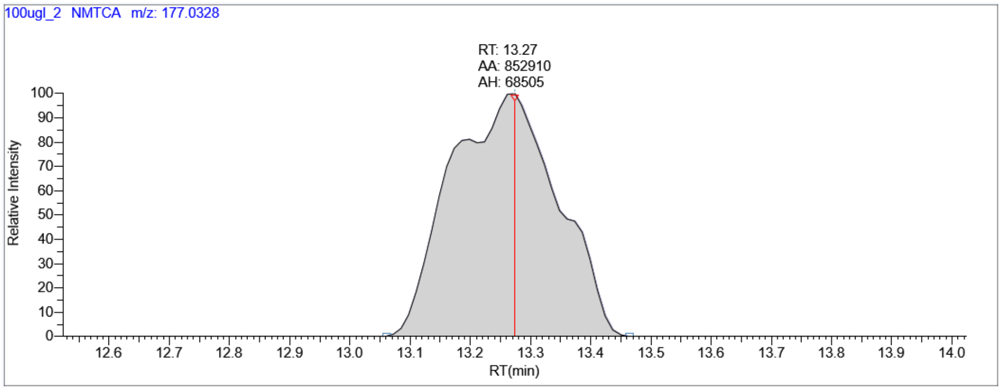

100ugl_2 NMTCA m/z: 177.0328
RT: 13.27
AA: 852910
AH: 68505
Relative Intensity
RT(min)

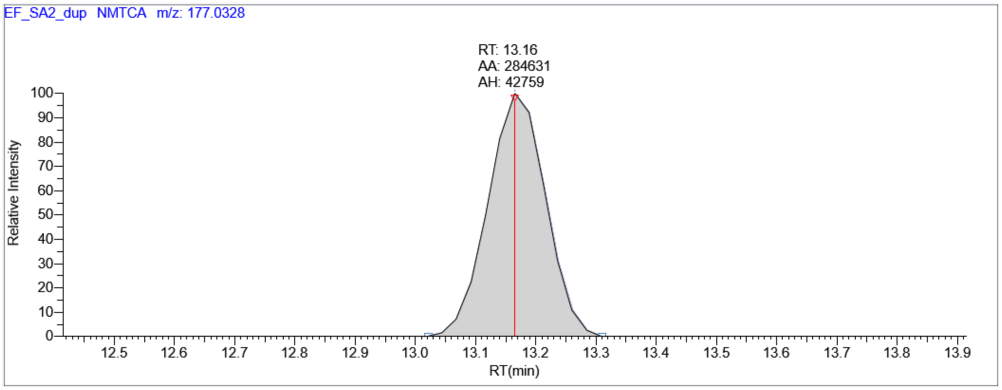

EF_SA2_dup NMTCA m/z: 177.0328
RT: 13.16
AA: 284631
AH: 42759
Relative Intensity
RT(min)

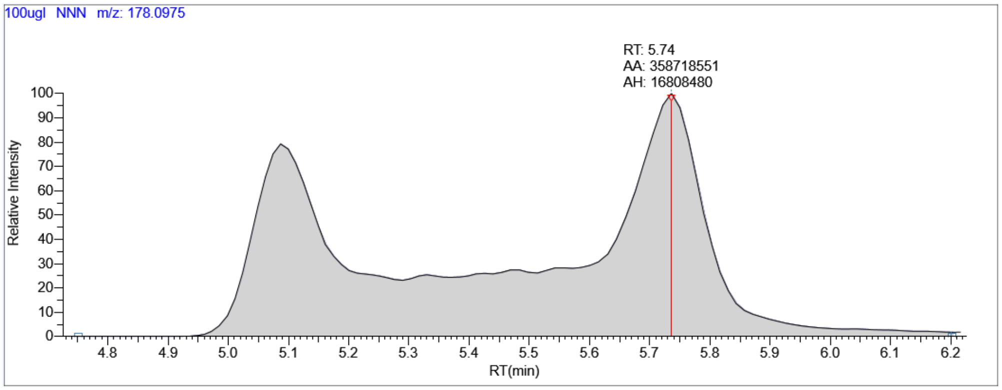
100ugl NNN m/z: 178.0975
RT: 5.74
AA: 358718551
AH: 16808480
Relative Intensity
RT(min)

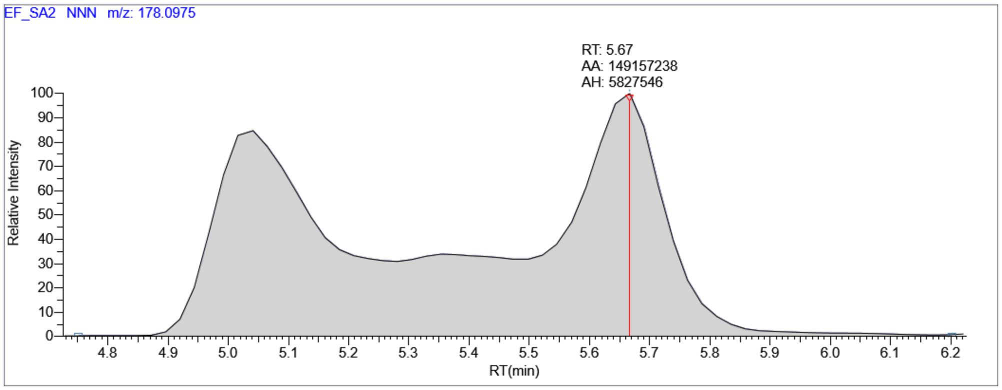
EF_SA2 NNN m/z: 178.0975
RT: 5.67
AA: 149157238
AH: 5827546
Relative Intensity
RT(min)

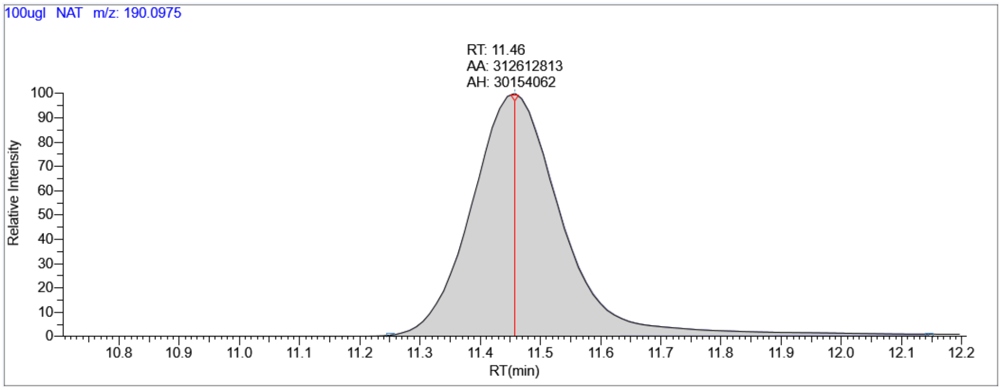
100ugl NAT m/z: 190.0975
RT: 11.46
AA: 312612813
AH: 30154062
Relative Intensity
RT(min)

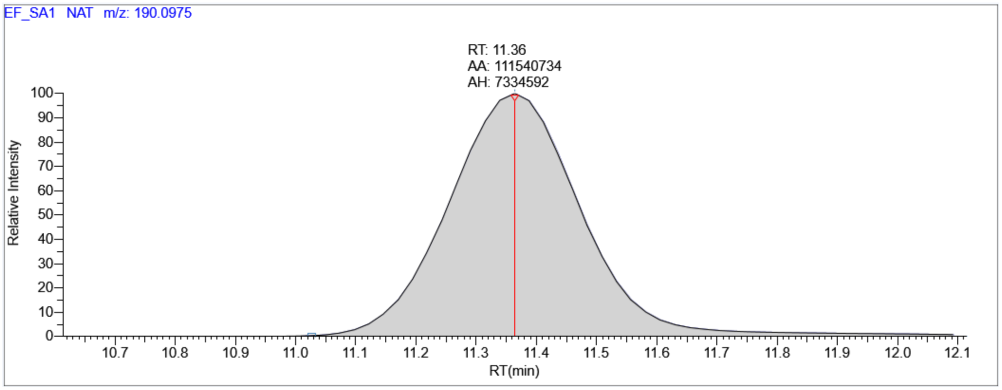
EF_SA1 NAT m/z: 190.0975
RT: 11.36
AA: 111540734
AH: 7334592
Relative Intensity
RT(min)

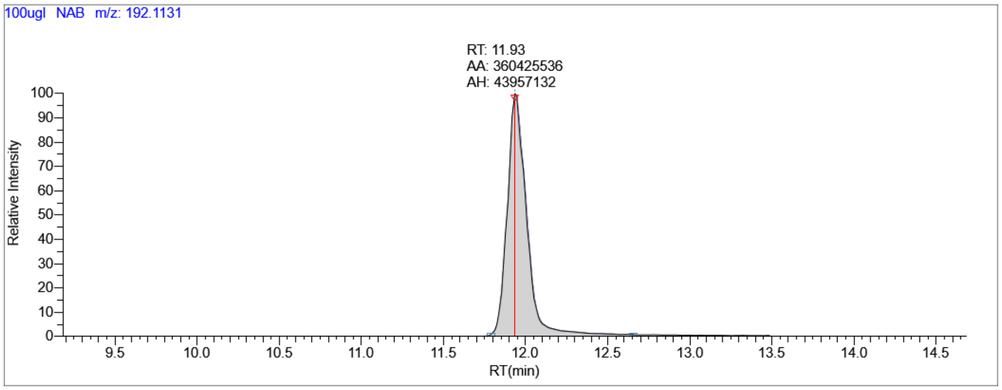
100ugl NAB m/z: 192.1131
RT: 11.93
AA: 360425536
AH: 43957132
Relative Intensity
RT(min)

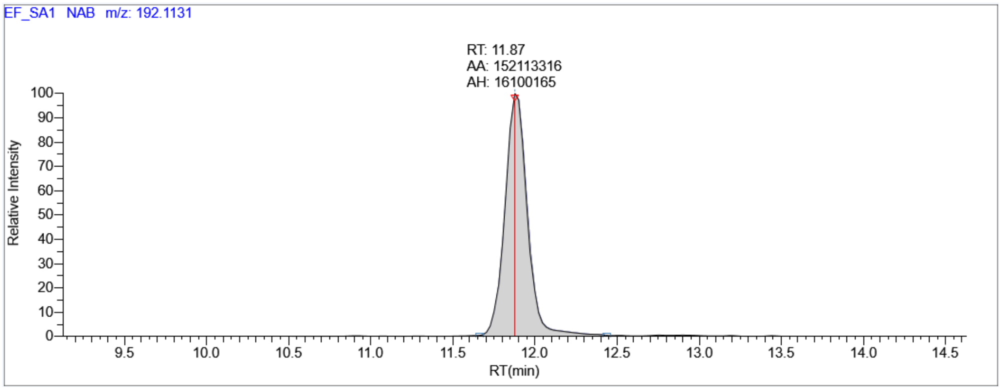
EF_SA1 NAB m/z: 192.1131
RT: 11.87
AA: 152113316
AH: 16100165
Relative Intensity
RT(min)

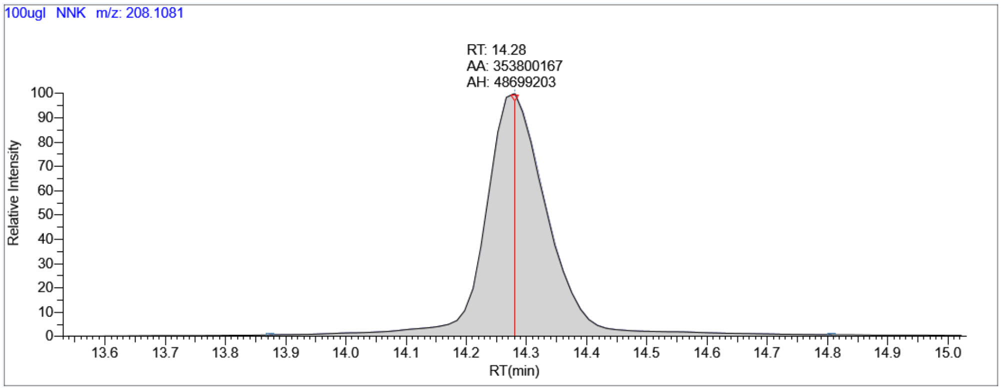
100ugl NNK m/z: 208.1081
RT: 14.28
AA: 353800167
AH: 48699203
Relative Intensity
RT(min)

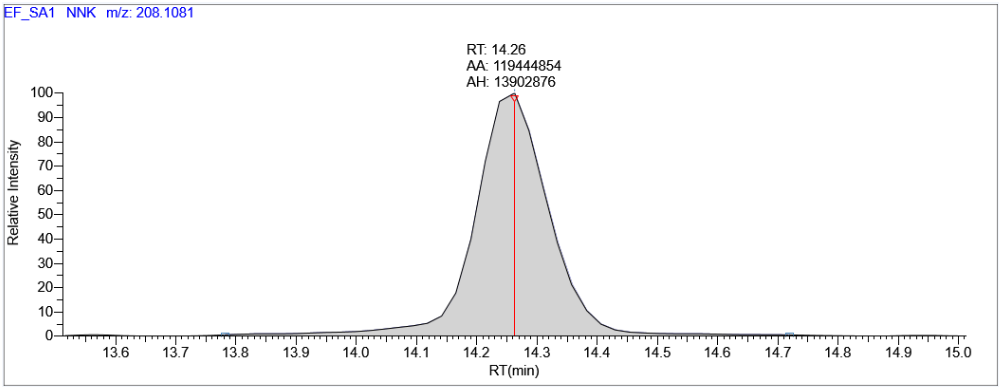
EF_SA1 NNK m/z: 208.1081
RT: 14.26
AA: 119444854
AH: 13902876
Relative Intensity
RT(min)

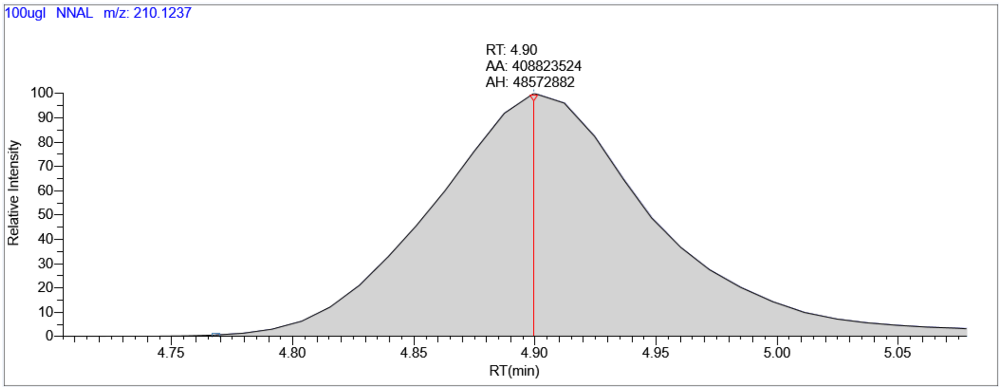
100ugl NNAL m/z: 210.1237
RT: 4.90
AA: 408823524
AH: 48572882
Relative Intensity
RT(min)

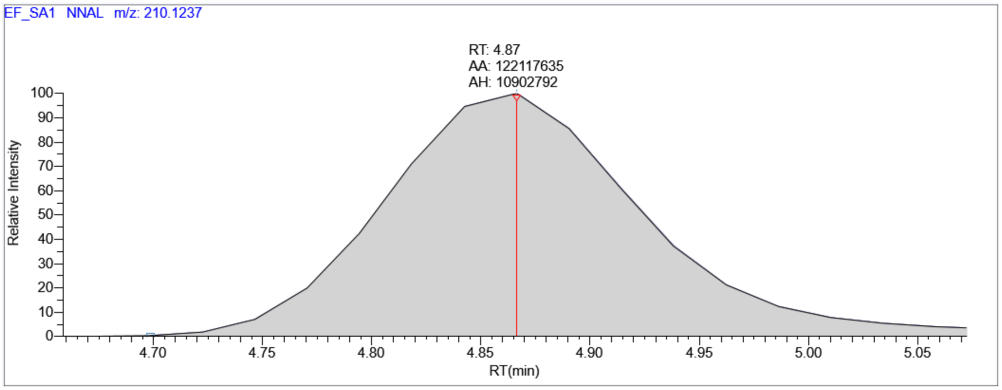
EF_SA1 NNAL m/z: 210.1237
RT: 4.87
AA: 122117635
AH: 10902792
Relative Intensity
RT(min)

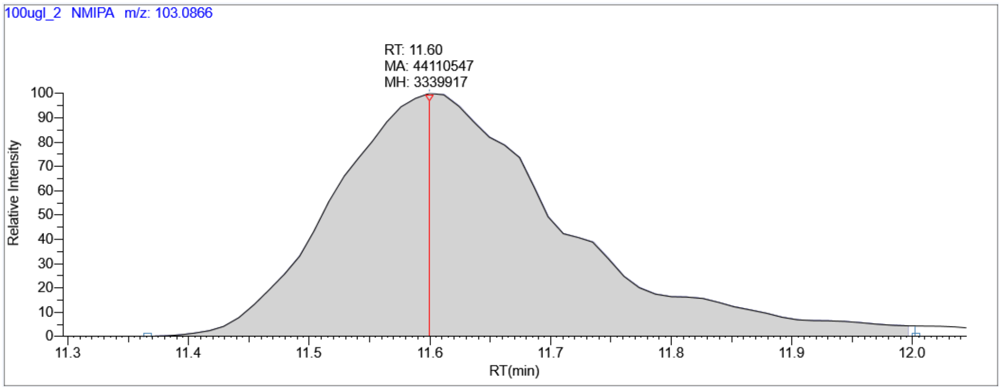
100ugl_2 NMIPA m/z: 103.0866
RT: 11.60
MA: 44110547
MH: 3339917
Relative Intensity
RT(min)

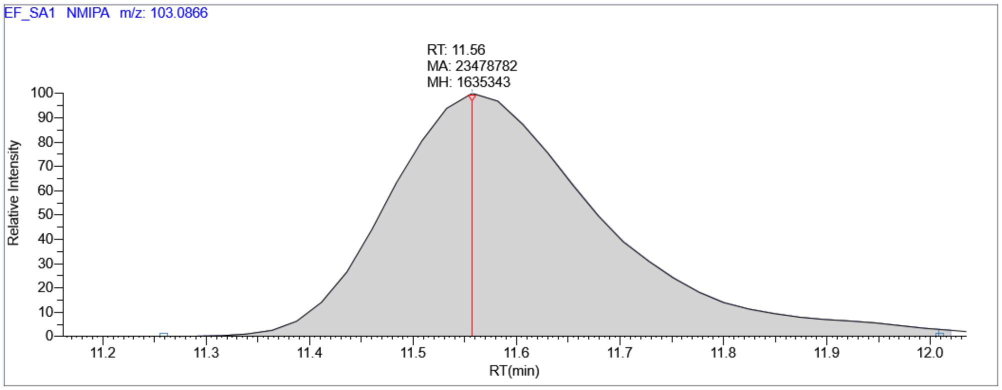
EF_SA1 NMIPA m/z: 103.0866
RT: 11.56
MA: 23478782
MH: 1635343
Relative Intensity
RT(min)

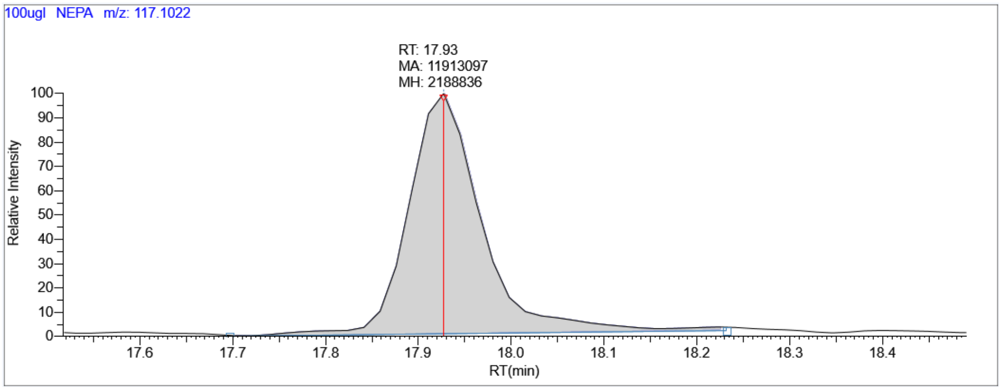
100ugl NEPA m/z: 117.1022
RT: 17.93
MA: 11913097
MH: 2188836
Relative Intensity
RT(min)

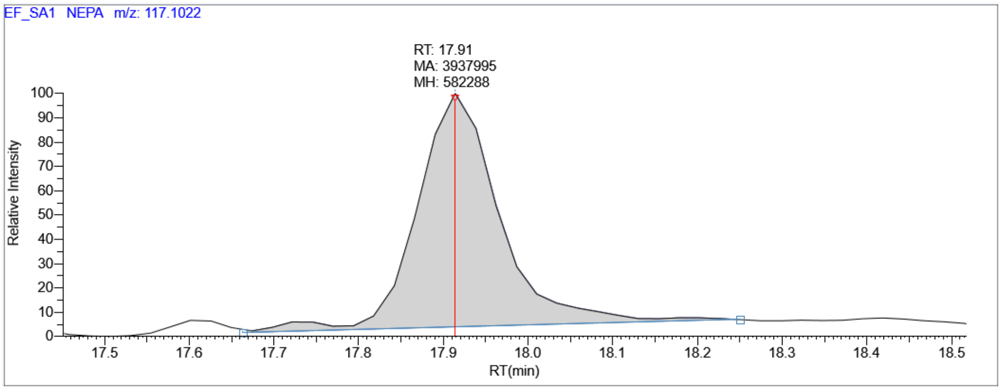
EF_SA1 NEPA m/z: 117.1022
RT: 17.91
MA: 3937995
MH: 582288
Relative Intensity
RT(min)

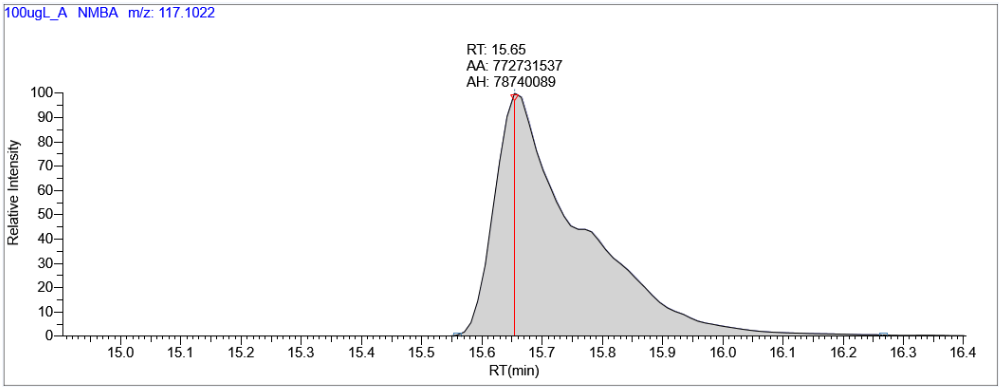
100ugL_A NMBA m/z: 117.1022
RT: 15.65
AA: 772731537
AH: 78740089
Relative Intensity
RT(min)

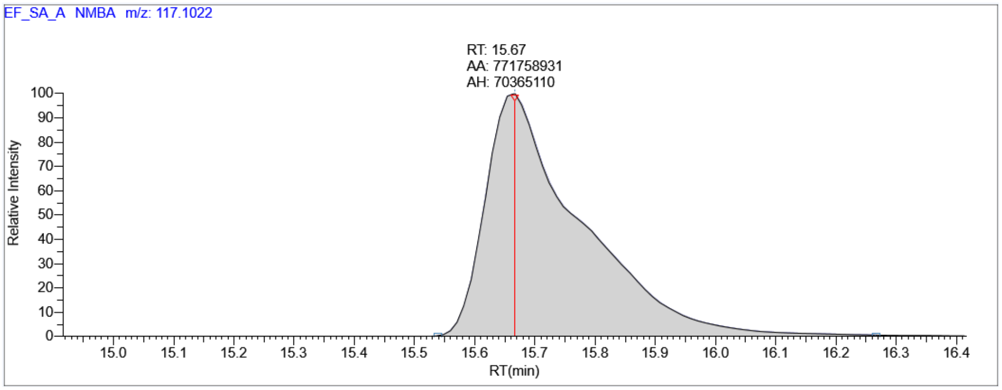
EF_SA_A NMBA m/z: 117.1022
RT: 15.67
AA: 771758931
AH: 70365110
Relative Intensity
RT(min)

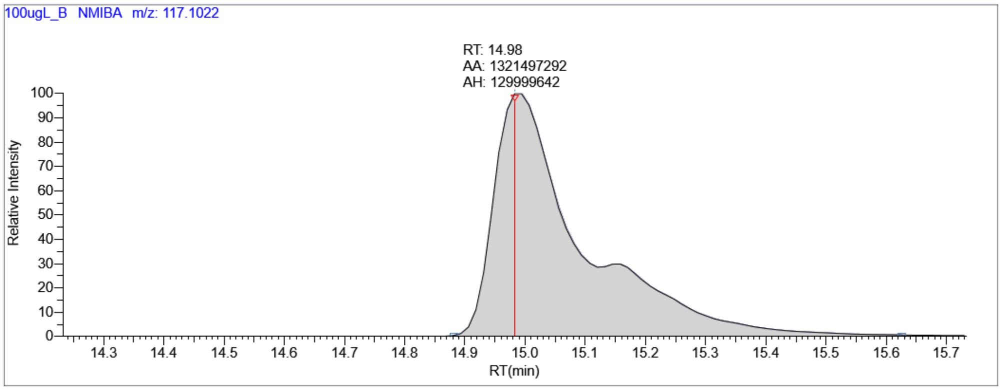

100ugL_B NMIBA m/z: 117.1022
RT: 14.98
AA: 1321497292
AH: 129999642
Relative Intensity
RT(min)

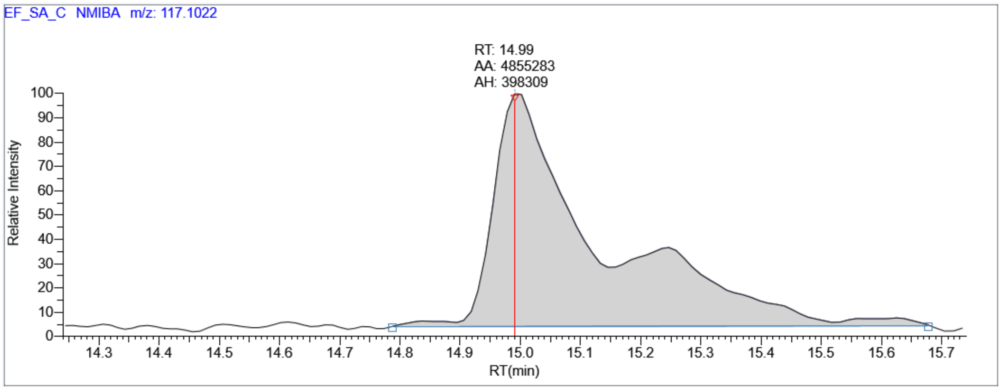

EF_SA_C NMIBA m/z: 117.1022
RT: 14.99
AA: 4855283
AH: 398309
Relative Intensity
RT(min)

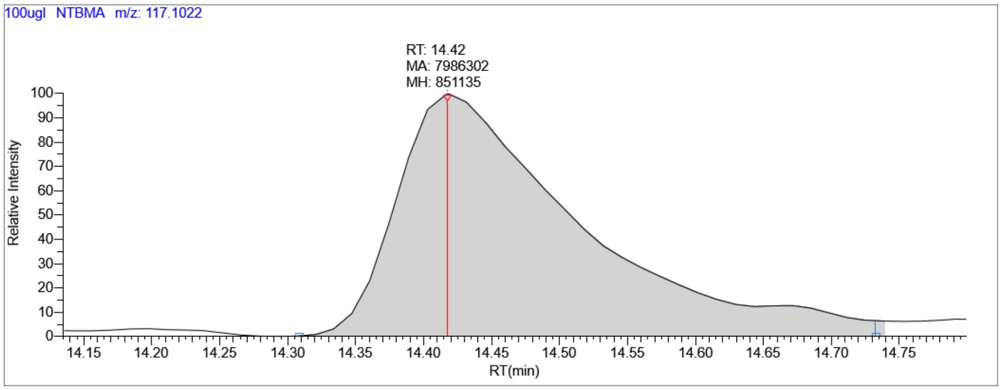

100ugl NTBMA m/z: 117.1022
RT: 14.42
MA: 7986302
MH: 851135
Relative Intensity
RT(min)

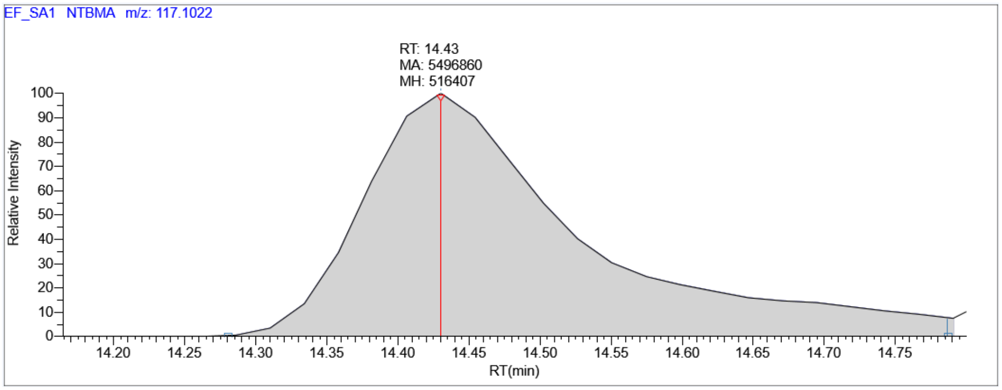

EF_SA1 NTBMA m/z: 117.1022
RT: 14.43
MA: 5496860
MH: 516407
Relative Intensity
RT(min)

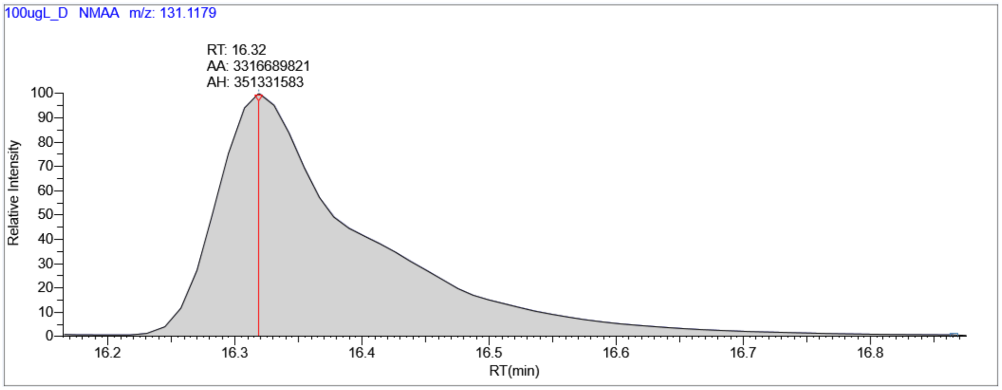
100ugL_D NMAA m/z: 131.1179
RT: 16.32
AA: 3316689821
AH: 351331583
Relative Intensity
RT(min)

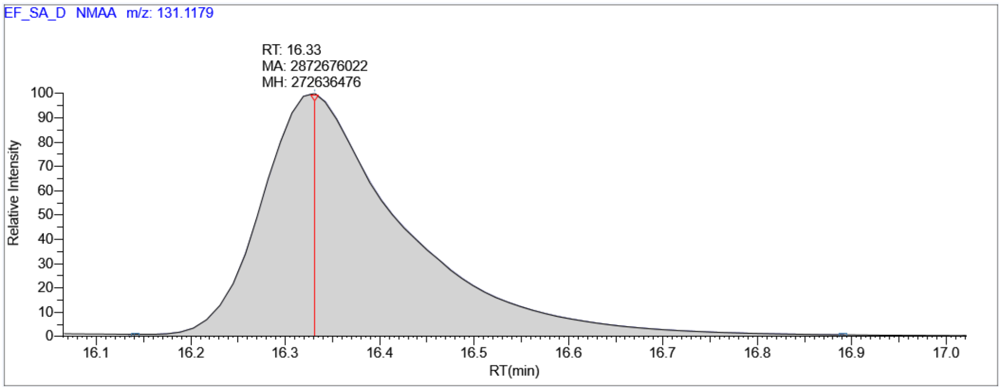
EF_SA_D NMAA m/z: 131.1179
RT: 16.33
MA: 2872676022
MH: 272636476
Relative Intensity
RT(min)

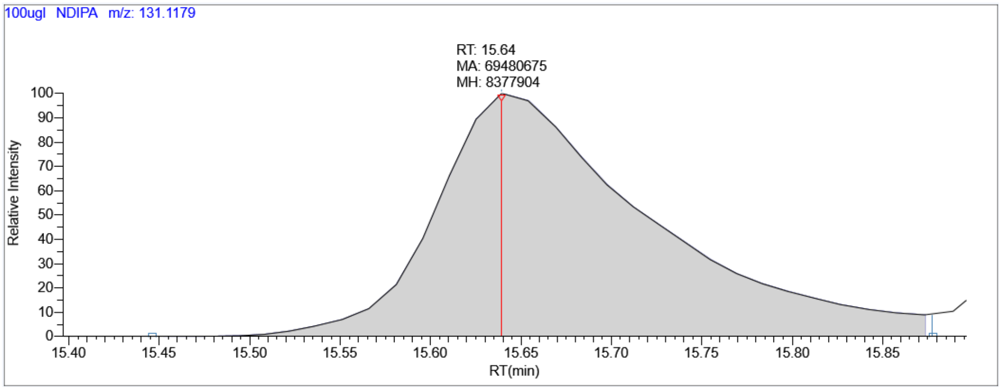
100ugl NDIPA m/z: 131.1179
RT: 15.64
MA: 69480675
MH: 8377904
Relative Intensity
RT(min)

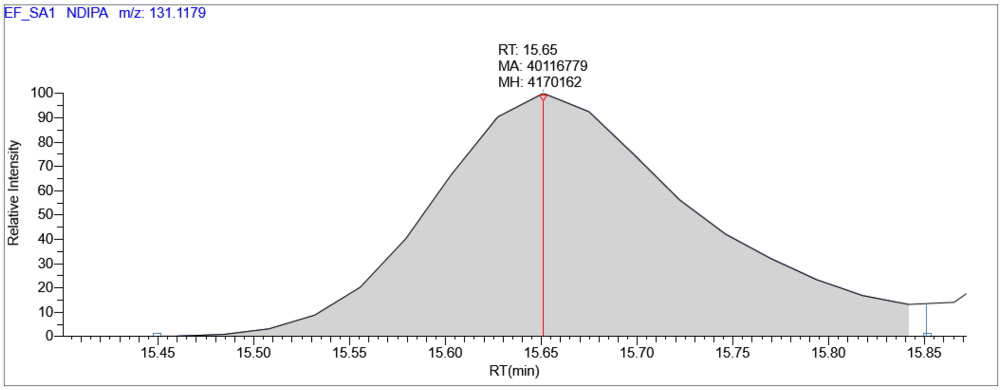
EF_SA1 NDIPA m/z: 131.1179
RT: 15.65
MA: 40116779
MH: 4170162
Relative Intensity
RT(min)

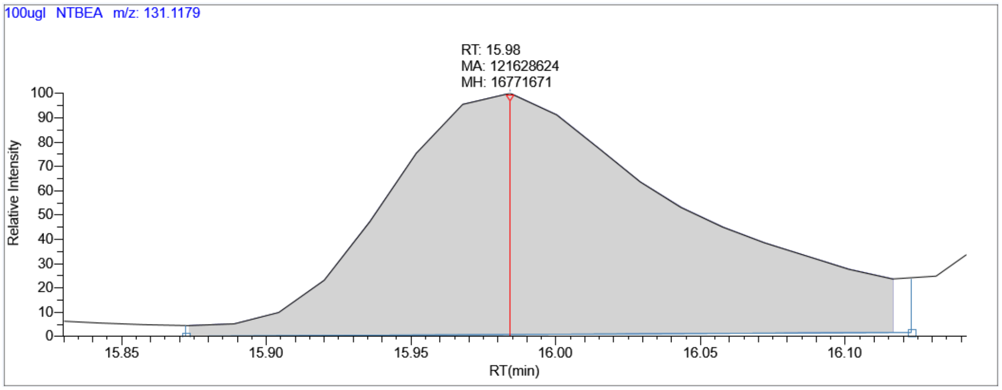
100ugl NTBEA m/z: 131.1179
RT: 15.98
MA: 121628624
MH: 16771671
Relative Intensity
RT(min)

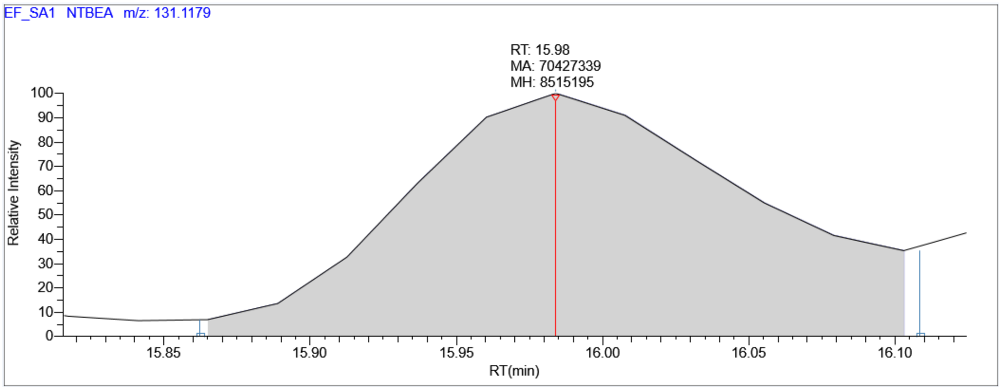
EF_SA1 NTBEA m/z: 131.1179
RT: 15.98
MA: 70427339
MH: 8515195
Relative Intensity
RT(min)

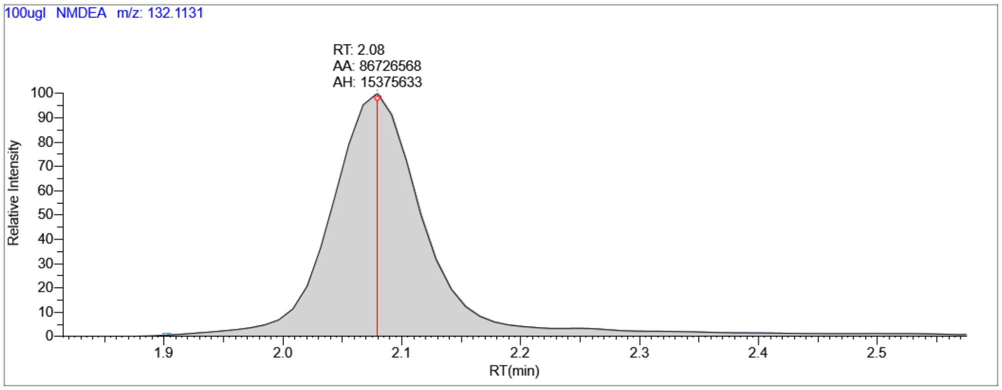
100ugl NMDEA m/z: 132.1131
RT: 2.08
AA: 86726568
AH: 15375633
Relative Intensity
RT(min)

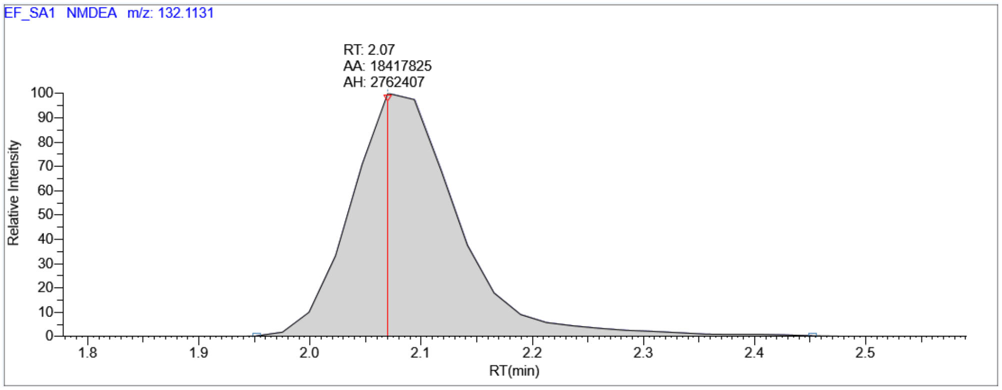
EF_SA1 NMDEA m/z: 132.1131
RT: 2.07
AA: 18417825
AH: 2762407
Relative Intensity
RT(min)

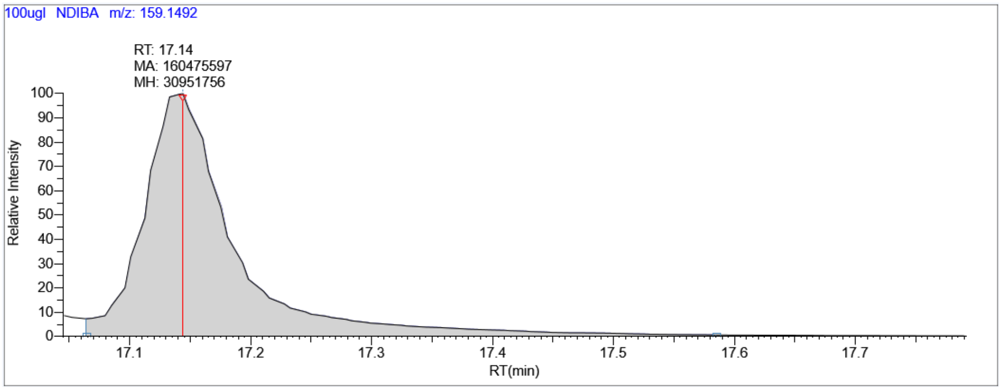

100ugl NDIBA m/z: 159.1492
RT: 17.14
MA: 160475597
MH: 30951756
Relative Intensity
RT(min)

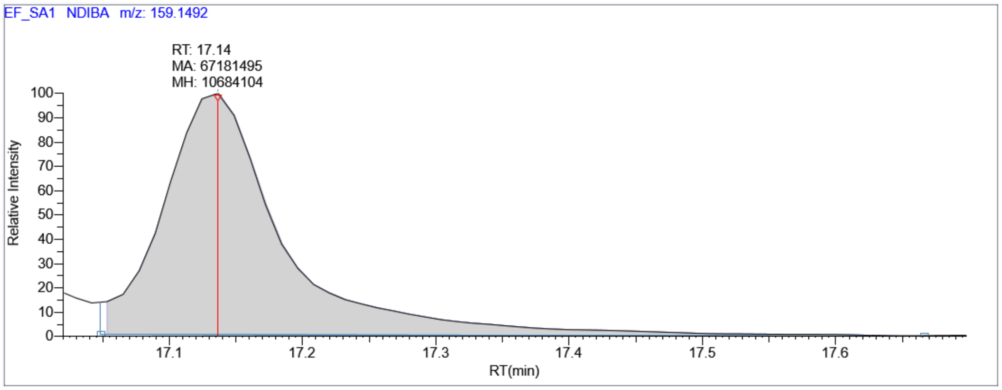

EF_SA1 NDIBA m/z: 159.1492
RT: 17.14
MA: 67181495
MH: 10684104
Relative Intensity
RT(min)

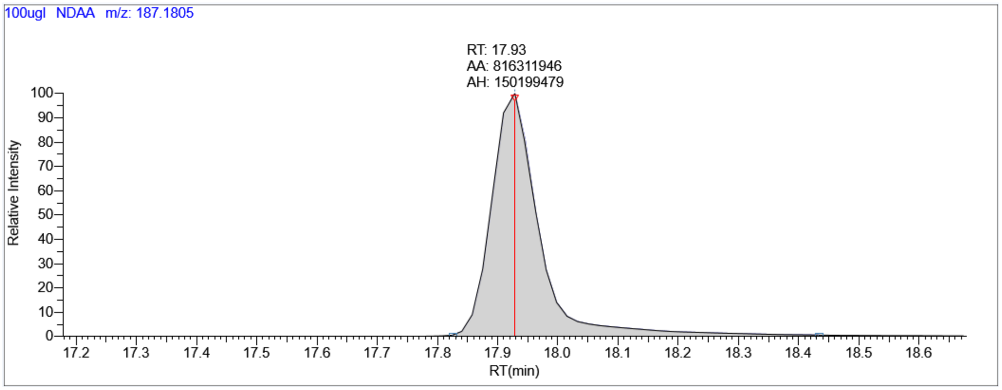

100ugl NDAA m/z: 187.1805
RT: 17.93
AA: 816311946
AH: 150199479
Relative Intensity
RT(min)

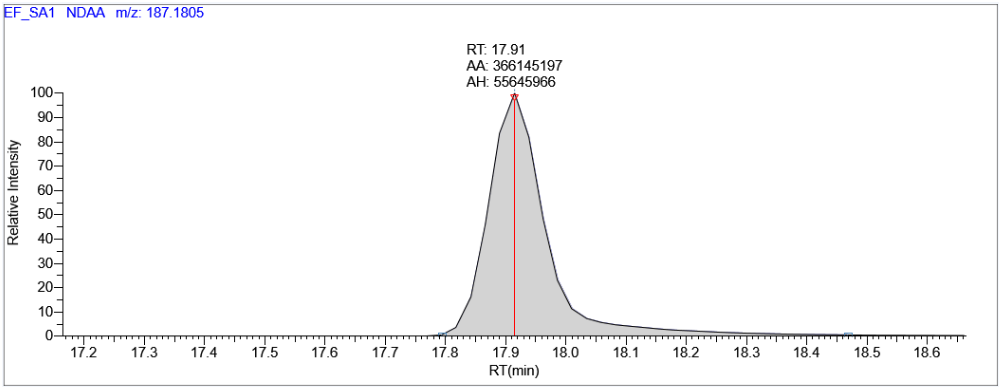

EF_SA1 NDAA m/z: 187.1805
RT: 17.91
AA: 366145197
AH: 55645966
Relative Intensity
RT(min)

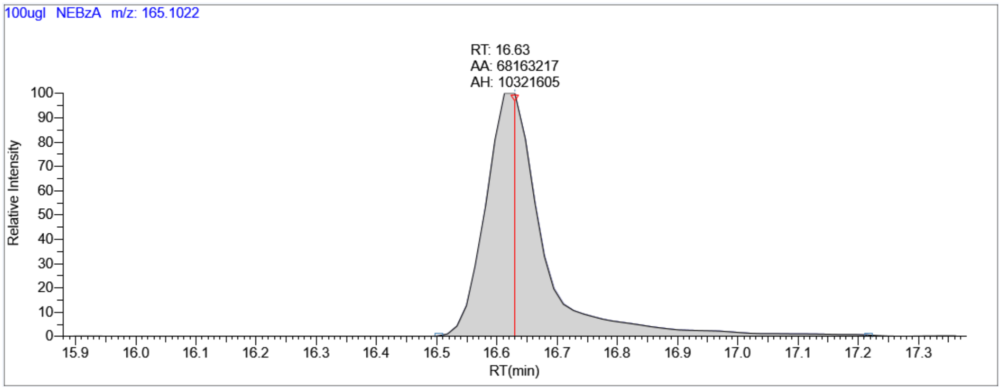
100ugl NEBzA m/z: 165.1022
RT: 16.63
AA: 68163217
AH: 10321605
Relative Intensity
RT(min)

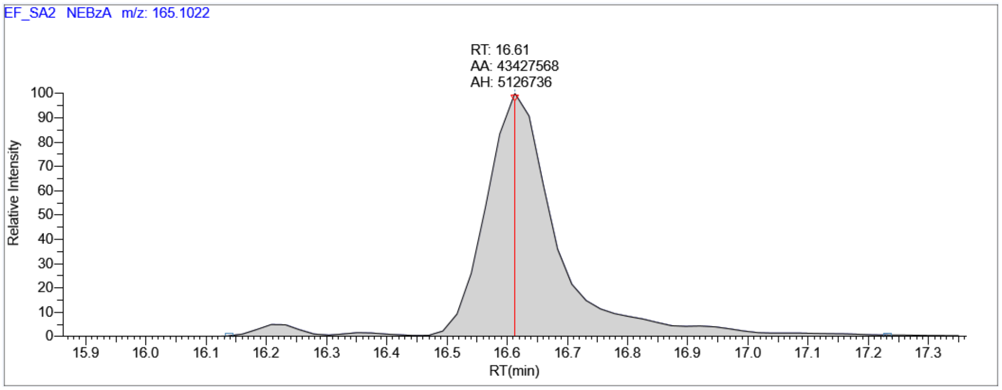
EF_SA2 NEBzA m/z: 165.1022
RT: 16.61
AA: 43427568
AH: 5126736
Relative Intensity
RT(min)

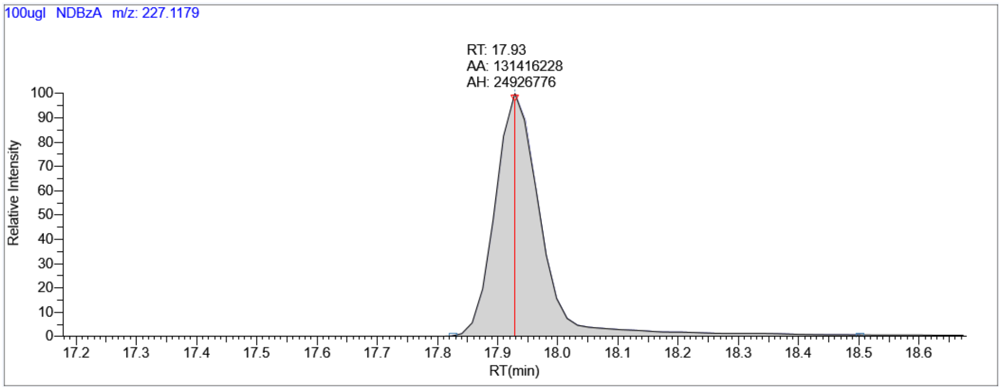
100ugl NDBzA m/z: 227.1179
RT: 17.93
AA: 131416228
AH: 24926776
Relative Intensity
RT(min)

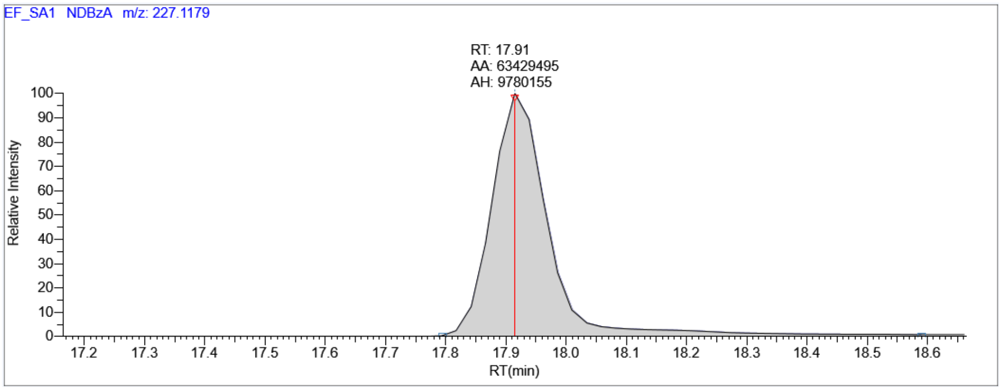
EF_SA1 NDBzA m/z: 227.1179
RT: 17.91
AA: 63429495
AH: 9780155
Relative Intensity
RT(min)

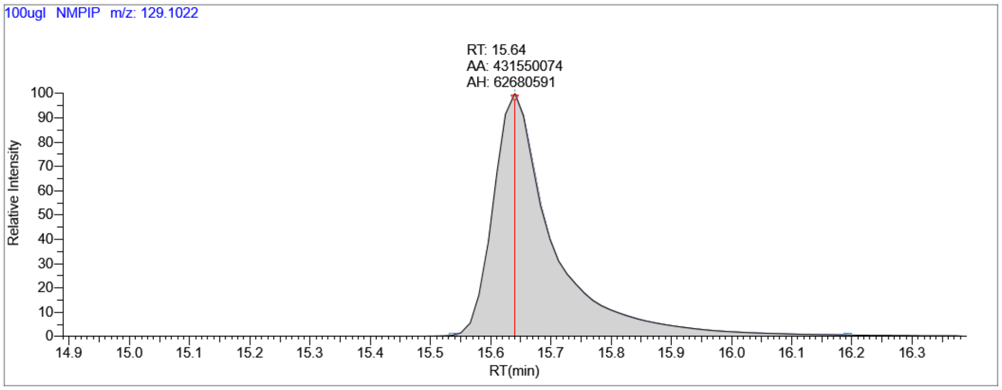
100ugl NMPIP m/z: 129.1022
RT: 15.64
AA: 431550074
AH: 62680591
Relative Intensity
RT(min)

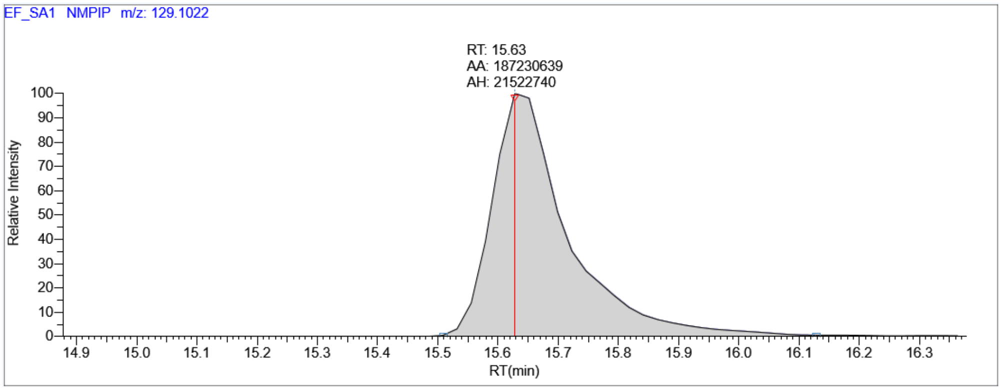
EF_SA1 NMPIP m/z: 129.1022
RT: 15.63
AA: 187230639
AH: 21522740
Relative Intensity
RT(min)

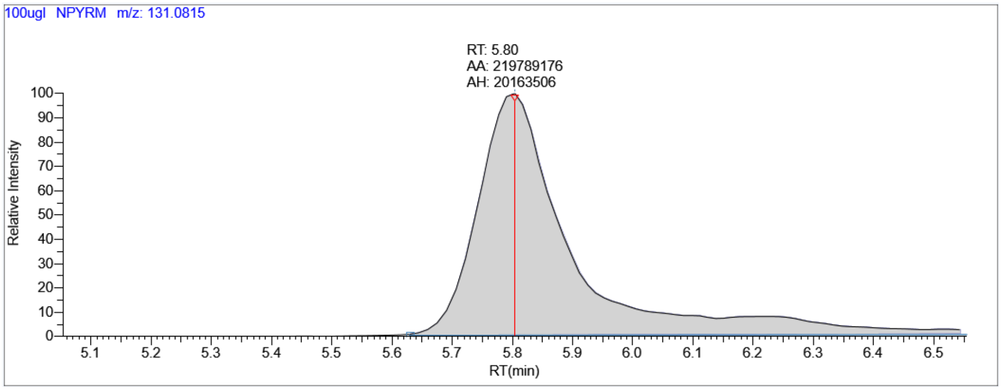
100ugl NPYRM m/z: 131.0815
RT: 5.80
AA: 219789176
AH: 20163506
Relative Intensity
RT(min)

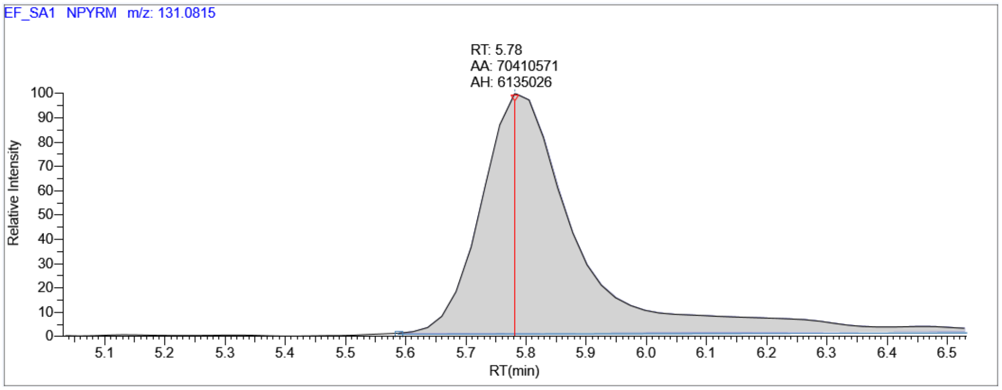
EF_SA1 NPYRM m/z: 131.0815
RT: 5.78
AA: 70410571
AH: 6135026
Relative Intensity
RT(min)

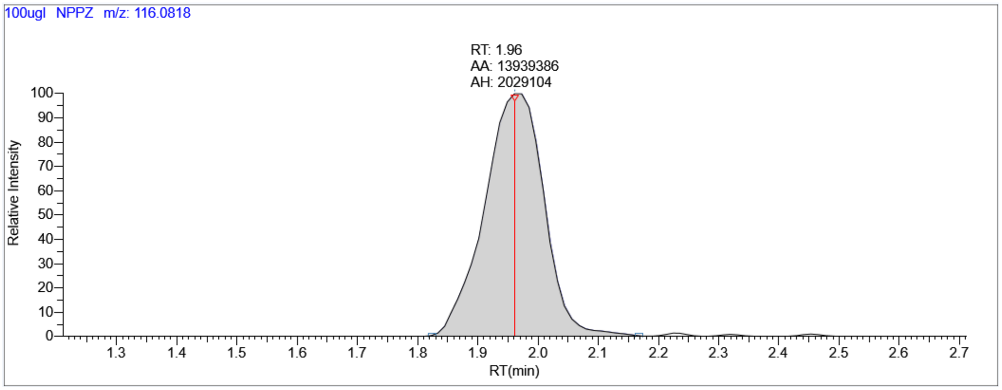
100ugl NPPZ m/z: 116.0818
RT: 1.96
AA: 13939386
AH: 2029104
Relative Intensity
RT(min)

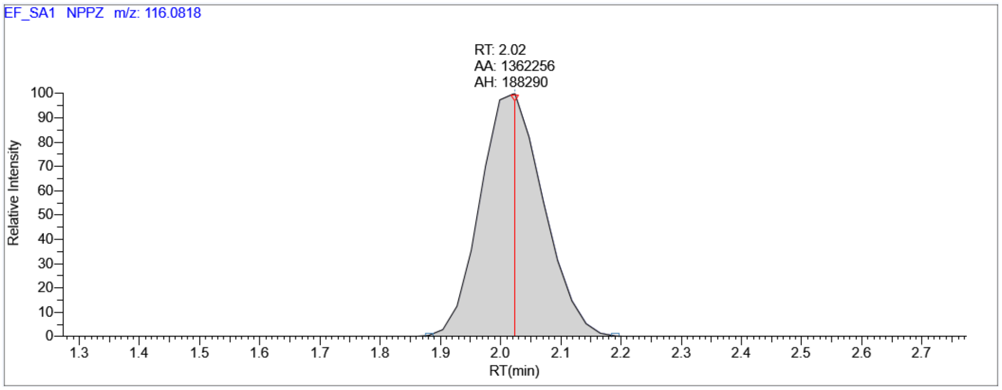
EF_SA1 NPPZ m/z: 116.0818
RT: 2.02
AA: 1362256
AH: 188290
Relative Intensity
RT(min)

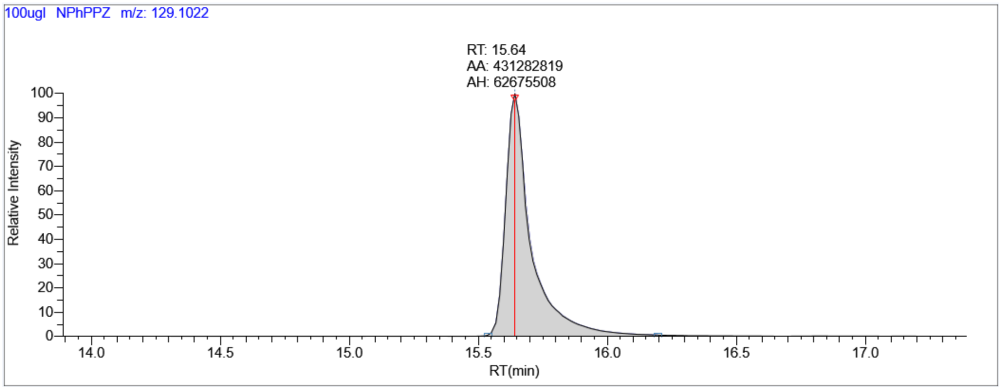
100ugl NPhPPZ m/z: 129.1022
RT: 15.64
AA: 431282819
AH: 62675508
Relative Intensity
RT(min)

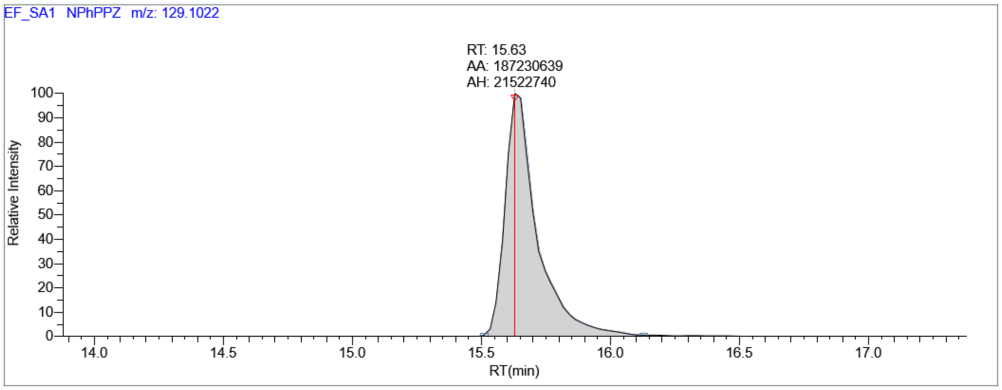
EF_SA1 NPhPPZ m/z: 129.1022
RT: 15.63
AA: 187230639
AH: 21522740
Relative Intensity
RT(min)

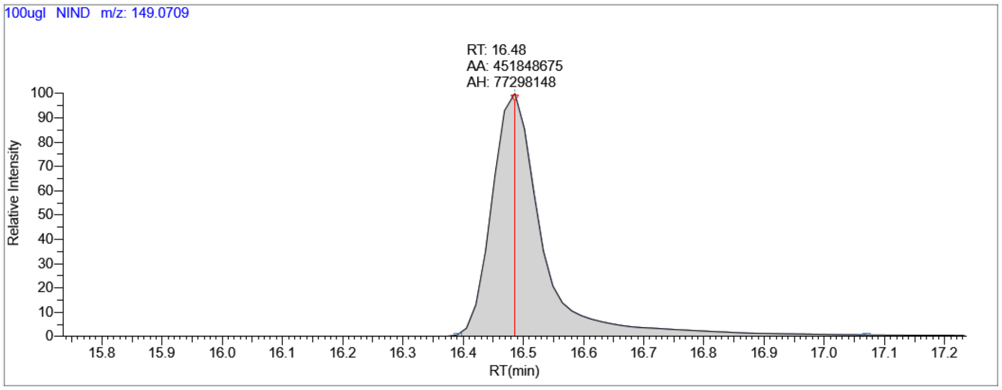
100ugl NIND m/z: 149.0709
RT: 16.48
AA: 451848675
AH: 77298148
Relative Intensity
RT(min)

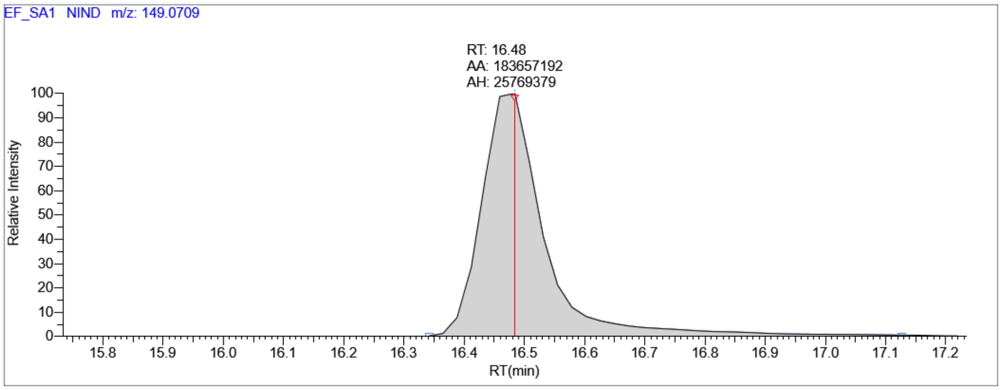
EF_SA1 NIND m/z: 149.0709
RT: 16.48
AA: 183657192
AH: 25769379
Relative Intensity
RT(min)

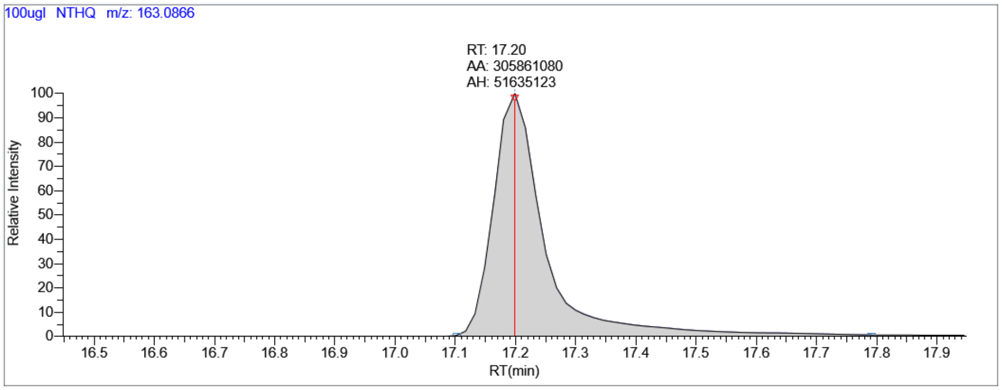
100ugl NTHQ m/z: 163.0866
RT: 17.20
AA: 305861080
AH: 51635123
Relative Intensity
RT(min)

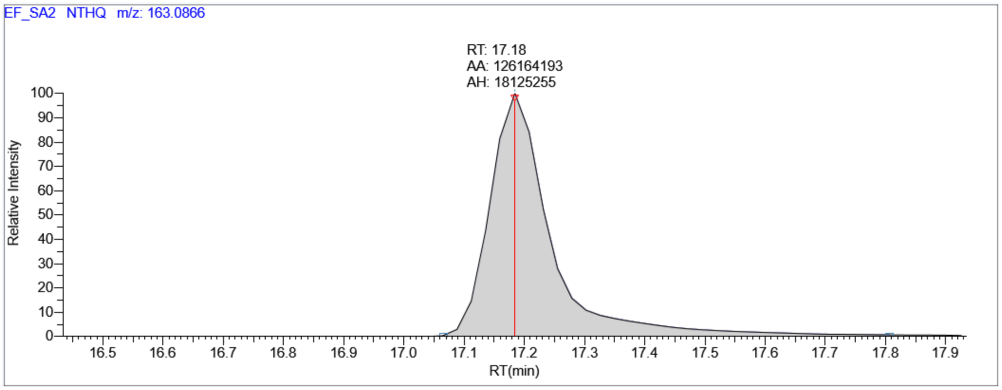
EF_SA2 NTHQ m/z: 163.0866
RT: 17.18
AA: 126164193
AH: 18125255
Relative Intensity
RT(min)

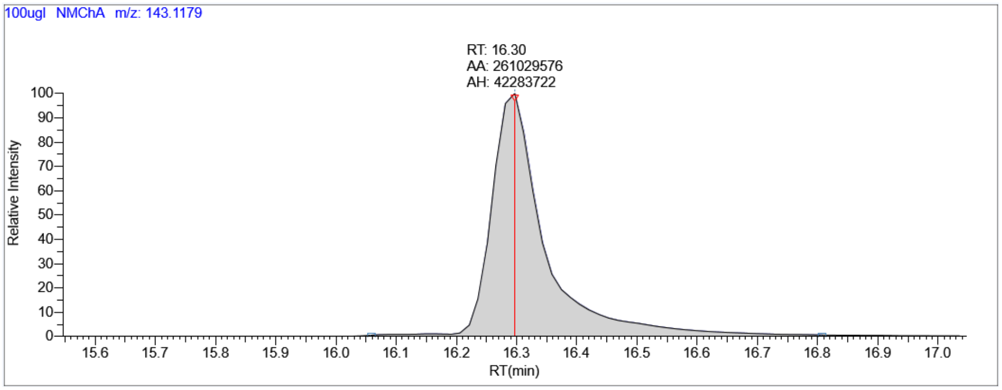
100ugl NMChA m/z: 143.1179
RT: 16.30
AA: 261029576
AH: 42283722
Relative Intensity
RT(min)

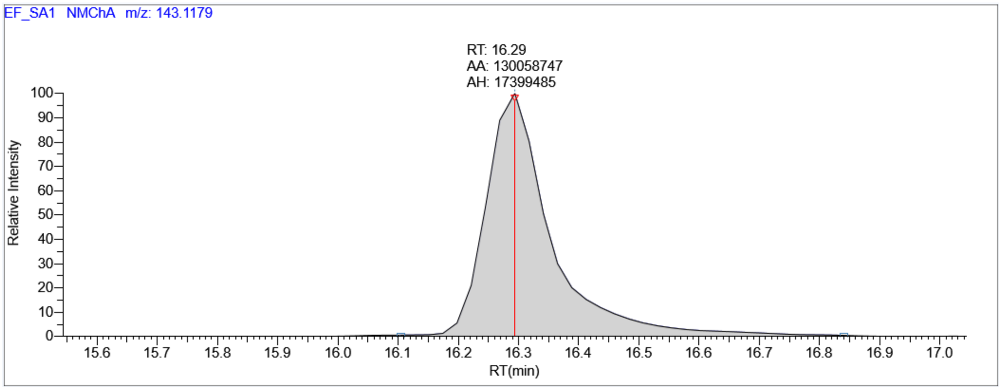
EF_SA1 NMChA m/z: 143.1179
RT: 16.29
AA: 130058747
AH: 17399485
Relative Intensity
RT(min)

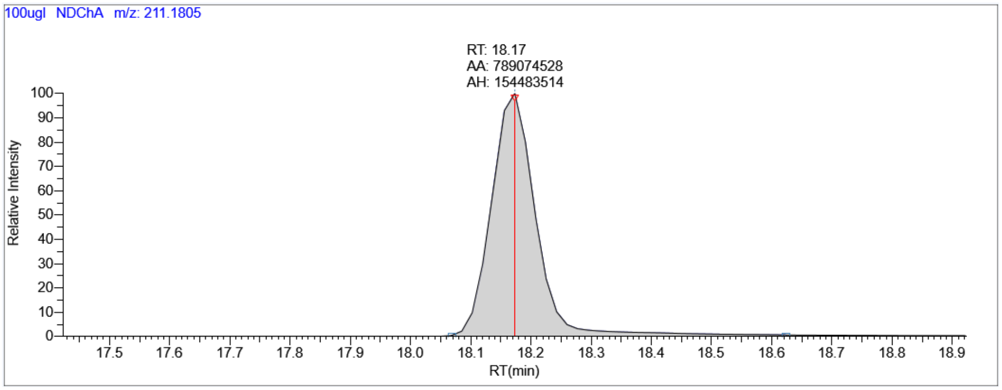
100ugl NDChA m/z: 211.1805
RT: 18.17
AA: 789074528
AH: 154483514
Relative Intensity
RT(min)

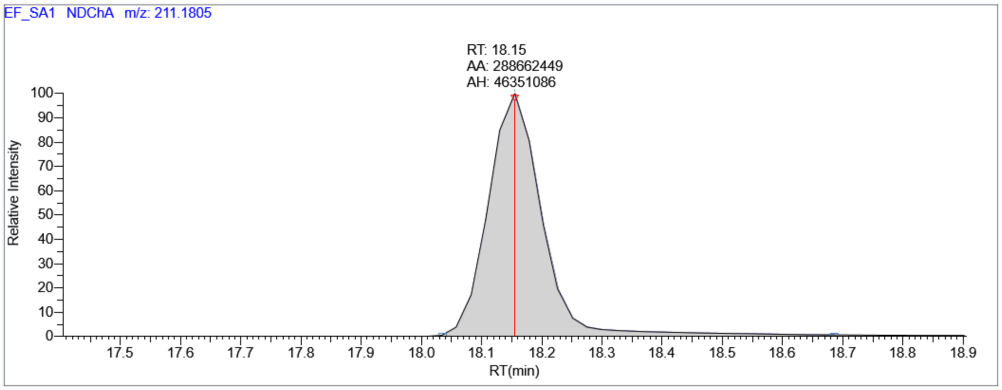
EF_SA1 NDChA m/z: 211.1805
RT: 18.15
AA: 288662449
AH: 46351086
Relative Intensity
RT(min)

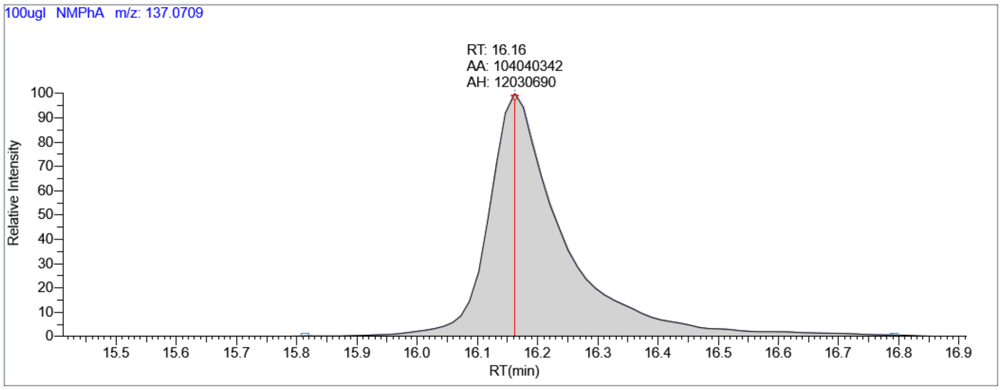

100ugl NMPhA m/z: 137.0709
RT: 16.16
AA: 104040342
AH: 12030690
Relative Intensity
RT(min)

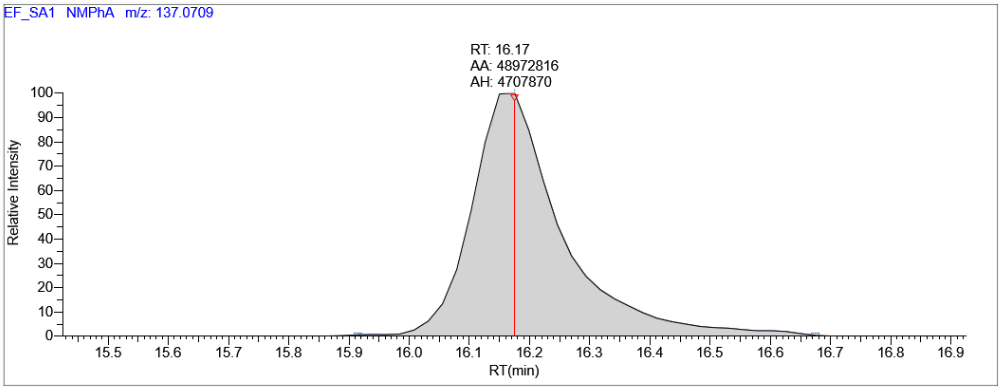

EF_SA1 NMPhA m/z: 137.0709
RT: 16.17
AA: 48972816
AH: 4707870
Relative Intensity
RT(min)

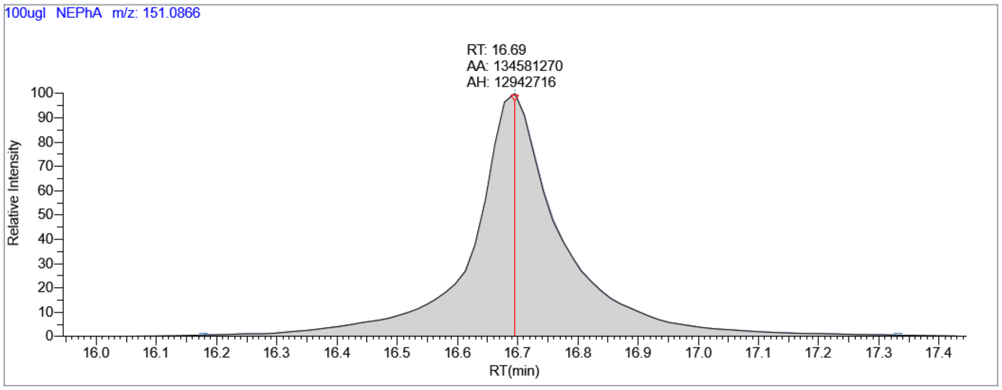

100ugl NEPhA m/z: 151.0866
RT: 16.69
AA: 134581270
AH: 12942716
Relative Intensity
RT(min)

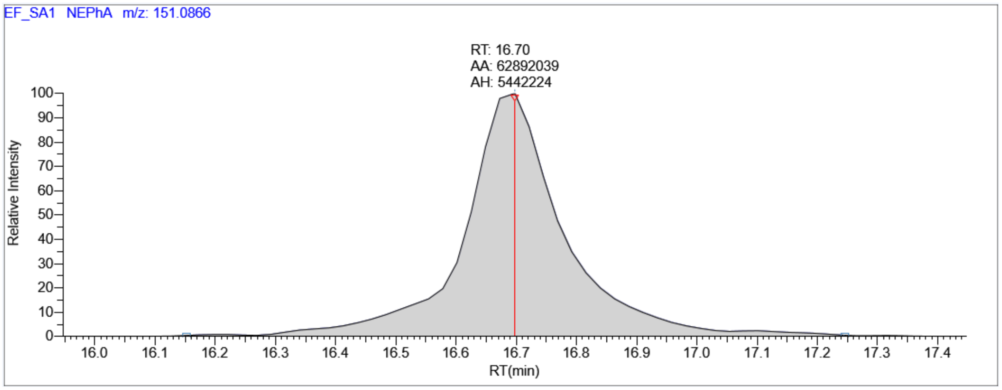

EF_SA1 NEPhA m/z: 151.0866
RT: 16.70
AA: 62892039
AH: 5442224
Relative Intensity
RT(min)

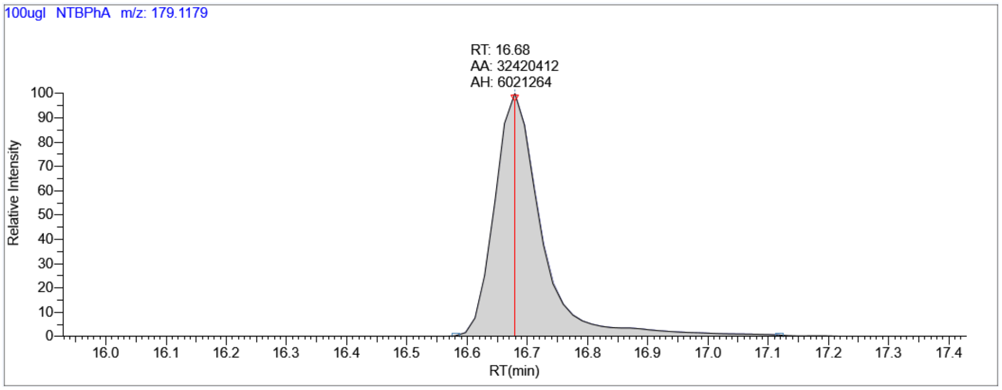
100ugl NTBPhA m/z: 179.1179
RT: 16.68
AA: 32420412
AH: 6021264
Relative Intensity
RT(min)

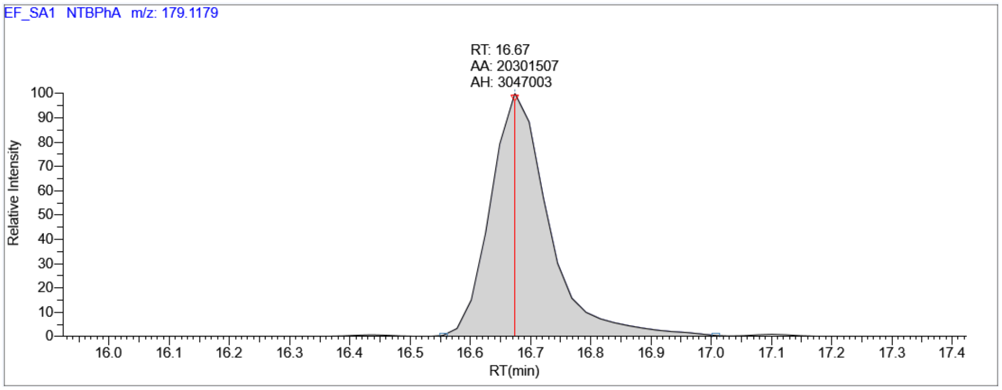
EF_SA1 NTBPhA m/z: 179.1179
RT: 16.67
AA: 20301507
AH: 3047003
Relative Intensity
RT(min)

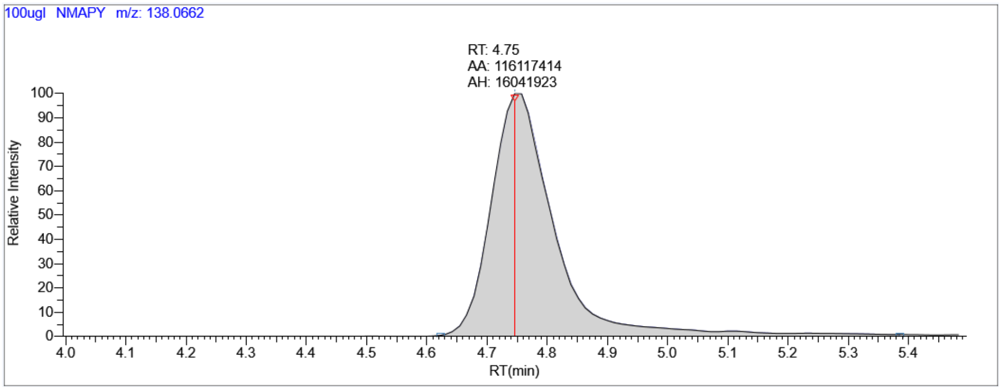
100ugl NMAPY m/z: 138.0662
RT: 4.75
AA: 116117414
AH: 16041923
Relative Intensity
RT(min)

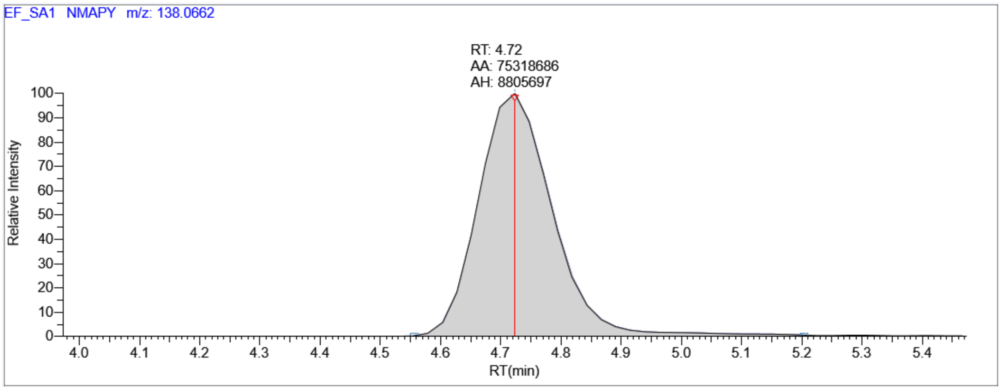
EF_SA1 NMAPY m/z: 138.0662
RT: 4.72
AA: 75318686
AH: 8805697
Relative Intensity
RT(min)

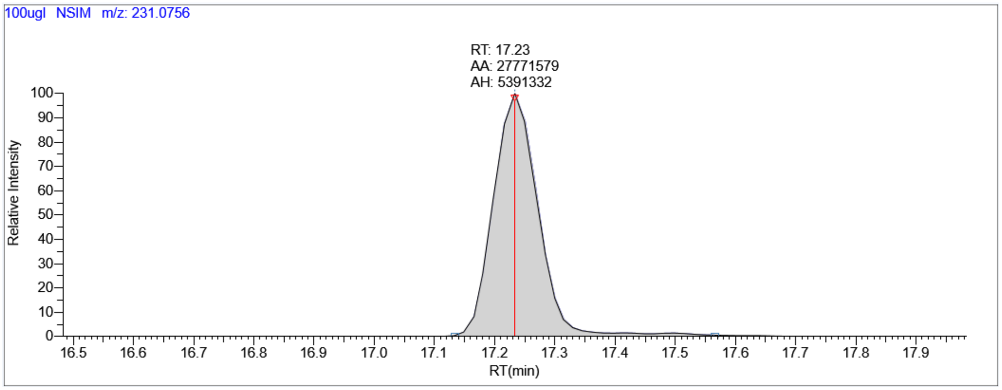
100ugl NSIM m/z: 231.0756
RT: 17.23
AA: 27771579
AH: 5391332
Relative Intensity
RT(min)

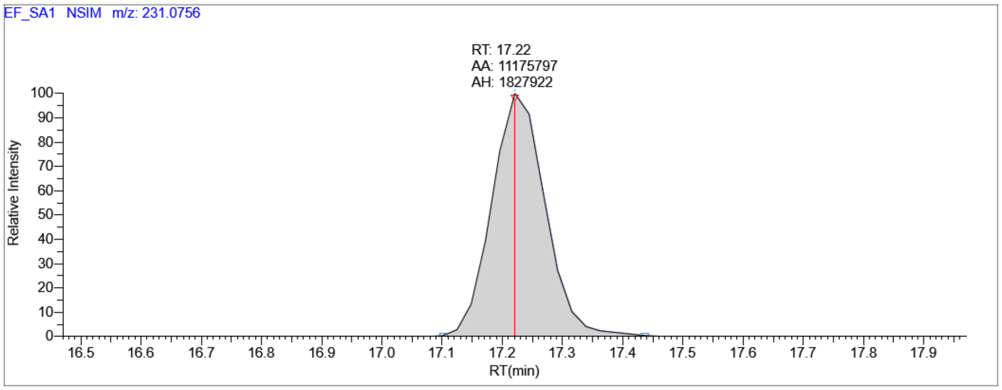
EF_SA1 NSIM m/z: 231.0756
RT: 17.22
AA: 11175797
AH: 1827922
Relative Intensity
RT(min)

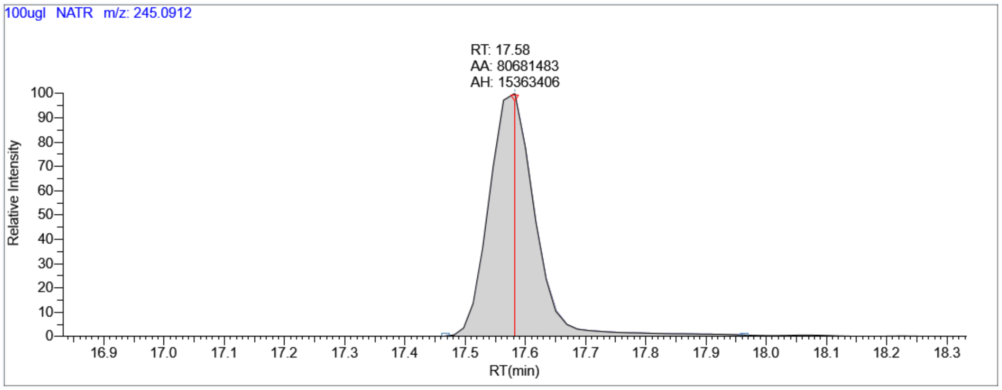
100ugl NATR m/z: 245.0912
RT: 17.58
AA: 80681483
AH: 15363406
Relative Intensity
RT(min)

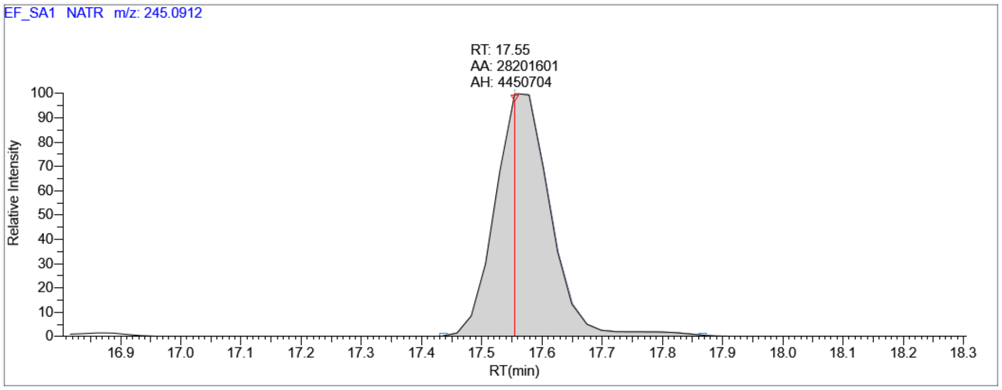
EF_SA1 NATR m/z: 245.0912
RT: 17.55
AA: 28201601
AH: 4450704
Relative Intensity
RT(min)

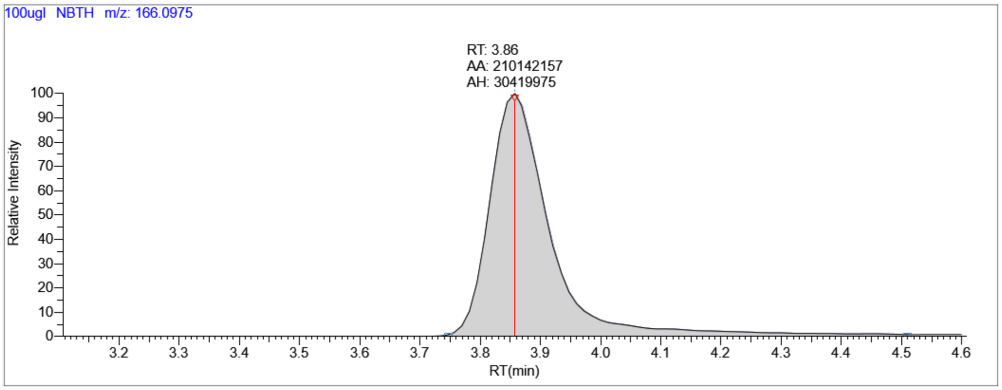

100ugl NBTH m/z: 166.0975
RT: 3.86
AA: 210142157
AH: 30419975
Relative Intensity
RT(min)

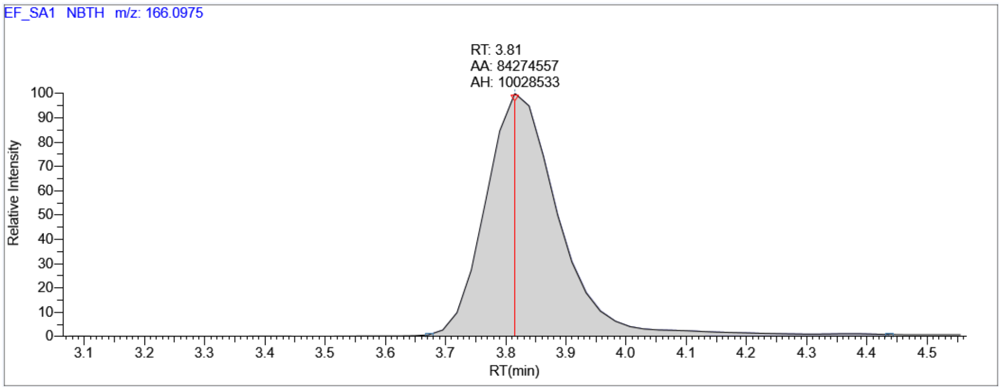

EF_SA1 NBTH m/z: 166.0975
RT: 3.81
AA: 84274557
AH: 10028533
Relative Intensity
RT(min)

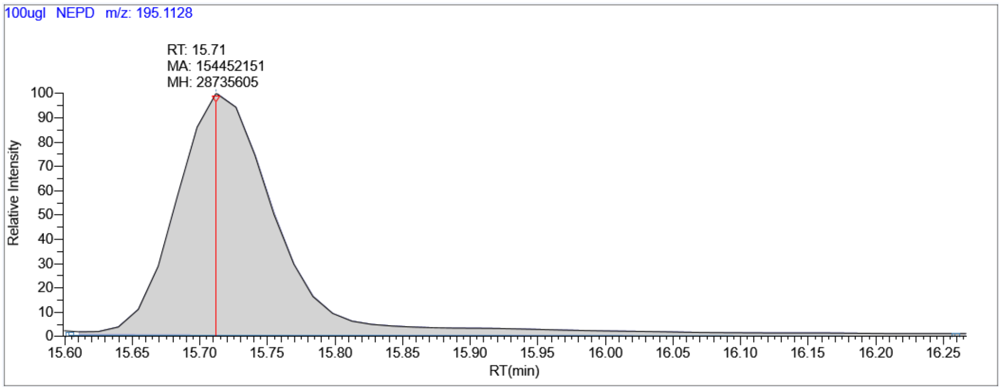

100ugl NEPD m/z: 195.1128
RT: 15.71
MA: 154452151
MH: 28735605
Relative Intensity
RT(min)

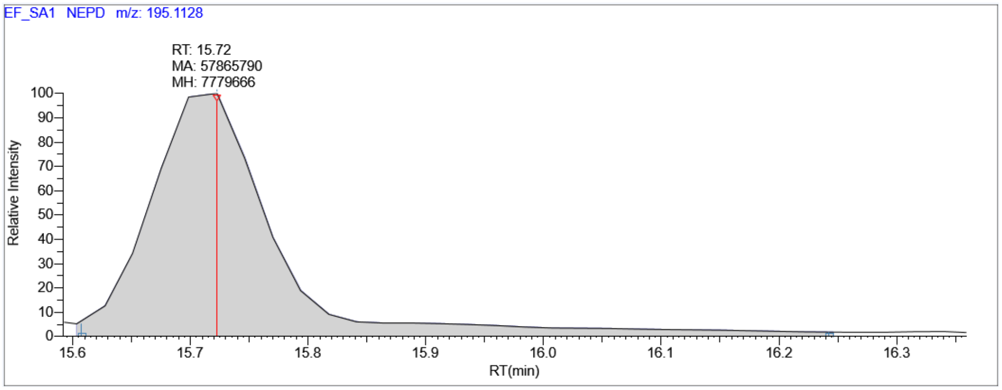

EF_SA1 NEPD m/z: 195.1128
RT: 15.72
MA: 57865790
MH: 7779666
Relative Intensity
RT(min)

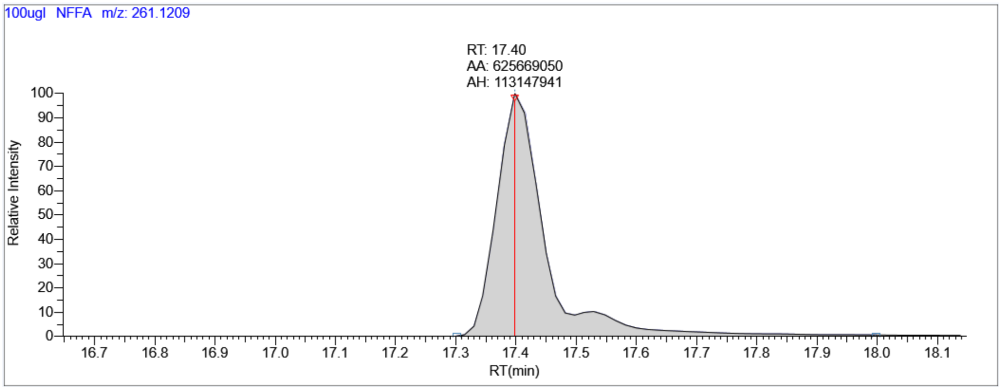
100ugl NFFA m/z: 261.1209
RT: 17.40
AA: 625669050
AH: 113147941
Relative Intensity
RT(min)

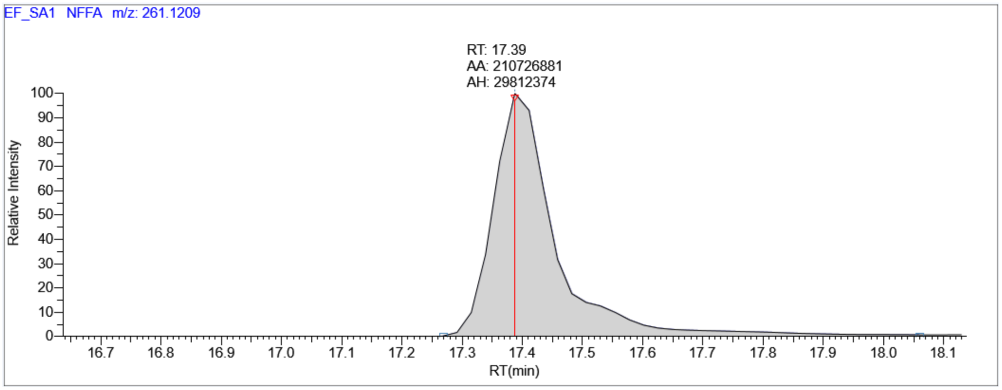
EF_SA1 NFFA m/z: 261.1209
RT: 17.39
AA: 210726881
AH: 29812374
Relative Intensity
RT(min)

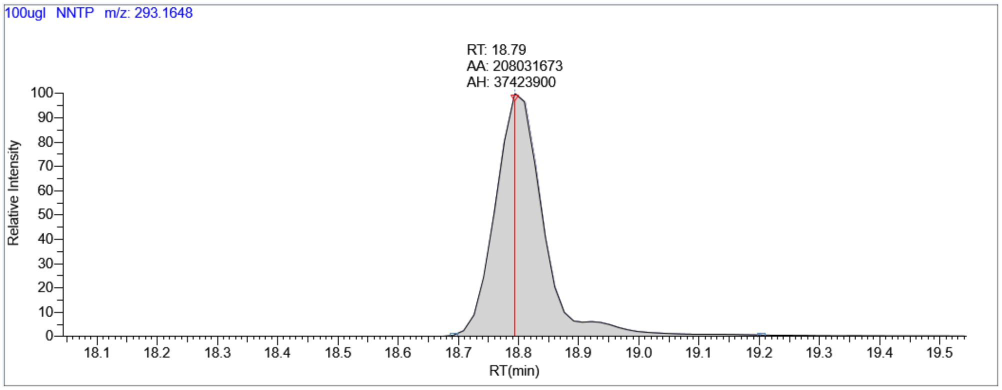
100ugl NNTP m/z: 293.1648
RT: 18.79
AA: 208031673
AH: 37423900
Relative Intensity
RT(min)

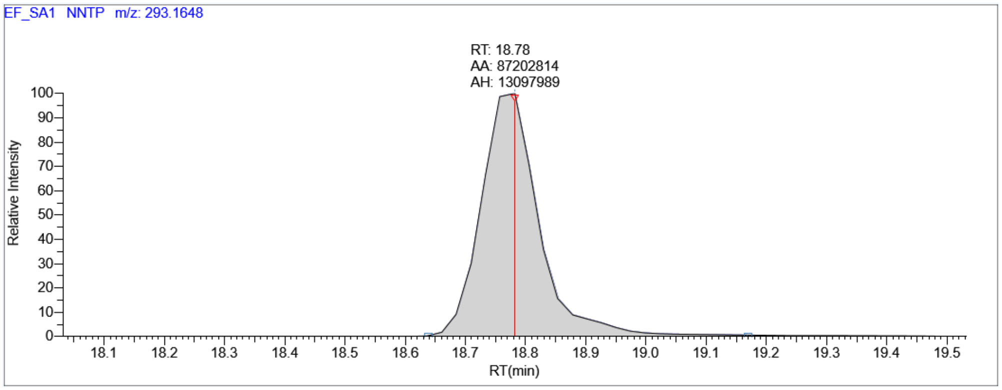
EF_SA1 NNTP m/z: 293.1648
RT: 18.78
AA: 87202814
AH: 13097989
Relative Intensity
RT(min)

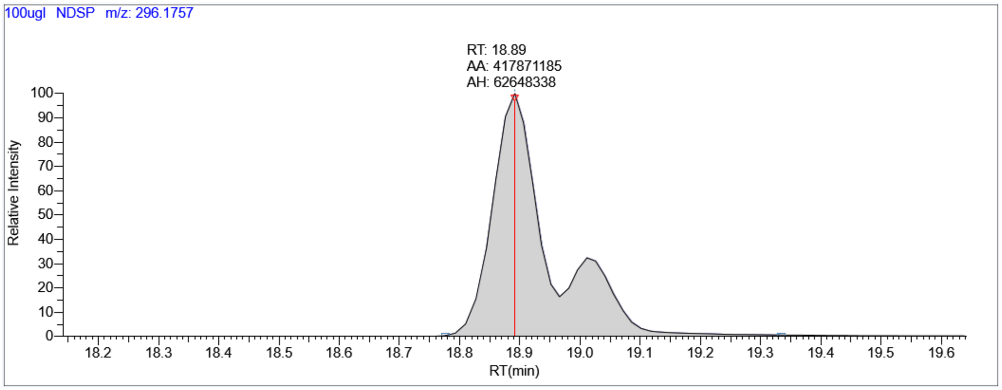
100ugl NDSP m/z: 296.1757
RT: 18.89
AA: 417871185
AH: 62648338
Relative Intensity
RT(min)

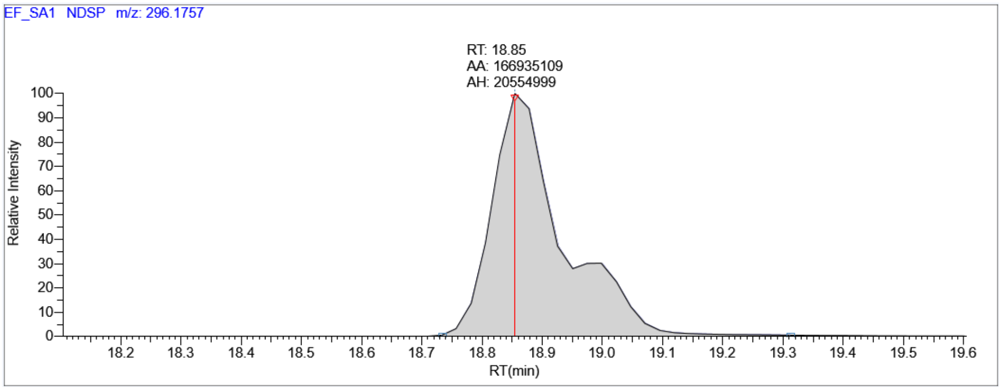
EF_SA1 NDSP m/z: 296.1757
RT: 18.85
AA: 166935109
AH: 20554999
Relative Intensity
RT(min)

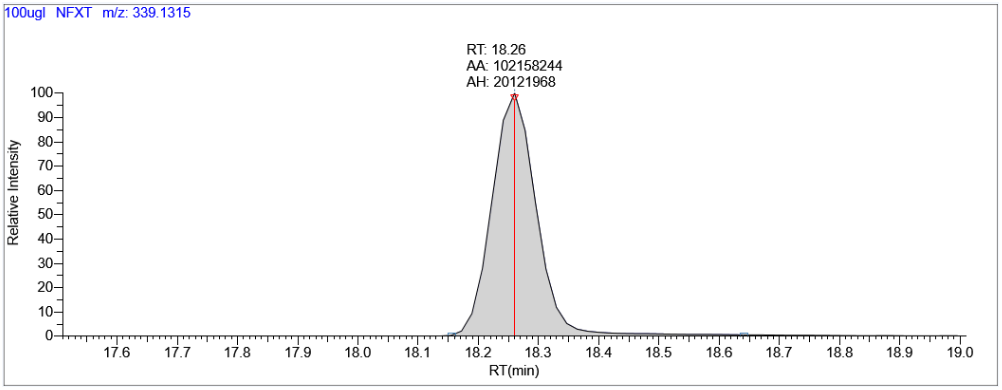
100ugl NFXT m/z: 339.1315
RT: 18.26
AA: 102158244
AH: 20121968
Relative Intensity
RT(min)

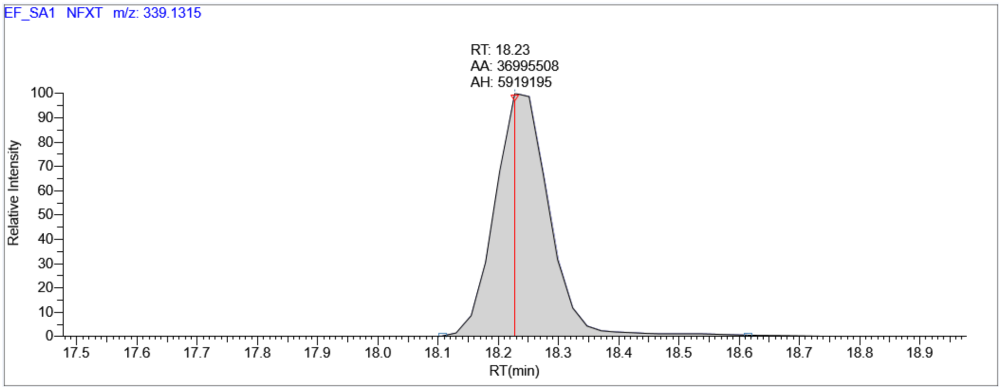
EF_SA1 NFXT m/z: 339.1315
RT: 18.23
AA: 36995508
AH: 5919195
Relative Intensity
RT(min)

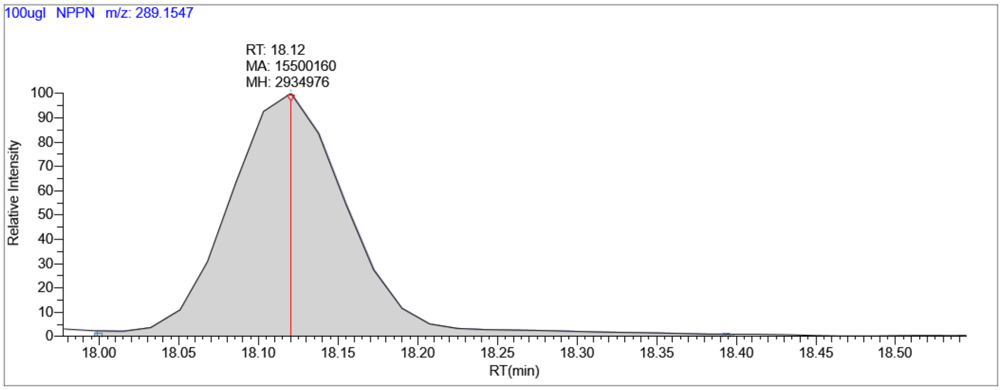
100ugl NPPN m/z: 289.1547
RT: 18.12
MA: 15500160
MH: 2934976
Relative Intensity
RT(min)

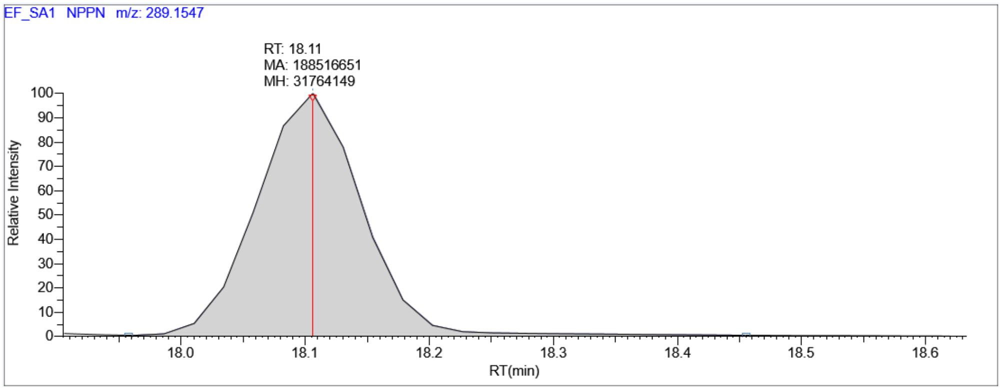
EF_SA1 NPPN m/z: 289.1547
RT: 18.11
MA: 188516651
MH: 31764149
Relative Intensity
RT(min)

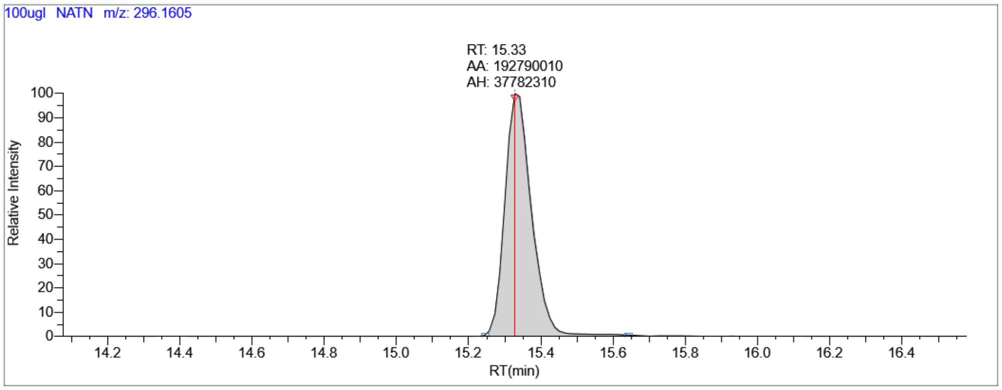
100ugl NATN m/z: 296.1605
RT: 15.33
AA: 192790010
AH: 37782310
Relative Intensity
RT(min)

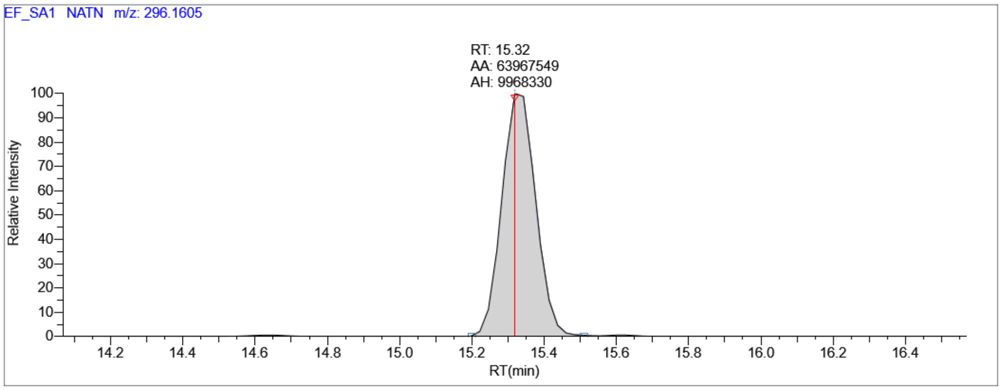
EF_SA1 NATN m/z: 296.1605
RT: 15.32
AA: 63967549
AH: 9968330
Relative Intensity
RT(min)

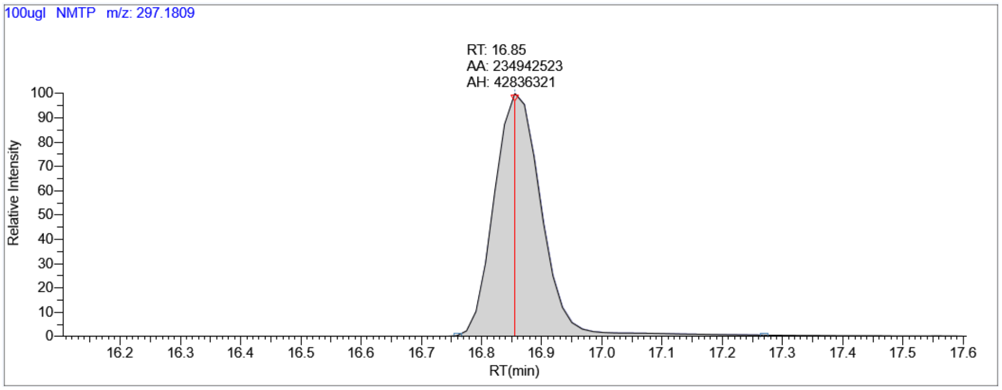
100ugl NMTP m/z: 297.1809
RT: 16.85
AA: 234942523
AH: 42836321
Relative Intensity
RT(min)

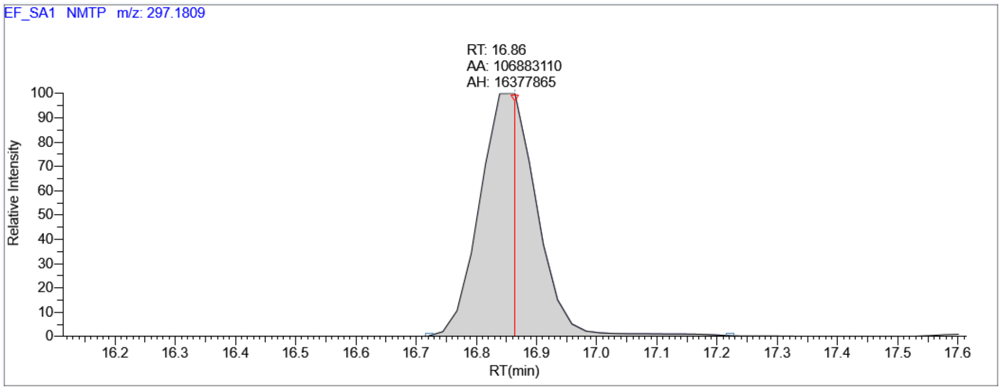
EF_SA1 NMTP m/z: 297.1809
RT: 16.86
AA: 106883110
AH: 16377865
Relative Intensity
RT(min)

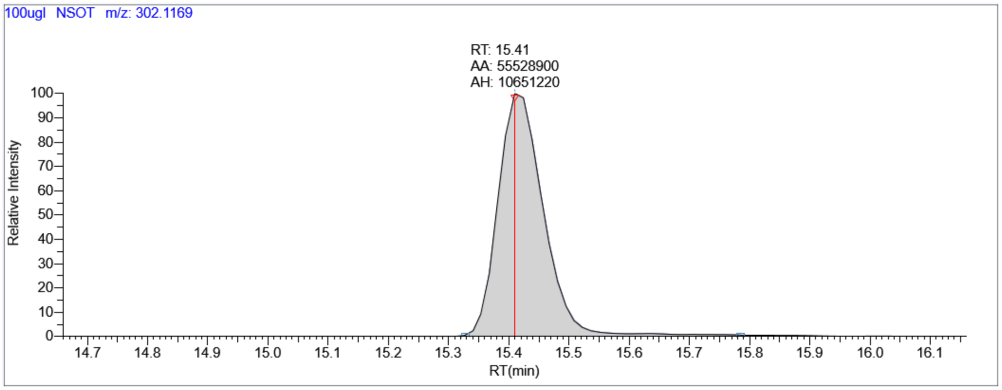
100ugl NSOT m/z: 302.1169
RT: 15.41
AA: 55528900
AH: 10651220
Relative Intensity
RT(min)

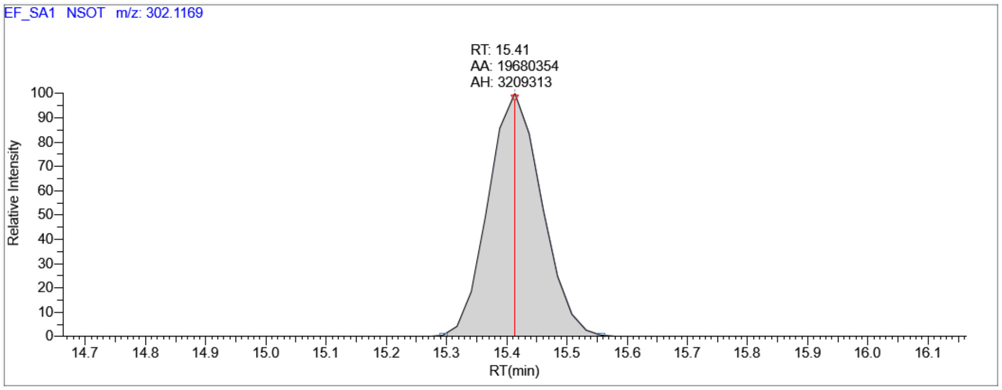
EF_SA1 NSOT m/z: 302.1169
RT: 15.41
AA: 19680354
AH: 3209313
Relative Intensity
RT(min)

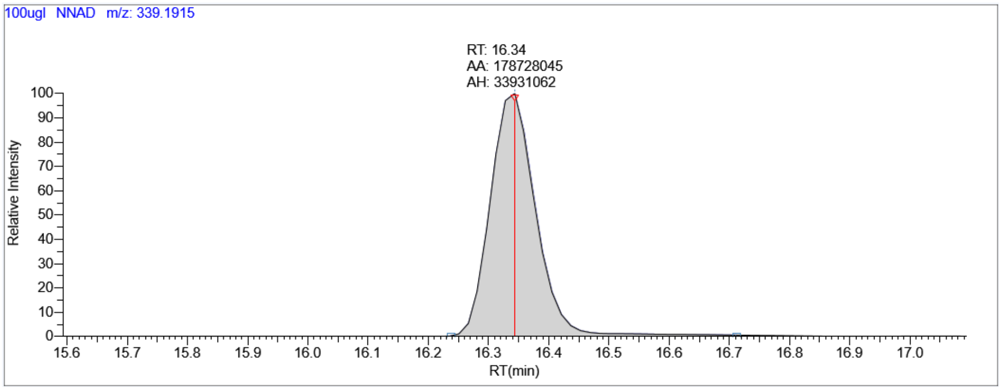
100ugl NNAD m/z: 339.1915
RT: 16.34
AA: 178728045
AH: 33931062
Relative Intensity
RT(min)

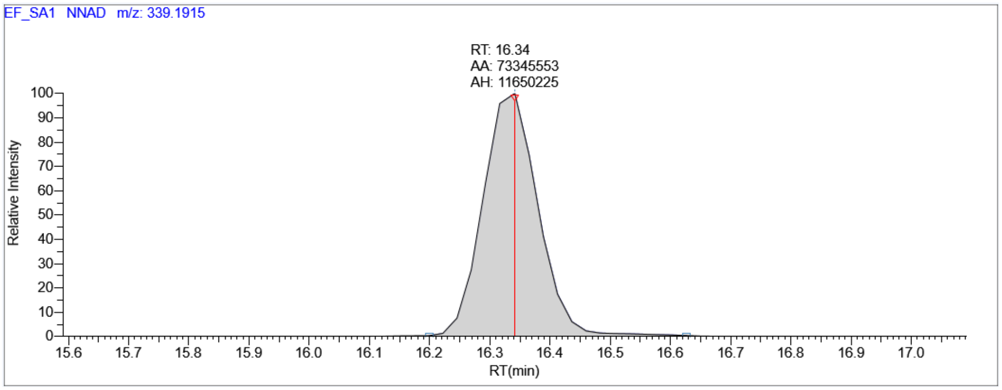
EF_SA1 NNAD m/z: 339.1915
RT: 16.34
AA: 73345553
AH: 11650225
Relative Intensity
RT(min)

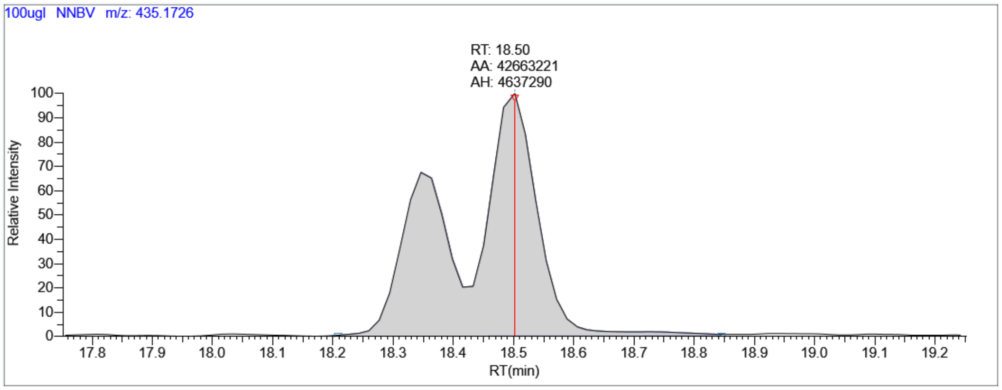
100ugl NNBV m/z: 435.1726
RT: 18.50
AA: 42663221
AH: 4637290
Relative Intensity
RT(min)

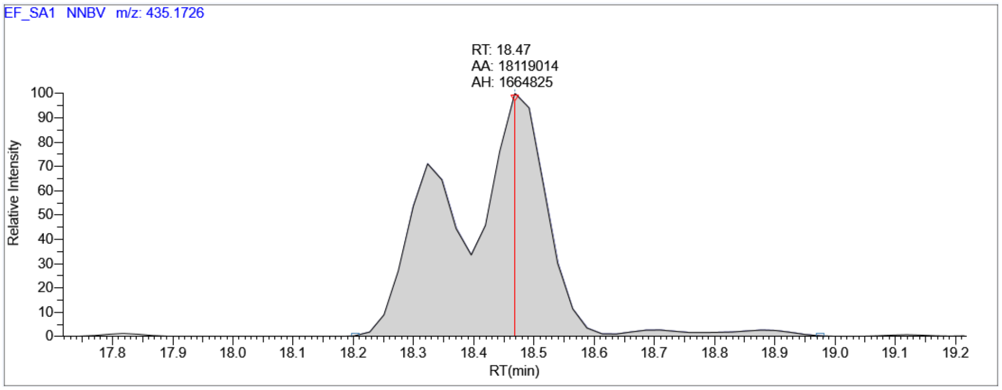
EF_SA1 NNBV m/z: 435.1726
RT: 18.47
AA: 18119014
AH: 1664825
Relative Intensity
RT(min)

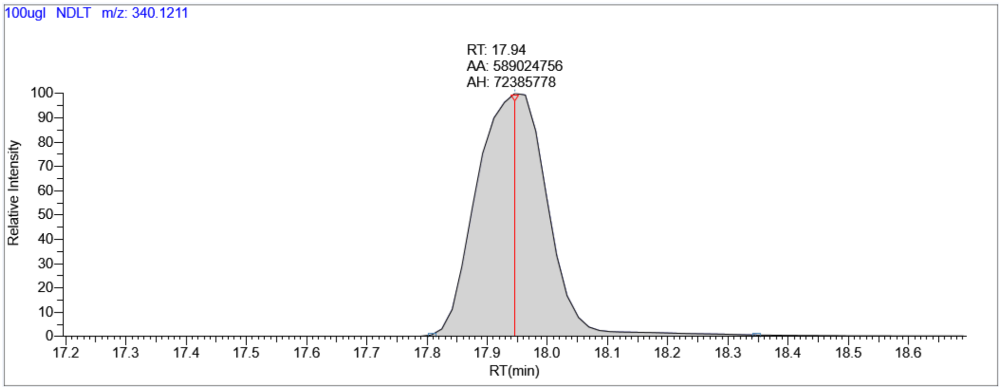
100ugl NDLT m/z: 340.1211
RT: 17.94
AA: 589024756
AH: 72385778
Relative Intensity
RT(min)

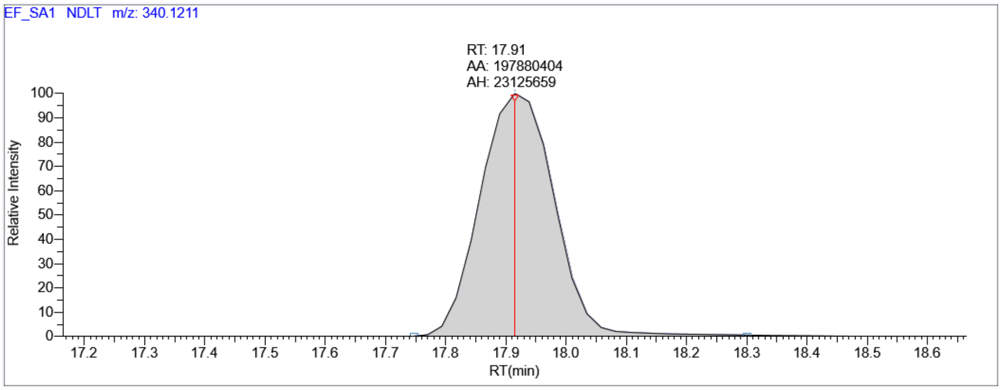
EF_SA1 NDLT m/z: 340.1211
RT: 17.91
AA: 197880404
AH: 23125659
Relative Intensity
RT(min)

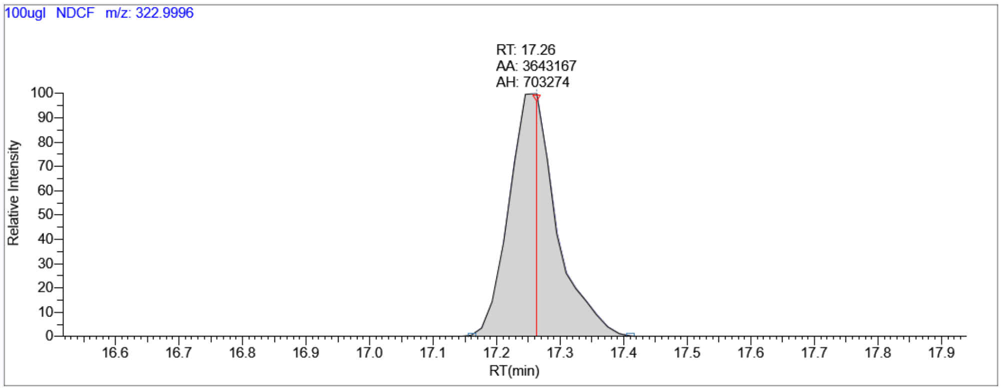
100ugl NDCF m/z: 322.9996
RT: 17.26
AA: 3643167
AH: 703274
Relative Intensity
RT(min)

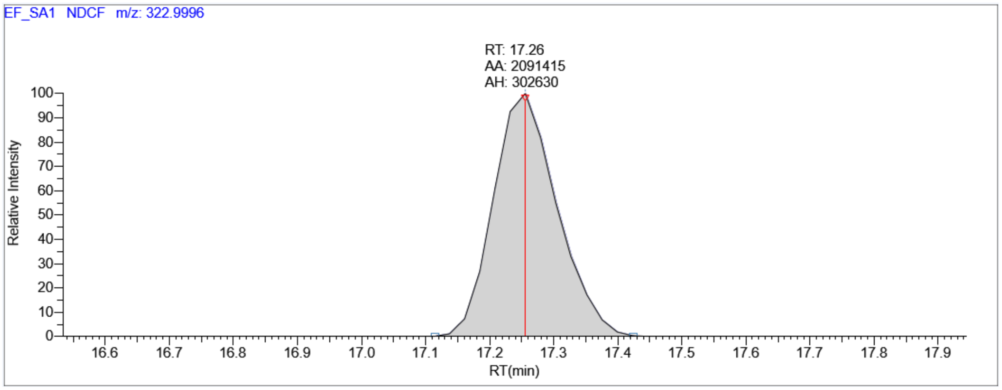
EF_SA1 NDCF m/z: 322.9996
RT: 17.26
AA: 2091415
AH: 302630
Relative Intensity
RT(min)

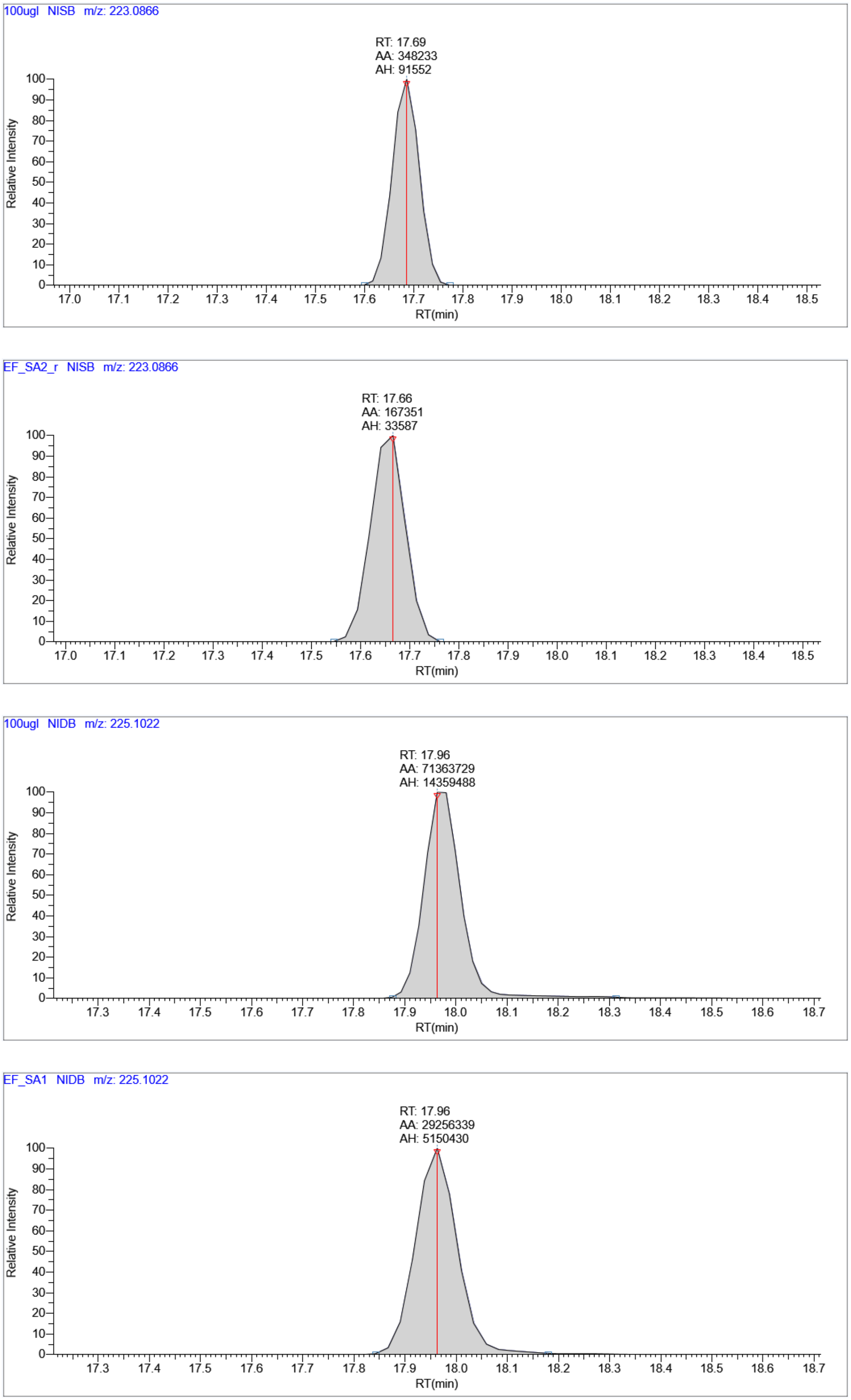

Figure S14. Comparison of LC-HRMS chromatograms of *N*-nitrosamines measured in a standard (top) and a spiked wastewater sample (bottom).

References

(1) Schreiber, I. M.; Mitch, W. A. Influence of the order of reagent addition on NDMA formation during chloramination. *Environmental Science & Technology* **2005**, *39* (10), 3811-3818.
(2) Hausladen, A.; Rafikov, R.; Angelo, M.; Singel, D. J.; Nudler, E.; Stamler, J. S. Assessment of nitric oxide signals by triiodide chemiluminescence. *Proceedings of the National Academy of Sciences* **2007**, *104* (7), 2157-2162.
(3) Sander, S. P.; Abbatt, J. P. D.; Barker, J. R.; Burkholder, J. B.; Friedl, R. R.; Golden, D. M.; Huie, R. E.; Kolb, C. E.; Kurylo, M. J.; Moortgat, G. K.; Orkin, V. L.; Wine, P. H. *Chemical kinetics and photochemical data for use in atmospheric studies: Evaluation number 17*; JPL Publication 10-6 Jet Propulsion Laboratory, Pasadena, CA, 2011. http://jpldataeval.jpl.nasa.gov/pdf/JPL%2010-6%20Final%2015June2011.pdf (accessed April 10, 2023).
(4) Kulshrestha, P.; McKinstry, K. C.; Fernandez, B. O.; Feelisch, M.; Mitch, W. A. Application of an optimized total *N*-nitrosamine (TONO) assay to pools: Placing *N*-nitrosodimethylamine (NDMA) determinations into perspective. *Environmental Science & Technology* **2010**, *44* (9), 3369-3375.
(5) Breider, F.; von Gunten, U. Quantification of total *N*-nitrosamine concentrations in aqueous samples via UV-photolysis and chemiluminescence detection of nitric oxide. *Analytical Chemistry* **2017**, *89* (3), 1574-1582.
(6) International Conference on Harmonization. *ICH Harmonized Tripartite Guideline. Validation of Analytical Procedures: Text and Methodology Q2(R1). Current Step 4 version*; International Conference on Harmonization, Geneva, Switzerland, 2015. https://database.ich.org/sites/default/files/Q2%28R1%29%20Guideline.pdf (accessed April 10, 2023).
(7) Baliga, B. T. Kinetics of Fischer-Hepp rearrangement. *The Journal of Organic Chemistry* **1970**, *35* (6), 2031-2032.
(8) Keefer, L. K.; Hrabie, J. A.; Hilton, B. D.; Wilbur, D. Nitrogen protonation of *N*-nitrosodimethylamine. *Journal of the American Chemical Society* **1988**, *110* (22), 7459-7462.
(9) Feelisch, M.; Rassaf, T.; Mnaimneh, S.; Singh, N.; Bryan, N. S.; Jourd'Heuil, D.; Kelm, M. Concomitant *S*-, *N*-, and heme-nitros(yl)ation in biological tissues and fluids: Implications for the fate of NO *in vivo*. *The FASEB Journal* **2002**, *16* (13), 1775-1785.
(10) Beard, J. C.; Swager, T. M. An organic chemist's guide to *N*-nitrosamines: Their structure, reactivity, and role as contaminants. *The Journal of Organic Chemistry* **2021**, *86* (3), 2037-2057.
(11) Polo, J.; Chow, Y. L. Efficient photolytic degradation of nitrosamines. *JNCI: Journal of the National Cancer Institute* **1976**, *56* (5), 997-1001.
(12) Bolton, J. R.; Stefan, M. I. Fundamental photochemical approach to the concepts of fluence (UV dose) and electrical energy efficiency in photochemical degradation reactions. *Research on Chemical Intermediates* **2002**, *28* (7), 857-870.
(13) St. John, P. C.; Guan, Y.; Kim, Y.; Kim, S.; Paton, R. S. Prediction of organic homolytic bond dissociation enthalpies at near chemical accuracy with sub-second computational cost. *Nature Communications* **2020**, *11* (1), 2328.
(14) Wen, M.; Blau, S. M.; Spotte-Smith, E. W. C.; Dwaraknath, S.; Persson, K. A. BonDNet: A graph neural network for the prediction of bond dissociation energies for charged molecules. *Chemical Science* **2021**, *12* (5), 1858-1868.
(15) ChemAxon. *MarvinSketch (Version 22.19.0)*. ChemAxon, Budapest, Hungary, https://www.chemaxon.com (accessed April 15, 2023).
(16) Mauri, A. alvaDesc: A tool to calculate and analyze molecular descriptors and fingerprints. *Ecotoxicological QSARs* **2020**, 801-820.
(17) Sushko, I.; Novotarskyi, S.; Körner, R.; Pandey, A. K.; Rupp, M.; Teetz, W.; Brandmaier, S.; Abdelaziz, A.; Prokopenko, V. V.; Tanchuk, V. Y.; Todeschini, R.; Varnek, A.; Marcou, G.; Ertl, P.; Potemkin, V.; Grishina, M.; Gasteiger, J.; Schwab, C.; Baskin, I. I.; Palyulin, V. A.; Radchenko, E. V.; Welsh, W. J.; Kholodovych, V.; Chekmarev, D.; Cherkasov, A.; Aires-de-Sousa, J.; Zhang, Q.-Y.; Bender, A.; Nigsch, F.; Patiny, L.; Williams, A.; Tkachenko, V.; Tetko, I. V. Online chemical modeling environment (OCHEM): Web platform for data storage,

model development and publishing of chemical information. *Journal of Computer-Aided Molecular Design* **2011**, *25* (6), 533-554.
(18) Moriwaki, H.; Tian, Y.-S.; Kawashita, N.; Takagi, T. Mordred: A molecular descriptor calculator. *Journal of Cheminformatics* **2018**, *10* (1), 4.
(19) Yap, C. W. PaDEL-descriptor: An open source software to calculate molecular descriptors and fingerprints. *Journal of Computational Chemistry* **2011**, *32* (7), 1466-1474.
(20) Friedman, J. H.; Hastie, T.; Tibshirani, R. Regularization paths for generalized linear models via coordinate descent. *Journal of Statistical Software* **2010**, *33* (1), 1 - 22.
(21) Hebbali, A. olsrr: Tools for Building OLS Regression Models. **2022**, (R package version 0.6.0).
(22) Todeschini, R.; Consonni, V. *Molecular Descriptors for Chemoinformatics*; WILEY-VCH Verlag GmbH & Co. KGaA, 2009. DOI: 10.1002/9783527628766.
(23) U.S. Environmental Protection Agency. *Definition and Procedure for the Determination of the Method Detection Limit, Revision 2*; EPA 821-R-16-006; U.S. Environmental Protection Agency, Washington, DC, 2016. https://www.epa.gov/sites/default/files/2016-12/documents/mdl-procedure_rev2_12-13-2016.pdf (accessed April 10, 2023).
(24) U.S. Army Engineer Research and Development Center. *Analytical Chemistry Detection Limits and the Evaluation of Dredged Sediments*; ADA430418; U.S. Army Engineer Research and Development Center, Vicksburg, MS, 2005. https://apps.dtic.mil/sti/pdfs/ADA430418.pdf (accessed April 10, 2023).
(25) Moschet, C.; Piazzoli, A.; Singer, H. P.; Hollender, J. Alleviating the reference standard dilemma using a systematic exact mass suspect screening approach with liquid chromatography-high resolution mass spectrometry. *Analytical Chemistry* **2013**, *85* (21), 10312-10320.
(26) Mansouri, K.; Grulke, C. M.; Judson, R. S.; Williams, A. J. OPERA models for predicting physicochemical properties and environmental fate endpoints. *Journal of Cheminformatics* **2018**, *10* (1), Article Number: 10.
(27) ChemAxon. *JChem for Excel (Version 20.20.0.732)*. ChemAxon, Budapest, Hungary, https://www.chemaxon.com (accessed April 15, 2023).
(28) Meints, D. A. Biofilm-related materials as total *N*-nitrosamine (TONO) precursors and hydroxylamine-based interferences in TONO and *N*-nitrosodimethylamine (NDMA) measurements. M.S. Thesis, University of Arkansas, 2015. https://scholarworks.uark.edu/etd/1124/ (accessed April 10, 2023).
(29) Krauss, M.; Hollender, J. Analysis of nitrosamines in wastewater: Exploring the trace level quantification capabilities of a hybrid linear ion trap/Orbitrap mass spectrometer. *Analytical Chemistry* **2008**, *80* (3), 834-842.
(30) Dai, N.; Zeng, T.; Mitch, W. A. Predicting *N*-nitrosamines: *N*-Nitrosodiethanolamine as a significant component of total *N*-nitrosamines in recycled wastewater. *Environmental Science & Technology Letters* **2015**, *2* (3), 54-58.
(31) Zeng, T.; Mitch, W. A. Contribution of *N*-nitrosamines and their precursors to domestic sewage by greywaters and blackwaters. *Environmental Science & Technology* **2015**, *49* (22), 13158-13167.
